# Aging-Associated Alterations in Mammary Epithelia and Stroma Revealed by Single-Cell RNA Sequencing

**DOI:** 10.1016/j.celrep.2020.108566

**Published:** 2020-12-29

**Authors:** Carman Man-Chung Li, Hana Shapiro, Christina Tsiobikas, Laura M. Selfors, Huidong Chen, Jennifer Rosenbluth, Kaitlin Moore, Kushali P. Gupta, G. Kenneth Gray, Yaara Oren, Michael J. Steinbaugh, Jennifer L. Guerriero, Luca Pinello, Aviv Regev, Joan S. Brugge

**Affiliations:** 1 Department of Cell Biology, Harvard Medical School, Boston, MA 02115, USA; 2 Broad Institute of MIT and Harvard, Cambridge, MA 02142, USA; 3 Molecular Pathology Unit & Cancer Center, Massachusetts General Hospital Research Institute and Harvard Medical School, Charlestown, MA 02129, USA; 4 Department of Biostatistics, Harvard T.H. Chan School of Public Health, Boston, MA 02115, USA; 5 Breast Tumor Immunology Laboratory, Dana-Farber Cancer Institute, Boston, MA 02115, USA; 6 Division of Breast Surgery, Department of Surgery, Brigham and Women’s Hospital, Boston, MA 02115, USA; 7 Present address: Genentech, 1 DNA Way, South San Francisco, CA 94080, USA; 8 Lead Contact

## Abstract

Aging is closely associated with increased susceptibility to breast cancer, yet there have been limited systematic studies of aging-induced alterations in the mammary gland. Here, we leverage high-throughput single-cell RNA sequencing to generate a detailed transcriptomic atlas of young and aged murine mammary tissues. By analyzing epithelial, stromal, and immune cells, we identify age-dependent alterations in cell proportions and gene expression, providing evidence that suggests alveolar maturation and physiological decline. The analysis also uncovers potential pro-tumorigenic mechanisms coupled to the age-associated loss of tumor suppressor function and change in microenvironment. In addition, we identify a rare, age-dependent luminal population co-expressing hormone-sensing and secretory-alveolar lineage markers, as well as two macrophage populations expressing distinct gene signatures, underscoring the complex heterogeneity of the mammary epithelia and stroma. Collectively, this rich single-cell atlas reveals the effects of aging on mammary physiology and can serve as a useful resource for understanding aging-associated cancer risk.

## In Brief

Using single-cell RNA-sequencing, Li et al. compare mammary epithelia and stroma in young and aged mice. Age-dependent changes at cell and gene levels provide evidence suggesting alveolar maturation, functional deterioration, and potential pro-tumorigenic and inflammatory alterations. Additionally, identification of heterogeneous luminal and macrophage subpopulations underscores the complexity of mammary lineages.

## Graphical Abstract

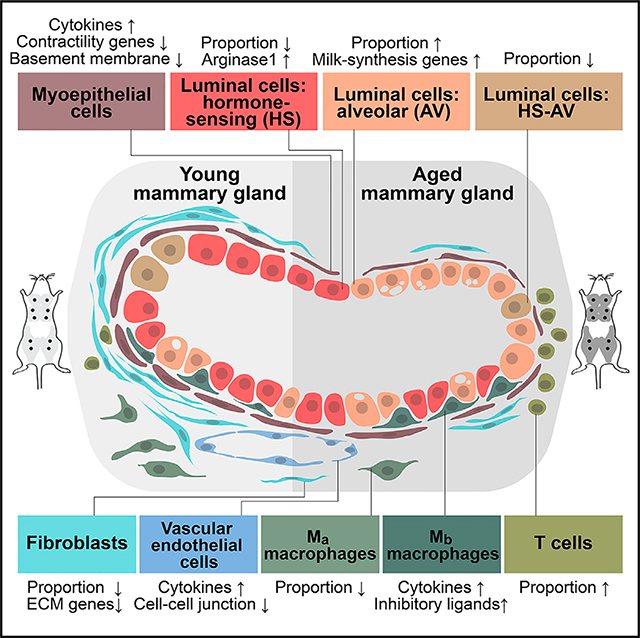

## INTRODUCTION

The mammary gland undergoes dynamic changes in cellular composition and gene expression over a lifetime (Inman et al., 2015). Although recent single-cell RNA sequencing (scRNA-seq) studies have characterized changes during embryonic development, puberty, and pregnancy (Bach et al., 2017; Giraddi et al., 2018; Pal et al., 2017; Wuidart et al., 2018), little is known about the alterations associated with aging, an important aspect of mammary development closely related to breast cancer (Jenkins et al., 2014). Although mammography, histology, and molecular analyses have revealed prominent changes with age, including decreased mammographic density, altered epithelial proportions, and reduced connective tissue (Azam et al., 2019; Dong et al., 2016; Garbe et al., 2012; Gertig et al., 1999; Hart et al., 1989; Hutson et al., 1985; McCormack et al., 2010; Pelissier Vatter et al., 2018; Pelissier et al., 2014), systematic single-cell transcriptome profiling can better capture age-associated effects at a higher resolution and on a larger scale. Moreover, given that increased age is strongly associated with breast cancer susceptibility in human and mouse models (Jenkins et al., 2014; LaBarge et al., 2016; Raafat et al., 2012), a single-cell atlas for aged mammary glands could help fill the gaps in our knowledge of aging and cancer risk. The use of human samples to study aging has been challenging because of confounding covariates, such as pregnancy history (parity) and accumulated environmental stress (Ginger and Rosen, 2003; Huh et al., 2015; Kamikawa et al., 2009; Russo et al., 2008; Dos Santos et al., 2015; Singletary and McNary, 1992). In contrast, mouse models can complement human studies by providing more-precise control of parity, hormone cycle, genetic background, and environmental factors, thus enabling a focus on age-specific effects.

Mammary gland function depends on both the epithelial and stromal compartments (Inman et al., 2015; Polyak and Kalluri, 2010). The mammary epithelial bilayer contains outer myoepithelial/basal cells and inner luminal cells, which, collectively, form branching ducts and terminal acinus. Myoepithelial cells are distinguished by their contractility and ability to synthesize basement membrane. Luminal cells, on the other hand, consist of two subtypes: hormone-sensing (HS) cells capable of responding to endocrine signals, such as estrogen, progesterone, and prolactin; and secretory alveolar (AV) cells capable of producing milk. HS and AV cells have also been referred to as mature luminal (ML) and luminal progenitor (LP) cells, respectively, based on their *in vitro* colony-forming ability (Fu et al., 2020; Shackleton et al., 2006; Stingl et al., 2006); however, cumulative evidence suggests that these two lineages are independent in adulthood (Lilja et al., 2018; Van Keymeulen et al., 2017; Wang et al., 2017). Surrounding the epithelial cells are various stromal cells, including fibroblasts, vascular/lymphatic cells, immune cells, and adipocytes. Although initial scRNA-seq studies provided valuable knowledge of mammary epithelia in early and adult development (Bach et al., 2017; Giraddi et al., 2018; Nguyen et al., 2018; Pal et al., 2017; Wuidart et al., 2018), characterization of stromal cells has been limited (Han et al., 2018; Kanaya et al., 2019; Tabula Muris Consortium, 2018, 2020).

Here, we used scRNA-seq to analyze young and aged, murine, virgin mammary glands, identifying a significant diversity of epithelial and stromal cell. We captured a rare population of luminal epithelial cells co-expressing HS and AV lineage markers but distinguishable by a unique gene signature. We also detected heterogeneous stromal/immune populations, including distinct macrophage subtypes. In addition, comparison of young and aged cells identified aging-associated changes in cell-type compositions and transcriptomic profiles, providing evidence that suggests alveolar maturation and physiological decline. The analysis also identified potentially pro-tumorigenic alterations that may provide initial insight into mechanisms for age-associated vulnerability to breast cancer. Overall, these observations are highly consistent across multiple samples, suggesting that the intrinsic effects of aging on mammary tissues may follow a defined biological program and are not merely consequences of stochastic deterioration.

## RESULTS

### Diverse Epithelial and Stromal Cell Types Identified by scRNA-Seq in Mammary Glands

**Figure 1.**
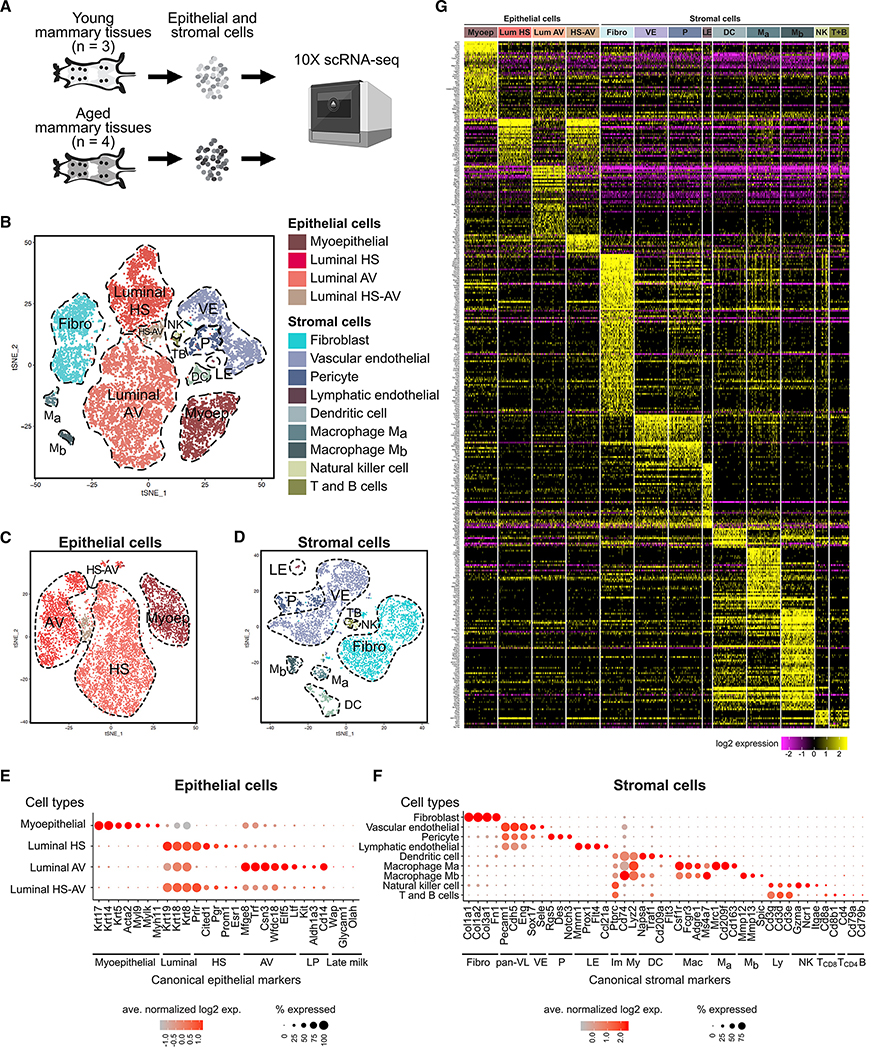
Diverse Epithelial and Stromal Cell Types Identified by scRNA-Seq in Mammary Glands (A) scRNA-seq experimental setup. Freshly dissociated epithelial and stromal cells were analyzed without FACS enrichment. (B) t-SNE plot showing epithelial and stromal clusters identified based on characteristic markers. (C and D) Epithelial (C) and stromal (D) cells subsetted and reclustered. (E and F) Expression of canonical markers of epithelial (E) and stromal (F) cell types. (G) Heatmap of cell type-specific gene signatures. See also Table S1. Abbreviations: Myoep, myoepithelial; HS, hormone-sensing; AV, alveolar; LP, luminal progenitors; Fibro, fibroblasts; pan-VL, pan-vascular/lymphatic cells; VE, vascular endothelial cells; P, pericytes; LE, lymphatic endothelial cells; Im, immune cells; My, myeloid cells; DC, dendritic cells; Mac, macrophages; M_a_, M_a_ macrophages, M_b_, M_b_ macrophages; Ly, lymphocytes; NK, natural killer cells; T, T cells; B, B cells.

To study aging in mammary tissues, we performed scRNA-seq analysis using the 10× Chromium platform on young (3–4 months old, n = 3) and aged (13–14 months old, n = 4), virgin mice (Figure 1A), which correspond to human early adulthood (20–30 years old) and perimenopause (45–55 years old), respectively (Diaz Brinton, 2012; Finch et al., 1984; Flurkey et al., 2007). Mice were analyzed at diestrus, when progesterone levels and epithelial cell proliferation peak, to facilitate detection of progenitor cells (Brisken and Ataca, 2015; Fata et al., 2001; Joshi et al., 2010). With stringent filtering at cell and gene levels to eliminate potential doublets, we captured a total of 13,684 cells and 27,998 genes. Single cells were clustered based on gene expression profiles using Seurat and visualized using t-distributed stochastic neighbor embedding (t-SNE) (Butler et al., 2018; Stuart et al., 2019) (Figures 1B and S1A). Doublet estimation using Scrublet (Wolock et al., 2019) and DoubletFinder (McGinnis et al., 2019) demonstrated that doublets likely accounted for a negligible fraction of our data and did not drive clustering (Figure S1B).

Epithelial and stromal cell types were identified using canonical markers (Figures 1B–1F). As expected, we detected epithelial cells (n = 8,901; expressing *Epcam*) of myoepithelial lineage (n = 1,670; expressing *Krt17*, *Krt14*, *Krt5*, *Acta2*, *Myl9*, *Mylk*, and *Myh11*) or luminal lineage (n = 7,231; expressing *Krt19*, *Krt18*, and *Krt8*) (Figure 1E). Luminal cells consisted of both HS cells (n = 2,138; expressing *Prlr*, *Pgr*, *Esr1*, *Cited1*, and *Prom1*) and AV cells (n = 4,820; expressing *Mfge8*, *Trf*, *Csn3*, *Wfdc18*, *Ltf*, and *Elf5*). AV cells expressed luminal progenitor markers (*Kit*, *Aldh1a3*, and *Cd14*) and milk biosynthesis-related genes (*Mfge8*, *Trf*, *Csn3*, *Wfdc18*, and *Ltf*), but not mature alveolar markers associated with pregnancy and lactation (*Wap*, *Glycam1*, and *Olah*) (Figure 1E), consistent with their alveolar progenitor differentiation state in virgin glands (Booth et al., 2007). In addition, we identified a rare luminal population co-expressing hormone-sensing markers and alveolar progenitor markers (n = 273; Figures 1B, 1C, and 1E). We termed these HS-AV cells. This population is unlikely to be doublets based on the doublet analyses above (Figure S1B).

Among stromal cells (n = 4,783), we detected fibroblasts (n = 1,993; expressing *Col1a1*, *Col1a2*, *Col3a1*, and *Fn1*), vascular/lymphatic cells (n = 2,196; expressing *Pecam1*, *Cdh5*, and *Eng*), and immune cells (n = 594; expressing *Ptprc*, which encodes CD45) (Figure 1F). The vascular/lymphatic population consisted of vascular endothelial cells (n = 1,819; expressing *Sox17* and *Sele*), pericytes (n = 349; expressing *Rgs5*, *Des*, and *Notch3*), and lymphatic endothelial cells (n = 28; expressing *Mmrn1*, *Prox1*, *Flt4*, and *Ccl21a*). The immune population contained both myeloid cells (marked by Cd74 and Lyz2) and lymphocytes (marked by *Cd3d*, *Cd3e*, and *Cd3g*). Within myeloid cells, we detected dendritic cells (n = 226; expressing *Napsa*, *Traf1*, *Cd209a*, and *Flt3*) and two populations of macrophages (n = 268; both expressing *Csf1r*, *Fcgr3*, *Adgre1*, and *Ms4a7*, but distinguishable by *Mrc1*, *Cd209f*, and *Cd163* [designated as M_a_ hereafter] or by *Mmp12, Mmp13*, and *Spic* [designated as M_b_]). Within lymphocytes, we detected natural killer (NK) cells (n = 40; expressing *Gzma*, *Ncr1*, and *Itgae*) as well as T and B cells (n = 60), with most being CD8 T cells (marked by *Cd8a* and *Cd8b1*) and a very small fraction being CD4 T cells (marked by *Cd4*) and B cells (marked by *Cd79a* and *Cd79b*). These immune cells represented mammary-tissue-specific cells because we discarded the lymph nodes in sample preparation. The only major stromal population not captured in our analysis was adipocytes, which were removed with the supernatant during tissue dissociation.

We generated gene signatures characterizing each cell type by multiple pairwise differential gene expression analyses (see Method Details). The resulting cell-type-specific signatures (Figure 1G; Table S1) can serve as a useful resource for identifying or isolating cell types within heterogeneous mammary populations. Importantly, the ability to capture relatively rare populations, such as lymphatic endothelial cells and various immune cells, as well as the ability to detect lesser-known populations, such as HS-AV cells and distinctive macrophages, highlights the power of scRNA-seq to identify diverse cell types in the mammary gland without *a priori* knowledge required to pre-isolate these populations.

### Prevalent Alterations in Cell Proportions and Gene Expression in Aged Mammary Glands

**Figure 2.**
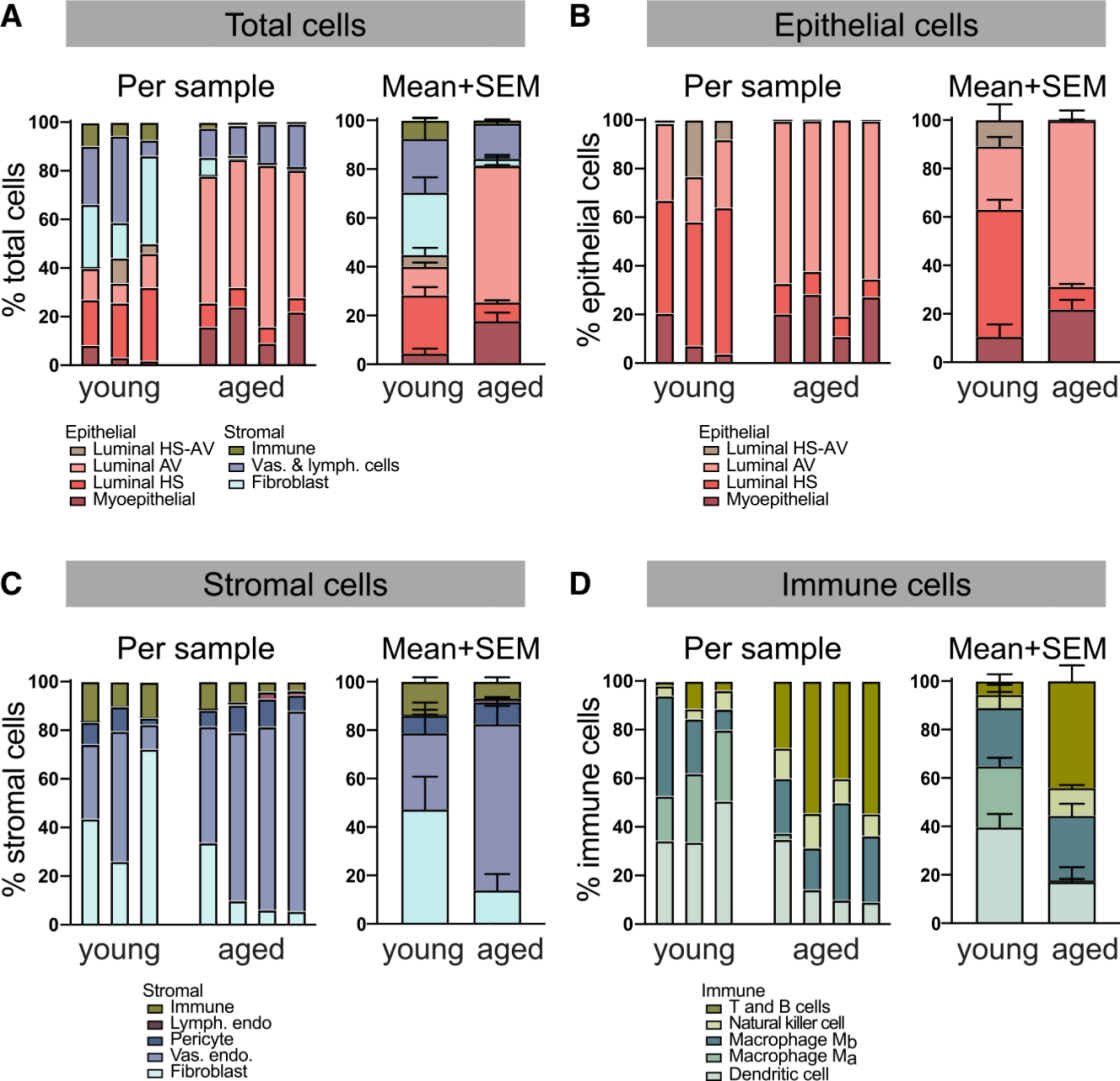
Prevalent Alterations in Cell Proportions in Aged Mammary Glands Relative proportions of specified cell types among total (A), epithelial (B), stromal (C), and immune (D) cells.

ScRNA-seq analysis revealed differences between young and aged mammary glands in both cell type composition and gene expression. Cell type composition inferred from the transcriptomic data is consistent among biological replicates despite sample-to-sample variation and differs significantly across age groups (Figures 2A–2D, S1A, and S2A; Table S2). The relative proportion of epithelial cells to stromal cells increased considerably with age: epithelial cells increased from 45% of total cells to 82%, whereas stromal cells decreased commensurately from 55% to 18% (Figure 2A; p < 0.0001, Fisher’s exact test). Within the epithelial and stromal compartments, cell type composition also changed with age. Among epithelial cells (Figure 2B), AV luminal cells expanded 3-fold (p < 0.0001, Fisher’s exact test), whereas HS luminal cells diminished 6-fold (p < 0.0001, Fisher’s exact test). Among stromal cells (Figure 2C), the relative abundance of fibroblasts fell 3-fold with aged cells (p < 0.0001, Fisher’s exact test), whereas vascular endothelial cells rose 2-fold (p < 0.0001, Fisher’s exact test). Furthermore, although the proportion of total immune cells within the stromal compartment remained relatively unaltered, the composition of immune cell types changed with age (Figure 2D). The proportion of dendritic cells decreased 2-fold (p < 0.0004, Fisher’s exact test). M_a_ macrophages decreased 40-fold (p < 0.0001, Fisher’s exact test), whereas M_b_ macrophages remained relatively constant, thus rendering M_b_ the dominant macrophage population in aged tissues. In contrast to the reduction in myeloid cells, the proportion of lymphocytes increased with age. Specifically, NK cell percentage increased 2-fold (p < 0.03, Fisher’s exact test), whereas CD8 T cells increased 8-fold (p < 0.0001, Fisher’s exact test). Gene signature-based cell-cycle inference analysis of epithelial populations revealed varying degrees of proliferation, which is consistent with the high proliferation rate expected at diestrus from progesterone stimulation (Figure S2B) (Fata et al., 2001; Joshi et al., 2010). The proportion of proliferating epithelial cells decreased with age, in accordance with previous observations (Raafat et al., 2012); the decrease was only moderate, again, likely due to the effect of diestrus. Notably, the higher proliferation of certain cell types (myoepithelial and AV cells) compared with others (HS and HS-AV) is concordant with previous studies (Clarke et al., 1997; Giraddi et al., 2015) and may partially explain the accumulation of myoepithelial and AV cells relative to HS and HS-AV cells at older age (Figure 2B). Besides proliferation, other factors, such as cell death, migration, and differentiation, may also account for the age-dependent changes in cell type proportions.

In addition to composition, cell type-specific gene expression differences were also detected in young and aged mammary glands (Figures S3A–S3L). Within the epithelial compartment, myoepithelial cells exhibited the largest number of differentially expressed genes (Figure S3A–S3D), whereas within the stromal compartment, vascular endothelial cells showed the most striking pattern (Figure S3E–S3H). Moreover, M_b_ macrophage gene expression was affected to a greater extent compared with other immune cells (Figure S3I–S3L). These age-dependent changes are examined in greater detail below. Analysis of the number of genes detected per cell indicated that these age-associated gene expression patterns were not simply driven by sample variation in total genes captured by scRNA-seq (Figure S3M). Overall, our results demonstrate that both cellular proportion and gene expression are profoundly affected by aging.

### Aged Myoepithelial Cells Show Altered Gene Expression

**Figure 3.**
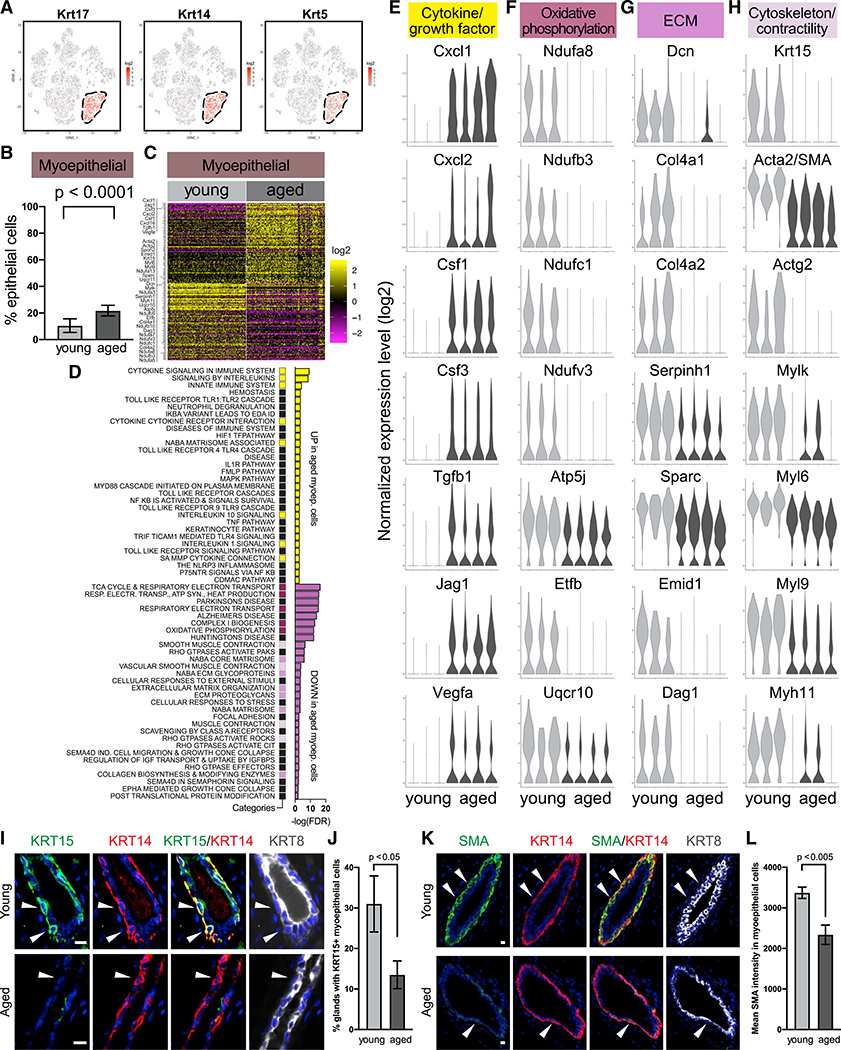
Aged Myoepithelial Cells Show Altered Gene Expression (A) Myoepithelial cells are identified by basal keratins in scRNA-seq data. (B) Relative proportion of myoepithelial cells in young (n = 3) and aged (n = 4) mice by scRNA-seq (Fisher’s exact test). (C) Differentially expressed genes in young versus aged myoepithelial cells. See also Table S3. (D) Top gene sets identified by pathway analysis of differentially expressed genes in (C), with recurring gene sets highlighted. See also Table S4. (E–H) Violin plots showing expression of select genes from (D). (I–L) Representative immunofluorescence staining of KRT15 (I) and SMA (K) in myoepithelial cells (stained as KRT14^+^ and KRT8^−^, indicated by arrows). Scale bar, 10 μm. Quantifications are shown in (J) and (L), respectively (n = 6 mice per age group, Student’s t test).

Myoepithelial cells, distinguished by basal keratins *Krt17*, *Krt14*, and *Krt5* (Figure 3A), only increased slightly in abundance with age (Figure 3B); however, they exhibited a striking degree of gene expression changes (Figure 3C; Table S3). Differential expression analysis detected 111 genes upregulated and 106 genes downregulated in aged myoepithelial cells. Pathway analysis indicated that those genes primarily fell into four functional categories—cytokines/growth factors, oxidative phosphorylation, extracellular matrix (ECM), and cytoskeleton/contractility genes (Figures 3D–3H; Table S4). More specifically, aged myoepithelial cells increased expression of multiple cytokines that are known to regulate the immune system (including *Cxcl1*, *Cxcl2*, *Cxcl16*, *Csf1*, and *Csf3*), as well as several growth factors (*Tgfb1*, *Jag1*, and *Vegfa*) (Figure 3E; Table S3). Aged myoepithelial cells also showed lower expression of oxidative phosphorylation genes (*Ndufa3*, *a5*, *a7*, *a8*, *a13*, *b3*, *b9*, *b10, c1*, *v3*, *Atp5j*, *Etfb*, *Uqcr10*, and *Uqcr11*) (Figure 3F; Table S3), which may reflect reduced metabolic activity. Expression of several ECM-related genes decreased with age (*Dcn*, *Col4a1*, *Col4a2*, *Serpinh1*, *Sparc*, *Emid1*, *Dag*, and *Spon2*) (Figure 3G; Table S3), indicating a potential impairment in the function to synthesize basement membrane. Expression is also reduced in multiple actomyosin-related genes (*Acta2*, *Actg2*, *Mylk*, *Myl6*, *Myl9*, and *Myh11*) as well as the cytokeratin intermediate filament *Krt15* (Figure 3H; Table S3), indicating a potential decline in the ability to maintain contractility.

Of note, because *Krt15* and *Acta2* (also known as smooth muscle actin [SMA]) have been reported as myoepithelial markers in murine mammary glands (Bach et al., 2017; Pal et al., 2017), their reduced expression with age would suggest that they may not be reliable markers for older myoepithelial cells. We, therefore, performed immunofluorescence staining to confirm their protein expression pattern in young and aged mammary glands, using KRT14 as a reference myoepithelial marker because its expression remained stable as detected by scRNA-seq. Consistent with scRNA-seq data, immunostaining demonstrated that KRT15 and ACTA2/SMA were expressed in young myoepithelial cells, but their level significantly decreased in aged glands (Figures 3I–3L). Therefore, our findings suggest that *Krt15* and *Acta2*, as well as other cytoskeletal or contractility genes (e.g., *Mylk*, *Myl6*, *Myl9*, and *Myh11*), should be used as myoepithelial markers with caution depending on age.

### Alterations in Luminal HS and AV Cells with Age

**Figure 4.**
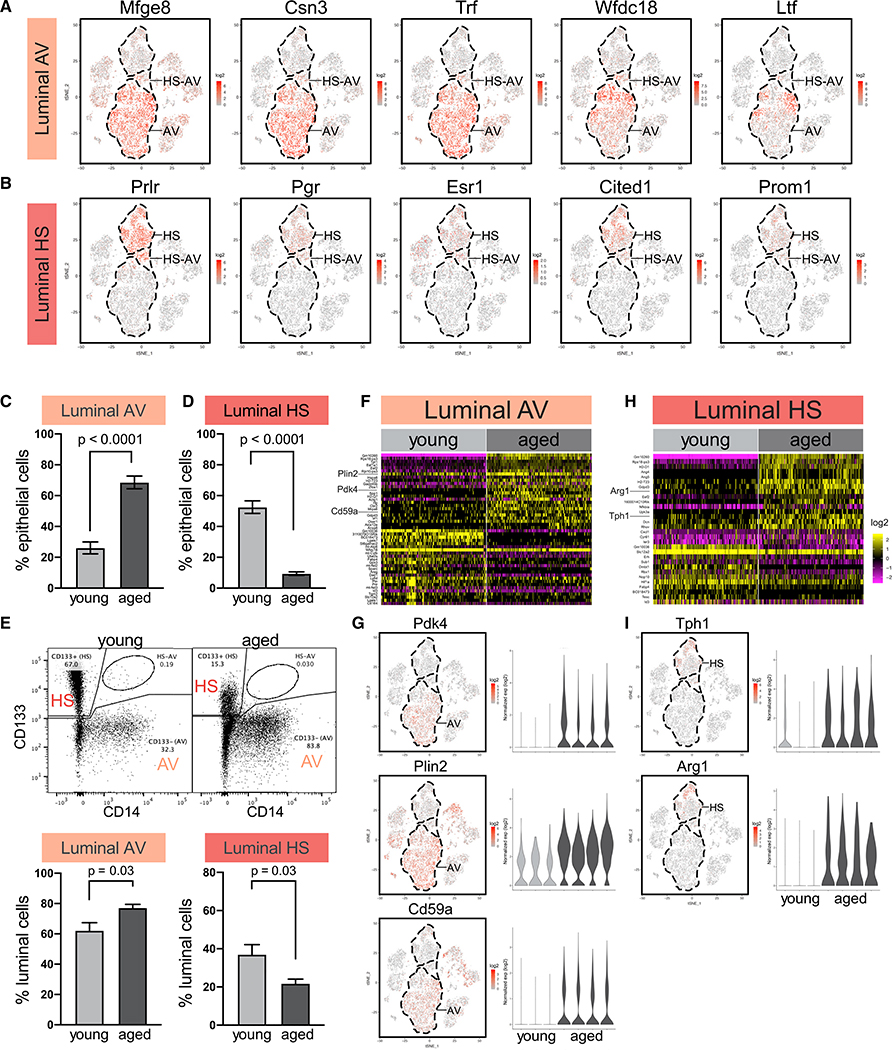
Alterations in Luminal Hormone-Sensing (HS) Cells and Alveolar (AV) Cells with Age (A) AV cells are distinguished by milk-related genes in scRNA-seq data. (B) HS cells are distinguished by hormone receptors and canonical HS lineage markers. (C and D) Relative proportion of AV (C) and HS (D) cells in young (n = 3) and aged (n = 4) mice by scRNA-seq (Fisher’s exact test). (E) FACS analysis of relative abundance of AV cells and HS cells in young (n = 8) and aged (n = 6) mice. In the representative FACS plots, 10,000 cells are displayed. Statistical significance is determined by Student’s t test. (F and G) Differentially expressed genes in young versus aged AV cells presented as heatmap (F) and specific examples (G). See also Table S3. (H and I) Differentially expressed genes in young versus aged HS cells presented as heatmap (H) and specific examples (I). See also Table S3.

We next examined age-related changes in luminal HS cells and AV cells. AV cells were identified by their characteristic milk-related genes *Mfge8*, *Csn3*, *Trf*, *Wfdc18*, and *Ltf* (Figure 4A), whereas HS cells were identified by hormone-sensing markers *Prlr*, *Pgr*, and *Esr1* and canonical HS lineage markers *Cited1* and *Prom1* (Figure 4B). The proportion of AV cells expanded with age from 26% to 69% within the epithelial compartment, and HS cells diminished from 53% to 9% (Figures 4C and 4D). Fluorescence-activated cell sorting (FACS) analysis of an independent cohort of mice confirmed the age-dependent increase of AV cells (Lin^−^, Epcam^+^, CD133^−^, CD14^+/−^; from 62% to 77% of the luminal compartment) and decrease of HS cells (Lin^−^, Epcam^+^, CD133^+^, CD14^−^; from 37% to 22% of the luminal compartment) (Figure 4E). The difference in the exact percentages detected by scRNA-seq and FACS is likely due to technical differences as well as post-transcriptional and/or translational regulations.

Differential expression analysis of young versus aged AV cells revealed 24 upregulated genes and 24 downregulated genes (Figure 4F; Table S3), including increased expression of several milk-biosynthesis regulators that are exclusively detected in AV cells but not HS cells. Those genes include pyruvate dehydrogenase kinase *Pdk4* (Kankel and Reinauer, 1976), milk lipid droplet-coating protein *Plin2* (Russell et al., 2011), and milk bioactive protein *Cd59a* (Bjørge et al., 1996) (Figure 4G). This expression pattern led us to examine other milk-related genes, many of which were also upregulated with age in AV cells in at least three of four replicates, including lactose synthase subunit *Lalba*; caseins *Csn1s1*, *Csn1s2a*, and *Csn2*; and milk osteopontin *Spp1* (Figure S4A). The increased expression of milk biosynthesis genes may potentially reflect a shift toward a more mature alveolar state. This is consistent with previous reports (Raafat et al., 2012) and our observation that aged mammary glands showed signs of activated alveolar cells independent of pregnancy, including increased secretory material in acini and dilated ducts (Figure S4B).

On the other hand, aged HS cells showed upregulation of 18 genes and downregulation of 12 genes (Figure 4H; Table S3). Many of those were regulated by hormone receptors, consistent with their expression in HS cells and potentially reflecting hormonal changes associated with aging (Ace and Okulicz, 1998; Gannon et al., 2010; Gundlah et al., 2005; Hickey et al., 2013; Ono et al., 2014; Sampath et al., 2002; Tsai et al., 2002; Tynan et al., 2005). Two upregulated genes, *Tph1* and *Arg1*, were particularly noteworthy because of their exclusive expression in HS cells and no other cell types (Figure 4I). Expression of *Tph1* (tryptophan hydroxylase 1) in the mammary gland is specifically activated during lactation for serotonin biosynthesis (Laporta et al., 2014; Matsuda et al., 2004). Its increased level in aged HS cells is consistent with the lactation-primed state of aged mammary glands discussed above. *Arg1* (arginase 1) inhibits proliferation and function of T cells and NK cells by depleting L-arginine in the microenvironment (Bronte and Zanovello, 2005; Gantt et al., 2014). *Arg1* is commonly expressed by immune inhibitory cells, such as tumor-associated macrophages and myeloid derived suppressor cells, as well as by a diversity of tumor cells, including breast cancer. We confirmed ARG1 protein expression in HS cells as well as its increase with age by performing tissue staining in an independent cohort of mice (Figure S4C). Increased *Arg1* expression in aged HS cells is consistent with an immunosuppressive microenvironment, as discussed later.

### Luminal HS-AV Cells Showed Age-Dependent Abundance

**Figure 5.**
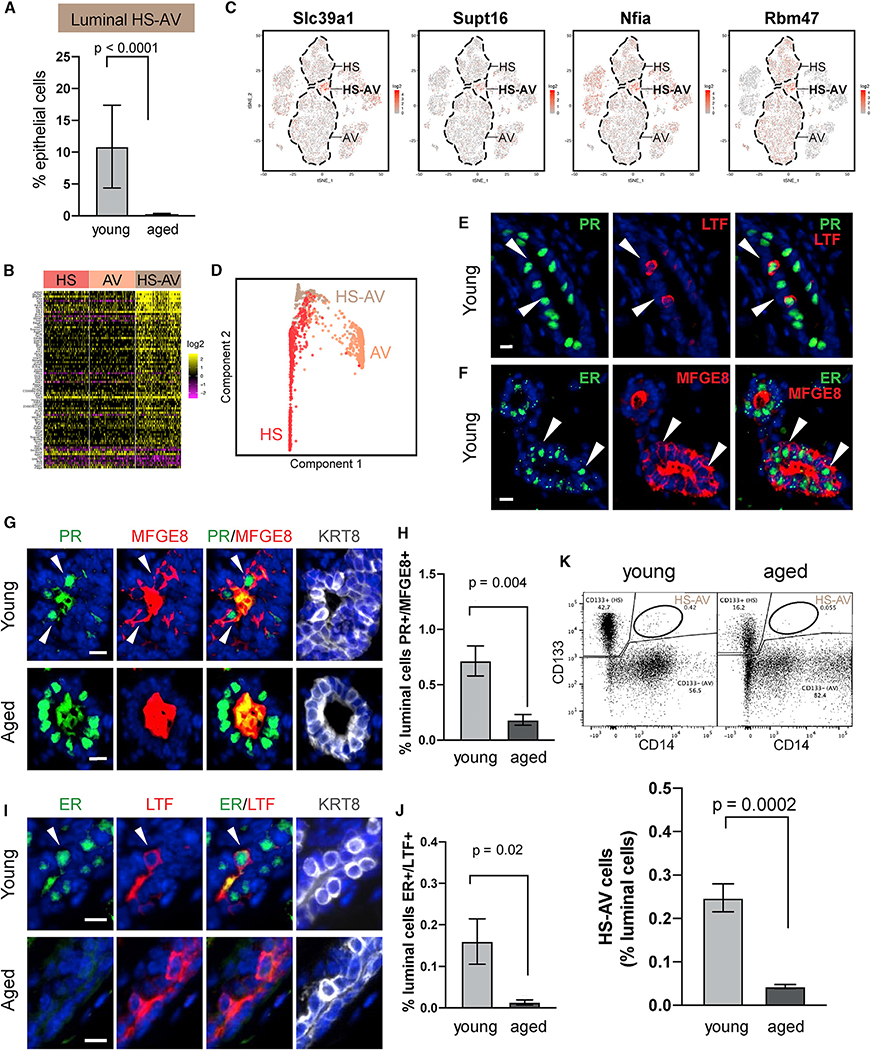
Luminal HS-AV Cells Show Age-Dependent Abundance (A) Relative proportion of HS-AV cells in young (n = 3) and aged (n = 4) mice by scRNA-seq (Fisher’s exact test). (B) Markers distinguishing HS-AV cells from both HS cells and AV cells in young mice. See also Table S5. (C) Expression pattern of select HS-AV markers in (B). (D) Dimensionality reduction plot from STREAM lineage trajectory analysis of luminal cells in young mice (n = 3). See also Figure S5D. (E and F) Representative immunofluorescence staining of HS-AV cells (indicated by arrows) co-expressing HS marker (PR or ER) and AV marker (LTF or MFGE8) in young mice. (G–J) Immunofluorescence staining of HS-AV cells (indicated by arrows) co-expressing PR and MFGE8 (G) or ER and LTF (I) in young and aged mice. Scale bar, 10 μm. Quantifications are shown in (H) and (J), respectively (n = 6 mice per age group, Student’s t test). In (G) and (I), MFGE8 and LTF are also detected as secreted proteins in the lumen. KRT8 marks luminal cells. (K) FACS quantification of HS-AV cells co-expressing HS marker CD133 and AV marker CD14 in an independent cohort of young (n = 8) and aged (n = 6) mice. In the representative FACS plots, 10,000 cells are displayed. Statistical significance is determined by Student’s t test.

Our scRNA-seq analysis also revealed a rare population of HS-AV cells that co-expressed HS and AV markers (Figures 1E, 4A, and 4B), accounting for an average of 11% of epithelial cells in young mammary glands (Figure 5A). Similar cells expressing HS and AV gene signatures have also been identified in young mice by Pal et al. (2017) and Bach et al. (2017) at comparable proportions and in ovariectomized mice by Kanaya et al. (2019) at higher abundance (Figures S5A and S5B), but they have not been further characterized. We performed differential gene expression analysis to identify markers distinguishing HS-AV cells from HS cells and AV cells, limiting the analysis to young tissues to focus on cell-type-specific difference independent of aging. The results revealed that HS-AV cells are distinguished by higher expression of 72 genes and lower expression of nine genes (Figures 5B and 5C; Table S5). Many of the upregulated markers fell into four functional categories, highlighting the potential implications for the biological roles of HS-AV cells (Figure S5C): (1) regulators of mammary development, lobulo-alveologenesis, and lactation, such as *Spry2*, *B4galt1*, *Slc39a1*, *Skil*, *Agpat1*, and *Neat1*, suggesting that HS-AV cells might play a role in mammary gland morphogenesis and milk production (Agarwal et al., 2017; Crisà et al., 2016; Jahchan et al., 2012; 2010; Littlejohn et al., 2010; Lv et al., 2015; McCormick et al., 2014; Sigurdsson et al., 2013; Standaert et al., 2014; Zhang et al., 2014, 2018b; Zhu and Nelson, 2013); (2) chromatin-modifying enzymes, including *Kmt2d/Mll2*, *Whsc1l1/Nsd3*, *Gatad1/Odag*, *Kdm6b/Jmjd3*, *Cbx3/Hp1*γ, and *Supt16/Fact140*, which have been implicated in promoting stem cell phenotypes and regulating differentiation (Burchfield et al., 2015; Denissov et al., 2014; Froimchuk et al., 2017; Lomniczi et al., 2015; Mas et al., 2018); (3) transcription factors, including *Nfia*, *Tmf1*, *Bhlhe41*, *Arglu1*, *Btg1*, and, in particular, *Sox9*, which is known to regulate luminal progenitor cell fate (Malhotra et al., 2014; Guo et al., 2012; Wang et al., 2017); and (4) mRNA processing and modification enzymes, namely *Srrm2*, *Wtap*, *Alkbh5*, *Rbm47*, *Hnrnpul1*, and *Pan3*, which regulate mRNA splicing, m^6^A methylation, mRNA processing, and degradation (Blencowe et al., 1998; Kim et al., 2019; Kzhyshkowska et al., 2003; Wahle and Winkler, 2013; Yue et al., 2018; Zheng et al., 2013). Overall, these gene signatures suggest a potential role for HS-AV cells in differentiation plasticity.

Next, we examined the lineage relationship of HS-AV cells to HS cells and to AV cells by performing STREAM (single-cell trajectories reconstruction, exploration, and mapping) lineage trajectory analysis (Chen et al., 2019) on scRNA-seq data of young mice. HS-AV cells primarily localized to the bifurcation junction between HS cells and AV cells (Figures 5D and S5D). This pattern was highly reproducible when the analysis was repeated within each of the three young samples (Figure S5D). The predicted lineage trajectory is consistent with the gene expression pattern of HS-AV cells being a hybrid state between the HS and AV lineages and suggests the possibility that HS-AV cells might have the potential to differentiate into HS cells and into AV cells.

Because HS-AV cells have not been reported beyond scRNA-seq studies, we further verified their existence using two orthogonal approaches. First, we performed *in situ* immunofluorescence staining in a panel of young mammary glands, using established hormone-sensing markers—progesterone receptor (PR) and estrogen receptor (ER)—and alveolar markers—lactotransferrin (LTF) and milk fat globule-EGF factor 8 (MFGE8). This analysis detected the presence of HS-AV cells co-expressing PR/ER and LTF/MFGE8, scattered within both ducts and acini (Figures 5E and 5F). Quantification showed that PR^+^/MFGE8^+^ cells and ER^+^/LTF^+^ cells represented 0.72% and 0.16% of luminal cells, respectively (Figures 5G–5J). The lower abundance of ER^+^/LTF^+^ cells compared with PR^+^/MFGE8^+^ cells by immunostaining is consistent with the lower abundance of LTF^+^ cells compared with MFGE8^+^ cells within the HS-AV population by scRNA-seq (Figure 4A). As further validation, we analyzed an independent set of young mice by FACS, using HS marker CD133 and AV marker CD14. Again, HS-AV cells can be detected as CD133^+^/CD14^+^ cells within the luminal population (Lin−, Epcam^hi^) in all young tissues examined, with an average abundance of 0.25% (Figure 5K). Similar CD133^+^/CD14^+^ HS-AV cells were also detected in murine mammary organoid cultures (Figure S5E). Importantly, we confirmed that these cells were not doublet artifacts formed by CD133^+^ cells and CD14^+^ cells because microscopy examination of FACS-isolated CD133^+^/CD14^+^ cells demonstrated that they were indeed singlets (Figure S5F). Of note, although HS-AV cells can be detected using multiple approaches, they were in lower abundance when analyzed at the protein level by immunofluorescence and FACS (Figures 5H, 5J, and 5K) than at the RNA level by scRNA-seq (Figure 5A). The limited number of protein markers used in immunofluorescence and FACS analyses likely only captured a subset of the HS-AV population identified transcriptomically. In addition, the difference may reflect gene expression regulation at the post-transcriptional and/or translational levels.

Interestingly, the abundance of HS-AV cells diminished dramatically with age. ScRNA-seq showed that HS-AV cells accounted for an average of 11% of epithelial cells in young mice but only 0.3% in aged mice (Figure 5A). We confirmed this age-dependent decrease of HS-AV cells by immunofluorescence staining of PR^+^/MFGE8^+^ cells and ER^+^/LTF^+^ cells in tissues (Figures 5G–5J). In addition, FACS analysis of an independent cohort also demonstrated that aged mice consistently harbored a lower proportion of HS-AV cells than young mice had (Figure 5K). Collectively, these results established an age-dependent existence of the HS-AV population.

### Age-Dependent Alterations in Stromal Fibroblasts and Vascular/Lymphatic Cells

**Figure 6.**
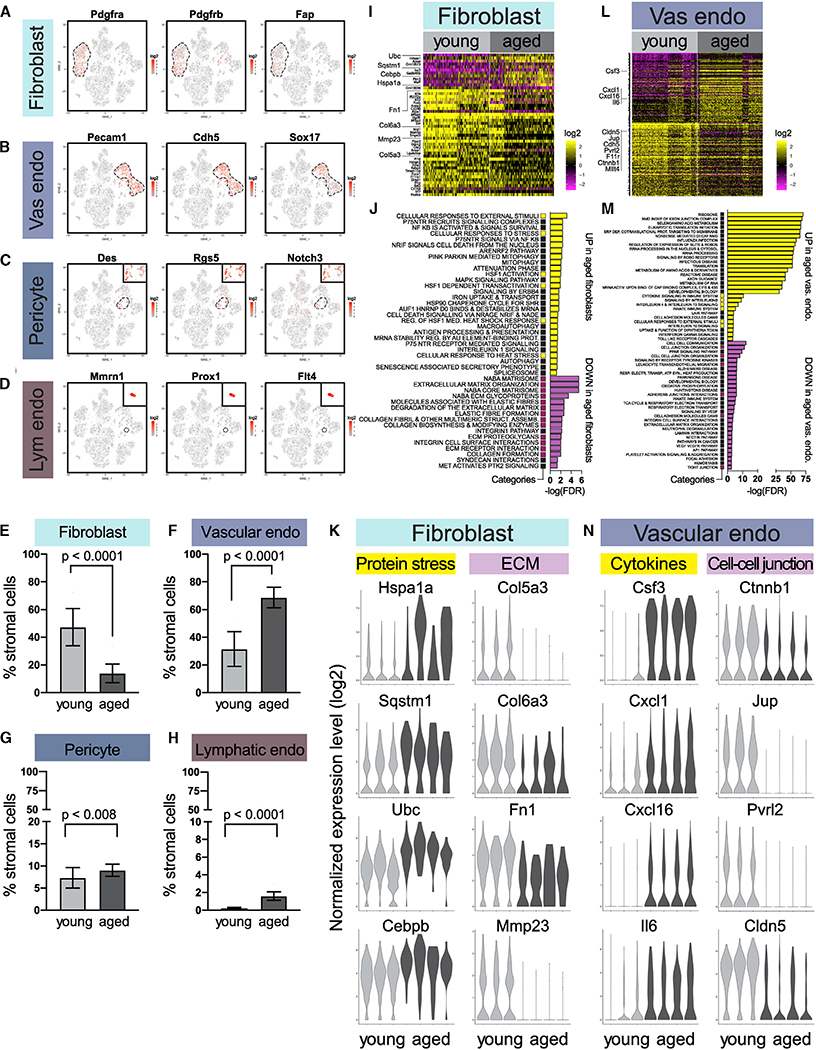
Age-Dependent Alterations in Stromal Fibroblasts and Vascular and Lymphatic Cells (A–D) Fibroblasts (A), vascular endothelial cells (B), pericytes (C), and lymphatic endothelial cells (D) are distinguished by characteristic makers in scRNA-seq data. (E–H) Relative proportions of fibroblasts (E), vascular endothelial cells (F), pericytes (G), and lymphatic endothelial cells (H) in young (n = 3) and aged (n = 4) mice by scRNA-seq (Fisher’s exact test). (I) Differentially expressed genes in young versus aged fibroblasts. See also Table S3. (J) Top gene sets identified by pathway analysis of differentially expressed genes in (I), with recurring gene sets highlighted. See also Table S4. (K) Violin plots of select genes in (I) and (J) for fibroblasts. (L) Differentially expressed genes in young versus aged vascular endothelial cells. See also Table S3. (M) Top gene sets identified by pathway analysis of differentially expressed genes in (L), with recurring gene sets highlighted. See also Table S4. (N) Violin plots of select genes in (L) and (M) for vascular endothelial cells.

In addition to mammary epithelial cells, we analyzed age-associated changes in stromal cells (Figures 6A–6D). The most abundant stromal cell type in young tissues was fibroblasts (Figures 6E–6H). These cells homogeneously expressed fibroblast markers *Pdgfra*, *Pdgfrb*, and *Fap* (Figure 6A). They also expressed high level of ECM genes (e.g., *Fn1*, *Col1a1*, *Col1a2*, and *Col3a1*) but not contractility genes (e.g., *Acta2*, *Myl9*, *Mylk*, and *Myh11*) (Figure S6A), indicating that they were ECM-producing fibroblasts instead of contractile myofibroblasts. The relative proportion of fibroblasts decreased with age (Figure 6E), a pattern corroborated by immunohistochemistry (IHC) staining using the fibroblast marker PDGFRα (Figure S6B). In addition, scRNA-seq differential expression analysis revealed 11 genes upregulated and 37 genes downregulated with age (Figure 6I; Table S3), including increased expression of stress-related genes (*Hspa1a*, *Sqstm1*, *Ubc*, *Cebpb*, and *Gadd45b*) and decreased expression of ECM-related genes (*Col5a3*, *Col6a3*, *Fn1*, and *Mmp23*) (Figures 6J and 6K; Table S4). Notably, fibronectin, encoded by *Fn1*, is a major ECM component and a master organizer of the matrix assembly (Halper and Kjaer, 2014; Pankov and Yamada, 2002; Theocharis et al., 2016). The decrease in both fibroblast proportion and ECM gene expression is consistent with previous reports in human mammary glands that aging is associated with a reduction of connective tissues (Gertig et al., 1999; Hutson et al., 1985).

Other stromal populations captured by scRNA-seq included vascular and lymphatic cells, namely vascular endothelial cells (expressing *Pecam1*, *Chd5*, and *Sox17*), pericytes (expressing *Des*, *Rgs5*, and *Notch3*), and lymphatic endothelial cells (expressing *Mmrn1*, *Prox1*, and *Flt4*) (Figures 6B–6D). Among those populations, vascular endothelial cells exhibited the most dramatic changes with age, increasing in relative abundance (Figure 6F) and expressing 159 upregulated and 169 downregulated genes (Figure 6L; Table S3). Pathway analysis revealed that many upregulated genes were associated with cytokines that are known to affect the immune microenvironment (*Csf3, Cxcl1*, *Cxcl16*, and *Il6*), whereas many downregulated genes were associated with cell-cell junctions (including adherens junction genes *Ctnnb1*, *Jup*, *Pvrl2*, *Cdh5*, and *Mllt4* and tight junction genes *Cldn5* and *F11r*) (Figures 6M and 6N; Table S4). These changes may reflect an altered endothelial-immune interaction and vascular permeability. In comparison, pericytes and lymphatic endothelial cells remained relatively constant with age in terms of proportion (Figures 6G and 6H; Table S2) and gene expression (Figures S3G and S3H).

### Age-Dependent Alterations in Myeloid and Lymphoid Immune Cells

**Figure 7.**
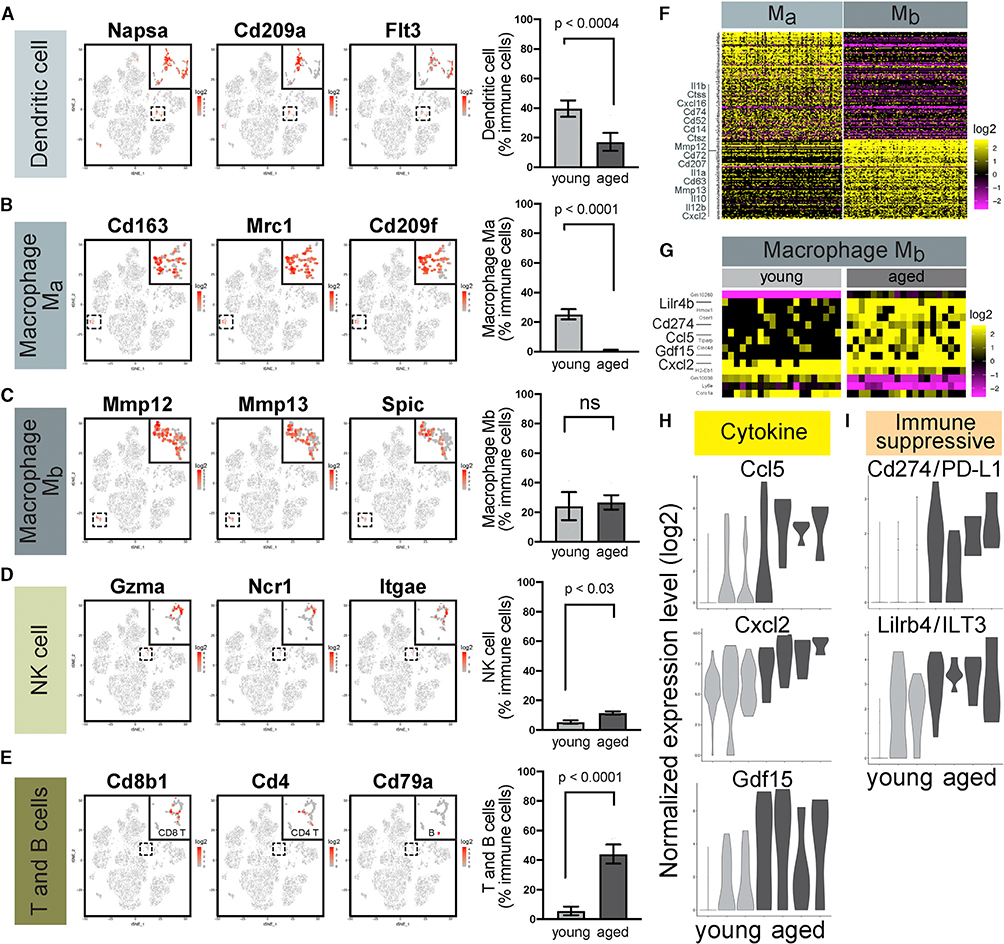
Age-Dependent Alterations in Myeloid and Lymphoid Immune Cells (A–E) M_a_ and M_b_ macrophages, dendritic cells, natural killer (NK) cells, and T and B cells, are distinguished by their respective markers in scRNA-seq data (left). Their relative abundance in young (n = 3) and aged (n = 4) mice are shown on the right (Fisher’s exact test). (F) Genes distinguishing M_a_ and M_b_ macrophages. See also Table S6. (G) Differentially expressed genes in young versus aged M_b_ macrophages. See also Table S3. (H and I) Violin plots of cytokines (H) and immunosuppressive ligands (I) upregulated in aged M_b_ macrophages compared to young cells.

ScRNA-seq also detected remarkable changes in the composition of immune cell types in aged mammary glands. As mentioned above, the proportion of myeloid cells diminished, whereas that of lymphoid cells expanded with age. Among myeloid cells, dendritic cells (expressing *Napsa*, *Cd209a*, and *Flt3*) and M_a_ macrophages (expressing *Cd163, Mrc1*, and *Cd209f*) decreased in relative abundance, leaving M_b_ macrophages (marked by *Mmp12, Mmp13*, and *Spic*) the major myeloid population in older mammary glands (Figures 7A–7C). Among lymphoid cells, the proportion of both NK cells (expressing *Gzma*, *Ncr1*, and *Itgae*) and CD8 T cells (expressing *Cd8a* and *Cd8b1*) increased with age (Figures 7D and 7E). Because M_a_ macrophages and CD8 T cells represented the most striking proportional changes, we also analyzed their relative abundance by IHC staining. CD163^+^ M_a_ macrophages, which are localized preferentially in stroma, decreased in abundance with age (Figure S7A). In contrast, CD8^+^ T cells, which were closely associated with the mammary epithelium, increased in abundance with age (Figure S7B). Compared with scRNA-seq, IHC is considerably more sensitive in detecting these rare cell types.

Macrophages have critical roles in mammary gland development and breast cancer (Brady et al., 2016; Gouon-Evans et al., 2002; Guerriero, 2018); however, the heterogeneity of resident macrophages within healthy mammary glands remains incompletely understood. To further characterize the two macrophage populations captured by scRNA-seq, we performed a differential gene expression analysis and obtained 241 markers that distinguished M_a_ and M_b_ macrophages (Figure 7F; Table S6). The results suggest that M_a_ and M_b_ macrophages do not show a clear association with classical M1/M2 polarization. Instead, comparison of these markers to a recent study on mammary macrophages (Jäppinen et al., 2019) revealed that M_a_ macrophages are enriched for key markers of fetal-derived macrophages, *Mrc1* (also known as *Cd206*) and *Adgre1* (also known as *F4/80*), whereas M_b_ macrophages are enriched for adult-derived macrophage markers, MHC II genes *H2-Aa*, *H2-Ab1*, *H2-DMa*, *H2-DMb1*, *H2-Eb1*, and *H2-Oa* (Figures 7F and S7C; Table S6). Therefore, M_a_ cells may represent fetal-derived macrophages arising from fetal yolk sac and liver, whereas M_b_ cells may represent adult-derived macrophages arising from the bone marrow. In addition, two recent studies proposed that mammary macrophages can be defined by their physical locations. Stromal macrophages (characterized as Mrc1/Cd206^+^, Lyve1^hi^, Itgax/Cd11c^−^, and Vcam1^lo^, or as Mrc1/Cd206^hi^, Lyve1^hi^, Cd209f^hi^, and Cd209g^hi^) survey the ECM and largely arise from fetal origin, whereas ductal macrophages (characterized as Mrc1/Cd206^−^, Lyve1^lo^, Itgax/Cd11c^+^, Vcam1^hi^, or as Mrc1/Cd206^lo^, Lyve1^lo^, Cd74^hi^, H2-Ab1^hi^) survey the epithelia and largely arise from adult bone marrow after puberty (Dawson et al., 2020; Wang et al., 2020). Notably, these gene signatures are very closely recapitulated by M_a_ and M_b_ macrophages, respectively, suggesting that M_a_ cells may represent stromal macrophages while M_b_ cells may be ductal macrophages (Figures S7D and S7E; Table S6). In our analysis, M_b_ macrophages are also distinguishable from M_a_ macrophages by higher expression of cell-surface antigens *Cd14*, *Cd52*, *Cd63*, *Cd72*, *Cd74*, and *Cd207*; cytokines *Il1a*, *Il1b*, *Il10*, *Il12b*, *Cxcl2*, and *Cxcl16*; and proteases *Mmp12*, *Mmp13*, *Ctss*, and *Ctsz* (Figure 7F). Their increased relative proportion with age is consistent with a replacement of fetal-derived macrophages by adult bone-marrow-derived macrophages as the mammary gland ages (Ginhoux and Guilliams, 2016; Hoeffel and Ginhoux, 2018). The recruitment of M_b_ macrophages may be mediated by the increased expression of *Csf1* and *Csf3* in aged myoepithelial cells and vascular endothelial cells (Figures 3E and 6N).

Aging induced a very modest number of gene expression changes in M_b_ macrophages compared with epithelial and non-immune stromal cell types, albeit more than other immune cells (Figure S3J), with 10 genes upregulated and four genes downregulated with age in a consistent manner across all samples (Figure 7G; Table S3). Although the number is very modest, it contains a few intriguing genes, including cytokines *Ccl5*, *Cxcl2*, and *Gdf15* (Figure 7H). CCL5 has been shown to recruit T cells (Arango Duque and Descoteaux, 2014); thus, its increased expression in aged M_b_ macrophages is consistent with the higher abundance of T cells observed in aged mammary glands. Furthermore, expression of two immunosuppressive ligands targeting T cells and NK cells also increased—*Cd274* (also known as *Pd-l1*) (Hsu et al., 2018; Lu et al., 2019; Yamazaki et al., 2002) and *Lilrb4* (also known as *Ilt3*) (Cella et al., 1997; Deng et al., 2018; Suciu-Foca and Cortesini, 2007) (Figure 7I)—thereby potentially promoting an immunosuppressive microenvironment. Taken together, these results highlight the diverse and dynamic immune landscape in aging mammary tissues.

## DISCUSSION

To better understand the effects of aging on mammary glands, we have created a single-cell transcriptomic atlas to capture murine mammary epithelial and stromal changes associated with aging, corresponding to the transition from human early adulthood to perimenopause. Considering the longer human lifespan, there may be additional age-related changes not captured by a murine model. However, unlike human studies, mouse analyses can directly assess aging-specific alterations without confounding factors such as pregnancy history, hormone cycle, genetic background, diet, and carcinogen exposure. Importantly, the cell- and gene-level profiles identified herein are consistent across multiple biological replicates, suggesting that the intrinsic effects of aging may follow a defined developmental program instead of a stochastic degeneration.

Our scRNA-seq data indicate a potential shift of the luminal populations toward a more lactation-primed state in aging. Cell-type proportion analysis suggests a preferential expansion of AV cells relative to HS cells with age. Additionally, aged AV cells acquire higher expression of genes that are associated with milk production and are known to be upregulated in gestation (Bach et al., 2017). These changes are consistent with previous reports (Raafat et al., 2012) and our histological observation that aged nulliparous mice exhibit lobuloalveolar maturation, likely resulting from accumulated exposure to hormonal stimulation through multiple estrous cycles. Similar observation of age-dependent expansion of lobuloalveolar epithelium was reported in humans (Garbe et al., 2012; Hutson et al., 1985; Pelissier Vatter et al., 2018).

Other age-associated alterations detected by scRNA-seq may reflect a decline or dysregulation in cellular functions. For instance, aged myoepithelial cells downregulate genes known to mediate contractility and synthesize basement membrane (Haaksma et al., 2011; Ormerod et al., 1983; Sopel, 2010; Warburton et al., 1981; 1982), in part, consistent with previous reports that thinner and discontinuous basement membrane is associated with aging (Frantz et al., 2010; LaBarge et al., 2016). Similarly, aged fibroblasts decrease in relative abundance and ECM gene expression, suggesting an impaired ability to maintain a stromal matrix, thus consistent with previous reports of reduced connective tissue and mammographic density in older women (Gertig et al., 1999; Hart et al., 1989; Hutson et al., 1985; Li et al., 2005; McCormack et al., 2010; Unsworth et al., 2014). Additionally, aged endothelial cells downregulate expression of cell-cell junction components, potentially contributing to a compromised endothelial barrier and dysregulated vascular permeability (Bazzoni and Dejana, 2004). Vascular hyperpermeability is strongly associated with aging and is partly attributed to inflammatory cytokines, such as *Il6* and *Vegfa* (Donato et al., 2015; Oakley and Tharakan, 2014), which we observe to be upregulated in aged vascular endothelial cells and myoepithelial cells, respectively. Finally, aged mammary glands exhibit signs of pro-inflammatory microenvironment, with increased production of inflammatory cytokines (including *Cxcl1*, *Cxcl2*, *Cxcl16*, *Csfs1*, *Csf3*, *Ccl5*, and *Il6*) by aged myoepithelial cells, vascular endothelial cells, and macrophages and expanded proportions of bone-marrow-derived macrophages, NK cells, and T cells. These observations also suggest potential inter-cell-type crosstalk and recruitment, leading to systemic changes in aged mammary glands.

Given that aging is significantly correlated with breast cancer frequency in mice and human (Jenkins et al., 2014; LaBarge et al., 2016; Raafat et al., 2012), our findings may also provide initial insights into its underlying mechanisms. First, the expansion of AV luminal progenitor cells may pose a greater cancer risk because they have been proposed to be the cells of origin for triple-negative breast cancer (Lim et al., 2009; Molyneux et al., 2010; Proia et al., 2011). Second, aged myoepithelial cells display increased expression of *Jag1* and *Tgfβ*, which can stimulate tumor proliferation and invasion (Cohen et al., 2010; Hu et al., 2008; Muraoka et al., 2003; Muraoka-Cook et al., 2004). Third, the downregulation of genes associated with contractility and basement membrane may impair the ability of myoepithelial cells to restrain malignant luminal cell dissemination (Adriance et al., 2005; Pandey et al., 2010; Sirka et al., 2018). Previous studies have demonstrated that reduced myoepithelial cell contractility promotes malignant cell invasion (Sirka et al., 2018), whereas compromised basement membrane integrity is associated with breast cancer progression (Gusterson et al., 1982; Nakano et al., 1999; Natali et al., 1984; Tsubura et al., 1988). Loss of basement membrane has also been shown to promote abnormal luminal cell polarity, a hallmark of cancer (Gudjonsson et al., 2002). Moreover, increased vascular endothelial permeability, as suggested by reduced expression of cell-cell junction components, may further potentiate malignant cell dissemination (Maishi and Hida, 2017).

Additionally, aged mammary glands exhibit numerous signs of an inflammatory microenvironment that may promote the proliferation and metastasis of malignant cells (Greten and Grivennikov, 2019; Landskron et al., 2014; Zhang et al., 2018a). First, the expression of pro-inflammatory cytokines increased in aged myoepithelial cells, vascular endothelial cells, and macrophages. Second, the change in proportions of M_a_ and M_b_ macrophages suggests a replacement of fetal yolk sac and liver-derived macrophages by adult bone-marrow-derived cells with age. Bone-marrow-derived macrophages in murine mammary tumors have been associated with pro-tumorigenic properties by their ability to inhibit CD8 T cells (Franklin et al., 2014). In line with this, three key markers of bone-marrow-derived, tumor-associated macrophages described by Franklin et al. (2014), *Vcam1*, *Itgb5*, and *Itgax*, are also more highly expressed in M_b_ macrophages, suggesting potential similarities between M_b_ and adult-derived tumor-associated macrophages. Third, aged M_b_ macrophages upregulate expression of immunosuppressive ligands *Cd274/Pd-l1* and *Lilrb4/Ilt3*, thus, potentially inhibiting NK cells and T cells, creating a pro-tumor immune microenvironment (Lu et al., 2019). Increased inflammation with age was also previously observed in mouse mammary glands and other organs, but the specific source and mediators of inflammation remains incompletely understood (Dong et al., 2016; Sanada et al., 2018). Future studies may focus on the inflammatory cytokines and immune cell types enriched in aged mammary glands as detected herein to further delineate their exact contribution to tumor formation and progression. Of note, basal-like luminal cells have been reported in aged mice with mammary hyperplasia and in older women (Dong et al., 2016; Garbe et al., 2012; Pelissier Vatter et al., 2018), but are undetectable in our healthy aged mice, suggesting that these cells might represent a more-aberrant population that is usually absent in normal murine mammary glands.

In addition to age-related alterations, the identification of HS-AV cells is of considerable interest. These cells were also captured in two initial scRNA-seq studies of young murine mammary glands (Bach et al., 2017; Pal et al., 2017). Although Bach et al. (2017) did not comment on the hybrid nature of their gene expression, Pal et al. (2017) described them as luminal intermediates between the AV and HS lineages. Here, we verify the existence of HS-AV cells by tissue staining and FACS and define a gene signature distinguishing this population from other luminal cells. Furthermore, we find that HS-AV cells decrease in abundance with age, a potential consequence of the lack of self-renewal, differentiation into HS or AV lineage, or out-competition by HS or AV proliferation.

The lineage relationship of HS-AV cells to HS cells and AV cells remains to be determined. Lineage trajectory analysis suggests that HS-AV cells may be more progenitor-like than HS cells and AV cells. However, lineage tracing studies have demonstrated that postnatal HS and AV lineages are largely maintained independently by unipotent progenitors (Lilja et al., 2018; Van Keymeulen et al., 2017; Wang et al., 2017); thus, it is unlikely that, under normal homeostasis, HS-AV cells could function as bi-potent progenitors contributing to the HS and AV lineages. In contrast, it is more likely that HS-AV cells represent a distinct and restricted population, independent of HS cells and AV cells. Furthermore, depending on the rate of their cell division, it is possible that these rare HS-AV cells may not form clones of sufficient size to be detectable in sparse labeling and short-term lineage-tracing analyses.

If HS-AV cells are a stable population, then it raises the question of whether they serve a specific function in mammary glands. One possibility is that they might serve as dormant, resident, unipotent or bipotent luminal progenitors that can be activated during tissue regeneration. Similar examples of dormant progenitors have been described in other organs, such as lungs (Desai et al., 2014; Kim et al., 2005; Vaughan et al., 2015) and liver (Huch et al., 2013; Miyajima et al., 2014). Several observations provide a hint in support of that hypothesis. First, in scRNA-seq lineage trajectory analysis, HS-AV cells are localized to the bifurcation of the HS and AV branches, suggesting the possibility that they might be transcriptionally primed to give rise to cells in either lineage. Second, when compared with HS cells and AV cells, HS-AV cells are enriched for the expression of *Sox9*, a transcription factor reported to confer progenitor property (Guo et al., 2012). HS-AV cells also express other progenitor markers, such as *Kit*, *Aldh1a3*, and *Cd14*. Third, the increased expression of chromatin remodeling proteins in HS-AV cells compared with HS cells and AV cells might suggest a higher potential of differentiation plasticity, given the critical role chromatin modification has in regulating differentiation (Holliday et al., 2018; Wahl and Spike, 2017). Fourth, the diminished abundance of HS-AV cells with age is consistent with age-dependent depletion of stem/progenitor cells in certain tissue types (Encinas et al., 2011; Nishimura et al., 2005; Schultz and Sinclair, 2016). Finally, it remains possible that HS-AV cells represent a transient intermediate between the HS and AV populations, for instance, during differentiation of HS cells from AV cells, as proposed by Pal et al. (2017). However, evidence for such a transition has been limited, and further studies will shed light on that model. Overall, the identification of HS-AV cells suggests a greater complexity in mammary differentiation than was previously appreciated. Future lineage tracing and functional studies will be required to determine the roles of HS-AV cells in normal development and tissue regeneration and to elucidate their relationship with HS and AV lineages.

Taken together, these single-cell transcriptomic profiles reveal the dynamic heterogeneity in mammary tissues at young and old age. They provide a resource for future studies to understand the interactions between epithelial and stromal cells in aging and cancer.

## STAR★METHODS

### RESOURCE AVAILABILITY

#### Lead Contact

Further information and requests for resources and reagents should be directed to the Lead Contact, Joan Brugge (joan_brugge@hms.harvard.edu).

#### Materials Availability

This study did not generate new unique reagents.

#### Data and Code Availability

The accession number for the scRNA-seq data reported in this paper is GEO: GSE150580.

The R scripts used for scRNA-seq analysis are included in the supplemental information (Data S1).

### EXPERIMENTAL MODEL AND SUBJECT DETAILS

For single-cell RNA sequencing, young (3–4 months old) and aged (13–14 months old) virgin/nulliparous female mice of a mixed FVB, 129, C57BL/6J background were used in this study. For immunostaining, additional young (3–4 months old) and aged (16-months old) mice were used. For FACS analysis, an independent cohort of young (3–4 months old) and aged (12–14 months old) mice were used. All mice were bred and maintained in house under regular conditions, with 12hr/12hr light/dark cycle, and food (LabDiet 5053) and water *ad libitum*. Daily vaginal cytology was performed to determine the stage of the estrous cycle, and only actively cycling mice in diestrus were analyzed to avoid confounding effects from hormonal changes. Aged mammary glands were confirmed to have normal histology by a rodent pathologist. All animal work was performed in accordance to protocols approved by the Institutional Animal Care and Use Committee at Harvard University.

### METHOD DETAILS

#### Tissue dissociation for single-cell RNA-seq

Abdominal #4 mammary glands from young (n = 3) and aged (n = 4) mice, with lymph nodes removed, were finely minced and then incubated in a digestion solution containing DMEM/F12 (GIBCO 11330), 10% heat-inactivated fetal bovine serum, 2 mg/ml collagenase XI (Sigma C9407), and 0.1 mg/ml hyaluronidase (Sigma H3506) for 1.5 hours at 37°C with constant shaking at 150 rpm. The dissociated cells were then subjected to red blood cell lysis (Biolegend 420301), a 5-minute treatment with 1 U/ml dispase (Stem Cell Technologies 07913) and 0.1 mg/ml DNase (Stem Cell Technologies 07470), a 5-minute treatment with TrypLE (GIBCO 12605010), and filtered through a 40 μm cell strainer. Cells were resuspended in PBS containing 0.04% BSA, counted manually under the microscope, and adjusted for loading 7,000 viable cells for single-cell RNA-sequencing.

#### Single-cell RNA library preparation and sequencing

Single cell capturing and cDNA library generation were performed using the 10X Chromium 3′ library construction kit v2 following the manufacturer’s instruction. The libraries were then pooled and sequenced by Illumina HiSeq X Ten.

#### Single-cell RNA-seq data processing

Paired-end reads from Illumina HiSeq were processed and mapped to the mm10 mouse genome using Cell Ranger v2.0. Sequencing saturation as determined by Cell Ranger showed fairly even saturation across samples (young mice: 82.3%, 79.9%, 70.6%; aged mice: 91.3%, 78.6%, 73.3%, 85.1%). We applied stringent filters to eliminate cells with (1) UMI counts < 1,000 or > 60,000, (2) gene counts < 500 or > 2,500, and (3) mitochondrial gene ratio > 10%. This pre-filtering resulted in the detection of 27,998 genes in 13,684 cells, with approximately 1,200–2,800 cells from each sample. A median of 1,649 genes and 5,538 transcripts were captured per cell. The filtered data were then analyzed using Seurat v3 (Butler et al., 2018; Stuart et al., 2019). Cell doublets were estimated using Scrublet (Wolock et al., 2019) and DoubletFinder (McGinnis et al., 2019). Lineage trajectory analysis was performed on the luminal epithelial cells of each of the three young samples, as well as on the three samples combined, using STREAM (Chen et al., 2019). Cell cycle estimation was performed in Seurat using the default cell-cycle gene signatures.

#### Cell type-specific gene signatures

Cell type-specific gene expression signatures were identified as genes that exhibited expression levels > 1.25-fold greater (with adjusted *p-value*s < 0.05) than each of the other cell types in multiple pairwise differential expression analyses using Seurat’s FindMarkers function (Wilcoxon rank sum test). Similarly, gene expression signature for HS-AV luminal cells was generated by identifying genes with expression levels at least 1.25-fold higher or lower (with adjusted *p-value*s < 0.05) when compared to luminal HS cells and to luminal AV cells separately. The list of markers distinguishing M_a_ and M_b_ macrophages was generated by identifying genes with expression levels differing by at least 2-fold (with adjusted *p-value*s < 0.05). Heatmaps for visualizing marker gene expression were median-centered and down-sampled to 100 cells per cell type.

#### Differential gene expression across age groups

Differential gene expression analysis was performed to compare young and aged samples within each cell type by using a combination of Seurat’s FindMarkers function (Wilcoxon rank sum test) and zingeR-edgeR zero-inflated negative binomial analysis (Robinson et al., 2010; Van den Berge et al., 2018) for increased stringency. Differentially expressed genes were identified using three criteria: (i) Grouped analysis criteria: an expression difference of at least 1.5-fold and an adjusted *p-value* of < 0.05 in a grouped comparison of young mice (n = 3) versus aged mice (n = 4); (ii) Per-sample analysis criteria: an expression difference of at least 1.25-fold in all of the 12 possible combinations of young-versus-aged per-sample pairwise comparison; (iii) Cross-method criteria: only genes satisfying criteria (i) and (ii) in both FindMarkers and zingeR-edgeR analyses were included in the final list of differentially expressed genes. Only genes with expression detected in at least 10% of cells in either the young or aged population were analyzed. Heatmaps for visualizing the differentially expressed genes were median-centered and down-sampled to 200 cells per age group, or, in the case of M_b_ macrophages where fewer cells were detected, down-sampled to 20 cells per group.

#### Pathway analysis

Pathway analysis was performed on differentially expressed genes using MSigDB v7.0 (Liberzon et al., 2015; Subramanian et al., 2005) curated canonical pathway database. To ensure proper gene name mapping, all mouse gene names were converted to their human homologs using NCBI Homologene prior to analysis in MSigDB. Gene sets with FDR < 0.05 were deemed statistically significant. Up to top 30 enriched gene sets were shown.

#### Histology and immunostaining

Mouse mammary tissues were fixed in 10% neutral buffered formalin overnight. Paraffin embedding and sectioning were performed by the Rodent Histopathology Core at Harvard Medical School. Immunostaining was performed on n = 6 animals per age group. For immunofluorescence staining, the following antibodies were used: ACTA2/SMA (Dako M0851, 1:100), KRT15 (BioLegend 833904, 1:50), KRT14 (BioLegend 905301, 1:250; Abcam ab206100, 1:150), KRT8 (DSHB Troma-I, 1:100; Abcam ab192467, 1:100), ARG1 (CST 66297, 1:100), PR (CST 8757, 1:25; Dako A0098, 1:20), ER (Santa Cruz sc-542, 1:20; BioRad MCA1799T, 1:25), LTF (Bioss bs-5810, 1:50), MFGE8 (Thermo MA5–23913, 1:50; R&D Systems MAB2805, 1:50). LTF and MFGE8 antibodies were validated by staining lactating mammary glands. For immunohistochemistry staining, the following antibodies were used: PDGFRα (CST 3174, 1:200), CD163 (Abcam ab182422, 1:500), CD8 (CST 98941, 1:50). Images were analyzed in a blinded fashion using Fiji (Schindelin et al., 2012).

#### FACS analysis

For FACS analysis of mammary tissues, freshly dissected #2, 3, and 4 mammary glands from young (n = 8) and aged (n = 6) mice were digested as described above. Cells were then incubated with CD16/32 antibody (eBioscience 16–0161) to block Fc receptor and with rat serum (eBioscience 24–5555) to block non-specific binding, and then stained for 30 minutes with the following antibodies/reagents: CD45-FITC (BioLegend 103108, 1:200), CD31-FITC (BioLegend 102405, 1:50), TER119-FITC (BioLegend 116205, 1:100), EPCAM-BV605 (BioLegend 118227, 1:100), CD133-APC (BioLegend 141207, 1:50), CD14-PE (BioLegend 150106, 1:50), and DAPI (1 μg/mL). Cell sorting and analysis was carried out on a BD FACSAria II cell sorter and FlowJo using the following gating strategy: SSC-H/SSC-W singlets, FSC-H/FSC-W singlets, DAPI- live cells, CD45-/CD31-/TER119- Lin- cells, EPCAM-high luminal epithelial cells. The gated luminal population was then analyzed for expression of CD133 and CD14. For FACS analysis of organoid cultures, primary mammary organoids were generated from 3–4 month-old FVB female mice using the aforementioned tissue digestion protocol and propagated for six weeks in defined basement-membrane extract and culture media, as described previously (Rosenbluth et al., 2020). Organoids were digested into single cells using TrypLE, blocked with CD16/32 antibody and rat serum, and then stained with a similar antibody panel as described above. EPCAM-BV605 was omitted in order to allow for subsequent microscopy examination of cells double positive for CD133-APC and CD14-PE without introducing spectral overlapping from EPCAM-BV605.

### QUANTIFICATION AND STATISTICAL ANALYSIS

For cell type composition comparison between young and aged mammary glands in scRNA-seq data, statistically significant difference was determined by Fisher’s exact test and Chi-square test. We noted that because all proportions sum to one in scRNA-seq data, an increase in the proportion of one cell population will necessarily lead to a decrease in the proportions of other cell populations. However, alternative multivariate tests for compositional data analysis (e.g., Dirichlet-multinomial regression test) were not applied as they have increased type 2 error over type 1 error. For quantification by immunofluorescence staining, immunohistochemistry staining, and FACS analysis, statistically significant difference was determined by Student’s t test. Statistical analysis was performed by using the GraphPad Prism software, and *p-value*s < 0.05 (two-tailed) were deemed statistically significant. Bar graphs represent mean ± SEM.

## Supplementary Material

1

2

3

4

5

6

7

8

9

Data S1

## Figures and Tables

**KEY RESOURCES TABLE:** 

REAGENT or RESOURCE	SOURCE	IDENTIFIER
Antibodies		

ACTA2/SMA	Dako	Cat#M0851; RRID: AB_2223500
ARG1	Cell Signaling Technology	Cat#66297; RRID: AB_2799705
CD163	Abcam	Cat#ab182422; RRID: AB_2753196
CD8α	Cell Signaling Technology	Cat#98941; RRID: AB_2756376
KRT15	BioLegend	Cat#833904; RRID: AB_2616894
KRT14	BioLegend	Cat#905301; RRID: AB_2565048
KRT14	Abcam	Cat#ab206100; RRID: AB_2811031
KRT8	DSHB	Cat#Troma-I; RRID: AB_531826
KRT8	Abcam	Cat#ab192467; RRID: AB_2864346
PDGFRα	Cell Signaling Technology	Cat#3174; RRID: AB_2162345
PR	Cell Signaling Technology	Cat#8757; RRID: AB_2797144
PR	Dako	Cat#A0098; RRID: AB_2315192
ER	Santa Cruz	Cat#sc-542; RRID: AB_631470
ER	BioRad	Cat#MCA1799T; RRID: AB_2102069
LTF	Bioss	Cat#bs-5810; RRID: AB_11078952
MFGE8	Thermo	Cat#MA5–23913; RRID: AB_2609198
MFGE8	R&D Systems	Cat#MAB2805; RRID: AB_2297564
CD45-FITC	BioLegend	Cat#103108; RRID: AB_312973
CD31-FITC	BioLegend	Cat#102405; RRID: AB_312900
TER119-FITC	BioLegend	Cat#116205; RRID: AB_313706
EPCAM-BV605	BioLegend	Cat#118227; RRID: AB_2563984
CD133-APC	BioLegend	Cat#141207; RRID: AB_10898121
CD14-PE	BioLegend	Cat#150106; RRID: AB_2728189

Critical Commercial Assays		

Chromium Single Cell 3′ Assay	10x Genomics	N/A

Deposited Data		

scRNA-seq data	This study	GSE150580

Experimental Models: Organisms/Strains		

Mouse: mixed FVB, 129, C57BL/6J background	In house	N/A

Software and Algorithms		

R (3.5.1)	R project	RRID: SCR_001905
Python (2.7, python 3.7)	Python Software Foundation	RRID:SCR_008394
Cell Ranger v2	10x Genomics	RRID:SCR_017344
Seurat v3	Butler et al., 2018; Stuart et al., 2019	RRID:SCR_016341
Scrublet	Wolock et al., 2019	RRID:SCR_018098
DoubletFinder	McGinnis et al., 2019	RRID:SCR_018771
STREAM	Chen et al., 2019	N/A
zinger-edgeR	Van den Berge et al., 2018	N/A
NCBI Homologene	https://www.ncbi.nlm.nih.gov/homologene	RRID:SCR_002924
MSigDB v7.0	Liberzon et al., 2015; Subramanian et al., 2005	RRID:SCR_016863
Fiji	Schindelin et al., 2012	RRID:SCR_002285
FlowJo v10	BD Biosciences	RRID:SCR_008520
Prism v8	Graphpad software	RRID:SCR_002798

## Highlights

- scRNA-seq captures aging-associated alterations in mammary epithelia and stroma
- Identification of luminal and macrophage subpopulations reveals lineage complexity
- Aging correlates with alveolar maturation and potential cellular functional decline
- Aging is coupled to a potentially pro-tumorigenic and inflammatory microenvironment
