# Supplementary material for: Aging-Associated Alterations in Mammary Epithelia and Stroma Revealed by Single-Cell RNA Sequencing
Source: Cell Rep. Author manuscript; Available in PMC 2021 Feb 22. (PMC7898263; doi:10.1016/j.celrep.2020.108566)
Supplement: 1 [file NIHMS1658643-supplement-1.pdf]

Cell Reports, Volume *33*

Supplemental Information

Aging-Associated Alterations in Mammary Epithelia and Stroma Revealed by Single-Cell RNA Sequencing

Carman Man-Chung Li, Hana Shapiro, Christina Tsiobikas, Laura M. Selfors, Huidong Chen, Jennifer Rosenbluth, Kaitlin Moore, Kushali P. Gupta, G. Kenneth Gray, Yaara Oren, Michael J. Steinbaugh, Jennifer L. Guerriero, Luca Pinello, Aviv Regev, and Joan S. Brugge

Figure S1

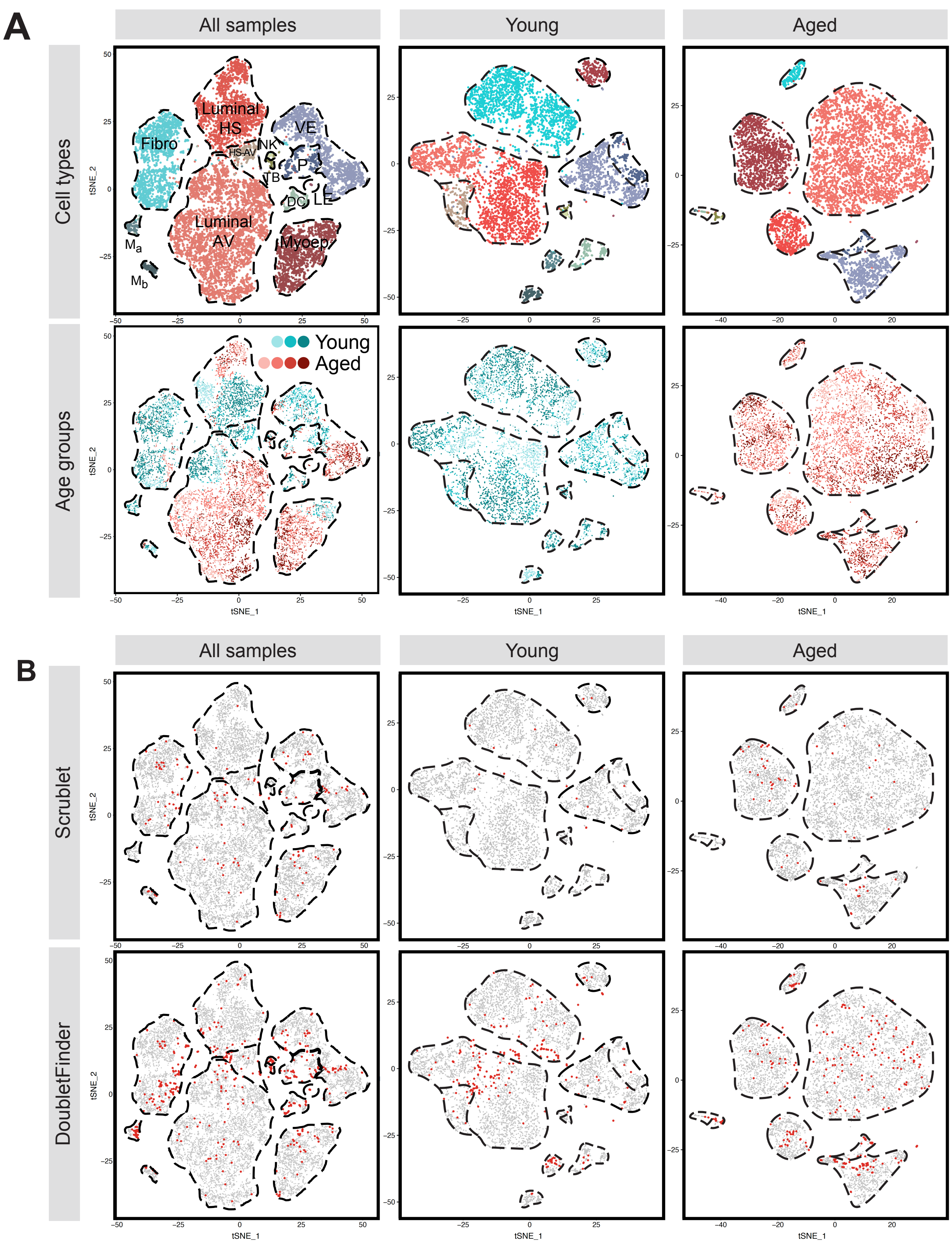
A
All samples
Young
Aged
Cell types
Age groups
Luminal HS
Fibro
VE
NK
HS-AV
P
TB
DC
LE
Luminal AV
Myoep
Ma
Mb
Young
Aged
tSNE_1
tSNE_2
B
Scrublet
DoubletFinder

Figure S1 (related to Figure 1). t-SNE Plots Showing Distribution of Samples and Identification of Potential Doublets

(A) t-SNE plots colored by age groups and by biological replicates.

(B) t-SNE plot showing predicted doublets in scRNA-seq data using Scrublet and DoubletFinder. If a cluster was overwhelmingly enriched with predicted doublets, it would likely represent an artifact doublet population generated by chance inclusion of two or more cells in a single droplet during the cell capturing step on the 10X Chromium platform. No such cluster is detected in our dataset.

Figure S2

A

Chi-square	Young (A)	Young (B)	Young (C)	Aged (A)	Aged (B)	Aged (C)	Aged (D)
**Young (A)**							
**Young (B)**	348						
**Young (C)**	555	729					
**Aged (A)**	926	990	1499				
**Aged (B)**	1718	1629	2669	141			
**Aged (C)**	1281	1179	1834	152	155		
**Aged (D)**	1227	1210	2039	135	32	105	

All p-values < 0.0001; df = 6

B

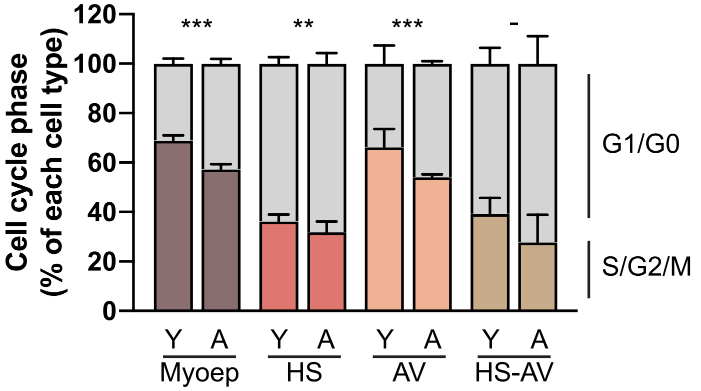

Young

	Myoep	HS	AV	HS-AV
**Myoep**				
**HS**	****			
**AV**	-	****		
**HS-AV**	****	-	****	

Aged

	Myoep	HS	AV	HS-AV
**Myoep**				
**HS**	****			
**AV**	*	****		
**HS-AV**	**	-	*	

Figure S2 (related to Figure 2A). Cell Type Proportions and Cell Cycle Fractions within and across Age Groups

(A) Cell type proportions are consistent within biological replicates and distinct across age groups. Table summarizes Chi-square test results for all per-sample pairwise comparisons of cell type proportions. The lower Chi-square values within each age group reflect consistency among biological replicates, whereas the higher Chi-square values across age groups reflect age-dependent differences.

(B) Bar graph of the fraction of cells in S/G2/M phases (colored bars) or G1/G0 phase (gray bars) within each epithelial cell type. Cell cycle estimation was performed using the default CellCycleScoring function in Seurat, with cells in G2/M/S considered cycling and G1/G0 non-cycling. All values represent mean + SEM. Statistically significant differences between young (Y) and aged (A) groups within a cell type are denoted above the bars (Fisher's exact test). Statistically significant differences between cell types within young mice or within aged mice are denoted in the respective tables (Fisher's exact test). All p-values are denoted by * (< 0.05), ** (<0.01), *** (<0.001), **** (<0.0001), or – (not significant). Within each cell type, n = 3 young mice and n = 4 aged mice were analyzed, except for HS-AV (n = 3+3), where such cells were not detected in one of the four aged mice. Abbreviations: Myoep, myoepithelial cells; HS, hormone-sensing luminal cells; AV, alveolar luminal cells.

Figure S3

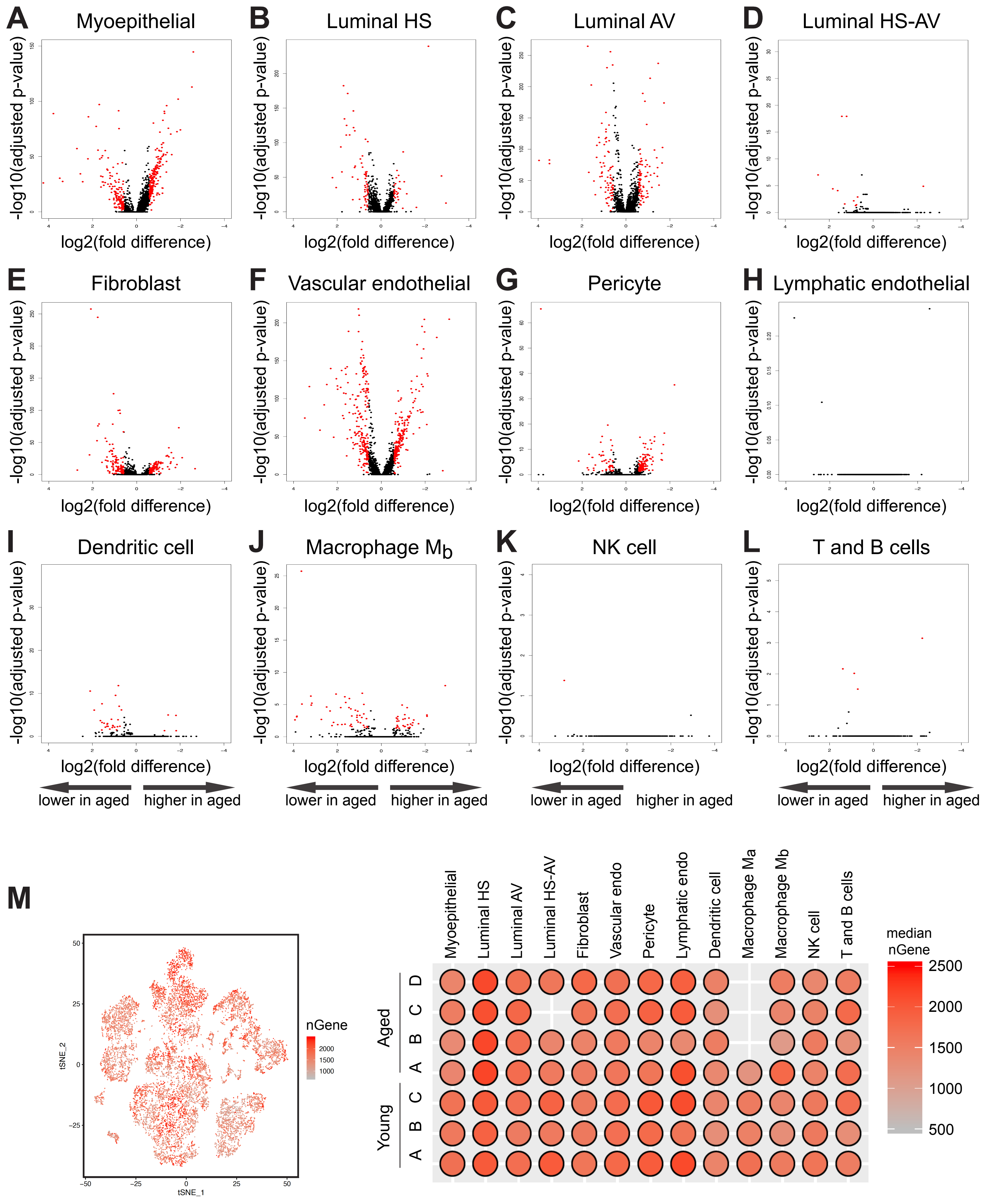
A Myoepithelial
B Luminal HS
C Luminal AV
D Luminal HS-AV
E Fibroblast
F Vascular endothelial
G Pericyte
H Lymphatic endothelial
I Dendritic cell
J Macrophage Mb
K NK cell
L T and B cells
-log10(adjusted p-value)
log2(fold difference)
lower in aged
higher in aged
M
tSNE_1
tSNE_2
nGene
2000
1500
1000
Myoepithelial
Luminal HS
Luminal AV
Luminal HS-AV
Fibroblast
Vascular endo
Pericyte
Lymphatic endo
Dendritic cell
Macrophage Ma
Macrophage Mb
NK cell
T and B cells
Aged
Young
D
C
B
A
median nGene
2500
2000
1500
1000
500

Figure S3 (related to Figures 3, 4, 6, and 7). Prevalent Alterations in Gene Expression in Aged Mammary Glands

(A-L) Volcano plots show expression levels of all detected genes in aged mammary glands (n = 4) compared to young mammary glands (n = 3) within the specified cell types. Genes with fold-difference above 1.5 and with adjusted *p*-value < 0.05 are highlighted in red. Differential expression analysis was not possible for M_a macrophages due to their extremely low abundance in aged mice.

(M) Number of genes (nGene) captured by scRNA-seq per cell (left) and median nGene within each cell type per sample (right). HS-AV cells and M_a macrophages were not detected in one or more aged mice.

Figure S4

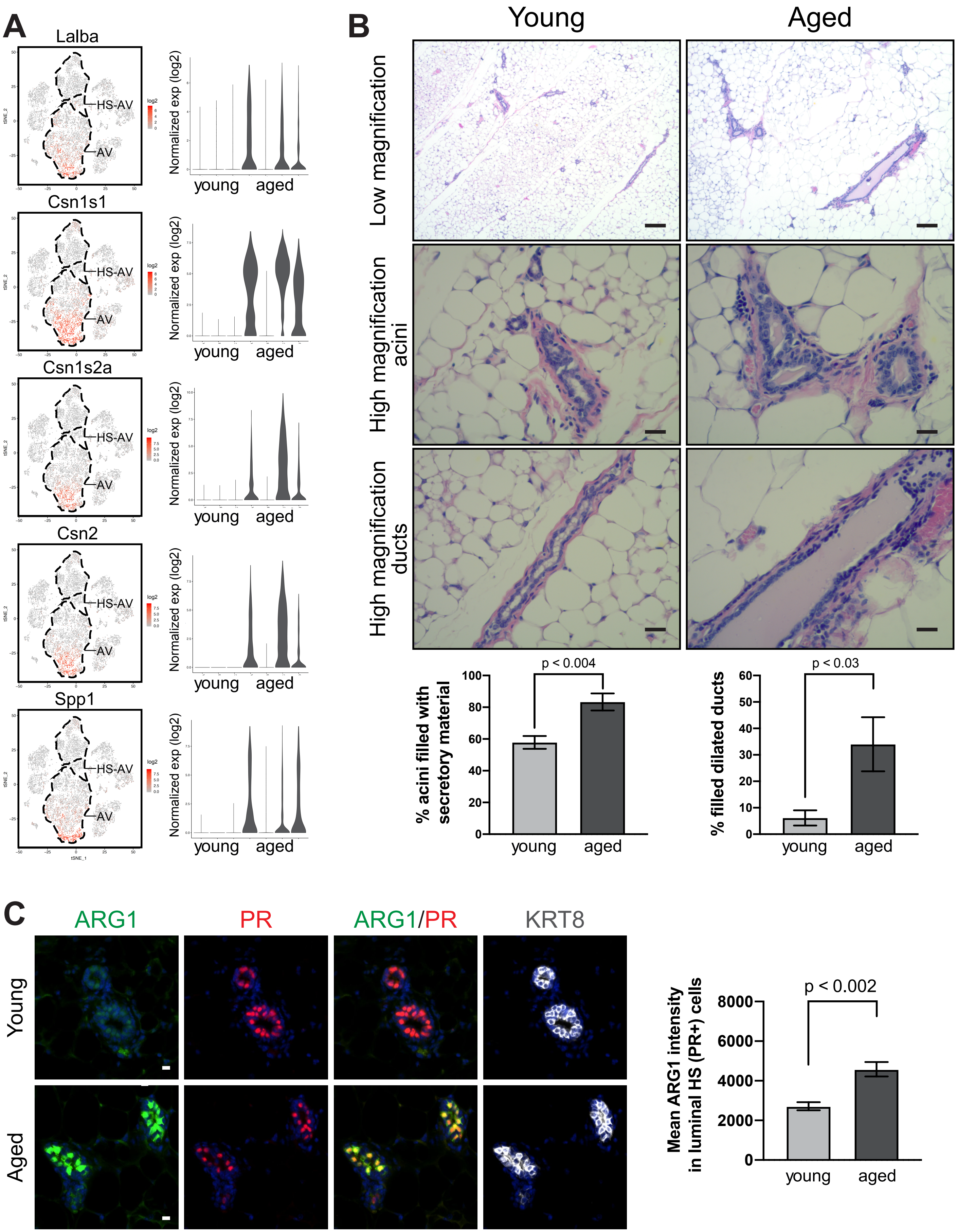
A
Lalba
Csn1s1
Csn1s2a
Csn2
Spp1
HS-AV
AV
Normalized exp (log2)
young aged
B
Young
Aged
Low magnification
High magnification acini
High magnification ducts
% acini filled with secretory material
p < 0.004
% filled dilated ducts
p < 0.03
C
ARG1
PR
ARG1/PR
KRT8
Young
Aged
Mean ARG1 intensity in luminal HS (PR+) cells
p < 0.002

Figure S4 (related to Figure 4). Alveolar and Hormone-Sensing Luminal Cell Changes with Aging

(A) t-SNE plots (left) showing the expression of milk biosynthesis-related genes in a subset of aged AV cells. Violin plots (right) showing that the expression of these genes is upregulated in AV cells in three out of four aged mammary glands compared to young glands.

(B) Representative H&E images and quantification of young and aged mammary acini and ducts. Aged mammary glands show increased secretory material in acini and dilated ducts. H&E at low magnification, scale bar = 100 µm; at high magnification, scale bar = 20 µm. For quantification of acini filled with secretory material, at least 20 acini per gland with diameter < 50 µm were analyzed. For quantification of ducts filled and dilated, at least 10 ducts per gland with cross-section length > 250 µm were analyzed. Ducts were considered dilated if the width:length ratio was > 1:4, i.e. 0.25. Analysis was performed on n = 6 animals per age group using Student's t test.

(C) Representative immunofluorescence staining pattern and quantification of ARG1 expression in luminal HS cells (stained as PR+) in young and aged mammary glands. KRT8 marks luminal cells. Scale bar = 10 µm. For quantification, at least 800 HS cells per mouse were analyzed. Analysis was performed on n = 6 animals per age group using Student's t test.

Figure S5

A

This Study		
Strain	Mixed FVB, 129, C57BL/6J mice	
Parity	Nulliparous	
Estrous staging	Diestrus	
Age	3-4	13-14 months
	n=3	n=4
Cell type	Young	Aged
HS	59%	12%
AV	29%	88%
HS-AV	12%	0.3%
Total luminal cells	100%	100%

Bach et al., 2017	
C57BL/6N mice	
Nulliparous	
Not estrous staged	
2 months	
	n=2
Cell type (orig. name)	Young
HS (HSd)	66%
AV (Lp)	21%
HS-AV (HSp)	13%
Total luminal cells	100%

Pal et al., 2017		
FVB/NJ mice		
Nulliparous		
	Estrus	Diestrus
2.5 months		
	n=2	n=2
Cell type (orig. name)	Young estrus	Young diestrus
HS (Mature luminal)	52%	53%
AV (Luminal progenitor)	33%	42%
HS-AV (Luminal intermediate)	10%	4%
Other 1 (Rare cluster)	1%	1%
Other 2 (Rare cluster)	3%	1%
Total luminal cells	100%	100%

Kanaya et al., 2019				
BALB/cj				
Nulliparous				
Ovariectomy at 9 weeks of age, one-week treatment before analysis				
5 months				
	n=2+2+2	n=2	n=2	n=2
Cell type (orig. name)	All	Vehicle	E2	E2+PBDE
HS (Mature luminal)	49%	28%	51%	53%
AV (Luminal progenitor)	14%	17%	17%	15%
HS-AV (Luminal intermediate)	36%	55%	33%	33%
Total luminal cells	100%	100%	100%	100%

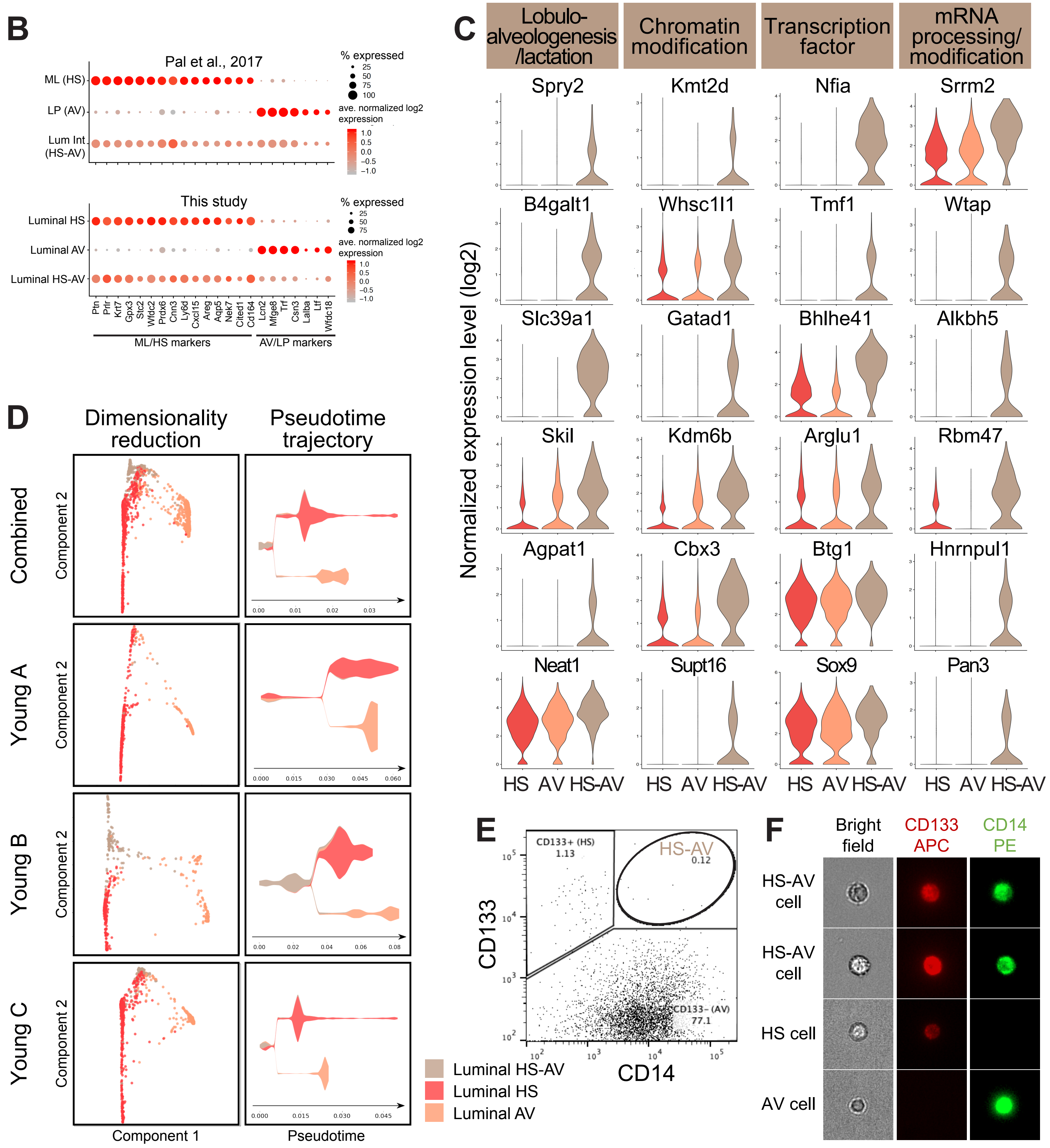

Figure S5 (related to Figure 5). HS-AV Gene Markers and Lineage Trajectory Analysis

(A) Proportions of luminal HS, AV, and HS-AV cells in young mice analyzed in this study compared to equivalent cell populations in other studies of young mice (adapted from Figure 6a in Pal et al., 2017 and Table 1 in Bach et al., 2017) and ovariectomized mice (adapted from Table 2 and Supplementary Figure 10a in Kanaya et al, 2019; E2, 17β-estradiol; PBDE, polybrominated diphenyl ethers).

(B) Expression of select HS and AV markers in Luminal Intermediate cells (Pal et al., 2017) and HS-AV cells (this study). Markers were partially adapted from Figure S4b of Pal et al., 2017.

(C) Violin plots of select genes identified in Figure 5B with expression enriched in HS-AV cells relative to HS cells and AV cells.

(D) Results from lineage trajectory analysis of luminal cells in young mice (n = 3), analyzed either in aggregate or as individual samples. In each case, HS-AV cells are localized to the bifurcation between the HS lineage and AV lineage.

(E) HS-AV cells co-expressing HS marker CD133 and AV marker CD14 are detected by FACS in organoid cultures of primary mammary cells.

(F) Example of HS-AV cells isolated by FACS and examined under the microscope to confirm that they are single cells with co-staining for CD133 and CD14, not doublets composed of CD133+ cell and CD14+ cell. CD133+ HS cells and CD14+ AV cells serve as controls.

Figure S6

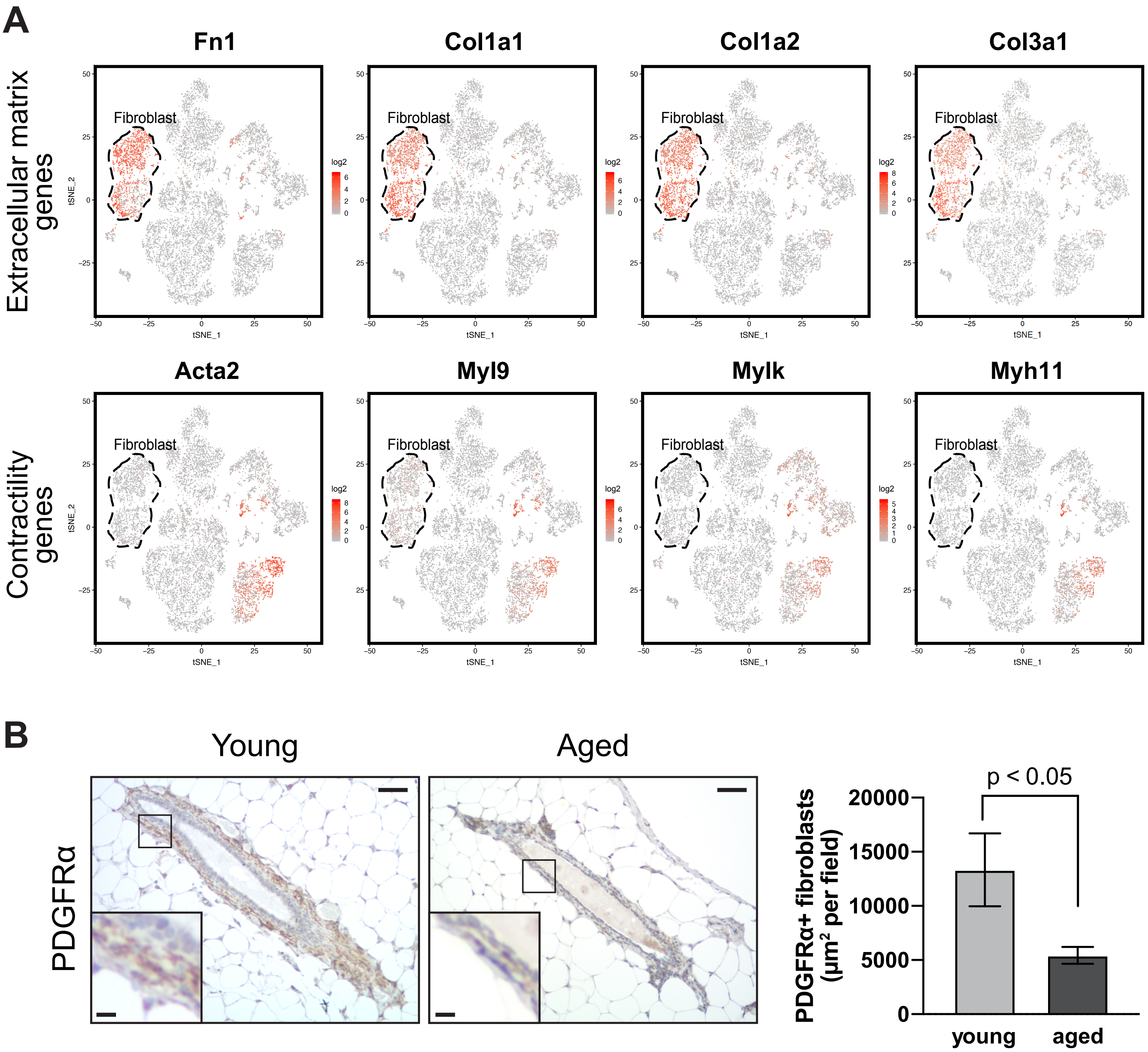

A
Fn1
Col1a1
Col1a2
Col3a1
Extracellular matrix genes
Fibroblast
log2
tSNE_1
tSNE_2
Acta2
Myl9
Mylk
Myh11
Contractility genes
B
Young
Aged
PDGFRα
p < 0.05
PDGFRα+ fibroblasts (μm² per field)
20000
15000
10000
5000
0
young
aged

Figure S6 (related to Figure 6). Fibroblast Markers and Immunostaining Validation of Altered Fibroblast Abundance with Age

(A) The fibroblasts captured by scRNA-seq express high level of extracellular matrix genes (top), but not contractility genes (bottom).

(B) Representative PDGFRα IHC staining of fibroblasts and quantification in young and aged mammary glands. Scale bar = 50 µm or 10 µm (inset). Analysis was performed on at least five regions per gland at 10X magnification, with n = 6 animals per age group, using Student's t test.

Figure S7

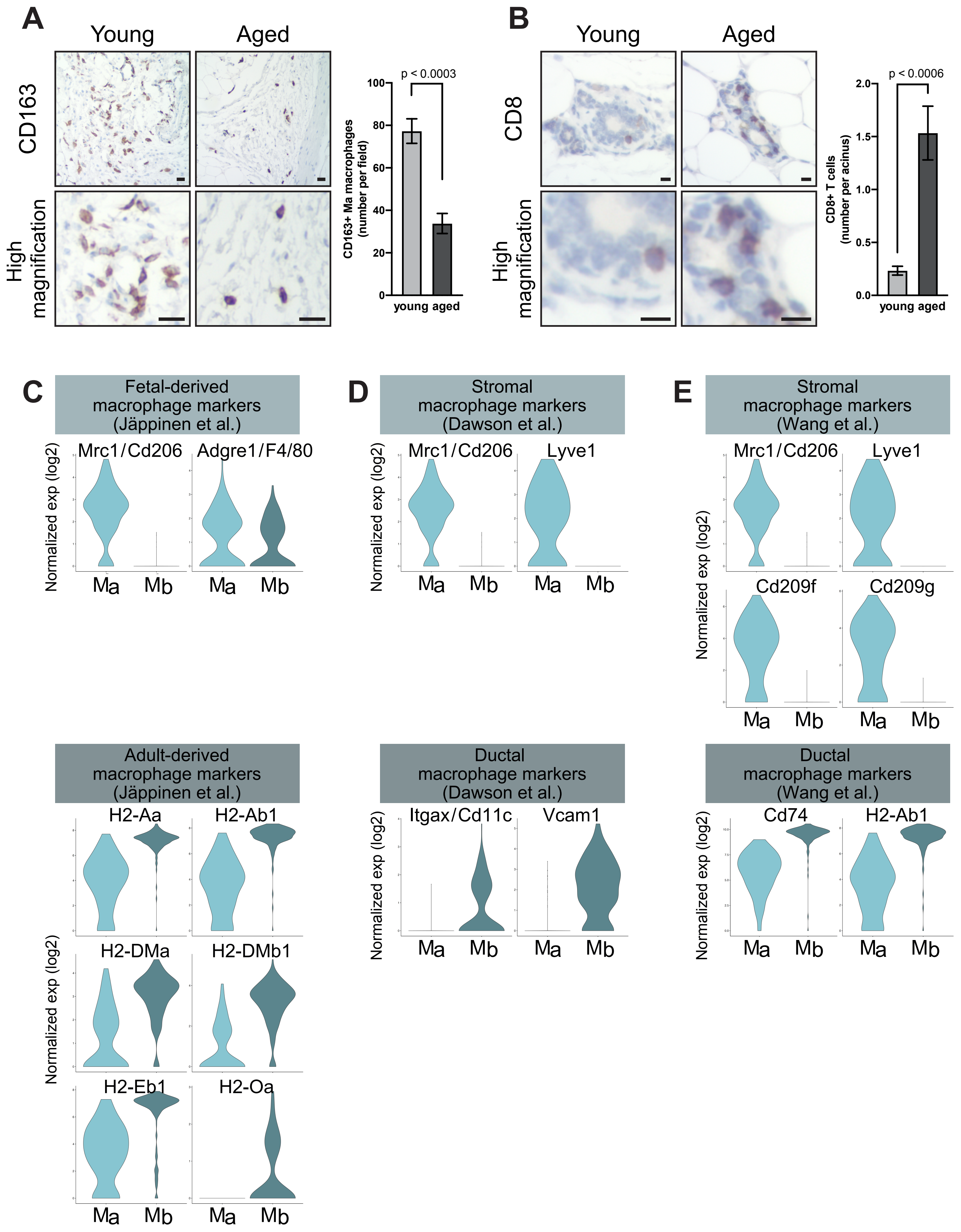
A
Young
Aged
CD163
High magnification
p < 0.0003
CD163+ Ma macrophages (number per field)
100
80
60
40
20
0
young aged
B
Young
Aged
CD8
High magnification
p < 0.0006
CD8+ T cells (number per acinus)
2.0
1.5
1.0
0.5
0.0
young aged
C
Fetal-derived macrophage markers (Jäppinen et al.)
Mrc1/Cd206
Adgre1/F4/80
Normalized exp (log2)
Ma
Mb
D
Stromal macrophage markers (Dawson et al.)
Mrc1/Cd206
Lyve1
E
Stromal macrophage markers (Wang et al.)
Mrc1/Cd206
Lyve1
Cd209f
Cd209g
Adult-derived macrophage markers (Jäppinen et al.)
H2-Aa
H2-Ab1
H2-DMa
H2-DMb1
H2-Eb1
H2-Oa
Ductal macrophage markers (Dawson et al.)
Itgax/Cd11c
Vcam1
Ductal macrophage markers (Wang et al.)
Cd74
H2-Ab1

Figure S7 (related to Figure 7). Immunostaining Validation of Altered Immune Cell Abundance and Comparison of Macrophage Markers

(A) Representative IHC staining of CD163+ M_a macrophages and quantification in young and aged mammary glands. Scale bar = 25 µm. Analysis was performed on 3-6 ECM-rich stromal regions per gland at 10X magnification, with n = 6 animals per age group, using Student's t test.

(B) Representative IHC staining of CD8+ T cells and quantification in young and aged mammary glands. Scale bar = 10 µm. Analysis was performed on at least 15 acini per gland at 20X magnification, with n = 6 animals per age group, using Student's t test.

(C) M_a macrophages express higher levels of markers for fetal-derived macrophages, whereas M_b macrophages express higher levels of markers for adult-derived macrophages. Markers were reported by Jäppinen et al., 2019.

(D-E) M_a macrophages are distinguished by stromal macrophage markers, and M_b macrophages by ductal macrophage markers. Markers were reported by Dawson et al., 2020 (D) and Wang et al, 2020 (E).
