# Supplementary material for: Aging-Associated Alterations in Mammary Epithelia and Stroma Revealed by Single-Cell RNA Sequencing
Source: Cell Rep. Author manuscript; Available in PMC 2021 Feb 22. (PMC7898263; doi:10.1016/j.celrep.2020.108566)
Supplement: Data S1 [file NIHMS1658643-supplement-Data_S1.pdf]

Cell Reports

Aging-associated alterations in mammary epithelia and stroma revealed by single-cell RNA sequencing

--Manuscript Draft--

**Manuscript Number:**	CELL-REPORTS-D-20-03092R3
**Full Title:**	Aging-associated alterations in mammary epithelia and stroma revealed by single-cell RNA sequencing
**Article Type:**	Resource Article
**Keywords:**	aging, mammary epithelia and stroma, luminal and myoepithelial cells, fibroblasts, endothelial cells, macrophages, single-cell RNA-seq
**Corresponding Author:**	Joan Brugge<br>Harvard Medical School<br>Boston, MA UNITED STATES
**First Author:**	Carman Man-Chung Li, Ph.D.
**Order of Authors:**	Carman Man-Chung Li, Ph.D.
	Hana Shapiro
	Christina Tsiobikas
	Laura M. Selfors
	Huidong Chen
	Jennifer Rosenbluth
	Kaitlin Moore
	Kushali P. Gupta
	G. Kenneth Gray
	Yaara Oren
	Michael J. Steinbaugh
	Jennifer L. Guerriero
	Luca Pinello
	Aviv Regev
	Joan S. Brugge
**Abstract:**	Aging is closely associated with increased susceptibility to breast cancer, yet there have been limited systematic studies of aging-induced alterations in the mammary gland. Here we leverage high-throughput single-cell RNA-sequencing to generate a detailed transcriptomic atlas of young and aged murine mammary tissues. By analyzing epithelial, stromal, and immune cells, we identify age-dependent alterations in cell proportions and gene expression, providing evidence that suggests alveolar maturation and physiological decline. The analysis also uncovers potential pro-tumorigenic mechanisms coupled to the age-associated loss of tumor suppressor function and change in microenvironment. In addition, we identify a rare, age-dependent luminal population co-expressing hormone-sensing and secretory-alveolar lineage markers, as well as two macrophage populations expressing distinct gene signatures, underscoring the complex heterogeneity of the mammary epithelia and stroma. Collectively, this rich single-cell atlas reveals the effects of aging on mammary physiology and can serve as a useful resource for understanding aging-associated cancer risk.
**Suggested Reviewers:**	Christine Watson, Ph.D.<br>Professor, University of Cambridge<br>cjw53@cam.ac.uk<br>Dr. Watson is an expert in mammary gland biology, especially pertaining to mammary

Powered by Editorial Manager® and ProduXion Manager® from Aries Systems Corporation

	luminal and basal/myoepithelial lineage development.
	Camila dos Santos, Ph.D.<br>Assistant Professor, Cold Spring Harbor Laboratory<br>dossanto@cshl.edu<br>Dr. dos Santos is an expert in mammary gland biology, especially pertaining to the luminal alveolar lineage.
	Benjamin Spike, Ph.D.<br>Assistant Professor, Huntsman Cancer Institute<br>benjamin.spike@hci.utah.edu<br>Dr. Spike is an expert in mammary gland biology, with considerable experience pertaining to mammary epithelial lineage development and single-cell RNA-seq analysis.
	Mark LaBarge, Ph.D.<br>Professor, E O Lawrence Berkeley National Laboratory<br>malabarge@lbl.gov<br>Dr. LaBarge is an expert in mammary gland biology, especially pertaining to mammary epithelial and stromal development and aging.
	Ian Macara, Ph.D.<br>Professor, Vanderbilt University<br>ian.g.macara@vanderbilt.edu<br>Dr. Macara is an expert in mammary gland biology, especially pertaining to mammary epithelial lineage development and breast cancer.
	Jeffrey Rosen, Ph.D.<br>Professor, Baylor College of Medicine<br>jrosen@bcm.edu<br>Dr. Rosen is an expert in mammary gland biology, especially pertaining to mammary epithelial and stromal development and breast cancer.
**Opposed Reviewers:**	Jane Visvader, Ph.D.<br>Professor, Walter and Eliza Hall Institute of Medical Research<br>visvader@wehi.edu.au<br>There is a conflict of interest due to a close overlap between our works.
	Geoffrey Lindeman, Ph.D.<br>Professor, Walter and Eliza Hall Institute of Medical Research<br>lindeman@wehi.edu.au<br>There is a conflict of interest due to a close overlap between our works.

Powered by Editorial Manager® and ProduXion Manager® from Aries Systems Corporation

LUDWIG
CANCER
RESEARCH

HARVARD
MEDICAL SCHOOL

BLAVATNIK INSTITUTE
CELL BIOLOGY

Joan S. Brugge, Ph. D.
Louise Foote Pfeiffer Professor of Cell Biology
Department of Cell Biology
Director, Ludwig Center at Harvard
Email: joan_brugge@hms.harvard.edu

Tel: (617) 432-3974
Fax: (617) 432-3969
240 Longwood Ave
Boston MA 02115

Nov 23, 2020

Thomas Pietri, Ph.D.
Scientific Editor
Cell Reports

Dear Dr. Pietri,

Thank you for your assistance in processing our manuscript for publication. We have made the following requested changes to the manuscript:

1. The graphical abstract is now of the correct size (1200 pixels square at 300 dpi), with larger fonts.

2. The supplemental R script is now mentioned after the STAR Methods section.

We have not made any other changes to the final files.

Please let us know if you have further questions or concerns.

Sincerely,

Joan S. Brugge, Ph.D.

Reviewer #1:

In this study the authors present a detailed atlas of the transcriptional signatures of mouse mammary cells, both epithelial and stromal, in young or aging mice. Overall, this is a comprehensive exploration of the cell populations that compose the mouse mammary gland and it validates findings from previous single cell analyses. The value of this work as a resource for future research is clear and should be applauded. I particularly appreciated the efforts in identifying all different cell types composing the mammary stromal cells.

We thank the reviewer for appreciating the value of this work as a resource for the research community.

However, the results are limited to one single 10X scRNAseq experiment, performed on two conditions (young and aged mice)

We would like to point out that the scRNA-seq experiment contained multiple biological replicates (n = 3 young mice and n = 4 aged mice), which show largely consistent trend across replicates (Table S2A). The number of replicates used is well on par with, if not better than, the going standard for single-cell RNA-seq studies. In addition, we have validated the key observations with orthogonal experiments, including flow cytometry, immunofluorescence, and immunohistochemistry.

… and I cannot find any conceptual advance over the previous literature. In particular regarding luminal cell heterogeneity, the authors reach the same conclusions as studies from other groups, see i.e. Pal et al. or Bach et al. Nat. Comm. 2017. Also, I do not think any definitive conclusion on lineage relationships can be drawn in the absence of more in vivo tracing experiments.

We believe that the conceptual advances in this study include the following, some of which were also pointed out by the other two reviewers:

- The high-resolution single-cell profiling presented here has captured the dynamic transcriptomic landscapes in young and aged mammary tissues and revealed gene expression patterns of functional maturation and physiological decline associated with aging. The strikingly consistent pattern observed across multiple replicates suggests a defined biological program of aging that is not merely a result of stochastic deterioration. We have also uncovered gene expression signatures of potential pro-tumorigenic mechanisms coupled to the age-associated loss of tumor suppressor function and the development of an inflammatory, immunosuppressive microenvironment. These data underscore pathways that mediate epithelial-stromal crosstalk in the aging process.

- Our study captured and characterized a rare and previously underappreciated epithelial cell population co-expressing markers of both hormone-sensing and alveolar lineages, which we termed HS-AV cells. These two mammary luminal lineages had been proposed to be completely independent based on lineage tracing studies (Van Keymeulen et al., *Cell Reps.* 2017; Wang et al., *Cell Reps.* 2017), whereas other initial mammary single-cell RNA-seq studies captured such hybrid cells without characterizing them in detail, leading to confusion in the field about whether these cells are doublet artifacts. (Bach et al, *Nat. Commun.* 2017; Pal et al, *Nat. Commun.* 2017). Here, we have confirmed the existence of such hybrid cells using *in situ* immunofluorescence staining and FACS analyses. We also demonstrated that

their abundance significantly diminished with age. These findings reveal a greater complexity of mammary epithelial differentiation than previously understood.

- The inclusion of stromal and immune single-cell profiles in our analyses complements initial single-cell RNA-seq studies that focused only on mammary epithelia (Bach et al, *Nat. Commun.* 2017; Pal et al, *Nat. Commun.* 2017). Importantly, our analysis of the immune compartment revealed two distinct mammary macrophage populations that bridge the gap between two recent mammary macrophages studies (Jappinen et al., *Nat. Commun.* 2019; Dawson et al., *Nat Cell Biol* 2020). Furthermore, we demonstrated that one of these populations exhibited properties associated with tumor-associated macrophages and acquired expression of immunosuppressive genes with age. These data captured the dynamic immune landscape during mammary gland aging.

Concerning the effects of aging on the mammary cellular composition, this is an interesting, relatively understudied question that would have been much more relevant if human mammary glands would have been interrogated, instead of mouse. Based on this major lack of novelty, I consider this study a methodical descriptive work which would be of interest for a more specific journal than Cell Reports.

We acknowledge that the murine mammary gland is not an exact replicate of the human breast, in spite of their overwhelming similarities in structure and development.

However, mouse model allows us to overcome a major problem of human studies in the degree of control over physiological variables, such as pregnancy history, hormonal cycle, diet, life-style differences, genetic background, etc. Many of these variables are understandably difficult to control for in human studies. In particular, high number of pregnancies and disrupted hormonal cycle have significant impact on mammary biology and yet are also closely associated with older age, making it very challenging to separate these confounding factors in aging study of the mammary glands. To confound matters further, information on pregnancy history, hormone cycles, and other variables are often not readily available for human samples, making it difficult to draw definitive conclusions.

Mouse analysis offers a major advantage in this regard. This study has carefully controlled for the animals' pregnancy history, hormonal cycle, husbandry environment, genetic background. This allows for identification of the effects specific to aging. As such, we believe that mouse studies can complement human studies to provide a better understanding of aging. Importantly, the cell- and gene-level profiles of aged mammary glands are consistent across multiple independent replicates, suggesting that the intrinsic effects of aging may follow a defined developmental program instead of being due to stochastic degeneration.

We have highlighted the relevant text in the manuscript to better illustrate this point. (Please see blue highlights at the end of page 2 and the second paragraph on page 10.)

Reviewer #2:

In this manuscript, the authors utilized scRNA-seq approaches to determine the impact of aging on mammary gland cellular compositions and their molecular characteristics. They performed a comprehensive analysis of both epithelial and stromal cells and found aging-associated alterations at both the cell level and transcriptomic levels. Interesting changes were found in the abundance of certain cell types, such as the expansion of the epithelial compartment, the marked increase of luminal AV cells, and the change of ratios of stromal and ductal macrophages. Additionally, the authors identified a population of luminal cells that have mixed features of HS and AV lineages (HS-AV cells). Interestingly, these HS-AV cells were greatly diminished in the aging glands. The author also identified significant gene-expression changes for several cell types that could contribute to increased cancer susceptibility. Overall, this is an interesting study providing much-needed information on the cellular and transcriptomic atlas of aging mammary glands. The experiments were carefully done, and the data are generally of high quality. While the above makes the work significant and novel, authors should attempt to address the following points to further improve the study.

We greatly appreciate the reviewer's highly positive comments on this study.

1. There is a big difference between the frequencies of HS-AV cells determined by scRNA-seq and by immunostaining or flow cytometry. In addition to the possibility of post-transcriptional/translational regulation suggested by the authors, it is also possible that these markers only identify a subset of the HS-AV cells. It will help to use some of the highly upregulated genes shown in Fig 5B to further evaluate the abundance and location of HS-AV cells in the mammary glands.

We completely agree with the reviewer about this technical difference between scRNA-seq and immunostaining/flow cytometry. We have now revised the text to better clarify this point. (Please see green highlight on page 8.)

We did make an exhaustive attempt to identify a broader HS-AV marker from the upregulated genes shown in Figure 5B, but were unfortunately unable to find a good candidate. Most of these markers were not appropriate for immunostaining or flow cytometry, as they have substantial baseline expression in HS or AV cells despite significant increase in HS-AV cells. This expression pattern makes it challenging to define a cutoff threshold to distinguish HS-AV cells from HS or AV cells by tissue staining or flow cytometry. For the few remaining markers, there is very limited options of antibodies that are compatible with immunostaining or flow cytometry, and none passed our antibody validation to be usable.

To directly compare the detection of these cells in our study to previous scRNA-seq reports on the mouse mammary gland (Bach et al., 2017 and Pal et al., 2017), we included a supplemental figure which shows that the proportion of HS-AV cells are fairly consistent. (Please see new Figure S5A.) This gives us confidence about the validity of the HS-AV cell population, even though we were only able to detect a subset of them using orthogonal approaches such as immunostaining and flow cytometry.

2. How do the HS-AV cells described here compare to the previously described luminal intermediate cells in Pal et al. 2017? Do they share similar signatures?

To compare the HS-AV cells identified in this study to the luminal intermediate cells in Pal et al., we have applied their luminal intermediate gene signature (as shown in Figure S4b of Pal et al., 2017) to our HS-AV cells. The result (Figure S5B) shows strong similarity between these two populations, suggesting that they are the same cell type.

In addition, as mentioned above, we have generated a table to summarize the proportions of HS-AV cells in this study as well as similar cell population (i.e. cells co-expressing HS and AV markers) in Pal et al. and Bach et al. (Figure S5A). The percentages across these studies are fairly comparable, further suggesting that the population is the same despite under different nomenclature.

3. It will be interesting to check whether the Ma and Mb macrophages have different M1/M2 polarization, as M2 macrophages are often associated with tumor-promoting microenvironments.

This is an interesting point. We found no clear M1/M2 association based on the Ma/Mb macrophage associated genes. Both Ma and Mb macrophage populations express a mixed subset of makers that would classically be considered as M1 or M2.

For example:
- Ma macrophages express higher levels of an M1 marker *Siglec1/Cd169* than Mb macrophages, and moderate levels of two other M1 markers *CD86* and *CD74*. However, they also express higher levels of M2 markers *Cd163*, *Mrc1/Cd206*, *Retnla*, *Mgl2*, and *Folr2* than Mb macrophages.
- Mb macrophages express higher levels of M1 markers *Fcgr1/Cd64*, *Cd86*, and *Cd74*, as well as the M2 markers *Mmp12*, *Mmp13*, and *Mmp14*.

We believe that this lack of clear M1/M2 distinction is consistent with the fact that these macrophages are detected in a normal, non-tumor bearing microenvironment. We have revised the text to clarify this point. (Please see page 9).

Reviewer #3:

Summary: <u>This is a well-written Resource</u> describing the single cell transcriptomic analysis of normal murine mammary glands in young and aged (perimenopausal) mice. Many single cell analyses have been reported for murine mammary development, but this is the only one to my knowledge that <u>addresses the issue of aging differences at the single cell level</u>. <u>Adding value, the authors take an "all-comers" approach</u> in that they do include all cell types classifiable using known markers in unsorted cell populations. <u>This offers the promise of providing a wholistic view of dynamic cellular changes that occur in the mammary gland during aging</u>.
<u>In general, the analyses are done very well.</u> There are some significant issues that need to be addressed, however, to firm up some of the conclusions that are made.

We very much appreciate the reviewer's positive comments on the approach and value of this study.

Comments:
1a. One of the major findings in this paper has to do with the identification of a putative hybrid cell type exhibiting transcriptomic similarities between hormone-sensing and mature alveolar luminal cells. This cell type is referred to as the "HS-AV" cluster. While the authors state that this cell type transcriptomically exhibits "differentiation plasticity" and "the potential to differentiate into HS and AV cell types", they are also careful to moderate the statement that lineage tracing does not support such a possibility with the caveat that a rare population might not be detectable by lineage tracing. <u>The alternative possibility is that this HS-AV population may represent a transient state of cells differentiating from LP to ML, instead of being a dedicated self-renewing progenitor population. This should be stated more clearly.</u>

We completely agree, and have now revised the text in the Discussion to state this more clearly: "Finally, it remains possible that HS-AV cells represent a transient intermediate between the HS and AV populations, for instance, during differentiation of HS cells from AV cells, as proposed by Pal et al. (Pal et al., 2017)." (Please see page 13.)

Also, while the authors state that this was an "unanticipated" cell type to find, cells with characteristics intermediate to the more commonly defines "ML+LP groups" have been reported by Pal et al. in WT FVB young adult mice and by Kanaya et al in an ovariectomy menopause model (https://www.nature.com/articles/s42003-019-0618-9). However, as the terminology used to described cell types in this paper is different than that used previously (e.g., here no markers were used for sorting, and the cell types are referred to only on the basis of gene expression identifiers), it is very difficult to deduce whether these cells are indeed the same. <u>The authors should change the way this population is described to reflect its prior identification, and they should present a figure or table where they show the relationships between their different luminal cell types and those reported previously (especially when referring to putative progenitors) to enable readers to more clearly see which are the same and which may be different.</u>

We thank the reviewer for making this suggestion. We did not intend to suggest that this population has not been observed before, but we feel that it is has not been well characterized and therefore warrants further analysis.

We have changed the description of the HS-AV cell population from “unanticipated” to “lesser-known”. (page 4)

We have also added a statement to further clarify this point:
“Similar cells co-expressing HS and AV gene signatures have also been identified in young mice by Pal et al. and Bach et al. at comparable proportions (Bach et al., 2017; Pal et al., 2017) and in ovariectomized mice by Kanaya et al. at higher abundance (Kanaya et al., 2019) (Figures S5A and S5B), but they have not been further characterize.” (page 7)

In addition, we have created a summary chart (Figure S5A) to show the relationship between the different luminal cell types in this study and in Bach et al., Pal et al., and Kanaya et al., namely, HS (ML), AV (LP), and HS-AV (luminal intermediates). The proportions are highly comparable across studies within normal young mice (this study, Pal et al., and Bach et al.), and are altered in aged mice (this study) and in ovariectomized mice (Kanaya et al.).

Finally, we have added a clarification in the introduction to match the luminal nomenclatures used in this study (HS, AV) and other studies (ML, LP). (page 2)

For the nomenclature of HS-AV cells, we would prefer to refrain from calling them “luminal intermediate” cells as in Pal et al. and Kanaya et al. It remains to be proven whether these cells are indeed intermediate in the differentiation transition from AV cells (LP) to HS cells (ML), or whether they represent an independent lineage. We believe that the term HS-AV is more neutral.

1b. There is considerable variance in the abundance of the HS-AV population in the 3 young mice analyzed (~ 0%, 25%, and 7%). These data suggest that this subset might represent a transient status. In order to be more certain about the estimated abundance of this population, <u>the authors should show a comparison of nUMI or nGene per cell between HS-AV, AV, and HS clusters in each young mouse sample</u>. In figure S1A, the young mice sample which does not have the HS-AV cluster seems to cluster separately from the other two young samples. <u>Could this be due to sequencing depth differences?</u> This comparison would also further address the issue of whether this population could represent doublets which should have higher numbers of reads per cell.

We have now added a comparison of nGene between HS-AV, AV, HS, as well as all other cell populations in each young and aged mouse sample (Figure S3M). As shown in the figure, the nGene level is fairly comparable across samples, suggesting that these populations do not likely represent doublets.

The sequencing saturation for the samples are also fairly consistent, as listed below. Thus, any sample-to-sample variation in the clustering pattern was unlikely a result of difference in sequencing depth. We have now added the sequence depth information to the Methods section (page 19)

- Young (A), 82.3% saturation
- Young (B), 79.9% saturation
- Young (C), 70.6% saturation
- Aged (A), 91.3% saturation
- Aged (B), 78.6% saturation
- Aged (C), 73.3% saturation
- Aged (D), 85.1% saturation

We noted that the young mice do have variations in HS-AV abundance within the epithelial compartment, despite their overall higher abundance than aged mice (as shown in table S2):

- Young (A), 1.3%
- Young (B), 23%
- Young (C), 8%
- Aged (A), 0.5%
- Aged (B), 0.3%
- Aged (C), 0.0%
- Aged (D), 0.3%

We believe this represents normal sample-to-sample variation, not a result of different sequencing depths.

2. Given the timepoint selected for the aged mice, it is possible that they were going through menopause. However, the mixed background of the mice used makes it difficult to be certain since the expected menopausal age varies significantly by strain. Analyzing mice at diestrus is not particularly informative because vaginal cytology can appear as persistent diestrus as mice enter menopause. The authors need to use other criteria to support their contention that the mice analyzed were indeed perimenopausal. Circulating estrogen levels would be optimal but if this is not possible, the authors need to explore other means of defining the extent to which these mice have entered menopause. Also, while it is likely not possible due to COVID restrictions, it would have been very interesting to have had one more time point of analysis to show a "trajectory" of the changes as mice proceed into an "aged mammary phenotype" and perhaps a later time point to see changes in objectively "old" mice.

We thank the reviewer for pointing out the concern about persistent diestrus in aged mice. We were aware that very old mice can undergo apparent persistent estrus or diestrus as they enter menopause. Therefore, we were careful to perform daily vaginal cytology for multiple days to make sure that these mice were indeed undergoing active estrous cycling, not stuck in diestrus. Given this precaution, menopause-related acyclicity is very unlikely to be a concern in this study. We have now clarified this point in the Methods section (page 18).

We feel that the current time points of 3-4 months and 13-14 months offer insight into the aged peri-menopausal state. Regarding a later time point, given the concern that very old mice frequently have multiple confounding issues (e.g. persistent diestrus, spontaneous tumors, galactorrhea, etc.), it will be challenging to analyze age-specific effects in even older mice. In addition, the COVID-related restrictions also make additional time point analysis very difficult.

3. Variation in the proportion of different cell types is often seen in single cell analyses. Such variations are often driven by technical factors instead of biology. In this light, Figure 1B and 1C show multiple clearly separated sub-clusters within the epithelial population. However, Figure S1A also shows obvious batch effects among biological replicates. Of significance, the HS-AV cluster appears to be mainly composed of cells from two of the young mice, is absent in the third, and the proportion varies significantly between the two mice in which it is present. Comparing the proportional changes from data with significant batch effects may be misleading. It is especially problematic when clusters with small cell numbers (such as the immune cells, and HS-AV cells) are directly compared for fold-changes in gene expression. The authors need to critically discuss this potential issue and tone down the inferences drawn from the proportional changes in gene expression. Orthogonal analytic methods also should be used to substantiate the conclusions drawn. For example, the statement "…its

increased expression in aged Mb macrophages is consistent with the higher infiltration of NK cells and T cells observed in aged mammary glands" should be supported by actual staining of lymphocyte infiltration instead of from the potentially inaccurate inferences of transcription data on rare populations with low depth transcript coverage.

We thank the reviewer for raising this concern on the observed changes of cell type proportions. We would like to point out that all scRNA-seq analyses were performed with internal normalization per sample in addition to global normalization across samples. Therefore, the relative abundance per cell type within each mouse should not be largely impacted by batch effects. We believe that the clustering pattern observed in Figure S1A was primarily driven by age-related differences.

As mentioned above, we noted sample-to-sample variation across biological replicates, such as variation in the HS-AV abundance within the epithelial compartment of young mice, despite their overall higher abundance than aged mice (as shown in table S2):
- Young (A), 1.3%
- Young (B), 23%
- Young (C), 8%
- Aged (A), 0.5%
- Aged (B), 0.3%
- Aged (C), 0.0%
- Aged (D), 0.3%

We believe this represents normal sample-to-sample variation in different animals.

To address this concern further, we have revised the text to specify that the proportional changes are “inferred from the transcriptomic data”. (page 4)

In addition, we have taken the reviewers suggestions and performed orthogonal validation of the proportional changes for additional cell types. These new data, together with existing orthogonal analyses, have validated the proportional changes observed by scRNA-seq.
- HS-AV : validated by immunofluorescence and FACS (at initial submission, Figures 5G-5K)
- HS : validated by FACS (at initial submission, Figure 4E)
- AV : validated by FACS (at initial submission, Figure 4E)
- Fibroblasts : validated by immunohistochemistry staining (new, Figure S6B)
- Ma macrophages : validated by immunohistochemistry staining (new, Figure S7A)
- T cells : validated by immunohistochemistry staining (new, Figure S7B)

Notably, the proportional increase of T cell abundance with age is highly consistent between scRNA-seq data (Figure 7E) and tissue staining (Figure S7B).

In addition to T cells, we have also performed immunohistochemistry staining on NK cells, but we observed extremely low NK cell count overall, in accordance with scRNA-seq data (<15% of total immune cells). This is in contrast to T cells, which were readily detectible by immunostaining. We therefore decided that it is more meaningful to focus our discussion on T cell increase than NK cells. We have now revised the text accordingly. (page 10)

4. The almost complete loss of fibroblast and macrophage populations in the aged mice is surprising. To rule out possibilities that those cells were lost during single cell preparation procedures, the author need to provide staining of the virgin vs aged mammary glands to confirm such dramatic decreases of fibroblasts and macrophages in aged mice.

We thank the reviewer for this suggestion. As mentioned above, we have now performed tissue staining to confirm the decrease in fibroblasts (Figure S6B) and M_a macrophages (Figure S7A) in aged mice compared to young mice.

Fibroblasts were stained using a well-established pan-fibroblast marker PDGFRα (as shown by scRNA-seq data in Figure 6A). Their proportional decrease with age is highly consistent between scRNA-seq results (Figure 6E) and tissue staining (Figure S6B).

M_a macrophages were stained using a cell-type specific marker CD163, which is highly expressed in M_a macrophages, but not in M_b macrophages or other immune cells (as shown by scRNA-seq data in Figure 7B). Tissue staining showed a decrease of M_a macrophage abundance with age (Figure S7A), similar to scRNA-seq (Figure 7B). However, we noted that the magnitude of decrease appears more striking in the scRNA-seq data than tissue staining, likely due to the advantage of immunohistochemistry staining in detecting rare cell populations. Immunohistochemistry focuses on a single cell marker and is therefore not limited by the other more abundant cell populations in the tissue. In contrast, scRNA-seq analysis is restricted to a few thousand cells per run, and is therefore less sensitive to very rare populations.

5. In figure S2B, it is <u>surprising to see that 30-70% of most cell types, and >80% of the NK, B and T cells in the mammary gland are cycling</u>. <u>Previous study from Raafat et al.(PMID: 22952723) and others showed that the proportion of proliferating cells in aged mammary epithelium decreases when compared to that in 14wk-old mice. This is the opposite of the result shown here.</u> The authors should justify their cell cycle phase inferences by staining with cell cycle markers such as Ki67 or PCNA.

We apologize for any potential confusion caused by Figure S2B. Our results are actually consistent with previous studies. We have now improved Figure S2B and clarified the text (page 4-5) to better explain our data.

The high proportion of cycling epithelial cells in our study is due to the fact that we specifically harvested the mammary tissue from mice in diestrus, which is the phase of peak of mammary proliferation during the estrous cycle. Our results are in fact highly consistent with previous studies showing that epithelial cells at diestrus are ~50% positive for Ki67 or PCNA (Fata et al., 2001, PMID 11514328; Joshi et al., 2010, PMID 20445538).

The high proportion of cycling stromal cells may also be explained by a similar reason. However, we acknowledge that the default cell cycle scoring algorithm we used in the standard Seurat analysis may be suboptimal for stromal cell analysis, as it was originally developed based on epithelial cells (Tirosh et al. 2016, PMID 27124452). As a result, we have decided to remove the stromal cell cycle analysis and instead focus on the epithelial populations in the revised manuscript.

Our results are also consistent with previous studies such as Raafat et al., 2012, PMID 22952723, in showing a decrease of epithelial proliferation with age. The extent of decrease in our data is only moderate, again likely due to the effect of diestrus.

The main point we would like to make with this cell cycle analysis was to provide some explanation for the altered cell proportions with age. The originally text in our manuscript stated that: "Seurat proliferation inference analysis using gene expression signatures of cell-cycle S/G2/M phases revealed that different cell types exhibited varying degrees of proliferation, which may in part explain the accumulation of the more proliferative cell types with age (Figure S2B)." By that, we did not

mean to imply that mammary epithelium become more proliferative with age. Instead, we meant to point out that one of the reasons for altered cell proportion over time could be the differences in cell proliferation between different cell types in young mice. In other words, the higher proliferation of a certain cell population (e.g. young myoepithelial and AV cells) compared to a less proliferative population (e.g. young HS and HS-AV cells) could potentially explain, in part, the accumulation of the former cell types over time, resulting in a higher relative proportion in older mice. The observation of higher baseline proliferation in myoepithelial cells and AV cells (ER- luminal cells) compared to HS cells (ER+ luminal cells) is consistent with previous Ki67 staining results in mice and human (e.g. Shehata et al., 2014, PMID 25056669, Figure 4D; Giraddi et al., 2015, PMID 26511661, Figure 2A; Clarke et al., 1997, PMID 9371488, Table 1.). We have now revised the text to make this point more explicitly (page 4-5).

In addition, we have clarified the text to acknowledge that other factors may account for the age-dependent changes in cell type proportions as well, including cell death, migration, and differentiation. Many of these additional factors are beyond the detection ability of scRNA-seq technology.

6. The pair-wise differential expression analyses rely on expression level fold-change with a minimum fold-change cutoff set at 1.25. This seems like a very small difference upon which to make significant inferences. It is necessary to match all samples for sequence depth before combining and doing differential expression analyses. It was not mentioned in the methods whether the authors did so. Considering the small number of DEGs being found, this is critical for between age group comparisons that were done for many cell clusters. New comparisons should be done on sequence depth-matched datasets.

We agree with the reviewer that it is important to ensure even sequencing depth across scRNA-seq samples. As mentioned above, the sequencing depth in our samples are fairly consistent, and is therefore not likely a cause for concern. We have now included this information to the Methods section (page 19, first paragraph):
“Sequencing saturation as determined by Cell Ranger showed fairly even saturation across samples (young mice: 82.3%, 79.9%, 70.6%; aged mice: 91.3%, 78.6%, 73.3%, 85.1%).”

Regarding fold-change cutoff, our threshold is actually set at 1.5 for pairwise comparison between young mice and aged mice. We only relaxed the threshold to 1.25 for the per-sample comparison to allow for minor sample-to-sample variation. However, the differentially expressed genes still needs to satisfy the >1.5 fold change in the grouped comparison in order to be deemed significant in our analysis. This information is detailed in the Methods section (page 19).

Minor:

1. Pal et al identified a minor basal population that they claimed could be a bipotent progenitor as it appeared to have cells expressing both basal and luminal markers. Other groups did not see this cluster or claimed it could be the result of "doublets." Was such a population observed here? Evidence for or against existence of such a population should be mentioned to help clear up the contentious literature on this topic.

Indeed, consistent with other groups (e.g. Bach et al., Kanaya et al.), our scRNA-seq study did not detect a population co-expressing basal and luminal markers in healthy mice at both young and old

age. It is possible that the minor population observed by Pal et al. represented doublets, but we do not have enough evidence to make that criticism about their study.

In the context of aging, we note that such basal-like luminal cells have been reported in aged mice with mammary hyperplasia and in older women (Dong et al., 2016; Garbe et al., 2012; Pelissier Vatter et al., 2018), suggesting that these cells might represent a more aberrant population that are not usually present in normal murine mammary tissues. Please see this point stated in the Discussion section (page 12).

2. Gene labels in the heatmap are difficult to read. Higher resolution image should be used.

We apologize that the figure file size needed to be reduced significantly to satisfy initial submission requirement. We are happy to provide higher-resolution heatmaps with legible gene names in a final version of the manuscript. In addition, all the gene names associated with the heatmaps are listed in Tables S1 and S3, in the same order as they appear in the heatmaps.

3. Figure S4B should include a quantitative summary of secreted content over a number of ducts+alveoli of the same size.

We thank the reviewers for this suggestion. The quantification has now been added (Figure S4B). While luminal secretory material is known to be present at low frequency in young virgins, we found that aged mammary glands have a higher frequency of secretion-filled alveoli as well as filled dilated ducts. These results are consistent with the lactation-primed gene expression state in aged AV cells as detected by scRNA-seq.

4. In previous scRNAseq profiling of young virgin adults, Tph1 and Arg1 are both expressed at abundant levels in the WT ML population (dataset from Pal et. al, Dong et al GSE136148, Giraddi et al.). Could this observation of upregulation be due to sequencing depth differences? Again, having a Figure or Table that enables the reader to easily determine how the clusters identified here transcriptomically align with previous ones identified using flow sorting would be very helpful, especially as this paper is submitted as a RESOURCE.

We thank the reviewer for making this point. We would like to clarify that both *Tph1* and *Arg1* are indeed detectable in young HS (ML) cells in our scRNA-seq data. However, their expression levels are dwarfed in scale when compared to the much higher expression levels in aged HS cells. (Please see TSNE plots and violin plots in Figure 4I).

In addition, this increased expression of *Tph1* and *Arg1* in aged HS cells compared to young cells echoes with the observation by Bach et al. that their expression increases in HS cells at pregnancy or post-involution compared to virgin mice. Thus, these expression patterns are consistent with the lactation-primed state of aged mammary glands.

In terms of sequencing depth, as mentioned above, the sequencing depth is comparable across samples and is unlikely to be a cause of concern.

We agree that it is helpful to create a table to summarize how the luminal cell population clusters identified in this study align with other studies under a different nomenclature. As mentioned above, we have added the summary in Figure S5A.

5. Is it possible that the HS population is comprised of 2 or more distinct populations? This is strongly indicated by Arg1 expression levels.

The Arg1-low and Arg1-high cells in the TSNE map in Figure 4I represent young and aged HS cells, respectively. We believe that the clustering pattern reflects age-associated difference in HS cells (please see Figure S1A), rather than two distinct HS subpopulations within the same mouse.

6. The finding that HS cells increase expression of Arg1 over time has exciting implications in breast tumorigenesis. The authors should (at the very least) stain for Arg1 to confirm protein expression. While this may be beyond the scope of this study, it may be of interest for the authors to test if old vs young HS cells differentially impacts T cell function. This could be determined by in vitro assays.

We have now added immunofluorescence staining for ARG1 to confirm its increased expression at the protein level in aged HS cells compared to young cells (Figure S4C).

We appreciate the reviewer's enthusiasm about the implication for breast tumorigenesis and T cell function. However, we agree that functional experiments on T cells indeed would go beyond the scope of this study. Such experiment would be more suitable for a different study in the future.

7. While it is helpful to see individual variation in cell proportions in figure 2, it is difficult to follow conclusions of fold-changes for each population (as stated in the text). Please also include a 100% stacked column graph with mean and error for each population.

We have now added a stacked column graph showing the mean and error bars for each population in Figure 2.

Manuscript Click here to view linked References

Aging-Associated Alterations in Mammary Epithelia and Stroma Revealed by Single-Cell RNA Sequencing

Carman Man-Chung Li[1], Hana Shapiro[1], Christina Tsiobikas[1], Laura M. Selfors[1], Huidong Chen[2,3], Jennifer Rosenbluth[1], Kaitlin Moore[1], Kushali P. Gupta[1], G. Kenneth Gray[1], Yaara Oren[1,2], Michael J. Steinbaugh[4], Jennifer L. Guerriero[5,6], Luca Pinello[2,3], Aviv Regev[2,7], Joan S. Brugge[1,8, *]

[1] Department of Cell Biology, Harvard Medical School, Boston, MA 02115, USA
[2] Broad Institute of MIT and Harvard, Cambridge, MA 02142, USA
[3] Molecular Pathology Unit & Cancer Center, Massachusetts General Hospital Research Institute and Harvard Medical School, Charlestown, MA 02129, USA
[4] Department of Biostatistics, Harvard T.H. Chan School of Public Health, Boston, MA 02115, USA
[5] Breast Tumor Immunology Laboratory, Dana-Farber Cancer Institute, Boston, MA 02115, USA
[6] Division of Breast Surgery, Department of Surgery, Brigham and Women's Hospital, Boston, MA 02115, USA
[7] Current address: Genentech, 1 DNA Way, South San Francisco, CA 94080, USA
[8] Lead Contact
*Correspondence: Joan_Brugge@hms.harvard.edu

SUMMARY

Aging is closely associated with increased susceptibility to breast cancer, yet there have been limited systematic studies of aging-induced alterations in the mammary gland. Here we leverage high-throughput single-cell RNA-sequencing to generate a detailed transcriptomic atlas of young and aged murine mammary tissues. By analyzing epithelial, stromal, and immune cells, we identify age-dependent alterations in cell proportions and gene expression, providing evidence that suggests alveolar maturation and physiological decline. The analysis also uncovers potential pro-tumorigenic mechanisms coupled to the age-associated loss of tumor suppressor function and change in microenvironment. In addition, we identify a rare, age-dependent luminal population co-expressing hormone-sensing and secretory-alveolar lineage markers, as well as two macrophage populations expressing distinct gene signatures, underscoring the complex heterogeneity of the mammary epithelia and stroma. Collectively, this rich single-cell atlas reveals the effects of aging on mammary physiology and can serve as a useful resource for understanding aging-associated cancer risk.

KEYWORDS

aging, mammary epithelia and stroma, luminal and myoepithelial cells, fibroblasts, endothelial cells, macrophages, single-cell RNA-seq

INTRODUCTION

The mammary gland undergoes dynamic changes in cellular composition and gene expression over a lifetime (Inman et al., 2015). Although recent single-cell RNA-sequencing (scRNA-seq) studies have characterized changes during embryonic development, puberty, and pregnancy (Bach et al., 2017; Giraddi et al., 2018; Pal et al., 2017; Wuidart et al., 2018), little is known about the alterations associated with aging, an important aspect of mammary development closely related to breast cancer (Jenkins et al., 2014). While mammography, histology, and molecular analyses have revealed prominent changes with age, including decreased mammographic density, altered epithelial proportions, and reduced connective tissue (Azam et al., 2019; Dong et al., 2016; Garbe et al., 2012; Gertig et al., 1999; Hart et al., 1989; Hutson et al., 1985; McCormack et al., 2010; Pelissier Vatter et al., 2018; Pelissier et al., 2014), systematic single-cell transcriptome profiling can better capture age-associated effects at higher resolution and on a larger scale. Moreover, given that increased age is strongly associated with breast cancer susceptibility in human and mouse models (Jenkins et al., 2014; LaBarge et al., 2016; Raafat et al., 2012), a single-cell atlas for aged mammary glands could help fill the gap in our knowledge of aging and cancer risk. The use of human samples to study aging has been challenging due to confounding covariates such as pregnancy history (parity) and accumulated environmental stress (Ginger and Rosen, 2003; Huh et al., 2015; Kamikawa et al., 2009; Russo et al., 2008; Santos et al., 2015; Singletary and McNary, 1992). In contrast, mouse models can complement human studies by providing more precise control of parity, hormone cycle, genetic background, and environmental factors, thus enabling a focus on age-specific effects.

Mammary gland function depends on both the epithelial and stromal compartments (Inman et al., 2015; Polyak and Kalluri, 2010). The mammary epithelial bilayer contains outer myoepithelial/basal cells and inner luminal cells, which collectively form branching ducts and terminal acinus. Myoepithelial cells are distinguished by their contractility and ability to synthesize basement membrane. Luminal cells, on the other hand, consist of two subtypes: hormone-sensing (HS) cells capable of responding to endocrine signals such as estrogen, progesterone, and prolactin, as well as secretory alveolar (AV) cells capable of producing milk. HS and AV cells have also been referred to as mature luminal (ML) and luminal progenitor (LP) cells, respectively, based on their *in vitro* colony-forming ability (Fu et al., 2020; Shackleton et al., 2006; Stingl et al., 2006); however, cumulative evidence suggests that these two lineages are independent in adulthood (Lilja et al., 2018; Van Keymeulen et al., 2017; C. Wang et al., 2017). Surrounding the epithelial cells are various stromal cells, including fibroblasts, vascular/lymphatic cells, immune cells, and adipocytes. While initial scRNA-seq studies provided valuable knowledge of mammary epithelia in early and adult development (Bach et al., 2017; Giraddi et al., 2018; Nguyen et al., 2018; Pal et al., 2017; Wuidart et al., 2018), characterization of stromal cells has been limited (Han et al., 2018; Kanaya et al., 2019; Tabula Muris Consortium, 2018; 2020).

Here, we used scRNA-seq to analyze young and aged murine virgin mammary glands, identifying a significant diversity of epithelial and stromal cell. We captured a rare population of luminal epithelial cells co-expressing HS and AV lineage markers but distinguishable by a unique gene signature. We also detected heterogeneous stromal/immune populations, including distinct macrophage subtypes. In addition, comparison of young and aged cells identified aging-associated changes in cell-type compositions and transcriptomic profiles, providing evidence that suggests alveolar maturation and physiological decline. The analysis also identified potentially pro-tumorigenic alterations that may provide initial insight into mechanisms for age-associated vulnerability to breast cancer. Overall, these observations are highly consistent across multiple samples, suggesting that the intrinsic effects of aging on mammary tissues may

follow a defined biological program, and are not merely consequences of stochastic deterioration.

RESULTS

Diverse Epithelial and Stromal Cell Types Identified by scRNA-seq in Mammary Glands

To study aging in mammary tissues, we performed scRNA-seq analysis using the 10X Chromium platform on young (3-4 months old, n = 3) and aged (13-14 months old, n = 4) virgin mice (Figure 1A), which correspond to human early adulthood (20-30 years of age) and perimenopause (45-55 years of age), respectively (Diaz Brinton, 2012; Finch et al., 1984; Flurkey et al., 2007). Mice were analyzed at diestrus, when progesterone levels and epithelial cell proliferation peak, to facilitate detection of progenitor cells (Brisken and Ataca, 2015; Fata et al., 2001; Joshi et al., 2010). With stringent filtering at cell and gene levels to eliminate potential doublets, we captured a total of 13,684 cells and 27,998 genes. Single cells were clustered based on gene expression profiles using Seurat and visualized using t-distributed stochastic neighbor embedding (t-SNE)(Butler et al., 2018; Stuart et al., 2019) (Figures 1B and S1A). Doublet estimation using Scrublet (Wolock and Klein, 2019) and DoubletFinder (McGinnis et al., 2019) demonstrated that doublets likely accounted for a negligible fraction of our data and did not drive clustering (Figure S1B).

Epithelial and stromal cell types were identified using canonical markers (Figures 1B-1F). As expected, we detected epithelial cells (n = 8,901; expressing *Epcam*) of myoepithelial lineage (n = 1,670; expressing *Krt17*, *Krt14*, *Krt5*, *Acta2*, *Myl9*, *Mylk*, and *Myh11*) or luminal lineage (n = 7,231; expressing *Krt19*, *Krt18*, and *Krt8*) (Figure 1E). Luminal cells consisted of both HS cells (n = 2,138; expressing *Prlr*, *Pgr*, *Esr1*, *Cited1*, and *Prom1*) and AV cells (n = 4,820; expressing *Mfge8*, *Trf*, *Csn3*, *Wfdc18*, *Ltf*, and *Elf5*). AV cells expressed luminal progenitor markers (*Kit*, *Aldh1a3*, and *Cd14*) and milk biosynthesis-related genes (*Mfge8*, *Trf*, *Csn3*, *Wfdc18*, and *Ltf*), but not mature alveolar markers associated with pregnancy and lactation (*Wap*, *Glycam1*, and *Olah*) (Figure 1E), consistent with their alveolar progenitor differentiation state in virgin glands (Booth et al., 2007). In addition, we identified a rare luminal population co-expressing hormone-sensing markers and alveolar progenitor markers (n = 273; Figures 1B, 1C, and 1E). We termed these HS-AV cells. This population is unlikely to be doublets based on doublet analyses above (Figure S1B).

Among stromal cells (n = 4,783), we detected fibroblasts (n = 1,993; expressing *Col1a1*, *Col1a2*, *Col3a1*, and *Fn1*), vascular/lymphatic cells (n = 2,196; expressing *Pecam1*, *Cdh5*, and *Eng*), and immune cells (n = 594; expressing *Ptprc*, which encodes CD45) (Figure 1F). The vascular/lymphatic population consisted of vascular endothelial cells (n = 1,819; expressing *Sox17* and *Sele*), pericytes (n = 349; expressing *Rgs5*, *Des*, and *Notch3*), and lymphatic endothelial cells (n = 28; expressing *Mmrn1*, *Prox1*, *Flt4*, and *Ccl21a*). The immune population contained both myeloid cells (marked by Cd74 and Lyz2) and lymphocytes (marked by *Cd3d*, *Cd3e*, and *Cd3g*). Within myeloid cells, we detected dendritic cells (n = 226; expressing *Napsa*, *Traf1*, *Cd209a*, and *Flt3*) and two populations of macrophages (n = 268; both expressing *Csf1r*, *Fcgr3*, *Adgre1*, and *Ms4a7*, but distinguishable by *Mrc1*, *Cd209f*, and *Cd163* (designated as M_a hereafter) or by *Mmp12, Mmp13*, and *Spic* (designated as M_b)). Within lymphocytes, we detected natural killer cells (NK cells; n = 40; expressing *Gzma*, *Ncr1* and *Itgae*) as well as T and B cells (n = 60), with the vast majority being CD8 T cells (marked by *Cd8a* and *Cd8b1*) and a very small fraction being CD4 T cells (marked by *Cd4*) and B cells (marked by *Cd79a* and *Cd79b*). These immune cells represented mammary tissue specific cells, as we discarded the

lymph nodes in sample preparation. The only major stromal population not captured in our analysis was adipocytes, which were removed with the supernatant during tissue dissociation.

We generated gene signatures characterizing each cell type by multiple pair-wise differential gene expression analyses (see Methods). The resulting cell type-specific signatures (Figure 1G and Table S1) can serve as a useful resource for identifying or isolating cell types within heterogeneous mammary populations. Importantly, the ability to capture relatively rare populations such as lymphatic endothelial cells and different immune cells, as well as the ability to detect lesser-known populations such as HS-AV cells and distinctive macrophages, highlight the power of scRNA-seq to identify diverse cell types in the mammary gland without *a priori* knowledge required to pre-isolate these populations.

Prevalent Alterations in Cell Proportions and Gene Expression in Aged Mammary Glands

ScRNA-seq analysis revealed differences between young and aged mammary glands in both cell type composition and gene expression. Cell type composition inferred from the transcriptomic data is consistent among biological replicates despite sample-to-sample variation, and differs significantly across age groups (Figures 2A-2D, S1A, S2A, and Table S2). The relative proportion of epithelial cells to stromal cells increased considerably with age, as epithelial cells increased from 45% of total cells to 82%, whereas stromal cells decreased commensurately from 55% to 18% (Figure 2A; $p < 0.0001$, Fisher's exact test). Within the epithelial and stromal compartments, cell type composition also changed with age. Among epithelial cells (Figure 2B), AV luminal cells expanded by 3-fold ($p < 0.0001$, Fisher's exact test) whereas HS luminal cells diminished by 6-fold ($p < 0.0001$, Fisher's exact test). Among stromal cells (Figure 2C), the relative abundance of fibroblasts fell by 3-fold with aged ($p < 0.0001$, Fisher's exact test), whereas vascular endothelial cells rose by 2-fold ($p < 0.0001$, Fisher's exact test). Furthermore, while the proportion of total immune cells within the stromal compartment remained relatively unaltered, the composition of immune cell types changed with age (Figure 2D). The proportion of dendritic cells decreased by 2-fold ($p < 0.0004$, Fisher's exact test). M_a macrophages decreased by 40-fold ($p < 0.0001$, Fisher's exact test) whereas M_b macrophages remained relatively constant, thus rendering M_b the dominant macrophage population in aged tissues. In contrast to the reduction in myeloid cells, the proportion of lymphocytes increased with age. Specifically, NK cell percentage increased by 2-fold ($p < 0.03$, Fisher's exact test), whereas CD8 T cells increased by 8-fold ($p < 0.0001$, Fisher's exact test). Gene signature-based cell-cycle inference analysis of epithelial populations revealed varying degrees of proliferation that are consistent with the high proliferation rate expected at diestrus due to progesterone stimulation (Figure S2B) (Fata et al., 2001; Joshi et al., 2010). The proportion of proliferating epithelial cells decreased with age, in accordance with previous observations (Raafat et al., 2012); the decrease was only moderate, again, likely due to the effect of diestrus. Notably, the higher proliferation of certain cell types (myoepithelial and AV cells) compared to others (HS and HS-AV) is concordant with previous studies (Clarke et al., 1997; Giraddi et al., 2015), and may partially explain the accumulation of myoepithelial and AV cells relative to HS and HS-AV cells at older age (Figure 2B). Besides proliferation, other factors such as cell death, migration, and differentiation may also account for the age-dependent changes in cell type proportions.

In addition to composition, cell type-specific gene expression differences were also detected in young and aged mammary glands (Figures S3A-S3L). Within the epithelial compartment, myoepithelial cells exhibited the largest number of differentially expressed genes (Figure S3A-S3D), whereas within the stromal compartment, vascular endothelial cells showed the most striking pattern (Figure S3E-S3H). Moreover, M_b macrophage gene expression was affected to

a greater extent compared to other immune cells (Figure S3I-S3L). These age-dependent changes are examined in greater detail below. Analysis of number of genes detected per cell indicated that these age-associated gene expression patterns were not simply driven by sample variation in total genes captured by scRNA-seq (Figure S3M). Overall, our results demonstrate that both cellular proportion and gene expression are profoundly affected by aging.

Aged Myoepithelial Cells Show Altered Gene Expression

Myoepithelial cells, distinguished by basal keratins *Krt17*, *Krt14*, and *Krt5* (Figure 3A), only increased slightly in abundance with age (Figure 3B); however, they exhibited a striking degree of gene expression changes (Figure 3C and Table S3). Differential expression analysis detected 111 genes upregulated and 106 genes downregulated in aged myoepithelial cells. Pathway analysis indicated that these genes primarily fell into four functional categories – cytokines/growth factors, oxidative phosphorylation, extracellular matrix (ECM), and cytoskeleton/contractility (Figure 3D-3H and Table S4). More specifically, aged myoepithelial cells increased expression of multiple cytokines that are known to regulate the immune system (including *Cxcl1*, *Cxcl2*, *Cxcl16*, *Csf1*, and *Csf3*), as well as several growth factors (*Tgfb1*, *Jag1*, and *Vegfa*) (Figure 3E and Table S3). Aged myoepithelial cells also showed lower expression of oxidative phosphorylation genes (*Ndufa3*, *a5*, *a7*, *a8*, *a13*, *b3*, *b9*, *b10, c1*, *v3*, *Atp5j*, *Etfb*, *Uqcr10*, and *Uqcr11*) (Figure 3F and Table S3), which may reflect reduced metabolic activity. Expression of several ECM-related genes decreased with age (*Dcn*, *Col4a1*, *Col4a2*, *Serpinh1*, *Sparc*, *Emid1*, *Dag*, and *Spon2*) (Figures 3G and Table S3), indicating a potential impairment in the function to synthesize basement membrane. Expression is also reduced in multiple actomyosin-related genes (*Acta2*, *Actg2*, *Mylk*, *Myl6*, *Myl9*, and *Myh11*) as well as the cytokeratin intermediate filament *Krt15* (Figures 3H and Table S3), indicating a potential decline in the ability to maintain contractility.

Of note, because *Krt15* and *Acta2* (also known as smooth muscle actin, SMA) have been reported as myoepithelial markers in murine mammary glands (Bach et al., 2017; Pal et al., 2017), their reduced expression with age would suggest that they may not be reliable markers for older myoepithelial cells. We therefore performed immunofluorescence staining to confirm their protein expression pattern in young and aged mammary glands, using KRT14 as a reference myoepithelial marker as its expression remained stable as detected by scRNA-seq. Consistent with scRNA-seq data, immunostaining demonstrated that KRT15 and ACTA2/SMA were expressed in young myoepithelial cells, but their level significantly decreased in aged glands (Figures 3I-3L). Therefore, our findings suggest that *Krt15* and *Acta2*, as well as other cytoskeletal/contractility genes (e.g. *Mylk*, *Myl6*, *Myl9*, and *Myh11*), should be used as myoepithelial markers with caution depending on age.

Alterations in Luminal HS and AV Cells with Age

We next examined age-related changes in luminal HS cells and AV cells. AV cells were identified by their characteristic milk-related genes *Mfge8*, *Csn3*, *Trf*, *Wfdc18*, and *Ltf* (Figure 4A), whereas HS cells were identified by hormone-sensing markers *Prlr*, *Pgr*, *Esr1*, and canonical HS lineage markers *Cited1* and *Prom1* (Figure 4B). The proportion of AV cells expanded with age from 26% to 69% within the epithelial compartment, and HS cells diminished from 53% to 9% (Figures 4C and 4D). FACS analysis of an independent cohort of mice confirmed the age-dependent increase of AV cells (Lin-, Epcam+, CD133-, CD14+/-; from 62% to 77% of the luminal compartment) and decrease of HS cells (Lin-, Epcam+, CD133+, CD14-; from 37% to 22% of the luminal compartment) (Figure 4E). The difference in exact percentages

detected by scRNA-seq and FACS is likely due to technical differences as well as post-transcriptional and/or translational regulations.

Differential expression analysis of young vs. aged AV cells revealed 24 upregulated genes and 24 downregulated genes (Figure 4F and Table S3), including increased expression of several milk-biosynthesis regulators that are exclusively detected in AV cells but not HS cells. These genes include pyruvate dehydrogenase kinase *Pdk4* (Kankel and Reinauer, 1976), milk lipid droplet coating protein *Plin2* (Russell et al., 2011), and milk bioactive protein *Cd59a* (Bjørge et al., 1996) (Figure 4G). This expression pattern led us to examine other milk-related genes, many of which were also upregulated with age in AV cells in at least three out of four replicates, including lactose synthase *Lalba*, caseins *Csn1s1*, *Csn1s2a*, *Csn2*, and milk osteopontin *Spp1* (Figure S4A). The increased expression of milk biosynthesis genes may potentially reflect a shift toward a more mature alveolar state. This is consistent with previous reports (Raafat et al., 2012) and our observation that aged mammary glands showed signs of activated alveolar cells independent of pregnancy, including increased secretory material in acini and dilated ducts (Figure S4B).

On the other hand, aged HS cells showed upregulation of 18 genes and downregulation of 12 genes (Figure 4H and Table S3). Many of these are regulated by hormone receptors, consistent with their expression in HS cells and potentially reflecting hormonal changes associated with aging (Ace and Okulicz, 1998; Gannon et al., 2010; Gundlah et al., 2005; Hickey et al., 2013; Ono et al., 2014; Sampath et al., 2002; Tsai et al., 2002; Tynan et al., 2005). Two upregulated genes, *Tph1* and *Arg1*, were particularly noteworthy due to their exclusive expression in HS cells and no other cell types (Figure 4I). Expression of *Tph1* (tryptophan hydroxylase 1) in the mammary gland is specifically activated during lactation for serotonin biosynthesis (Laporta et al., 2014; Matsuda et al., 2004). Its increased level in aged HS cells is consistent with the lactation-primed state of aged mammary glands discussed above. *Arg1* (arginase 1) inhibits proliferation and function of T cells and NK cells by depleting L-arginine in the microenvironment (Bronte and Zanovello, 2005; Gantt et al., 2014). *Arg1* is commonly expressed by immune inhibitory cells, such as tumor-associated macrophages and myeloid derived suppressor cells, as well as by a diversity of tumor cells, including breast cancer. We confirmed ARG1 protein expression in HS cells as well as its increase with age by performing tissue staining in an independent cohort of mice (Figure S4C). Increased *Arg1* expression in aged HS cells is consistent with an immunosuppressive microenvironment, as discussed later.

Luminal HS-AV Cells Showed Age-Dependent Abundance

Our scRNA-seq analysis also revealed a rare population of HS-AV cells that co-expressed HS and AV markers (Figures 1E, 4A, and 4B), accounting for an average of 11% of epithelial cells in young mammary glands (Figure 5A). Similar cells expressing HS and AV gene signatures have also been identified in young mice by Pal et al. and Bach et al. at comparable proportions (Bach et al., 2017; Pal et al., 2017) and in ovariectomized mice by Kanaya et al. at higher abundance (Kanaya et al., 2019) (Figures S5A and S5B), but they have not been further characterized. We performed differential gene expression analysis to identify markers distinguishing HS-AV cells from HS cells and AV cells, limiting the analysis to young tissues in order to focus on cell type-specific difference independent of aging. The results revealed that HS-AV cells are distinguished by higher expression of 72 genes and lower expression of 9 genes (Figures 5B, 5C, and Table S5). Many of the upregulated markers fell into four functional categories, highlighting the potential implications for the biological roles of HS-AV cells (Figure S5C). (1) Regulators of mammary development, lobulo-alveologenesis, and lactation, such as *Spry2*, *B4galt1*, *Slc39a1*, *Skil*, *Agpat1*, and *Neat1*, suggesting that HS-AV cells might play a role

in mammary gland morphogenesis and milk production (Agarwal et al., 2017; Crisà et al., 2016; Jahchan et al., 2012; 2010; Littlejohn et al., 2010; Lv et al., 2015; McCormick et al., 2014; Sigurdsson et al., 2013; Standaert et al., 2014; X. Zhang et al., 2014; Y. Zhang et al., 2018; Zhu and Nelson, 2013). (2) Chromatin modifying enzymes, including *Kmt2d/Mll2*, *Whsc1l1/Nsd3*, *Gatad1/Odag*, *Kdm6b/Jmjd3*, *Cbx3/Hp1*γ, and *Supt16/Fact140*, which have been implicated in promoting stem cell phenotypes and regulating differentiation (Burchfield et al., 2015; Denissov et al., 2014; Froimchuk et al., 2017; Lomniczi et al., 2015; Mas et al., 2018). (3) Transcription factors including *Nfia*, *Tmf1*, *Bhlhe41*, *Arglu1*, *Btg1*, and *Sox9*. In particular, *Sox9* is known to regulate luminal progenitor cell fate (Edwards et al., 2014; Guo et al., 2012; C. Wang et al., 2017). (4) mRNA processing and modification enzymes, namely *Srrm2*, *Wtap*, *Alkbh5*, *Rbm47*, *Hnrnpul1*, and *Pan3*, which regulate mRNA splicing, m^6A methylation, mRNA processing, and degradation (Blencowe et al., 1998; Y.-E. Kim et al., 2019; Kzhyshkowska et al., 2003; Wahle and Winkler, 2013; Yue et al., 2018; Zheng et al., 2013). Overall, these gene signatures suggest a potential role for HS-AV cells in differentiation plasticity.

Next, we examined the lineage relationship of HS-AV cells to HS cells and AV cells by performing STREAM lineage trajectory analysis (Chen et al., 2019) on scRNA-seq data of young mice. HS-AV cells primarily localized to the bifurcation junction between HS cells and AV cells (Figures 5D and S5D). This pattern was highly reproducible when the analysis was repeated within each of the three young samples (Figure S5D). The predicted lineage trajectory is consistent with the gene expression pattern of HS-AV cells being a hybrid state between the HS and AV lineages, and suggests the possibility that HS-AV cells might have the potential to differentiate into HS cells and AV cells.

Since HS-AV cells have not been reported beyond scRNA-seq studies, we further verified their existence using two orthogonal approaches. First, we performed *in situ* immunofluorescence staining in a panel of young mammary glands, using established hormone-sensing markers – Progesterone receptor (PR) and Estrogen receptor (ER), and alveolar markers – Lactotransferrin (LTF) and Milk fat globule-EGF factor 8 (MFGE8). This analysis detected presence of HS-AV cells co-expressing PR/ER and LTF/MFGE8, scattered within both ducts and acini (Figures 5E-5F). Quantification showed that PR+/MFGE8+ cells and ER+/LTF+ cells represented 0.72% and 0.16% of luminal cells, respectively (Figures 5G-5J). The lower abundance of ER+/LTF+ cells compared to PR+/MFGE8+ cells by immunostaining is consistent with the lower abundance of LTF+ cells compared to MFGE8+ cells within the HS-AV population by scRNA-seq (Figure 4A). As further validation, we analyzed an independent set of young mice by FACS using HS marker CD133 and AV marker CD14. Again, HS-AV cells can be detected as CD133+/CD14+ cells within the luminal population (Lin-, Epcam^{hi}) in all young tissues examined, with an average abundance of 0.25% (Figure 5K). Similar CD133+/CD14+ HS-AV cells were also detected in murine mammary organoid cultures (Figure S5E). Importantly, we confirmed that these cells were not doublet artifacts formed by CD133+ cells and CD14+ cells, as microscopy examination of FACS-isolated CD133+/CD14+ cells demonstrated that they were indeed singlets (Figure S5F). Of note, while HS-AV cells can be detected using multiple approaches, they were lower in abundance when analyzed at the protein level by immunofluorescence and FACS (Figures 5H, 5J, and 5K) than at the RNA level by scRNA-seq (Figure 5A). The limited number of protein markers used in immunofluorescence and FACS analyses likely only captured a subset of the HS-AV population identified transcriptomically. In addition, the difference may reflect gene expression regulation at the post-transcriptional and/or translational levels.

Interestingly, the abundance of HS-AV cells diminished dramatically with age. ScRNA-seq showed that HS-AV cells accounted for an average of 11% of epithelial cells in young mice but

only 0.3% in aged mice (Figure 5A). We confirmed this age-dependent decrease of HS-AV cells by immunofluorescence staining of PR+/MFGE8+ cells and ER+/LTF+ cells in tissues (Figures 5G-5J). In addition, FACS analysis of an independent cohort also demonstrated that aged mice consistently harbored a lower proportion of HS-AV cells than young mice (Figure 5K). Collectively, these results established an age-dependent existence of the HS-AV population.

Age-Dependent Alterations in Stromal Fibroblasts and Vascular/Lymphatic Cells

In addition to mammary epithelial cells, we analyzed age-associated changes in stromal cells (Figures 6A-6D). The most abundant stromal cell type in young tissues was fibroblasts (Figures 6E-6H). These cells homogeneously expressed fibroblast markers *Pdgfra*, *Pdgfrb*, and *Fap* (Figure 6A). They also expressed high level of ECM genes (e.g. *Fn1*, *Col1a1*, *Col1a2*, *Col3a1*) but not contractility genes (e.g. *Acta2*, *Myl9*, *Mylk*, *Myh11*) (Figure S6A), indicating that they were ECM-producing fibroblasts instead of contractile myofibroblasts. The relative proportion of fibroblasts decreased with age (Figure 6E), a pattern corroborated by immunohistochemistry (IHC) staining using fibroblast marker PDGFRα (Figure S6B). In addition, scRNA-seq differential expression analysis revealed 11 genes upregulated and 37 genes downregulated with age (Figure 6I and Table S3), including increased expression of stress-related genes (*Hspa1a*, *Sqstm1*, *Ubc*, *Cebpb*, and *Gadd45b*) and decreased expression of ECM-related genes (*Col5a3*, *Col6a3*, *Fn1*, and *Mmp23*) (Figures 6J, 6K and Table S4). Notably, fibronectin, encoded by *Fn1*, is a major ECM component and a master organizer of matrix assembly (Halper and Kjaer, 2014; Pankov and Yamada, 2002; Theocharis et al., 2016). The decrease in both fibroblast proportion and ECM gene expression is consistent with previous reports in human mammary glands that aging is associated with a reduction of connective tissues (Gertig et al., 1999; Hutson et al., 1985).

Other stromal populations captured by scRNA-seq included vascular/lymphatic cells, namely vascular endothelial cells (expressing *Pecam1*, *Chd5*, and *Sox17*), pericytes (expressing *Des*, *Rgs5*, and *Notch3*), and lymphatic endothelial cells (expressing *Mmrn1*, *Prox1*, and *Flt4*) (Figures 6B-6D). Among these populations, vascular endothelial cells exhibited the most dramatic changes with age, increasing in relative abundance (Figure 6F) and expressing 159 upregulated and 169 downregulated genes (Figure 6L and Table S3). Pathway analysis revealed that many upregulated genes were associated with cytokines that are known to affect the immune microenvironment (*Csf3, Cxcl1*, *Cxcl16*, and *Il6*), whereas many downregulated genes were associated with cell-cell junctions (including adherens junction genes *Ctnnb1*, *Jup*, *Pvrl2*, *Cdh5*, *Mllt4*, and tight junction genes *Cldn5* and *F11r*) (Figures 6M, 6N and Table S4). These changes may reflect altered endothelial-immune interaction and vascular permeability. In comparison, pericytes and lymphatic endothelial cells remained relatively constant with age in terms of proportion (Figures 6G, 6H, and Table S2) and gene expression (Figures S3G and S3H).

Age-Dependent Alterations in Myeloid and Lymphoid Immune Cells

ScRNA-seq also detected remarkable changes in the composition of immune cell types in aged mammary glands. As mentioned above, the proportion of myeloid cells diminished whereas that of lymphoid cells expanded with age. Among myeloid cells, dendritic cells (expressing *Napsa*, *Cd209a*, and *Flt3*) and M_a macrophages (expressing *Cd163, Mrc1*, and *Cd209f*) decreased in relative abundance, leaving M_b macrophages (marked by *Mmp12, Mmp13*, and *Spic*) the major myeloid population in older mammary glands (Figures 7A-C). Among lymphoid cells, the proportion of both NK cells (expressing *Gzma*, *Ncr1* and *Itgae*) and CD8 T cells (expressing *Cd8a* and *Cd8b1*) increased with age (Figures 7D-E). Since M_a macrophages and CD8 T cells represented the most striking proportional changes, we also

analyzed their relative abundance by IHC staining. CD163+ M_a macrophages, which are localized preferentially in stroma, decreased in abundance with age (Figure S7A). In contrast, CD8+ T cells, which were closely associated with the mammary epithelium, increased in abundance with age (Figure S7B). Compared to scRNA-seq, IHC is considerably more sensitive in detecting these rare cell types.

Macrophages play critical roles in mammary gland development and breast cancer (Brady et al., 2016; Gouon-Evans et al., 2002; Guerriero, 2018); however, the heterogeneity of resident macrophages within normal mammary glands remains incompletely understood. To further characterize the two macrophage populations captured by scRNA-seq, we performed differential gene expression analysis and obtained 241 markers that distinguished M_a and M_b macrophages (Figure 7F and Table S6). The results suggest that M_a and M_b macrophages do not show clear association with classical M1/M2 polarization. Instead, comparison of these markers to a recent study on mammary macrophages (Jäppinen et al., 2019) revealed that M_a macrophages are enriched for key markers of fetal-derived macrophages, *Mrc1* (also known as *Cd206*) and *Adgre1* (also known as *F4/80*), whereas M_b macrophages are enriched for adult-derived macrophage markers, MHC II genes *H2-Aa*, *H2-Ab1*, *H2-DMa*, *H2-DMb1*, *H2-Eb1*, and *H2-Oa* (Figures 7F, S7C, and Table S6). Therefore, M_a cells may represent fetal-derived macrophages arising from fetal yolk sac and liver, whereas M_b cells may represent adult-derived macrophages arising from the bone marrow. In addition, two recent studies proposed that mammary macrophages can be defined by their physical locations. Stromal macrophages (characterized as Mrc1/Cd206+, Lyve1^{hi}, Itgax/Cd11c-, Vcam1^{lo}, or as Mrc1/Cd206^{hi}, Lyve1^{hi}, Cd209f^{hi}, Cd209g^{hi}) survey the ECM and largely arise from fetal origin, whereas ductal macrophages (characterized as Mrc1/Cd206-, Lyve1^{lo}, Itgax/Cd11c+, Vcam1^{hi}, or as Mrc1/Cd206^{lo}, Lyve1^{lo}, Cd74^{hi}, H2-Ab1^{hi}) survey the epithelia and largely arise from adult bone marrow after puberty (Dawson et al., 2020; Y. Wang et al., 2020). Notably, these gene signatures are very closely recapitulated by M_a and M_b macrophages, respectively, suggesting that M_a cells may represent stromal macrophages while M_b cells may be ductal macrophages (Figure S7D-E and Table S6). In our analysis, M_b macrophages are also distinguishable from M_a macrophages by higher expression of cell surface antigens *Cd14*, *Cd52*, *Cd63*, *Cd72*, *Cd74*, *Cd207*, cytokines *Il1a*, *Il1b*, *Il10*, *Il12b*, *Cxcl2*, *Cxcl16*, as well as proteases *Mmp12*, *Mmp13*, *Ctss*, and *Ctsz* (Figure 7F). Their increased relative proportion with age is consistent with a replacement of fetal-derived macrophages by adult bone marrow-derived macrophages as the mammary gland ages (Ginhoux and Guilliams, 2016; Hoeffel and Ginhoux, 2018). The recruitment of M_b macrophages may be mediated by the increased expression of *Csf1* and *Csf3* in aged myoepithelial cells and vascular endothelial cells (Figures 3E and 6N).

Aging induced a very modest number of gene expression changes in M_b macrophages compared to epithelial and non-immune stromal cell types, albeit more than other immune cells (Figure S3J), with 10 genes upregulated and 4 genes downregulated with age in a consistent manner across all samples (Figure 7G and Table S3). While the number is very modest, it contains a few intriguing genes, including cytokines *Ccl5*, *Cxcl2*, and *Gdf15* (Figure 7H). CCL5 has been shown to recruit T cells (Arango Duque and Descoteaux, 2014); thus, its increased expression in aged M_b macrophages is consistent with the higher abundance of T cells observed in aged mammary glands. Furthermore, expression of two immunosuppressive ligands targeting T cells and NK cells also increased – *Cd274* (also known as *Pd-l1*) (Hsu et al., 2018; Lu et al., 2019; Yamazaki et al., 2002) and *Lilrb4* (also known as *Ilt3*) (Cella et al., 1997; Deng et al., 2018; Suciu-Foca and Cortesini, 2007) (Figure 7I), thereby potentially promoting an immunosuppressive microenvironment. Taken together, these results highlight the diverse and dynamic immune landscape in aging mammary tissues.

DISCUSSION

To better understand the effects of aging on mammary glands, we have created a single-cell transcriptomic atlas to capture murine mammary epithelial and stromal changes associated with

aging, corresponding to the transition from human early adulthood to perimenopause. Considering the longer human lifespan, there may be additional age-related changes not captured by a murine model. However, unlike human studies, mouse analyses can directly assess aging-specific alterations without confounding factors such as pregnancy history, hormone cycle, genetic background, diet, and carcinogen exposure. Importantly, the cell- and gene-level profiles identified herein are consistent across multiple biological replicates, suggesting that the intrinsic effects of aging may follow a defined developmental program instead of stochastic degeneration.

Our scRNA-seq data indicate a potential shift of the luminal populations toward a more lactation-primed state in aging. Cell-type proportion analysis suggests a preferential expansion of AV cells relative to HS cells with age. Additionally, aged AV cells acquire higher expression of genes that are associated with milk production and are known to be upregulated in gestation (Bach et al., 2017). These changes are consistent with previous reports (Raafat et al., 2012) and our histological observation that aged nulliparous mice exhibit lobuloalveolar maturation, likely resulting from accumulated exposure to hormonal stimulation through multiple estrous cycles. Similar observation of age-dependent expansion of lobuloalveolar epithelium was reported in human (Garbe et al., 2012; Hutson et al., 1985; Pelissier Vatter et al., 2018).

Other age-associated alterations detected by scRNA-seq may reflect a decline or dysregulation in cellular functions. For instance, aged myoepithelial cells downregulate genes known to mediate contractility and synthesize basement membrane (Haaksma et al., 2011; Ormerod et al., 1983; Sopel, 2010; Warburton et al., 1982; 1981), in part consistent with previous reports that thinner and discontinuous basement membrane is associated with aging (Frantz et al., 2010; LaBarge et al., 2016). Similarly, aged fibroblasts decrease in relative abundance and ECM gene expression, suggesting an impaired ability to maintain stromal matrix, thus consistent with previous reports of reduced connective tissue and mammographic density in older women (Gertig et al., 1999; Hart et al., 1989; Hutson et al., 1985; Li et al., 2005; McCormack et al., 2010; Unsworth et al., 2014). Additionally, aged endothelial cells downregulate expression of cell-cell junction components, potentially contributing to compromised endothelial barrier and dysregulated vascular permeability (Bazzoni and Dejana, 2004). Vascular hyperpermeability is strongly associated with aging and is partly attributed to inflammatory cytokines such as *Il6* and *Vegfa* (Donato et al., 2015; Oakley and Tharakan, 2014), which we observe to be upregulated in aged vascular endothelial cells and myoepithelial cells, respectively. Finally, aged mammary glands exhibit signs of pro-inflammatory microenvironment, with increased production of inflammatory cytokines (including *Cxcl1*, *Cxcl2*, *Cxcl16*, *Csfs1*, *Csf3*, *Ccl5*, and *Il6*) by aged myoepithelial cells, vascular endothelial cells, and macrophages, and expanded proportions of bone marrow-derived macrophages, NK cells and T cells. These observations also suggest potential inter-cell type crosstalk and recruitment, leading to systemic changes in aged mammary glands.

Given that aging is significantly correlated with breast cancer frequency in mice and human (Jenkins et al., 2014; LaBarge et al., 2016; Raafat et al., 2012), our findings may also provide initial insights into its underlying mechanisms. First, the expansion of AV luminal progenitor cells may pose a higher cancer risk, as they have been proposed to be the cells-of-origin for triple-negative breast cancer (Lim et al., 2009; Molyneux et al., 2010; Proia et al., 2011). Second, aged myoepithelial cells display increased expression of *Jag1* and *Tgfβ*, which can stimulate tumor proliferation and invasion (Cohen et al., 2010; Hu et al., 2008; Muraoka et al., 2003; Muraoka-Cook et al., 2004). Third, the downregulation of genes associated with contractility and basement membrane may impair the ability of myoepithelial cells to restrain malignant luminal cell dissemination (Adriance et al., 2005; Pandey et al., 2010; Sirka et al., 2018). Previous

studies have demonstrated that reduced myoepithelial cell contractility promotes malignant cell invasion (Sirka et al., 2018), whereas compromised basement membrane integrity is associated with breast cancer progression (Gusterson et al., 1982; Nakano et al., 1999; Natali et al., 1984; Tsubura et al., 1988). Loss of basement membrane has also been shown to promote abnormal luminal cell polarity, a hallmark of cancer (Gudjonsson et al., 2002). Moreover, increased vascular endothelial permeability, as suggested by reduced expression of cell-cell junction components, may further potentiate malignant cell dissemination (Maishi and Hida, 2017).

Additionally, aged mammary glands exhibit numerous signs of an inflammatory microenvironment that may promote the proliferation and metastasis of malignant cells (Greten and Grivennikov, 2019; Landskron et al., 2014; S. Zhang et al., 2018). First, the expression of pro-inflammatory cytokines increased in aged myoepithelial cells, vascular endothelial cells, and macrophages. Second, the change in proportions of M_a and M_b macrophages suggests a replacement of fetal yolk sac and liver-derived macrophages by adult bone marrow-derived cells with age. Bone marrow-derived macrophages in murine mammary tumors have been associated with pro-tumorigenic properties by their ability to inhibit CD8 T cells (Franklin et al., 2014). In line with this, three key markers of bone marrow-derived tumor-associated macrophages described by Franklin et al., *Vcam1*, *Itgb5*, and *Itgax*, are also more highly expressed in M_b macrophages, suggesting potential similarities between M_b and adult-derived tumor-associated macrophages. Third, aged M_b macrophages upregulate expression of immunosuppressive ligands *Cd274/Pd-l1* and *Lilrb4/Ilt3*, thus potentially inhibiting NK cells and T cells, creating a pro-tumor immune microenvironment (Lu et al., 2019). Increased inflammation with age was also previously observed in mouse mammary glands and other organs, but the specific source and mediators of inflammation remains incompletely understood (Dong et al., 2016; Sanada et al., 2018). Future studies may focus on the inflammatory cytokines and immune cell types enriched in aged mammary glands as detected herein to further delineate their exact contribution to tumor formation and progression. Of note, basal-like luminal cells have been reported in aged mice with mammary hyperplasia and in older women (Dong et al., 2016; Garbe et al., 2012; Pelissier Vatter et al., 2018), but are undetectable in our healthy aged mice, suggesting that these cells might represent a more aberrant population that is usually absent in normal murine mammary glands.

In addition to age-related alterations, the identification of HS-AV cells is of considerable interest. These cells were also captured in two initial scRNA-seq studies of young murine mammary glands (Bach et al., 2017; Pal et al., 2017). While Bach et al. did not comment on the hybrid nature of their gene expression, Pal et al. described them as luminal intermediate between the AV and HS lineages. Here, we verify the existence of HS-AV cells by tissue staining and FACS, and define a gene signature distinguishing this population from other luminal cells. Furthermore, we find that HS-AV cells decrease in abundance with age, a potential consequence of lack of self-renewal, differentiation into HS or AV lineage, or out-competition by HS or AV proliferation.

The lineage relationship of HS-AV cells to HS cells and AV cells remains to be determined. Lineage trajectory analysis suggests that HS-AV cells may be more progenitor-like than HS cells and AV cells. However, lineage tracing studies have demonstrated that postnatal HS and AV lineages are largely maintained independently by unipotent progenitors (Lilja et al., 2018; Van Keymeulen et al., 2017; C. Wang et al., 2017); thus, it is unlikely that under normal homeostasis HS-AV cells could function as bi-potent progenitors contributing to the HS and AV lineages. In contrast, it is more likely that HS-AV cells represent a distinct and restricted population independent of HS cells and AV cells. Furthermore, depending on the rate of their cell division, it is possible that these rare HS-AV cells may not form clones of sufficient size to be detectable in sparse labeling and short-term lineage tracing analyses.

If HS-AV cells are a stable population, then it raises the question of whether they serve a specific function in mammary glands. One possibility is that they might serve as dormant resident unipotent or bipotent luminal progenitors that can be activated during tissue regeneration. Similar examples of dormant progenitors have been described in other organs such as lungs (Desai et al., 2014; C. Kim et al., 2005; Vaughan et al., 2015) and liver (Huch et al., 2013; Miyajima et al., 2014). Several observations provide a hint in support of this hypothesis. First, in scRNA-seq lineage trajectory analysis, HS-AV cells are localized to the bifurcation of the HS and AV branches, suggesting the possibility that they might be transcriptionally primed to give rise to cells in either lineage. Second, when compared to HS cells and AV cells, HS-AV cells are enriched for the expression of *Sox9*, a transcription factor reported to confer progenitor property (Guo et al., 2012). HS-AV cells also express other progenitor markers, such as *Kit*, *Aldh1a3*, and *Cd14*. Third, the increased expression of chromatin remodeling proteins in HS-AV cells compared to HS cells and AV cells might suggest a higher potential of differentiation plasticity, given the critical role chromatin modification plays in regulating differentiation (Holliday et al., 2018; Wahl and Spike, 2017). Fourth, the diminished abundance of HS-AV cells with age is consistent with age-dependent depletion of stem/progenitor cells in certain tissue types (Encinas et al., 2011; Nishimura et al., 2005; Schultz and Sinclair, 2016). Finally, it remains possible that HS-AV cells represent a transient intermediate between the HS and AV populations, for instance, during differentiation of HS cells from AV cells, as proposed by Pal et al. (Pal et al., 2017). However, evidence for such a transition has been limited and further studies will shed light on this model. Overall, the identification of HS-AV cells suggests a greater complexity in mammary differentiation than was previously appreciated. Future lineage tracing and functional studies will be required to determine the roles of HS-AV cells in normal development and tissue regeneration and to elucidate their relationship with HS and AV lineages.

Taken together, these single-cell transcriptomic profiles reveal the dynamic heterogeneity in mammary tissues at young and old age. They provide a resource for future studies to understand the interactions between epithelial and stromal cells in aging and cancer.

AUTHOR CONTRIBUTIONS

C.M.L. and J.S.B. conceived the study and designed the experiments. C.M.L. performed the experiments and analyses with assistance from G.K.G. for tissue dissociation, A.R. for scRNA-seq, L.M.S., H.C., L.P., Y.O., and M.J.S. for bioinformatics analyses, H.S., C.T., and K.P.G. for tissue staining and microscopy, and H.S., J.R., and K.M. for FACS analysis of tissues and organoids. All authors contributed to the interpretation of experimental results. C.M.L. and J.S.B. wrote the manuscript, with contribution from all authors. J.S.B. provided funding and project supervision.

ACKNOWLEDGMENTS

We thank Dr. Aviv Regev's laboratory (Broad Institute), including Danielle Dionne, Lan Nguyen, Julia Waldman, Michael Cuoco, Michal Slyper, Christopher Rodman, Orr Ashenberg, Marcin Tabaka, and Orit Rozenblatt-Rosen, as well as Dr. Asaf Rotem (Dana-Farber Cancer Institute) for scRNA-seq assistance. We thank Dr. Roderick Bronson (Harvard Medical School) for histopathology consultation, the Dana-Farber Flow Cytometry Core for FACS service, the Harvard Nikon Imaging Center for microscopy support, Dr. Lai Ding (NeuroTechnology Studio, Brigham and Women's Hospital) for image analysis consultation and assistance, and all members of the Brugge lab, especially Jason Zoeller, Nomeda Girnius, Mackenzie Boedicker, and Angie Martinez Gakidis for assistance in conducting this study and preparing the manuscript. We also thank Dr. Marie Bao for comments on the manuscript. This work was supported in part by funding from Susan G. Komen Postdoctoral Fellowship (C.M.L.), Croucher Postdoctoral Fellowship (C.M.L.), NIH F31 Fellowship (G.K.G.), Hope Funds Postdoctoral Fellowship (Y.O.), Marsha Rivkin Center for Ovarian Cancer Research (Y.O.), Chan Zuckerberg Initiative Donor-Advised Fund (grant number 2018-182734 to L.P.), an advised fund of Silicon Valley Community Foundation (L.P.), NHGRI Career Development Award and Genome Innovator Award (R00HG008399 and R35HG010717 to L.P.), the Susan G. Komen Breast Cancer Foundation (J.S.B.), and the Breast Cancer Research Foundation (J.S.B.).

DECLARATION OF INTERESTS

J.L.G. is a consultant for Glaxo-Smith Kline (GSK), Codagenix, Verseau, Kymera and Array BioPharma and receives sponsored research support from GSK, Array BioPharma and Eli Lilly. A.R. is a founder and equity holder of Celsius Therapeutics, an equity holder in Immunitas Therapeutics and until Aug 31, 2020 was on the SAB of Syros Pharmaceuticals, Neogene Therapeutics, Asimov and ThermoFisher Scientific. From Aug 1, 2020, A.R. is an employee of Genentech, a member of the Roche Group. J.S.B. is on the SAB of Agios Pharmaceuticals, eFFECTOR Therapeutics and Frontier Medicines.

Figure 1. Diverse Epithelial and Stromal Cell Types Identified by scRNA-seq in Mammary Glands
(A) scRNA-seq experimental setup. Freshly dissociated epithelial and stromal cells were analyzed without FACS enrichment.
(B) t-SNE plot showing epithelial and stromal clusters identified based on characteristic markers.
(C-D) Epithelial (C) and stromal (D) cells subsetted and reclustered.
(E-F) Expression of canonical markers of epithelial (E) and stromal (F) cell types.
(G) Heatmap of cell type-specific gene signatures. See also Table S1.
Abbreviations: Myoep, myoepithelial; HS, hormone-sensing; AV, alveolar; LP, luminal progenitors; Fibro, fibroblasts; pan-VL, pan-vascular/lymphatic cells; VE, vascular endothelial cells; P, pericytes; LE, lymphatic endothelial cells; Im, immune cells; My, myeloid cells; DC, dendritic cells; Mac, macrophages; M_a, M_a macrophages, M_b, M_b macrophages; Ly, lymphocytes; NK, natural killer cells; T, T cells; B, B cells.

Figure 2. Prevalent Alterations in Cell Proportions in Aged Mammary Glands
Relative proportions of specified cell types among total cells (A), epithelial cells (B), stromal cells (C), and immune cells (D).

Figure 3. Aged Myoepithelial Cells Show Altered Gene Expression
(A) Myoepithelial cells are identified by basal keratins in scRNA-seq data.
(B) Relative proportion of myoepithelial cells in young (n = 3) and aged (n = 4) mice by scRNA-seq (Fisher's exact test).
(C) Differentially expressed genes in young vs. aged myoepithelial cells. See also Table S3.
(D) Top gene sets identified by pathway analysis of differentially expressed genes in (C), with recurring gene sets highlighted. See also Table S4.
(E-H) Violin plots showing expression of select genes from (D).
(I-L) Representative immunofluorescence staining of KRT15 (I) and SMA (K) in myoepithelial cells (stained as KRT14+ and KRT8-, indicated by arrows). Scale bar = 10 µm. Quantifications are shown in (J) and (L), respectively (n = 6 mice per age group, Student's t test).

Figure 4. Alterations in Luminal Hormone-Sensing (HS) Cells and Alveolar (AV) Cells with Age
(A) AV cells are distinguished by milk-related genes in scRNA-seq data.
(B) HS cells are distinguished by hormone receptors and canonical HS lineage markers.
(C-D) Relative proportion of AV cells (C) and HS cells (D) in young (n = 3) and aged (n = 4) mice by scRNA-seq (Fisher's exact test).
(E) FACS analysis of relative abundance of AV cells and HS cells in young (n = 8) and aged (n = 6) mice. In the representative FACS plots, 10,000 cells are displayed.
(F-G) Differentially expressed genes in young vs. aged AV cells presented as heatmap (F) and specific examples (G). See also Table S3.
(H-I) Differentially expressed genes in young vs. aged HS cells presented as heatmap (H) and specific examples (I). See also Table S3.

Figure 5. Luminal HS-AV Cells Show Age-Dependent Abundance
(A) Relative proportion of HS-AV cells in young (n = 3) and aged (n = 4) mice by scRNA-seq (Fisher's exact test).
(B) Markers distinguishing HS-AV cells from both HS cells and AV cells in young mice. See also Table S5.
(C) Expression pattern of select HS-AV markers in (B).

(D) Dimensionality reduction plot from STREAM lineage trajectory analysis of luminal cells in young mice (n = 3). See also Figure S5D.
(E-F) Representative immunofluorescence staining of HS-AV cells (indicated by arrows) co-expressing HS marker (PR or ER) and AV marker (LTF or MFGE8) in young mice.
(G-J) Immunofluorescence staining of HS-AV cells (indicated by arrows) co-expressing PR and MFGE8 (G) or ER and LTF (I) in young and aged mice. Scale bar = 10 µm. Quantifications are shown in (H) and (J), respectively (n = 6 mice per age group, Student's t-test). In (G) and (I), MFGE8 and LTF are also detected as secreted proteins in the lumen. KRT8 marks luminal cells.
(K) FACS quantification of HS-AV cells co-expressing HS marker CD133 and AV marker CD14 in an independent cohort of young (n = 8) and aged (n = 6) mice. In the representative FACS plots, 10,000 cells are displayed.

Figure 6. Age-Dependent Alterations in Stromal Fibroblasts and Vascular/Lymphatic Cells
(A-D) Fibroblasts, vascular endothelial cells, pericytes, and lymphatic endothelial cells are distinguished by characteristic makers in scRNA-seq data.
(E-H) Relative proportions of the above stromal cell types in young (n = 3) and aged (n = 4) mice by scRNA-seq (Fisher's exact test).
(I) Differentially expressed genes in young vs. aged fibroblasts. See also Table S3.
(J) Top gene sets identified by pathway analysis of differentially expressed genes in (I), with recurring gene sets highlighted. See also Table S4.
(K) Violin plots of select genes in (I) and (J) for fibroblasts.
(L) Differentially expressed genes in young vs. aged vascular endothelial cells. See also Table S3.
(M) Top gene sets identified by pathway analysis of differentially expressed genes in (L), with recurring gene sets highlighted. See also Table S4.
(N) Violin plots of select genes in (L) and (M) for vascular endothelial cells.

Figure 7. Age-Dependent Alterations in Myeloid and Lymphoid Immune Cells
(A-E) M_a and M_b macrophages, dendritic cells, natural killer (NK) cells, and T and B cells, are distinguished by their respective markers in scRNA-seq data (left). Their relative abundance in young (n = 3) and aged (n = 4) mice are shown on the right (Fisher's exact test).
(F) Genes distinguishing M_a and M_b macrophages. See also Table S6.
(G) Differentially expressed genes in young vs. aged M_b macrophages. See also Table S3.
(H-I) Violin plots of cytokines (H) and immunosuppressive ligands (I) upregulated in aged M_b macrophages compared to young cells.

STAR METHODS

RESOURCE AVAILABILITY

Lead Contact

Further information and requests for resources and reagents should be directed to the Lead Contact, Joan Brugge (joan_brugge@hms.harvard.edu).

Materials Availability

This study did not generate new unique reagents.

Data and Code Availability

The scRNA-seq data generated in this study are available at Gene Expression Omnibus (accession number GSE150580).
The R scripts used for scRNA-seq analysis are included in the supplemental information.

EXPERIMENTAL MODEL AND SUBJECT DETAILS

Mice

For single-cell RNA sequencing, young (3-4 months old) and aged (13-14 months old) virgin/nulliparous female mice of a mixed FVB, 129, C57BL/6J background were used in this study. For immunostaining, additional young (3-4 months old) and aged (16-months old) mice were used. For FACS analysis, an independent cohort of young (3-4 months old) and aged (12-14 months old) mice were used. All mice were bred and maintained in house under regular conditions, with 12hr/12hr light/dark cycle, and food (LabDiet 5053) and water ad libitum. Daily vaginal cytology was performed to determine the stage of the estrous cycle, and only actively cycling mice in diestrus were analyzed to avoid confounding effects from hormonal changes. Aged mammary glands were confirmed to have normal histology by a rodent pathologist. All animal work was performed in accordance to protocols approved by the Institutional Animal Care and Use Committee at Harvard University.

METHOD DETAILS

Tissue dissociation for single-cell RNA-seq

Abdominal #4 mammary glands from young (n = 3) and aged (n = 4) mice, with lymph nodes removed, were finely minced and then incubated in a digestion solution containing DMEM/F12 (Gibco 11330), 10% heat-inactivated fetal bovine serum, 2 mg/ml collagenase XI (Sigma C9407), and 0.1 mg/ml hyaluronidase (Sigma H3506) for 1.5 hours at 37°C with constant shaking at 150 rpm. The dissociated cells were then subjected to red blood cell lysis (Biolegend 420301), a 5-minute treatment with 1 U/ml dispase (Stem Cell Technologies 07913) and 0.1 mg/ml DNase (Stem Cell Technologies 07470), a 5-minute treatment with TrypLE (Gibco 12605010), and filtered through a 40 µm cell strainer. Cells were resuspended in PBS containing 0.04% BSA, counted manually under the microscope, and adjusted for loading 7,000 viable cells for single-cell RNA-sequencing.

Single-cell RNA library preparation and sequencing

Single cell capturing and cDNA library generation were performed using the 10X Chromium 3' library construction kit v2 following the manufacturer's instruction. The libraries were then pooled and sequenced by Illumina HiSeq X Ten.

Single-cell RNA-seq data processing

Paired-end reads from Illumina HiSeq were processed and mapped to the mm10 mouse genome using Cell Ranger v2.0. Sequencing saturation as determined by Cell Ranger showed fairly even saturation across samples (young mice: 82.3%, 79.9%, 70.6%; aged mice: 91.3%, 78.6%, 73.3%, 85.1%). We applied stringent filters to eliminate cells with (1) UMI counts < 1,000 or > 60,000, (2) gene counts < 500 or > 2,500, and (3) mitochondrial gene ratio > 10%. This pre-filtering resulted in the detection of 27,998 genes in 13,684 cells, with approximately 1,200-2,800 cells from each sample. A median of 1,649 genes and 5,538 transcripts were captured per cell. The filtered data were then analyzed using Seurat v3 (Butler et al., 2018; Stuart et al., 2019). Cell doublets were estimated using Scrublet (Wolock and Klein, 2019) and DoubletFinder (McGinnis et al., 2019). Lineage trajectory analysis was performed on the luminal epithelial cells of each of the three young samples, as well as on the three samples combined, using STREAM (Chen et al., 2019). Cell cycle estimation was performed in Seurat using the default cell-cycle gene signatures.

Cell type-specific gene signatures

Cell type-specific gene expression signatures were identified as genes that exhibited expression levels >1.25-fold greater (with adjusted *p*-values < 0.05) than each of the other cell types in multiple pairwise differential expression analyses using Seurat's FindMarkers function (Wilcoxon rank sum test). Similarly, gene expression signature for HS-AV luminal cells was generated by identifying genes with expression levels at least 1.25-fold higher or lower (with adjusted *p*-values < 0.05) when compared to luminal HS cells and to luminal AV cells separately. The list of markers distinguishing M_a and M_b macrophages was generated by identifying genes with expression levels differing by at least 2-fold (with adjusted *p*-values < 0.05). Heatmaps for visualizing marker gene expression were median-centered and down-sampled to 100 cells per cell type.

Differential gene expression across age groups

Differential gene expression analysis was performed to compare young and aged samples within each cell type by using a combination of Seurat's FindMarkers function (Wilcoxon rank sum test) and zingeR-edgeR zero-inflated negative binomial analysis (Robinson et al., 2010; Van den Berge et al., 2018) for increased stringency. Differentially expressed genes were identified using three criteria: (i) Grouped analysis criteria: an expression difference of at least 1.5-fold and an adjusted *p*-value of < 0.05 in a grouped comparison of young mice (n = 3) versus aged mice (n = 4); (ii) Per-sample analysis criteria: an expression difference of at least 1.25-fold in all of the 12 possible combinations of young-versus-aged per-sample pairwise comparison; (iii) Cross-method criteria: only genes satisfying criteria (i) and (ii) in both FindMarkers and zingeR-edgeR analyses were included in the final list of differentially expressed genes. Only genes with expression detected in at least 10% of cells in either the young or aged population were analyzed. Heatmaps for visualizing the differentially expressed genes were median-centered and down-sampled to 200 cells per age group, or, in the case of M_b macrophages where fewer cells were detected, down-sampled to 20 cells per group.

Pathway analysis

Pathway analysis was performed on differentially expressed genes using MSigDB v7.0 (Liberzon et al., 2015; Subramanian et al., 2005) curated canonical pathway database. To ensure proper gene name mapping, all mouse gene names were converted to their human homologs using NCBI Homologene prior to analysis in MSigDB. Gene sets with FDR < 0.05 were deemed statistically significant. Up to top 30 enriched gene sets were shown.

Histology and immunostaining

Mouse mammary tissues were fixed in 10% neutral buffered formalin overnight. Paraffin embedding and sectioning were performed by the Rodent Histopathology Core at Harvard Medical School. Immunostaining was performed on n = 6 animals per age group. For immunofluorescence staining, the following antibodies were used: ACTA2/SMA (Dako M0851, 1:100), KRT15 (BioLegend 833904, 1:50), KRT14 (BioLegend 905301, 1:250; Abcam ab206100, 1:150), KRT8 (DSHB Troma-I, 1:100; Abcam ab192467, 1:100), ARG1 (CST 66297, 1:100), PR (CST 8757, 1:25; Dako A0098, 1:20), ER (Santa Cruz sc-542, 1:20; BioRad MCA1799T, 1:25), LTF (Bioss bs-5810, 1:50), MFGE8 (Thermo MA5-23913, 1:50; R&D Systems MAB2805, 1:50). LTF and MFGE8 antibodies were validated by staining lactating mammary glands. For immunohistochemistry staining, the following antibodies were used: PDGFRα (CST 3174, 1:200), CD163 (Abcam ab182422, 1:500), CD8 (CST 98941, 1:50). Images were analyzed in a blinded fashion using Fiji (Schindelin et al., 2012).

FACS analysis

For FACS analysis of mammary tissues, freshly dissected #2, 3, and 4 mammary glands from young (n = 8) and aged (n = 6) mice were digested as described above. Cells were then incubated with CD16/32 antibody (eBioscience 16-0161) to block Fc receptor and with rat serum (eBioscience 24-5555) to block non-specific binding, and then stained for 30 minutes with the following antibodies/reagents: CD45-FITC (BioLegend 103108, 1:200), CD31-FITC (BioLegend 102405, 1:50), TER119-FITC (BioLegend 116205, 1:100), EPCAM-BV605 (BioLegend 118227, 1:100), CD133-APC (BioLegend 141207, 1:50), CD14-PE (BioLegend 150106, 1:50), and DAPI (1 µg/mL). Cell sorting and analysis was carried out on a BD FACSAria II cell sorter and FlowJo using the following gating strategy: SSC-H/SSC-W singlets, FSC-H/FSC-W singlets, DAPI- live cells, CD45-/CD31-/TER119- Lin- cells, EPCAM-high luminal epithelial cells. The gated luminal population was then analyzed for expression of CD133 and CD14. For FACS analysis of organoid cultures, primary mammary organoids were generated from 3-4 month-old FVB female mice using the aforementioned tissue digestion protocol and propagated for six weeks in defined basement-membrane extract and culture media, as described previously (Rosenbluth et al., 2020). Organoids were digested into single cells using TrypLE, blocked with CD16/32 antibody and rat serum, and then stained with a similar antibody panel as described above. EPCAM-BV605 was omitted in order to allow for subsequent microscopy examination of cells double positive for CD133-APC and CD14-PE without introducing spectral overlapping from EPCAM-BV605.

QUANTIFICATION AND STATISTICAL ANALYSIS

For cell type composition comparison between young and aged mammary glands in scRNA-seq data, statistically significant difference was determined by Fisher's exact test and Chi-square test. We noted that because all proportions sum to one in scRNA-seq data, an increase in the proportion of one cell population will necessarily lead to a decrease in the proportions of other cell populations. However, alternative multivariate tests for compositional data analysis (e.g. Dirichlet-multinomial regression test) were not applied as they have increased type 2 error over type 1 error. For quantification by immunofluorescence staining, immunohistochemistry staining, and FACS analysis, statistically significant difference was determined by Student's t-test. Statistical analysis was performed by using the GraphPad Prism software, and *p*-values < 0.05 (two-tailed) were deemed statistically significant. Bar graphs represent mean ± SEM.

LIST OF SUPPLEMENTARY TABLES AND R SCRIPT

Table S1 (related to Figure 1). Cell type-specific gene signatures.

Table S2 (related to Figures 2). Cell type proportions in young and aged mammary glands.

Table S3 (related to Figures 3, 4, 6, and 7). Young-versus-aged differentially expressed genes per cell type.

Table S4 (related to Figures 3, 4, and 6). Pathway analysis of age-regulated genes per cell type.

Table S5 (related to Figure 5). List of gene markers that distinguish HS-AV cells from both HS cells and AV cells.

Table S6 (related to Figure 7). List of markers distinguishing the two macrophage subpopulations.

R script (related to STAR Methods). scRNA-seq analysis R script.

REFERENCES

Ace, C.I., and Okulicz, W.C. (1998). A progesterone-induced endometrial homolog of a new candidate tumor suppressor, DMBT1. J. Clin. Endocrinol. Metab. *83*, 3569–3573.

Adriance, M.C., Inman, J.L., Petersen, O.W., and Bissell, M.J. (2005). Myoepithelial cells: good fences make good neighbors. Breast Cancer Res *7*, 190–197.

Agarwal, A.K., Tunison, K., Dalal, J.S., Nagamma, S.S., Hamra, F.K., Sankella, S., Shao, X., Auchus, R.J., and Garg, A. (2017). Metabolic, Reproductive, and Neurologic Abnormalities in Agpat1-Null Mice. Endocrinology *158*, 3954–3973.

Arango Duque, G., and Descoteaux, A. (2014). Macrophage cytokines: involvement in immunity and infectious diseases. Front Immunol *5*, 491.

Azam, S., Sjölander, A., Eriksson, M., Gabrielson, M., Czene, K., and Hall, P. (2019). Determinants of Mammographic Density Change. JNCI Cancer Spectr *3*, pkz004.

Bach, K., Pensa, S., Grzelak, M., Hadfield, J., Adams, D.J., Marioni, J.C., and Khaled, W.T. (2017). Differentiation dynamics of mammary epithelial cells revealed by single-cell RNA sequencing. Nat Commun *8*, 2128.

Bazzoni, G., and Dejana, E. (2004). Endothelial cell-to-cell junctions: molecular organization and role in vascular homeostasis. Physiol Rev *84*, 869–901.

Bjørge, L., Jensen, T.S., Kristoffersen, E.K., Ulstein, M., and Matre, R. (1996). Identification of the complement regulatory protein CD59 in human colostrum and milk. Am. J. Reprod. Immunol. *35*, 43–50.

Blencowe, B.J., Issner, R., Nickerson, J.A., and Sharp, P.A. (1998). A coactivator of pre-mRNA splicing. Gene Dev *12*, 996–1009.

Booth, B.W., Boulanger, C.A., and Smith, G.H. (2007). Alveolar progenitor cells develop in mouse mammary glands independent of pregnancy and lactation. J Cell Physiol *212*, 729–736.

Brady, N.J., Chuntova, P., and Schwertfeger, K.L. (2016). Macrophages: Regulators of the Inflammatory Microenvironment during Mammary Gland Development and Breast Cancer. Mediators Inflamm. *2016*, 4549676–13.

Brisken, C., and Ataca, D. (2015). Endocrine hormones and local signals during the development of the mouse mammary gland. Wiley Interdiscip Rev Dev Biol *4*, 181–195.

Bronte, V., and Zanovello, P. (2005). Regulation of immune responses by L-arginine metabolism. Nat Rev Immunol *5*, 641–654.

Burchfield, J.S., Li, Q., Wang, H.Y., and Wang, R.-F. (2015). JMJD3 as an epigenetic regulator in development and disease. Int J Biochem Cell Biol *67*, 148–157.

Butler, A., Hoffman, P., Smibert, P., and Satija, R. (2018). Integrating single-cell transcriptomic data across different conditions, technologies, and species. Nat Biotechnol *36*, 411–420.

Cella, M., Döhring, C., Samaridis, J., Dessing, M., Brockhaus, M., Lanzavecchia, A., and Colonna, M. (1997). A novel inhibitory receptor (ILT3) expressed on monocytes, macrophages, and dendritic cells involved in antigen processing. J Exp Med *185*, 1743–1751.

Chen, H., Albergante, L., Hsu, J.Y., Lareau, C.A., Bosco, Lo, G., Guan, J., Zhou, S., Gorban, A.N., Bauer, D.E., Aryee, M.J., et al. (2019). Single-cell trajectories reconstruction, exploration and mapping of omics data with STREAM. Nat Commun *10*, 1903–1914.

Clarke, R.B., Howell, A., Potten, C.S., and Anderson, E. (1997). Dissociation between steroid receptor expression and cell proliferation in the human breast. Cancer Res *57*, 4987–4991.

Cohen, B., Shimizu, M., Izrailit, J., Ng, N.F.L., Buchman, Y., Pan, J.G., Dering, J., and Reedijk, M. (2010). Cyclin D1 is a direct target of JAG1-mediated Notch signaling in breast cancer. Breast Cancer Res Treat *123*, 113–124.

Crisà, A., Ferrè, F., Chillemi, G., and Moioli, B. (2016). RNA-Sequencing for profiling goat milk transcriptome in colostrum and mature milk. BMC Vet. Res. *12*, 264–21.

Dawson, C.A., Pal, B., Vaillant, F., Gandolfo, L.C., Liu, Z., Bleriot, C., Ginhoux, F., Smyth, G.K., Lindeman, G.J., Mueller, S.N., et al. (2020). Tissue-resident ductal macrophages survey the mammary epithelium and facilitate tissue remodelling. Nat Cell Biol *1*, 201–213.
Deng, M., Gui, X., Kim, J., Xie, L., Chen, W., Li, Z., He, L., Chen, Y., Chen, H., Luo, W., et al. (2018). LILRB4 signalling in leukaemia cells mediates T cell suppression and tumour infiltration. Nature *562*, 605–609.
Denissov, S., Hofemeister, H., Marks, H., Kranz, A., Ciotta, G., Singh, S., Anastassiadis, K., Stunnenberg, H.G., and Stewart, A.F. (2014). Mll2 is required for H3K4 trimethylation on bivalent promoters in embryonic stem cells, whereas Mll1 is redundant. Development *141*, 526–537.
Desai, T.J., Brownfield, D.G., and Krasnow, M.A. (2014). Alveolar progenitor and stem cells in lung development, renewal and cancer. Nature *507*, 190–194.
Diaz Brinton, R. (2012). Minireview: translational animal models of human menopause: challenges and emerging opportunities. Endocrinology *153*, 3571–3578.
Donato, A.J., Morgan, R.G., Walker, A.E., and Lesniewski, L.A. (2015). Cellular and molecular biology of aging endothelial cells. J. Mol. Cell. Cardiol. *89*, 122–135.
Dong, Q., Gao, H., Shi, Y., Zhang, F., Gu, X., Wu, A., Wang, D., Chen, Y., Bandyopadhyay, A., Yeh, I.-T., et al. (2016). Aging is associated with an expansion of CD49fhi mammary stem cells that show a decline in function and increased transformation potential. Aging *8*, 2754–2776.
Edwards, E., Kopp, J.L., Naramura, M., Band, H., and Band, V. (2014). The role of Sox9 in mouse mammary gland development and maintenance of mammary stem and luminal progenitor cells. BMC Dev. Biol. *14*, 47–11.
Encinas, J.M., Michurina, T.V., Peunova, N., Park, J.-H., Tordo, J., Peterson, D.A., Fishell, G., Koulakov, A., and Enikolopov, G. (2011). Division-coupled astrocytic differentiation and age-related depletion of neural stem cells in the adult hippocampus. Cell Stem Cell *8*, 566–579.
Fata, J.E., Chaudhary, V., and Khokha, R. (2001). Cellular turnover in the mammary gland is correlated with systemic levels of progesterone and not 17beta-estradiol during the estrous cycle. Biol Reprod *65*, 680–688.
Finch, C.E., Felicio, L.S., Mobbs, C.V., and Nelson, J.F. (1984). Ovarian and steroidal influences on neuroendocrine aging processes in female rodents. Endocr. Rev. *5*, 467–497.
Flurkey, K., Currer, J.M., and Harrison, D.E. (2007). The Mouse in Biomedical Research. Vol III: Normative Biology, Husbandry, and Models (Elsevier).
Franklin, R.A., Liao, W., Sarkar, A., Kim, M.V., Bivona, M.R., Liu, K., Pamer, E.G., and Li, M.O. (2014). The cellular and molecular origin of tumor-associated macrophages. Science *344*, 921–925.
Frantz, C., Stewart, K.M., and Weaver, V.M. (2010). The extracellular matrix at a glance. J Cell Sci *123*, 4195–4200.
Froimchuk, E., Jang, Y., and Ge, K. (2017). Histone H3 lysine 4 methyltransferase KMT2D. Gene *627*, 337–342.
Fu, N.Y., Nolan, E., Lindeman, G.J., and Visvader, J.E. (2020). Stem Cells and the Differentiation Hierarchy in Mammary Gland Development. Physiol Rev *100*, 489–523.
Gannon, P.O., Godin-Ethier, J., Hassler, M., Delvoye, N., Aversa, M., Poisson, A.O., Péant, B., Alam Fahmy, M., Saad, F., Lapointe, R., et al. (2010). Androgen-regulated expression of arginase 1, arginase 2 and interleukin-8 in human prostate cancer. PLoS ONE *5*, e12107.
Gantt, S., Gervassi, A., Jaspan, H., and Horton, H. (2014). The role of myeloid-derived suppressor cells in immune ontogeny. Front Immunol *5*, 387.
Garbe, J.C., Pepin, F., Pelissier, F.A., Sputova, K., Fridriksdottir, A.J., Guo, D.E., Villadsen, R., Park, M., Petersen, O.W., Borowsky, A.D., et al. (2012). Accumulation of Multipotent Progenitors with a Basal Differentiation Bias during Aging of Human Mammary Epithelia. Cancer Res *72*, 3687–3701.

Gertig, D., Stillman, I., Byrne, C., Spiegelman, D., Schnitt, S., Connolly, J., Colditz, G., and Hunter, D. (1999). Association of Age and Reproductive Factors with Benign Breast Tissue Composition. Cancer Epidemiol Biomarkers Prev *8*, 873–879.

Ginger, M.R., and Rosen, J.M. (2003). Pregnancy-induced changes in cell-fate in the mammary gland. Breast Cancer Res *5*, 192–197.

Ginhoux, F., and Guilliams, M. (2016). Tissue-Resident Macrophage Ontogeny and Homeostasis. Immunity *44*, 439–449.

Giraddi, R.R., Chung, C.-Y., Heinz, R.E., Balcioglu, O., Novotny, M., Trejo, C.L., Dravis, C., Hagos, B.M., Mehrabad, E.M., Rodewald, L.-W., et al. (2018). Single-Cell Transcriptomes Distinguish Stem Cell State Changes and Lineage Specification Programs in Early Mammary Gland Development. Cell Rep *24*, 1653–1666.e1657.

Giraddi, R.R., Shehata, M., Gallardo, M., Blasco, M.A., Simons, B.D., and Stingl, J. (2015). Stem and progenitor cell division kinetics during postnatal mouse mammary gland development. Nat Commun *6*, 8487.

Gouon-Evans, V., Lin, E.Y., and Pollard, J.W. (2002). Requirement of macrophages and eosinophils and their cytokines/chemokines for mammary gland development. Breast Cancer Research *4*, 155–164.

Greten, F.R., and Grivennikov, S.I. (2019). Inflammation and Cancer: Triggers, Mechanisms, and Consequences. Immunity *51*, 27–41.

Gudjonsson, T., Rønnov-Jessen, L., Villadsen, R., Rank, F., Bissell, M.J., and Petersen, O.W. (2002). Normal and tumor-derived myoepithelial cells differ in their ability to interact with luminal breast epithelial cells for polarity and basement membrane deposition. J Cell Sci *115*, 39–50.

Guerriero, J.L. (2018). Macrophages: The Road Less Traveled, Changing Anticancer Therapy. Trends Mol Med *24*, 472–489.

Gundlah, C., Alves, S.E., Clark, J.A., Pai, L.-Y., Schaeffer, J.M., and Rohrer, S.P. (2005). Estrogen receptor-beta regulates tryptophan hydroxylase-1 expression in the murine midbrain raphe. Biol. Psychiatry *57*, 938–942.

Guo, W., Keckesova, Z., Donaher, J.L., Shibue, T., Tischler, V., Reinhardt, F., Itzkovitz, S., Noske, A., Zürrer-Härdi, U., Bell, G., et al. (2012). Slug and Sox9 cooperatively determine the mammary stem cell state. Cell *148*, 1015–1028.

Gusterson, B.A., Warburton, M.J., Mitchell, D., Ellison, M., Neville, A.M., and Rudland, P.S. (1982). Distribution of myoepithelial cells and basement membrane proteins in the normal breast and in benign and malignant breast diseases. Cancer Res *42*, 4763–4770.

Haaksma, C.J., Schwartz, R.J., and Tomasek, J.J. (2011). Myoepithelial cell contraction and milk ejection are impaired in mammary glands of mice lacking smooth muscle alpha-actin. Biol Reprod *85*, 13–21.

Halper, J., and Kjaer, M. (2014). Basic components of connective tissues and extracellular matrix: elastin, fibrillin, fibulins, fibrinogen, fibronectin, laminin, tenascins and thrombospondins. Adv Exp Med Biol *802*, 31–47.

Han, X., Han, X., Wang, R., Wang, R., Zhou, Y., Zhou, Y., Fei, L., Fei, L., Sun, H., Sun, H., et al. (2018). Mapping the Mouse Cell Atlas by Microwell-Seq. Cell *172*, 1091–1107.e17.

Hart, B.L., Steinbock, R.T., Mettler, F.A., Pathak, D.R., and Bartow, S.A. (1989). Age and race related changes in mammographic parenchymal patterns. Cancer *63*, 2537–2539.

Hickey, D.K., Fahey, J.V., and Wira, C.R. (2013). Estrogen receptor α antagonists mediate changes in CCL20 and CXCL1 secretions in the murine female reproductive tract. Am. J. Reprod. Immunol. *69*, 159–167.

Hoeffel, G., and Ginhoux, F. (2018). Fetal monocytes and the origins of tissue-resident macrophages. Cell. Immunol. *330*, 5–15.

Holliday, H., Baker, L.A., Junankar, S.R., Clark, S.J., and Swarbrick, A. (2018). Epigenomics of mammary gland development. Breast Cancer Res *20*, 100–111.

Hsu, J., Hodgins, J.J., Marathe, M., Nicolai, C.J., Bourgeois-Daigneault, M.-C., Trevino, T.N., Azimi, C.S., Scheer, A.K., Randolph, H.E., Thompson, T.W., et al. (2018). Contribution of NK cells to immunotherapy mediated by PD-1/PD-L1 blockade. J Clin Invest *128*, 4654–4668.

Hu, M., Yao, J., Carroll, D.K., Weremowicz, S., Chen, H., Carrasco, D., Richardson, A., Violette, S., Nikolskaya, T., Nikolsky, Y., et al. (2008). Regulation of in situ to invasive breast carcinoma transition. Cancer Cell *13*, 394–406.

Huch, M., Dorrell, C., Boj, S.F., van Es, J.H., Li, V.S.W., van de Wetering, M., Sato, T., Hamer, K., Sasaki, N., Finegold, M.J., et al. (2013). In vitro expansion of single Lgr5+ liver stem cells induced by Wnt-driven regeneration. Nature *494*, 247–250.

Huh, S.J., Clement, K., Jee, D., Merlini, A., Choudhury, S., Maruyama, R., Yoo, R., Chytil, A., Boyle, P., Ran, F.A., et al. (2015). Age- and pregnancy-associated DNA methylation changes in mammary epithelial cells. Stem Cell Reports *4*, 297–311.

Hutson, S.W., Cowen, P.N., and Bird, C.C. (1985). Morphometric studies of age related changes in normal human breast and their significance for evolution of mammary cancer. J Clin Pathol *38*, 281–287.

Inman, J.L., Robertson, C., Mott, J.D., and Bissell, M.J. (2015). Mammary gland development: cell fate specification, stem cells and the microenvironment. Development *142*, 1028–1042.

Jahchan, N.S., Wang, D., Bissell, M.J., and Luo, K. (2012). SnoN regulates mammary gland alveologenesis and onset of lactation by promoting prolactin/Stat5 signaling. Development *139*, 3147–3156.

Jahchan, N.S., You, Y.-H., Muller, W.J., and Luo, K. (2010). Transforming growth factor-beta regulator SnoN modulates mammary gland branching morphogenesis, postlactational involution, and mammary tumorigenesis. Cancer Res *70*, 4204–4213.

Jäppinen, N., Félix, I., Lokka, E., Tyystjärvi, S., Pynttäri, A., Lahtela, T., Gerke, H., Elima, K., Rantakari, P., and Salmi, M. (2019). Fetal-derived macrophages dominate in adult mammary glands. Nat Commun *10*, 281–12.

Jenkins, E.O., Deal, A.M., Anders, C.K., Prat, A., Perou, C.M., Carey, L.A., and Muss, H.B. (2014). Age-specific changes in intrinsic breast cancer subtypes: a focus on older women. Oncologist *19*, 1076–1083.

Joshi, P.A., Jackson, H.W., Beristain, A.G., Di Grappa, M.A., Mote, P.A., Clarke, C.L., Stingl, J., Waterhouse, P.D., and Khokha, R. (2010). Progesterone induces adult mammary stem cell expansion. Nature *465*, 803–807.

Kamikawa, A., Ichii, O., Yamaji, D., Imao, T., Suzuki, C., Okamatsu-Ogura, Y., Terao, A., Kon, Y., and Kimura, K. (2009). Diet-induced obesity disrupts ductal development in the mammary glands of nonpregnant mice. Dev Dyn *238*, 1092–1099.

Kanaya, N., Chang, G., Wu, X., Saeki, K., Bernal, L., Shim, H.-J., Wang, J., Warden, C., Yamamoto, T., Li, J., et al. (2019). Single-cell RNA-sequencing analysis of estrogen- and endocrine-disrupting chemical-induced reorganization of mouse mammary gland. Commun Biol *2*, 406–415.

Kankel, K.F., and Reinauer, H. (1976). Activity of pyruvate dehydrogenase complex in the mammary gland of normal and diabetic rats. Diabetologia *12*, 149–154.

Kim, C., Jackson, E., Woolfenden, A., Lawrence, S., Babar, I., Vogel, S., Crowley, D., Bronson, R., and Jacks, T. (2005). Identification of bronchioalveolar stem cells in normal lung and lung cancer. Cell *121*, 823–835.

Kim, Y.-E., Won, M., Lee, S.-G., Park, C., Song, C.-H., and Kim, K.K. (2019). RBM47-regulated alternative splicing of TJP1 promotes actin stress fiber assembly during epithelial-to-mesenchymal transition. Oncogene *38*, 6521–6536.

Kzhyshkowska, J., Rusch, A., Wolf, H., and Dobner, T. (2003). Regulation of transcription by the heterogeneous nuclear ribonucleoprotein E1B-AP5 is mediated by complex formation with the novel bromodomain-containing protein BRD7. Biochem J *371*, 385–393.

LaBarge, M.A., Mora-Blanco, E.L., Samson, S., and Miyano, M. (2016). Breast Cancer beyond the Age of Mutation. Gerontology *62*, 434–442.
Landskron, G., la Fuente, De, M., Thuwajit, P., Thuwajit, C., and Hermoso, M.A. (2014). Chronic inflammation and cytokines in the tumor microenvironment. J Immunol Res *2014*, 149185–19.
Laporta, J., Keil, K.P., Vezina, C.M., and Hernandez, L.L. (2014). Peripheral serotonin regulates maternal calcium trafficking in mammary epithelial cells during lactation in mice. PLoS ONE *9*, e110190.
Li, T., Sun, L., Miller, N., Nicklee, T., Woo, J., Hulse-Smith, L., Tsao, M.-S., Khokha, R., Martin, L., and Boyd, N. (2005). The association of measured breast tissue characteristics with mammographic density and other risk factors for breast cancer. Cancer Epidemiol Biomarkers Prev *14*, 343–349.
Liberzon, A., Birger, C., Thorvaldsdottir, H., Ghandi, M., Mesirov, J.P., and Tamayo, P. (2015). The Molecular Signatures Database (MSigDB) hallmark gene set collection. Cell Syst *1*, 417–425.
Lilja, A.M., Rodilla, V., Huyghe, M., Hannezo, E., Landragin, C., Renaud, O., Leroy, O., Rulands, S., Simons, B.D., and Fre, S. (2018). Clonal analysis of Notch1-expressing cells reveals the existence of unipotent stem cells that retain long-term plasticity in the embryonic mammary gland. Nat Cell Biol *20*, 677–687.
Lim, E., Vaillant, F., Wu, D., Forrest, N.C., Pal, B., Hart, A.H., Asselin-Labat, M.-L., Gyorki, D.E., Ward, T., Partanen, A., et al. (2009). Aberrant luminal progenitors as the candidate target population for basal tumor development in BRCA1 mutation carriers. Nat Med *15*, 907–913.
Littlejohn, M.D., Walker, C.G., Ward, H.E., Lehnert, K.B., Snell, R.G., Verkerk, G.A., Spelman, R.J., Clark, D.A., and Davis, S.R. (2010). Effects of reduced frequency of milk removal on gene expression in the bovine mammary gland. Physiol Genomics *41*, 21–32.
Lomniczi, A., Wright, H., Castellano, J.M., Matagne, V., Toro, C.A., Ramaswamy, S., Plant, T.M., and Ojeda, S.R. (2015). Epigenetic regulation of puberty via Zinc finger protein-mediated transcriptional repression. Nat Commun *6*, 10195–16.
Lu, D., Ni, Z., Liu, X., Feng, S., Dong, X., Shi, X., Zhai, J., Mai, S., Jiang, J., Wang, Z., et al. (2019). Beyond T Cells: Understanding the Role of PD-1/PD-L1 in Tumor-Associated Macrophages. J Immunol Res *2019*, 1919082–1919087.
Lv, Y., Guan, W., Qiao, H., Wang, C., Chen, F., Zhang, Y., and Liao, Z. (2015). Veterinary Medicine and Omics (Veterinomics): Metabolic Transition of Milk Triacylglycerol Synthesis in Sows from Late Pregnancy to Lactation. Omics *19*, 602–616.
Maishi, N., and Hida, K. (2017). Tumor endothelial cells accelerate tumor metastasis. Cancer Sci *108*, 1921–1926.
Mas, G., Blanco, E., Ballaré, C., Sansó, M., Spill, Y.G., Hu, D., Aoi, Y., Le Dily, F., Shilatifard, A., Marti-Renom, M.A., et al. (2018). Promoter bivalency favors an open chromatin architecture in embryonic stem cells. Nat Genet *50*, 1452–1462.
Matsuda, M., Imaoka, T., Vomachka, A.J., Gudelsky, G.A., Hou, Z., Mistry, M., Bailey, J.P., Nieport, K.M., Walther, D.J., Bader, M., et al. (2004). Serotonin regulates mammary gland development via an autocrine-paracrine loop. Dev Cell *6*, 193–203.
McCormick, N.H., Hennigar, S.R., Kiselyov, K., and Kelleher, S.L. (2014). The biology of zinc transport in mammary epithelial cells: implications for mammary gland development, lactation, and involution. J Mammary Gland Biol *19*, 59–71.
McCormack, V.A., Perry, N.M., Vinnicombe, S.J., and Santos Silva, Dos, I. (2010). Changes and tracking of mammographic density in relation to Pike's model of breast tissue aging: a UK longitudinal study. International Journal of Cancer Journal International Du Cancer *127*, 452–461.

McGinnis, C.S., Murrow, L.M., and Gartner, Z.J. (2019). DoubletFinder: Doublet Detection in Single-Cell RNA Sequencing Data Using Artificial Nearest Neighbors. Cell Syst *8*, 329–337.e4.

Miyajima, A., Tanaka, M., and Itoh, T. (2014). Stem/progenitor cells in liver development, homeostasis, regeneration, and reprogramming. Cell Stem Cell *14*, 561–574.

Molyneux, G., Geyer, F.C., Magnay, F.-A., McCarthy, A., Kendrick, H., Natrajan, R., Mackay, A., Grigoriadis, A., Tutt, A., Ashworth, A., et al. (2010). BRCA1 basal-like breast cancers originate from luminal epithelial progenitors and not from basal stem cells. Cell Stem Cell *7*, 403–417.

Muraoka, R.S., Koh, Y., Roebuck, L.R., Sanders, M.E., Brantley-Sieders, D., Gorska, A.E., Moses, H.L., and Arteaga, C.L. (2003). Increased malignancy of Neu-induced mammary tumors overexpressing active transforming growth factor beta1. Mol Cell Biol *23*, 8691–8703.

Muraoka-Cook, R.S., Kurokawa, H., Koh, Y., Forbes, J.T., Roebuck, L.R., Barcellos-Hoff, M.H., Moody, S.E., Chodosh, L.A., and Arteaga, C.L. (2004). Conditional overexpression of active transforming growth factor beta1 in vivo accelerates metastases of transgenic mammary tumors. Cancer Res *64*, 9002–9011.

Nakano, S., Iyama, K., Ogawa, M., Yoshioka, H., Sado, Y., Oohashi, T., and Ninomiya, Y. (1999). Differential tissular expression and localization of type IV collagen alpha1(IV), alpha2(IV), alpha5(IV), and alpha6(IV) chains and their mRNA in normal breast and in benign and malignant breast tumors. Lab Invest *79*, 281–292.

Natali, P.G., Giacomini, P., Bigotti, G., Nicotra, M.R., Bellocci, M., and De Martino, C. (1984). Heterogeneous distribution of actin, myosin, fibronectin and basement membrane antigens in primary and metastatic human breast cancer. Virchows Arch a Pathol Anat Histopathol *405*, 69–83.

Nguyen, Q.H., Pervolarakis, N., Blake, K., Ma, D., Davis, R.T., James, N., Phung, A.T., Willey, E., Kumar, R., Jabart, E., et al. (2018). Profiling human breast epithelial cells using single cell RNA sequencing identifies cell diversity. Nat Commun *9*, 2028.

Nishimura, E.K., Granter, S.R., and Fisher, D.E. (2005). Mechanisms of hair graying: incomplete melanocyte stem cell maintenance in the niche. Science *307*, 720–724.

Oakley, R., and Tharakan, B. (2014). Vascular hyperpermeability and aging. Aging Dis *5*, 114–125.

Ono, Y.J., Terai, Y., Tanabe, A., Hayashi, A., Hayashi, M., Yamashita, Y., Kyo, S., and Ohmichi, M. (2014). Decorin induced by progesterone plays a crucial role in suppressing endometriosis. J Endocrinol *223*, 203–216.

Ormerod, E.J., Warburton, M.J., Hughes, C., and Rudland, P.S. (1983). Synthesis of basement membrane proteins by rat mammary epithelial cells. Dev Biol *96*, 269–275.

Pal, B., Chen, Y., Vaillant, F., Jamieson, P., Gordon, L., Rios, A.C., Wilcox, S., Fu, N., Liu, K.H., Jackling, F.C., et al. (2017). Construction of developmental lineage relationships in the mouse mammary gland by single-cell RNA profiling. Nat Commun *8*, 1627.

Pandey, P.R., Saidou, J., and Watabe, K. (2010). Role of myoepithelial cells in breast tumor progression. Front Biosci (Landmark Ed) *15*, 226–236.

Pankov, R., and Yamada, K.M. (2002). Fibronectin at a glance. J Cell Sci *115*, 3861–3863.

Pelissier Vatter, F.A., Schapiro, D., Chang, H., Borowsky, A.D., Lee, J.K., Parvin, B., Stampfer, M.R., LaBarge, M.A., Bodenmiller, B., and Lorens, J.B. (2018). High-Dimensional Phenotyping Identifies Age-Emergent Cells in Human Mammary Epithelia. Cell Rep *23*, 1205–1219.

Pelissier, F.A., Garbe, J.C., Ananthanarayanan, B., Miyano, M., Lin, C., Jokela, T., Kumar, S., Stampfer, M.R., Lorens, J.B., and LaBarge, M.A. (2014). Age-related dysfunction in mechanotransduction impairs differentiation of human mammary epithelial progenitors. Cell Rep *7*, 1926–1939.

Polyak, K., and Kalluri, R. (2010). The Role of the Microenvironment in Mammary Gland Development and Cancer. Cold Spring Harb Perspect Biol *2*, a003244–a003244.

Proia, T.A., Keller, P.J., Gupta, P.B., Klebba, I., Jones, A.D., Sedic, M., Gilmore, H., Tung, N., Naber, S.P., Schnitt, S., et al. (2011). Genetic Predisposition Directs Breast Cancer Phenotype by Dictating Progenitor Cell Fate. Cell Stem Cell *8*, 149–163.

Raafat, A., Strizzi, L., Lashin, K., Ginsburg, E., McCurdy, D., Salomon, D., Smith, G.H., Medina, D., and Callahan, R. (2012). Effects of age and parity on mammary gland lesions and progenitor cells in the FVB/N-RC mice. PLoS ONE *7*, e43624.

Robinson, M.D., McCarthy, D.J., and Smyth, G.K. (2010). edgeR: a Bioconductor package for differential expression analysis of digital gene expression data. Bioinformatics *26*, 139–140.

Rosenbluth, J.M., Schackmann, R.C.J., Gray, G.K., Selfors, L.M., Li, C.M.-C., Boedicker, M., Kuiken, H.J., Richardson, A., Brock, J., Garber, J., et al. (2020). Organoid cultures from normal and cancer-prone human breast tissues preserve complex epithelial lineages. Nat Commun *11*, 1711–1714.

Russell, T.D., Schaack, J., Orlicky, D.J., Palmer, C., Chang, B.H.-J., Chan, L., and McManaman, J.L. (2011). Adipophilin regulates maturation of cytoplasmic lipid droplets and alveolae in differentiating mammary glands. J Cell Sci *124*, 3247–3253.

Russo, J., Balogh, G.A., and Russo, I.H. (2008). Full-term pregnancy induces a specific genomic signature in the human breast. Cancer Epidemiol Biomarkers Prev *17*, 51–66.

Sampath, D., Winneker, R.C., and Zhang, Z. (2002). The angiogenic factor Cyr61 is induced by the progestin R5020 and is necessary for mammary adenocarcinoma cell growth. Endocrine *18*, 147–159.

Sanada, F., Taniyama, Y., Muratsu, J., Otsu, R., Shimizu, H., Rakugi, H., and Morishita, R. (2018). Source of Chronic Inflammation in Aging. Front Cardiovasc Med *5*, 12.

Santos, dos, C.O., Dolzhenko, E., Hodges, E., Smith, A.D., and Hannon, G.J. (2015). An epigenetic memory of pregnancy in the mouse mammary gland. Cell Rep *11*, 1102–1109.

Schindelin, J., Arganda-Carreras, I., Frise, E., Kaynig, V., Longair, M., Pietzsch, T., Preibisch, S., Rueden, C., Saalfeld, S., Schmid, B., et al. (2012). Fiji: an open-source platform for biological-image analysis. Nat Methods *9*, 676–682.

Schultz, M.B., and Sinclair, D.A. (2016). When stem cells grow old: phenotypes and mechanisms of stem cell aging. Development *143*, 3–14.

Shackleton, M., Vaillant, F., Simpson, K.J., Stingl, J., Smyth, G.K., Asselin-Labat, M.-L., Wu, L., Lindeman, G.J., and Visvader, J.E. (2006). Generation of a functional mammary gland from a single stem cell. Nature *439*, 84–88.

Sigurdsson, V., Ingthorsson, S., Hilmarsdottir, B., Gustafsdottir, S.M., Franzdottir, S.R., Arason, A.J., Steingrimsson, E., Magnusson, M.K., and Gudjonsson, T. (2013). Expression and functional role of sprouty-2 in breast morphogenesis. PLoS ONE *8*, e60798.

Singletary, K.W., and McNary, M.Q. (1992). Effect of moderate ethanol consumption on mammary gland structural development and DNA synthesis in the female rat. Alcohol *9*, 95–101.

Sirka, O.K., Shamir, E.R., and Ewald, A.J. (2018). Myoepithelial cells are a dynamic barrier to epithelial dissemination. J Cell Biol *217*, 3368–3381.

Sopel, M. (2010). The myoepithelial cell: its role in normal mammary glands and breast cancer. Folia Morphol. (Warsz) *69*, 1–14.

Standaert, L., Adriaens, C., Radaelli, E., Van Keymeulen, A., Blanpain, C., Hirose, T., Nakagawa, S., and Marine, J.-C. (2014). The long noncoding RNA Neat1 is required for mammary gland development and lactation. Rna *20*, 1844–1849.

Stingl, J., Eirew, P., Ricketson, I., Shackleton, M., Vaillant, F., Choi, D., Li, H.I., and Eaves, C.J. (2006). Purification and unique properties of mammary epithelial stem cells. Nature *439*, 993–997.

Stuart, T., Butler, A., Hoffman, P., Hafemeister, C., Mauck, W.M., Hao, Y., Stoeckius, M., Smibert, P., and Satija, R. (2019). Comprehensive Integration of Single-Cell Data. Cell *177*, 1888–1902.e21.

Subramanian, A., Tamayo, P., Mootha, V.K., Mukherjee, S., Ebert, B.L., Gillette, M.A., Paulovich, A., Pomeroy, S.L., Golub, T.R., Lander, E.S., et al. (2005). Gene set enrichment analysis: a knowledge-based approach for interpreting genome-wide expression profiles. P Natl Acad Sci Usa *102*, 15545–15550.

Suciu-Foca, N., and Cortesini, R. (2007). Central role of ILT3 in the T suppressor cell cascade. Cell. Immunol. *248*, 59–67.

Tabula Muris Consortium (2018). Single-cell transcriptomics of 20 mouse organs creates a Tabula Muris. Nature *562*, 367–372.

Tabula Muris Consortium (2020). A single-cell transcriptomic atlas characterizes ageing tissues in the mouse. Nature *583*, 590–595.

Theocharis, A.D., Skandalis, S.S., Gialeli, C., and Karamanos, N.K. (2016). Extracellular matrix structure. Adv. Drug Deliv. Rev. *97*, 4–27.

Tsai, M.-S., Bogart, D.F., Li, P., Mehmi, I., and Lupu, R. (2002). Expression and regulation of Cyr61 in human breast cancer cell lines. Oncogene *21*, 964–973.

Tsubura, A., Shikata, N., Inui, T., Morii, S., Hatano, T., Oikawa, T., and Matsuzawa, A. (1988). Immunohistochemical localization of myoepithelial cells and basement membrane in normal, benign and malignant human breast lesions. Virchows Arch a Pathol Anat Histopathol *413*, 133–139.

Tynan, S., Pacia, E., Haynes-Johnson, D., Lawrence, D., D'Andrea, M.R., Guo, J.-Z., Lundeen, S., and Allan, G. (2005). The putative tumor suppressor deleted in malignant brain tumors 1 is an estrogen-regulated gene in rodent and primate endometrial epithelium. Endocrinology *146*, 1066–1073.

Unsworth, A., Anderson, R., and Britt, K. (2014). Stromal fibroblasts and the immune microenvironment: partners in mammary gland biology and pathology? J Mammary Gland Biol *19*, 169–182.

Van den Berge, K., Perraudeau, F., Soneson, C., Love, M.I., Risso, D., Vert, J.-P., Robinson, M.D., Dudoit, S., and Clement, L. (2018). Observation weights unlock bulk RNA-seq tools for zero inflation and single-cell applications. Genome Biol *19*, 24–17.

Van Keymeulen, A., Fioramonti, M., Centonze, A., Bouvencourt, G., Achouri, Y., and Blanpain, C. (2017). Lineage-Restricted Mammary Stem Cells Sustain the Development, Homeostasis, and Regeneration of the Estrogen Receptor Positive Lineage. Cell Rep *20*, 1525–1532.

Vaughan, A.E., Brumwell, A.N., Xi, Y., Gotts, J.E., Brownfield, D.G., Treutlein, B., Tan, K., Tan, V., Liu, F.C., Looney, M.R., et al. (2015). Lineage-negative progenitors mobilize to regenerate lung epithelium after major injury. Nature *517*, 621–625.

Wahl, G.M., and Spike, B.T. (2017). Cell state plasticity, stem cells, EMT, and the generation of intra-tumoral heterogeneity. Npj Breast Cancer *3*, 14–13.

Wahle, E., and Winkler, G.S. (2013). RNA decay machines: deadenylation by the Ccr4-not and Pan2-Pan3 complexes. Biochim Biophys Acta *1829*, 561–570.

Wang, C., Christin, J.R., Oktay, M.H., and Guo, W. (2017). Lineage-Biased Stem Cells Maintain Estrogen-Receptor-Positive and -Negative Mouse Mammary Luminal Lineages. Cell Rep *18*, 2825–2835.

Wang, Y., Chaffee, T.S., LaRue, R.S., Huggins, D.N., Witschen, P.M., Ibrahim, A.M., Nelson, A.C., Machado, H.L., and Schwertfeger, K.L. (2020). Tissue resident macrophages promote extracellular matrix homeostasis in the mammary gland stroma of nulliparous mice. Elife *9*, 1399.

Warburton, M.J., Mitchell, D., Ormerod, E.J., and Rudland, P. (1982). Distribution of myoepithelial cells and basement membrane proteins in the resting, pregnant, lactating, and involuting rat mammary gland. J Histochem Cytochem *30*, 667–676.
Warburton, M.J., Ormerod, E.J., Monaghan, P., Ferns, S., and Rudland, P.S. (1981). Characterization of a myoepithelial cell line derived from a neonatal rat mammary gland. J Cell Biol *91*, 827–836.
Wolock, S.L., and Klein, A.M. (2019). Scrublet: Computational Identification of Cell Doublets in Single-Cell Transcriptomic Data. Cell Syst *8*, 281–291.e289.
Wuidart, A., Sifrim, A., Fioramonti, M., Matsumura, S., Brisebarre, A., Brown, D., Centonze, A., Dannau, A., Dubois, C., Van Keymeulen, A., et al. (2018). Early lineage segregation of multipotent embryonic mammary gland progenitors. Nat Cell Biol *20*, 666–676.
Yamazaki, T., Akiba, H., Iwai, H., Matsuda, H., Aoki, M., Tanno, Y., Shin, T., Tsuchiya, H., Pardoll, D.M., Okumura, K., et al. (2002). Expression of programmed death 1 ligands by murine T cells and APC. J Immunol *169*, 5538–5545.
Yue, Y., Liu, J., Cui, X., Cao, J., Luo, G., Zhang, Z., Cheng, T., Gao, M., Shu, X., Ma, H., et al. (2018). VIRMA mediates preferential m6A mRNA methylation in 3'UTR and near stop codon and associates with alternative polyadenylation. Cell Discov *4*, 10–17.
Zhang, S., Yang, X., Wang, L., and Zhang, C. (2018a). Interplay between inflammatory tumor microenvironment and cancer stem cells. Oncol Lett *16*, 679–686.
Zhang, X., Qiao, G., and Lu, P. (2014). Modulation of fibroblast growth factor signaling is essential for mammary epithelial morphogenesis. PLoS ONE *9*, e92735.
Zhang, Y., Zhang, S., Guan, W., Chen, F., Cheng, L., Lv, Y., and Chen, J. (2018b). GLUT1 and lactose synthetase are critical genes for lactose synthesis in lactating sows. Nutr Metab (Lond) *15*, 40–13.
Zheng, G., Dahl, J.A., Niu, Y., Fedorcsak, P., Huang, C.-M., Li, C.J., Vågbø, C.B., Shi, Y., Wang, W.-L., Song, S.-H., et al. (2013). ALKBH5 is a mammalian RNA demethylase that impacts RNA metabolism and mouse fertility. Mol Cell *49*, 18–29.
Zhu, W., and Nelson, C.M. (2013). PI3K regulates branch initiation and extension of cultured mammary epithelia via Akt and Rac1 respectively. Dev Biol *379*, 235–245.

KEY RESOURCES TABLE

REAGENT or RESOURCE	SOURCE	IDENTIFIER
Antibodies		
ACTA2/SMA	Dako	Cat#M0851;<br>RRID: AB_2223500
ARG1	Cell Signaling Technology	Cat#66297;<br>RRID: AB_2799705
CD163	Abcam	Cat#ab182422;<br>RRID: AB_2753196
CD8α	Cell Signaling Technology	Cat#98941;<br>RRID: AB_2756376
KRT15	BioLegend	Cat#833904;<br>RRID: AB_2616894
KRT14	BioLegend	Cat#905301;<br>RRID: AB_2565048
KRT14	Abcam	Cat#ab206100;<br>RRID: AB_2811031
KRT8	DSHB	Cat#Troma-I;<br>RRID: AB_531826
KRT8	Abcam	Cat#ab192467;<br>RRID: AB_2864346
PDGFRα	Cell Signaling Technology	Cat#3174;<br>RRID: AB_2162345
PR	Cell Signaling Technology	Cat#8757;<br>RRID: AB_2797144
PR	Dako	Cat#A0098;<br>RRID: AB_2315192
ER	Santa Cruz	Cat#sc-542;<br>RRID: AB_631470
ER	BioRad	Cat#MCA1799T;<br>RRID: AB_2102069
LTF	Bioss	Cat#bs-5810;<br>RRID: AB_11078952
MFGE8	Thermo	Cat#MA5-23913;<br>RRID: AB_2609198
MFGE8	R&D Systems	Cat#MAB2805;<br>RRID: AB_2297564
CD45-FITC	BioLegend	Cat#103108;<br>RRID: AB_312973
CD31-FITC	BioLegend	Cat#102405;<br>RRID: AB_312900
TER119-FITC	BioLegend	Cat#116205;<br>RRID: AB_313706
EPCAM-BV605	BioLegend	Cat#118227;<br>RRID: AB_2563984
CD133-APC	BioLegend	Cat#141207;<br>RRID: AB_10898121
CD14-PE	BioLegend	Cat#150106;

		RRID: AB_2728189
Critical Commercial Assays		
Chromium Single Cell 3' Assay	10x Genomics	N/A
Deposited Data		
scRNA-seq data	This study	GSE150580
Experimental Models: Organisms/Strains		
Mouse: mixed FVB, 129, C57BL/6J background	In house	N/A
Software and Algorithms		
R (3.5.1)	R project	RRID: SCR_001905
Python (2.7, python 3.7)	Python Software Foundation	RRID:SCR_008394
Cell Ranger v2	10x Genomics	RRID:SCR_017344
Seurat v3	Butler et al., 2018; Stuart et al., 2019	RRID:SCR_016341
Scrublet	Wolock and Klein, 2019	RRID:SCR_018098
DoubletFinder	McGinnis et al., 2019	RRID:SCR_018771
STREAM	Chen et al., 2019	N/A
zinger-edgeR	Van den Berge et al., 2017	N/A
NCBI Homologene	http://www.ncbi.nlm.nih.gov/homologene	RRID:SCR_002924
MSigDB v7.0	Liberzon et al., 2015; Subramanian et al., 2005	RRID:SCR_016863
Fiji	Schindelin et al., 2012	RRID:SCR_002285
FlowJo v10	BD Biosciences	RRID:SCR_008520
Prism v8	Graphpad software	RRID:SCR_002798

Figure 1

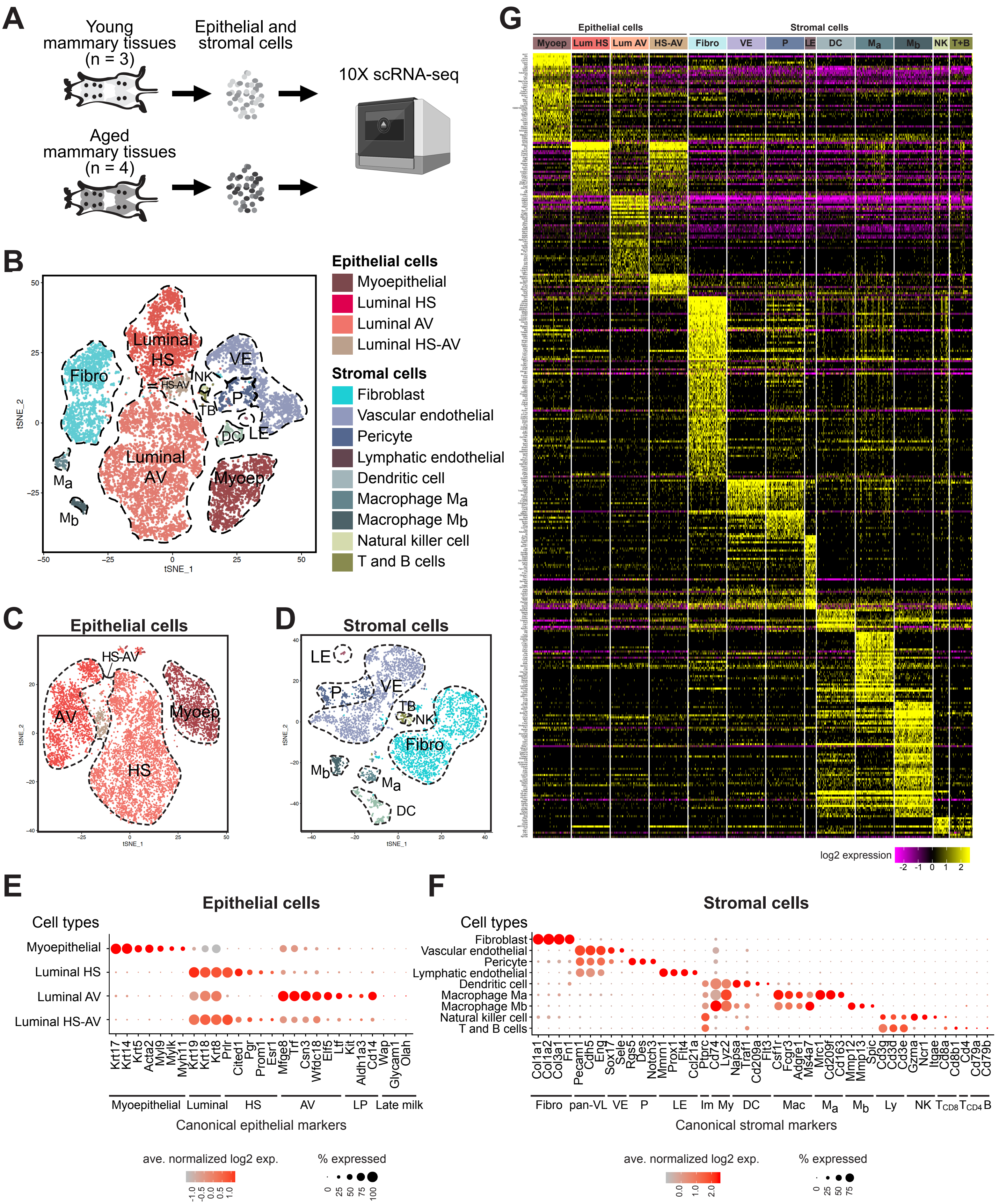
A
Young mammary tissues (n = 3)
Aged mammary tissues (n = 4)
Epithelial and stromal cells
10X scRNA-seq
B
Epithelial cells
Myoepithelial
Luminal HS
Luminal AV
Luminal HS-AV
Stromal cells
Fibroblast
Vascular endothelial
Pericyte
Lymphatic endothelial
Dendritic cell
Macrophage Ma
Macrophage Mb
Natural killer cell
T and B cells
tSNE_1
tSNE_2
C
Epithelial cells
D
Stromal cells
G
Epithelial cells
Stromal cells
Myoep
Lum HS
Lum AV
HS-AV
Fibro
VE
P
LE
DC
NK
T+B
log2 expression
E
Epithelial cells
Cell types
Myoepithelial
Luminal HS
Luminal AV
Luminal HS-AV
Krt17 Krt14 Krt5 Acta2 Myl9 Mylk Myh11 Krt19 Krt18 Krt8 Prlr Cited1 Pgr Prom1 Esr1 Mfge8 Trf Csn3 Wfdc18 Elf5 Ltf Kit Aldh1a3 Cd14 Wap Glycam1 Olah
Myoepithelial Luminal HS AV LP Late milk
Canonical epithelial markers
ave. normalized log2 exp.
% expressed
F
Stromal cells
Cell types
Fibroblast
Vascular endothelial
Pericyte
Lymphatic endothelial
Dendritic cell
Macrophage Ma
Macrophage Mb
Natural killer cell
T and B cells
Col1a1 Col1a2 Col3a1 Fn1 Pecam1 Cdh5 Eng Sox17 Sele Rgs5 Des Notch3 Mmrn1 Prox1 Flt4 Ccl21a Ptprc Cd74 Lyz2 Napsa Traf1 Cd209a Flt3 Csf1r Fcgr3 Adgre1 Ms4a7 Mrc1 Cd209f Cd163 Mmp12 Mmp13 Spic Cd3g Cd3d Cd3e Gzma Ncr1 Itgae Cd8a Cd8b1 Cd4 Cd79a Cd79b
Fibro pan-VL VE P LE Im My DC Mac Ma Mb Ly NK TCD8 TCD4 B
Canonical stromal markers
ave. normalized log2 exp.
% expressed

Figure 2

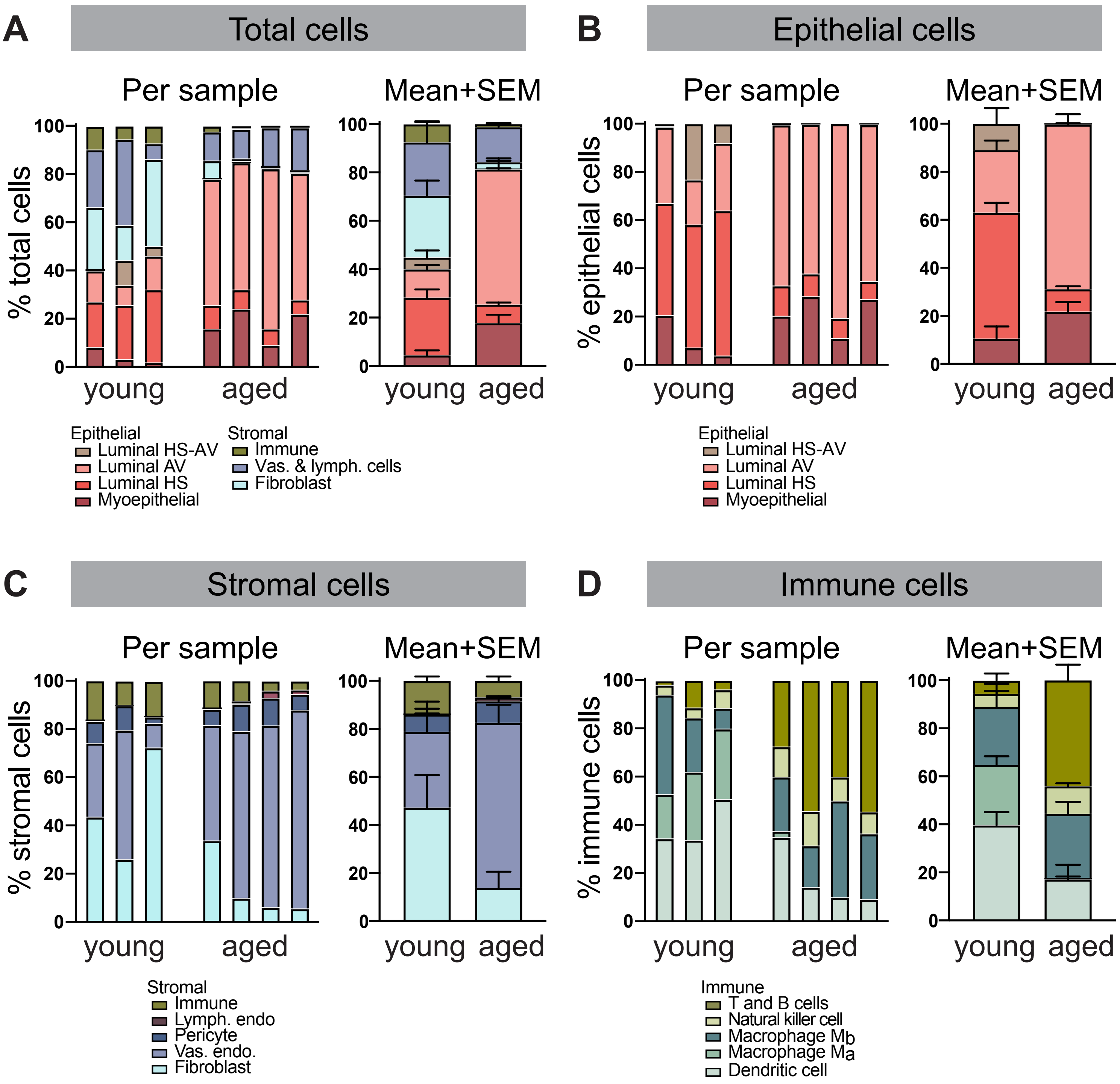
A
Total cells
Per sample
Mean+SEM
% total cells
young
aged
Epithelial
Luminal HS-AV
Luminal AV
Luminal HS
Myoepithelial
Stromal
Immune
Vas. & lymph. cells
Fibroblast
B
Epithelial cells
Per sample
Mean+SEM
% epithelial cells
young
aged
Epithelial
Luminal HS-AV
Luminal AV
Luminal HS
Myoepithelial
C
Stromal cells
Per sample
Mean+SEM
% stromal cells
young
aged
Stromal
Immune
Lymph. endo
Pericyte
Vas. endo.
Fibroblast
D
Immune cells
Per sample
Mean+SEM
% immune cells
young
aged
Immune
T and B cells
Natural killer cell
Macrophage Mb
Macrophage Ma
Dendritic cell

Figure 3

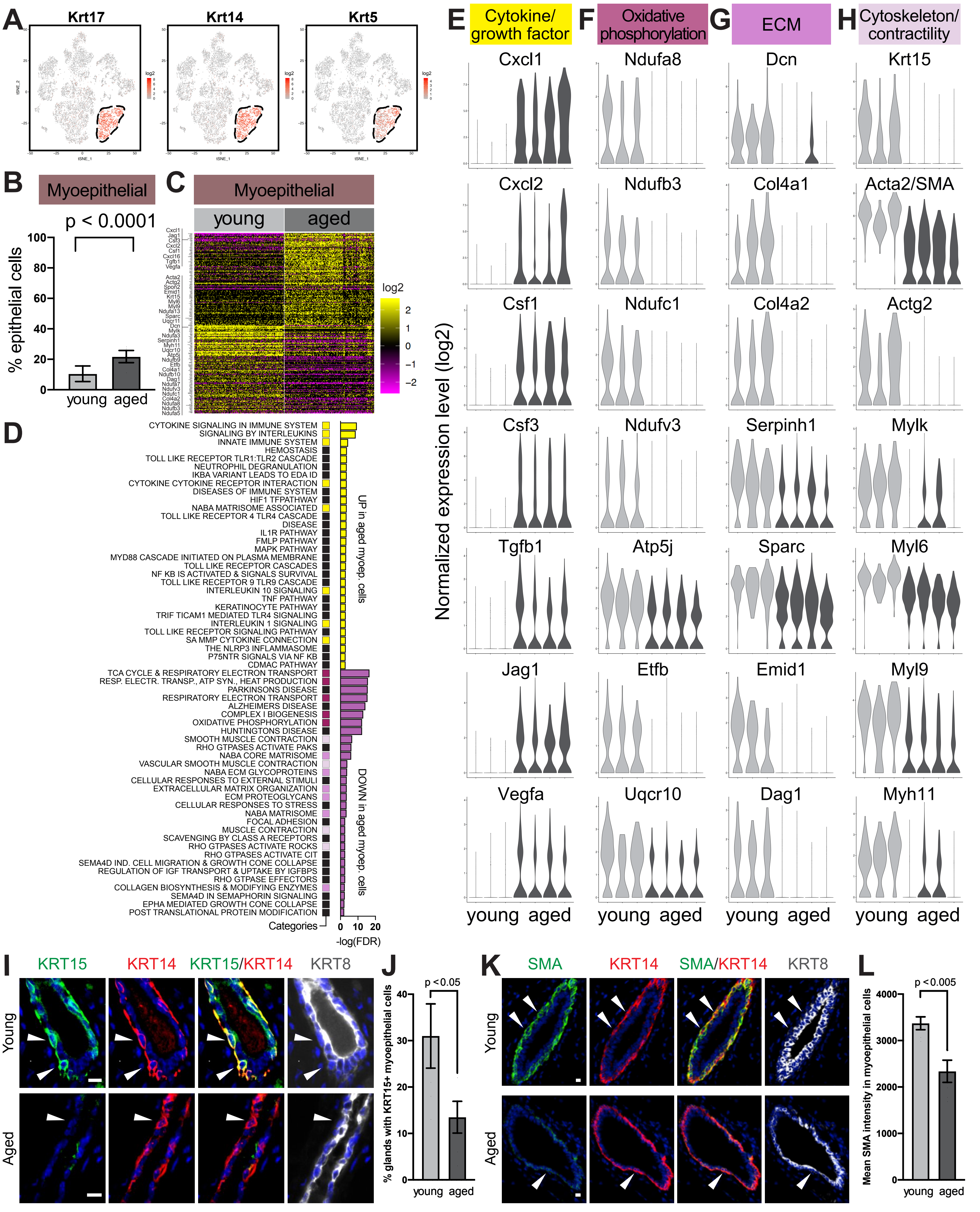
A
Krt17
Krt14
Krt5
tSNE_1
tSNE_2
log2
B
Myoepithelial
p < 0.0001
% epithelial cells
young
aged
C
Myoepithelial
young
aged
log2
Cxcl1
Jag1
Csf3
Cxcl2
Csf1
Cxcl16
Tgfb1
Vegfa
Acta2
Actg2
Spon2
Emid1
Krt15
Myl6
Myl9
Ndufa13
Sparc
Uqcr11
Dcn
Mylk
Ndufa3
Serpinh1
Myh11
Uqcr10
Atp5j
Ndufb9
Etfb
Col4a1
Ndufb10
Dag1
Ndufa7
Ndufv3
Ndufc1
Col4a2
Ndufa8
Ndufb3
Ndufa5
D
CYTOKINE SIGNALING IN IMMUNE SYSTEM
SIGNALING BY INTERLEUKINS
INNATE IMMUNE SYSTEM
HEMOSTASIS
TOLL LIKE RECEPTOR TLR1:TLR2 CASCADE
NEUTROPHIL DEGRANULATION
IKBA VARIANT LEADS TO EDA ID
CYTOKINE CYTOKINE RECEPTOR INTERACTION
DISEASES OF IMMUNE SYSTEM
HIF1 TFPATHWAY
NABA MATRISOME ASSOCIATED
TOLL LIKE RECEPTOR 4 TLR4 CASCADE
DISEASE
IL1R PATHWAY
FMLP PATHWAY
MAPK PATHWAY
MYD88 CASCADE INITIATED ON PLASMA MEMBRANE
TOLL LIKE RECEPTOR CASCADES
NF KB IS ACTIVATED & SIGNALS SURVIVAL
TOLL LIKE RECEPTOR 9 TLR9 CASCADE
INTERLEUKIN 10 SIGNALING
TNF PATHWAY
KERATINOCYTE PATHWAY
TRIF TICAM1 MEDIATED TLR4 SIGNALING
INTERLEUKIN 1 SIGNALING
TOLL LIKE RECEPTOR SIGNALING PATHWAY
SA MMP CYTOKINE CONNECTION
THE NLRP3 INFLAMMASOME
P75NTR SIGNALS VIA NF KB
CDMAC PATHWAY
TCA CYCLE & RESPIRATORY ELECTRON TRANSPORT
RESP. ELECTR. TRANSP., ATP SYN., HEAT PRODUCTION
PARKINSONS DISEASE
RESPIRATORY ELECTRON TRANSPORT
ALZHEIMERS DISEASE
COMPLEX I BIOGENESIS
OXIDATIVE PHOSPHORYLATION
HUNTINGTONS DISEASE
SMOOTH MUSCLE CONTRACTION
RHO GTPASES ACTIVATE PAKS
NABA CORE MATRISOME
VASCULAR SMOOTH MUSCLE CONTRACTION
NABA ECM GLYCOPROTEINS
CELLULAR RESPONSES TO EXTERNAL STIMULI
EXTRACELLULAR MATRIX ORGANIZATION
ECM PROTEOGLYCANS
CELLULAR RESPONSES TO STRESS
NABA MATRISOME
FOCAL ADHESION
MUSCLE CONTRACTION
SCAVENGING BY CLASS A RECEPTORS
RHO GTPASES ACTIVATE ROCKS
RHO GTPASES ACTIVATE CIT
SEMA4D IND. CELL MIGRATION & GROWTH CONE COLLAPSE
REGULATION OF IGF TRANSPORT & UPTAKE BY IGFBPS
RHO GTPASE EFFECTORS
COLLAGEN BIOSYNTHESIS & MODIFYING ENZYMES
SEMA4D IN SEMAPHORIN SIGNALING
EPHA MEDIATED GROWTH CONE COLLAPSE
POST TRANSLATIONAL PROTEIN MODIFICATION
Categories
-log(FDR)
UP in aged myoep. cells
DOWN in aged myoep. cells
E
Cytokine/ growth factor
F
Oxidative phosphorylation
G
ECM
H
Cytoskeleton/ contractility
Normalized expression level (log2)
Cxcl1
Ndufa8
Dcn
Krt15
Cxcl2
Ndufb3
Col4a1
Acta2/SMA
Csf1
Ndufc1
Col4a2
Actg2
Csf3
Ndufv3
Serpinh1
Mylk
Tgfb1
Atp5j
Sparc
Myl6
Jag1
Etfb
Emid1
Myl9
Vegfa
Uqcr10
Dag1
Myh11
young aged
I
KRT15
KRT14
KRT15/KRT14
KRT8
Young
Aged
J
p < 0.05
% glands with KRT15+ myoepithelial cells
young
aged
K
SMA
KRT14
SMA/KRT14
KRT8
Young
Aged
L
p < 0.005
Mean SMA intensity in myoepithelial cells
young
aged

Figure 4

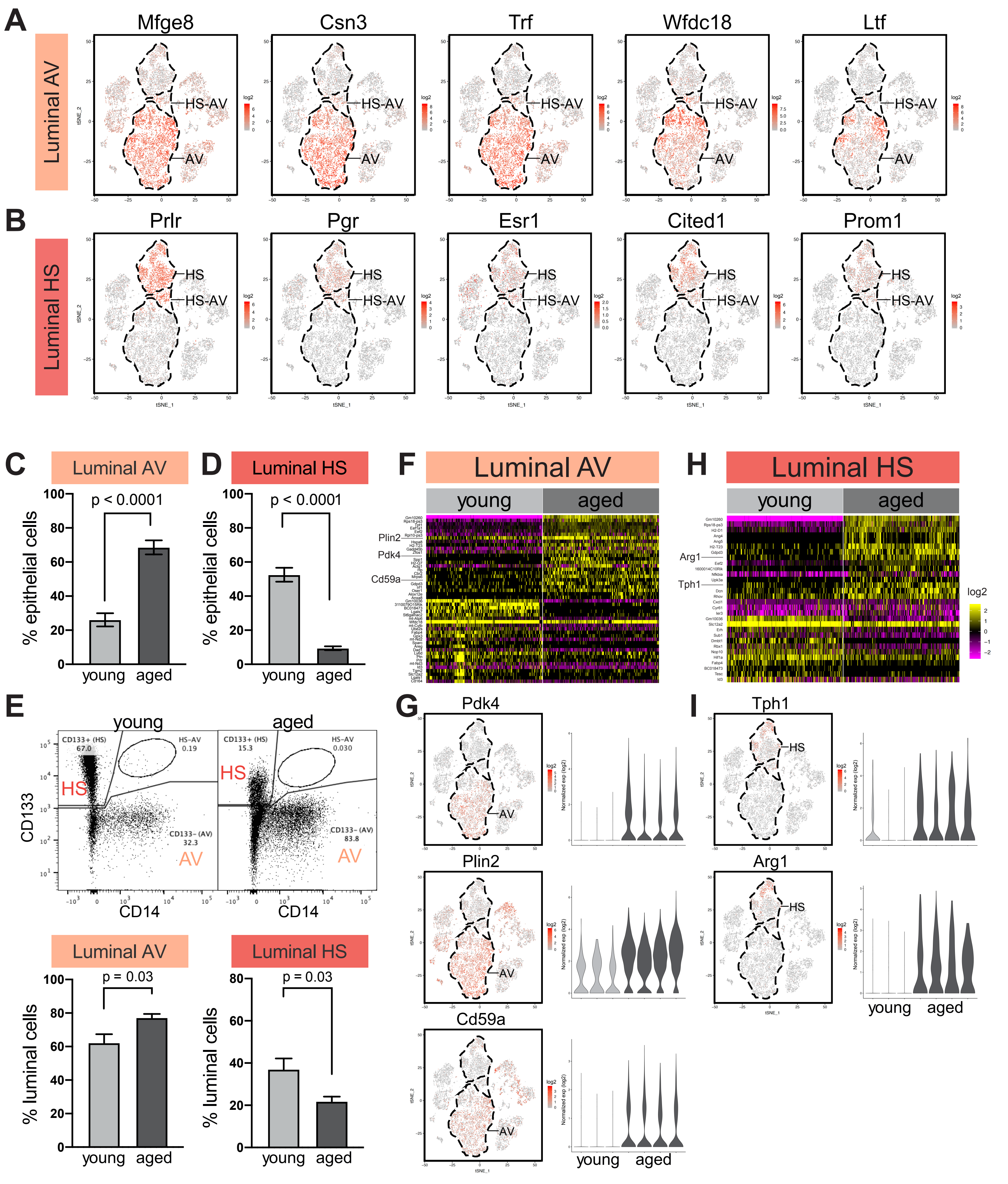
A
Luminal AV
Mfge8
Csn3
Trf
Wfdc18
Ltf
HS-AV
AV
B
Luminal HS
Prlr
Pgr
Esr1
Cited1
Prom1
HS
HS-AV
C
Luminal AV
p < 0.0001
% epithelial cells
young
aged
D
Luminal HS
p < 0.0001
% epithelial cells
young
aged
F
Luminal AV
young
aged
Plin2
Pdk4
Cd59a
H
Luminal HS
young
aged
Arg1
Tph1
log2
E
young
aged
CD133+ (HS)
67.0
HS-AV
0.19
CD133- (AV)
32.3
CD133+ (HS)
15.3
HS-AV
0.030
CD133- (AV)
83.8
HS
AV
CD133
CD14
Luminal AV
p = 0.03
% luminal cells
young
aged
Luminal HS
p = 0.03
% luminal cells
young
aged
G
Pdk4
Plin2
Cd59a
AV
young
aged
I
Tph1
Arg1
HS
young
aged

Figure 5

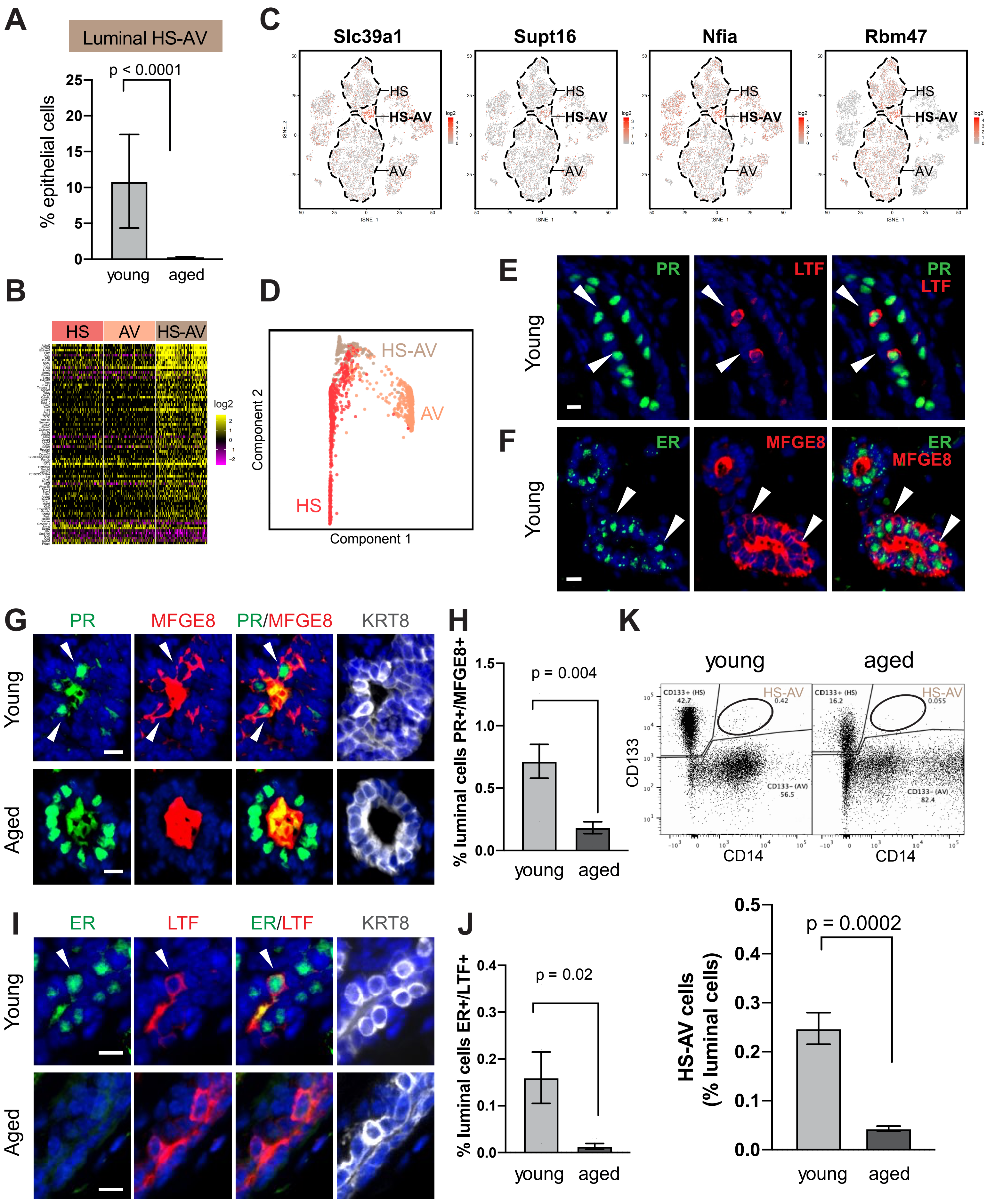
A
Luminal HS-AV
p < 0.0001
% epithelial cells
young
aged
B
HS
AV
HS-AV
log2
C
Slc39a1
Supt16
Nfia
Rbm47
HS
HS-AV
AV
tSNE_1
tSNE_2
D
HS-AV
AV
HS
Component 1
Component 2
E
Young
PR
LTF
F
ER
MFGE8
G
PR
MFGE8
PR/MFGE8
KRT8
Young
Aged
H
p = 0.004
% luminal cells PR+/MFGE8+
young
aged
K
young
aged
CD133+ (HS)
42.7
16.2
HS-AV
0.42
0.055
CD133- (AV)
56.5
82.4
CD133
CD14
p = 0.0002
HS-AV cells
(% luminal cells)
I
ER
LTF
ER/LTF
KRT8
J
p = 0.02
% luminal cells ER+/LTF+

Figure 6

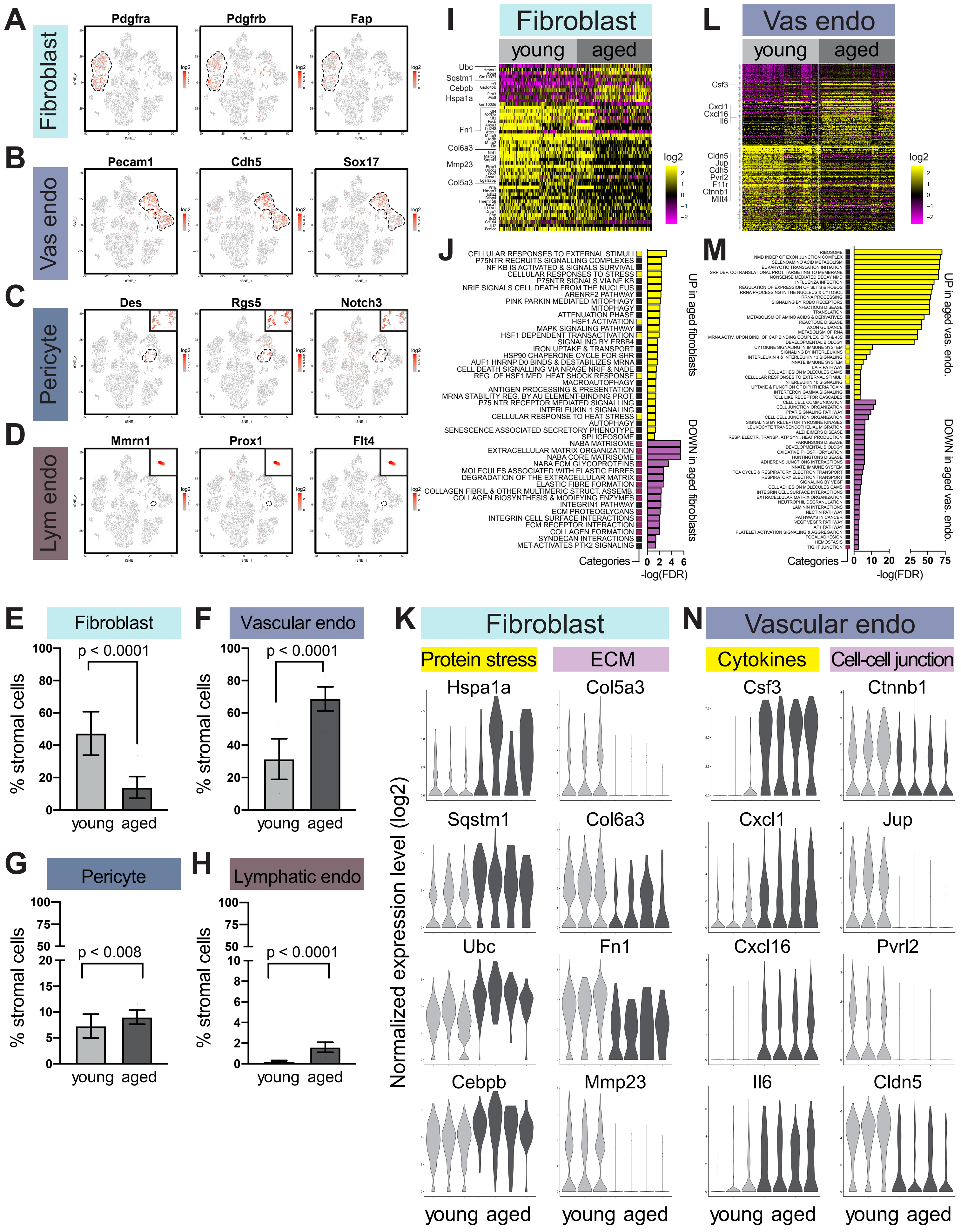
A
Fibroblast
Pdgfra
Pdgfrb
Fap
B
Vas endo
Pecam1
Cdh5
Sox17
C
Pericyte
Des
Rgs5
Notch3
D
Lym endo
Mmrn1
Prox1
Flt4
log2
tSNE_1
tSNE_2
E
Fibroblast
p < 0.0001
% stromal cells
young
aged
F
Vascular endo
p < 0.0001
% stromal cells
young
aged
G
Pericyte
p < 0.008
% stromal cells
young
aged
H
Lymphatic endo
p < 0.0001
% stromal cells
young
aged
I
Fibroblast
young
aged
Ubc
Sqstm1
Cebpb
Hspa1a
Fn1
Col6a3
Mmp23
Col5a3
log2
J
CELLULAR RESPONSES TO EXTERNAL STIMULI
P75NTR RECRUITS SIGNALLING COMPLEXES
NF KB IS ACTIVATED & SIGNALS SURVIVAL
CELLULAR RESPONSES TO STRESS
P75NTR SIGNALS VIA NF KB
NRIF SIGNALS CELL DEATH FROM THE NUCLEUS
ARENRF2 PATHWAY
PINK PARKIN MEDIATED MITOPHAGY
MITOPHAGY
ATTENUATION PHASE
HSF1 ACTIVATION
MAPK SIGNALING PATHWAY
HSF1 DEPENDENT TRANSACTIVATION
SIGNALING BY ERBB4
IRON UPTAKE & TRANSPORT
HSP90 CHAPERONE CYCLE FOR SHR
AUF1 HNRNP D0 BINDS & DESTABILIZES MRNA
CELL DEATH SIGNALLING VIA NRAGE NRIF & NADE
REG. OF HSF1 MED. HEAT SHOCK RESPONSE
MACROAUTOPHAGY
ANTIGEN PROCESSING & PRESENTATION
MRNA STABILITY REG. BY AU ELEMENT-BINDING PROT.
P75 NTR RECEPTOR MEDIATED SIGNALLING
INTERLEUKIN 1 SIGNALING
CELLULAR RESPONSE TO HEAT STRESS
AUTOPHAGY
SENESCENCE ASSOCIATED SECRETORY PHENOTYPE
SPLICEOSOME
NABA MATRISOME
EXTRACELLULAR MATRIX ORGANIZATION
NABA CORE MATRISOME
NABA ECM GLYCOPROTEINS
MOLECULES ASSOCIATED WITH ELASTIC FIBRES
DEGRADATION OF THE EXTRACELLULAR MATRIX
ELASTIC FIBRE FORMATION
COLLAGEN FIBRIL & OTHER MULTIMERIC STRUCT. ASSEMB.
COLLAGEN BIOSYNTHESIS & MODIFYING ENZYMES
INTEGRIN1 PATHWAY
ECM PROTEOGLYCANS
INTEGRIN CELL SURFACE INTERACTIONS
ECM RECEPTOR INTERACTION
COLLAGEN FORMATION
SYNDECAN INTERACTIONS
MET ACTIVATES PTK2 SIGNALING
Categories
-log(FDR)
UP in aged fibroblasts
DOWN in aged fibroblasts
L
Vas endo
young
aged
Csf3
Cxcl1
Cxcl16
Il6
Cldn5
Jup
Cdh5
Pvrl2
F11r
Ctnnb1
Mllt4
log2
M
Categories
-log(FDR)
UP in aged vas. endo.
DOWN in aged vas. endo.
K
Fibroblast
Protein stress
ECM
Hspa1a
Sqstm1
Ubc
Cebpb
Col5a3
Col6a3
Fn1
Mmp23
N
Vascular endo
Cytokines
Cell-cell junction
Csf3
Cxcl1
Cxcl16
Il6
Ctnnb1
Jup
Pvrl2
Cldn5
Normalized expression level (log2)
young
aged

Figure 7

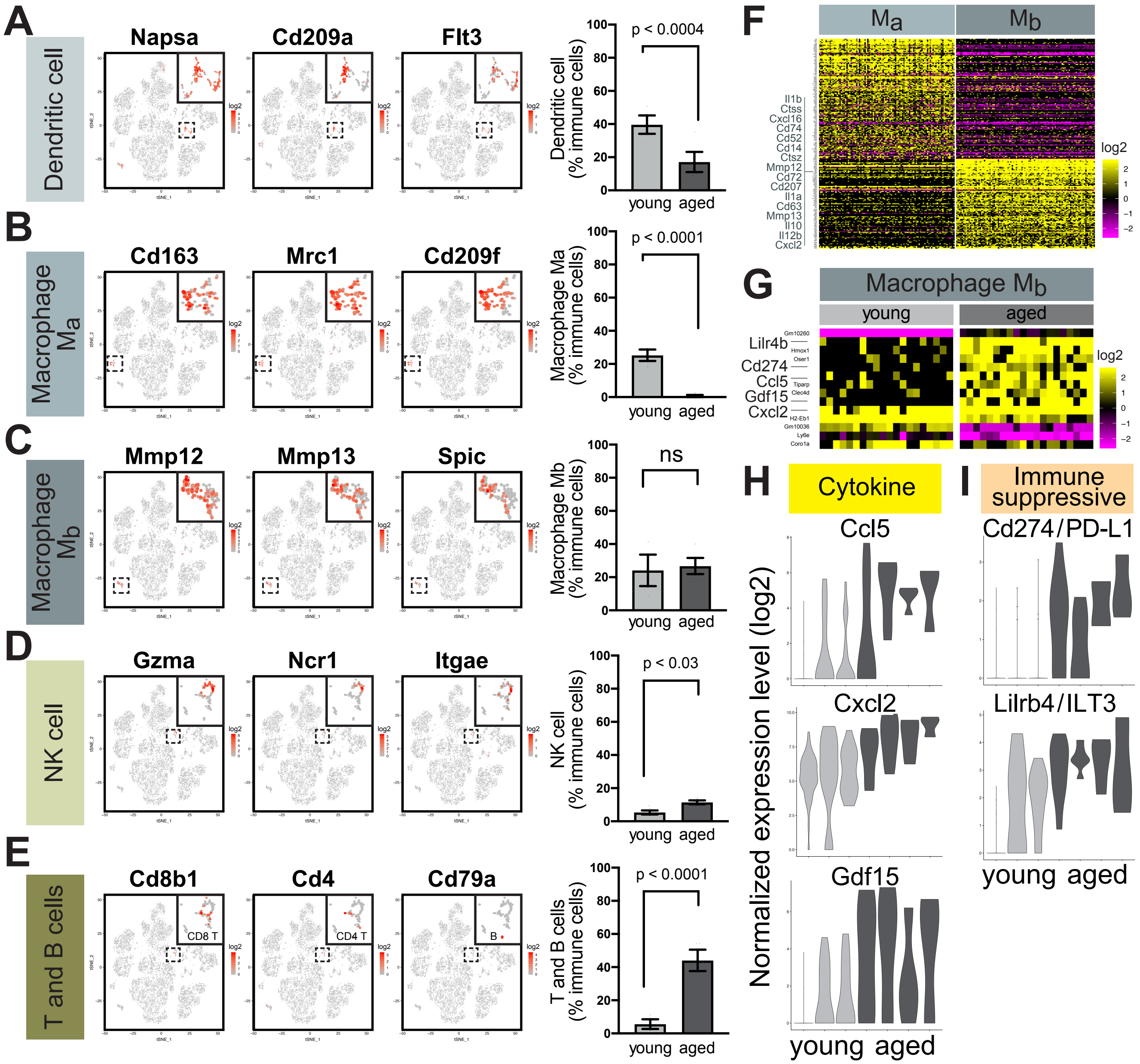
A
Dendritic cell
Napsa
Cd209a
Flt3
Dendritic cell (% immune cells)
p < 0.0004
young
aged
B
Macrophage Ma
Cd163
Mrc1
Cd209f
Macrophage Ma (% immune cells)
p < 0.0001
C
Macrophage Mb
Mmp12
Mmp13
Spic
Macrophage Mb (% immune cells)
ns
D
NK cell
Gzma
Ncr1
Itgae
NK cell (% immune cells)
p < 0.03
E
T and B cells
Cd8b1
Cd4
Cd79a
CD8 T
CD4 T
B
T and B cells (% immune cells)
p < 0.0001
F
Ma
Mb
Il1b
Ctss
Cxcl16
Cd74
Cd52
Cd14
Ctsz
Mmp12
Cd72
Cd207
Il1a
Cd63
Mmp13
Il10
Il12b
Cxcl2
log2
G
Macrophage Mb
young
aged
Lilr4b
Cd274
Ccl5
Gdf15
Cxcl2
log2
H
Cytokine
Ccl5
Cxcl2
Gdf15
I
Immune suppressive
Cd274/PD-L1
Lilrb4/ILT3
Normalized expression level (log2)
young aged

Figure S1

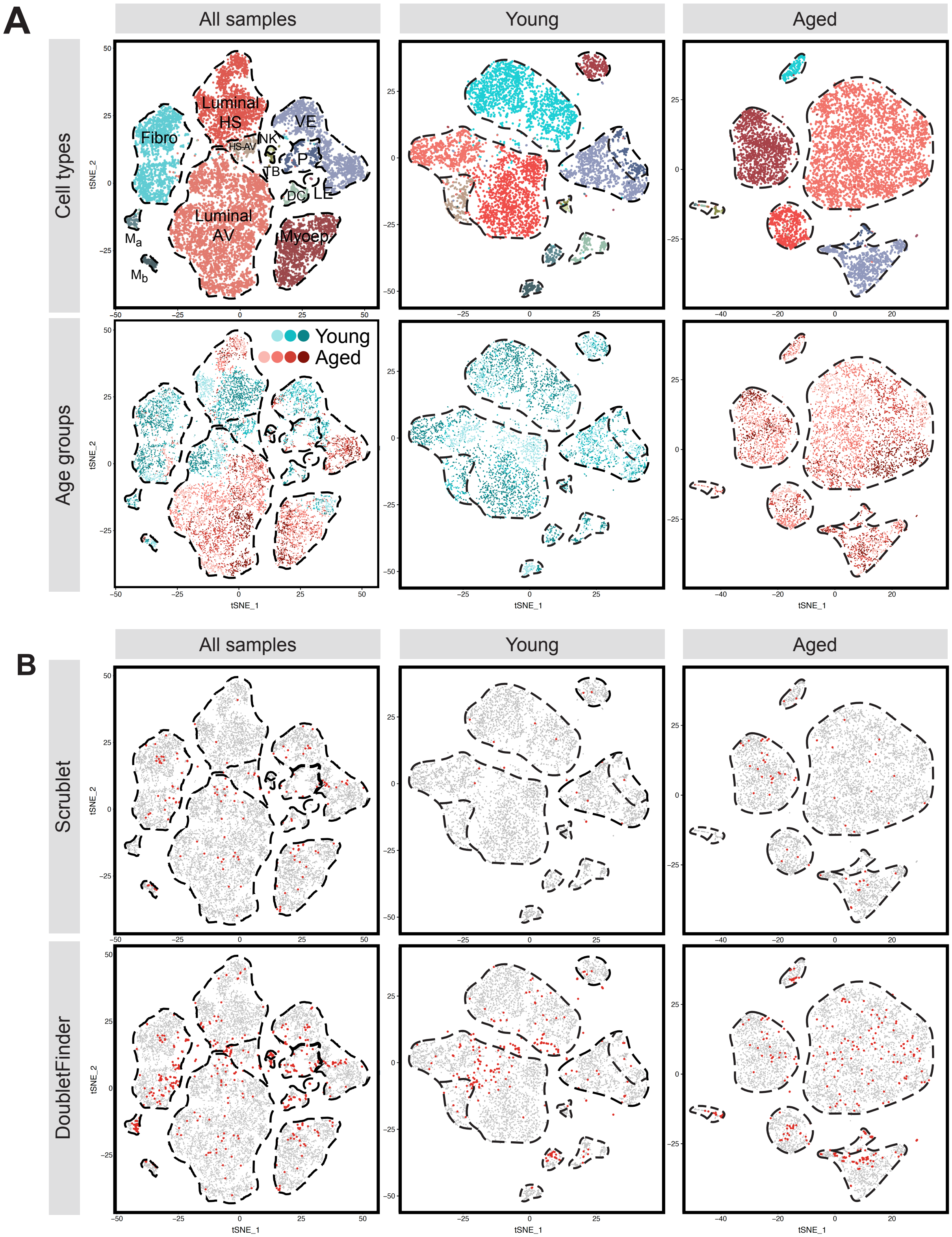
A
All samples
Young
Aged
Cell types
Age groups
Luminal HS
VE
Fibro
NK
HS-AV
P
TB
DC
LE
Luminal AV
Myoep
M_a
M_b
Young
Aged
B
Scrublet
DoubletFinder
tSNE_1
tSNE_2

Figure S1 (related to Figure 1). t-SNE Plots Showing Distribution of Samples and Identification of Potential Doublets

(A) t-SNE plots colored by age groups and by biological replicates.

(B) t-SNE plot showing predicted doublets in scRNA-seq data using Scrublet and DoubletFinder. If a cluster was overwhelmingly enriched with predicted doublets, it would likely represent an artifact doublet population generated by chance inclusion of two or more cells in a single droplet during the cell capturing step on the 10X Chromium platform. No such cluster is detected in our dataset.

Figure S2

A

Chi-square	Young (A)	Young (B)	Young (C)	Aged (A)	Aged (B)	Aged (C)	Aged (D)
**Young (A)**							
**Young (B)**	348						
**Young (C)**	555	729					
**Aged (A)**	926	990	1499				
**Aged (B)**	1718	1629	2669	141			
**Aged (C)**	1281	1179	1834	152	155		
**Aged (D)**	1227	1210	2039	135	32	105	

All p-values < 0.0001; df = 6

B

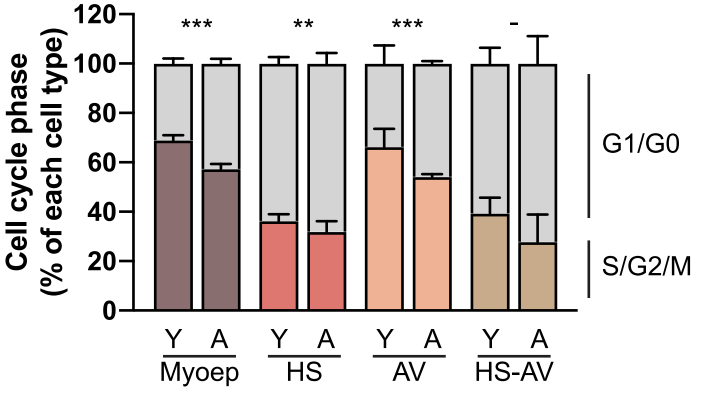

Young

	Myoep	HS	AV	HS-AV
**Myoep**				
**HS**	****			
**AV**	-	****		
**HS-AV**	****	-	****	

Aged

	Myoep	HS	AV	HS-AV
**Myoep**				
**HS**	****			
**AV**	*	****		
**HS-AV**	**	-	*	

Figure S2 (related to Figure 2A). Cell Type Proportions and Cell Cycle Fractions within and across Age Groups

(A) Cell type proportions are consistent within biological replicates and distinct across age groups. Table summarizes Chi-square test results for all per-sample pairwise comparisons of cell type proportions. The lower Chi-square values within each age group reflect consistency among biological replicates, whereas the higher Chi-square values across age groups reflect age-dependent differences.

(B) Bar graph of the fraction of cells in S/G2/M phases (colored bars) or G1/G0 phase (gray bars) within each epithelial cell type. Cell cycle estimation was performed using the default CellCycleScoring function in Seurat, with cells in G2/M/S considered cycling and G1/G0 non-cycling. All values represent mean + SEM. Statistically significant differences between young (Y) and aged (A) groups within a cell type are denoted above the bars (Fisher's exact test). Statistically significant differences between cell types within young mice or within aged mice are denoted in the respective tables (Fisher's exact test). All p-values are denoted by * (< 0.05), ** (<0.01), *** (<0.001), **** (<0.0001), or – (not significant). Within each cell type, n = 3 young mice and n = 4 aged mice were analyzed, except for HS-AV (n = 3+3), where such cells were not detected in one of the four aged mice.

Abbreviations: Myoep, myoepithelial cells; HS, hormone-sensing luminal cells; AV, alveolar luminal cells.

Figure S3

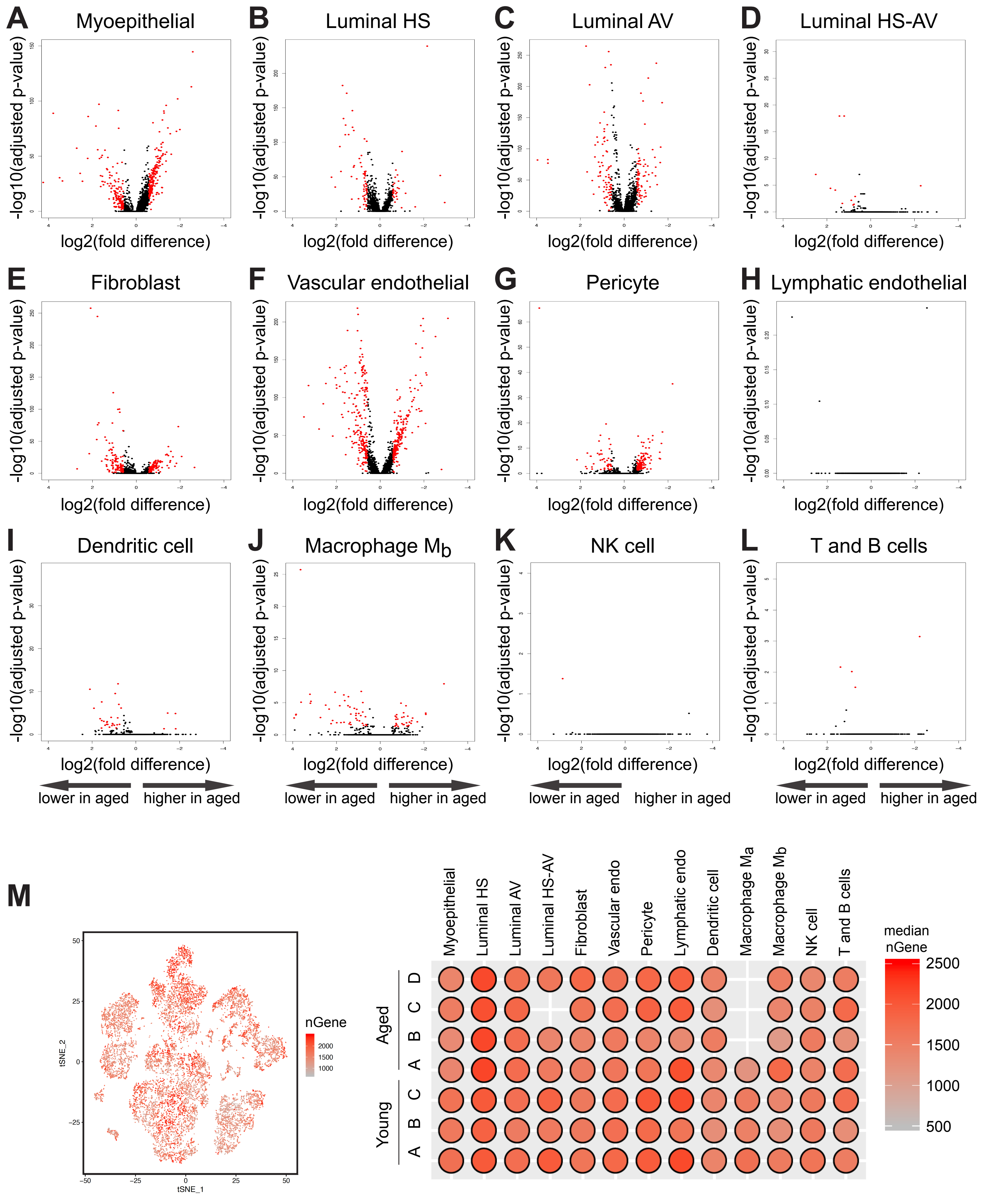
A Myoepithelial
B Luminal HS
C Luminal AV
D Luminal HS-AV
E Fibroblast
F Vascular endothelial
G Pericyte
H Lymphatic endothelial
I Dendritic cell
J Macrophage Mb
K NK cell
L T and B cells
-log10(adjusted p-value)
log2(fold difference)
lower in aged
higher in aged
M
tSNE_1
tSNE_2
nGene
2000
1500
1000
Myoepithelial
Luminal HS
Luminal AV
Luminal HS-AV
Fibroblast
Vascular endo
Pericyte
Lymphatic endo
Dendritic cell
Macrophage Ma
Macrophage Mb
NK cell
T and B cells
Aged
Young
D
C
B
A
median nGene
2500
2000
1500
1000
500

Figure S3 (related to Figures 3, 4, 6, and 7). Prevalent Alterations in Gene Expression in Aged Mammary Glands

(A-L) Volcano plots show expression levels of all detected genes in aged mammary glands (n = 4) compared to young mammary glands (n = 3) within the specified cell types. Genes with fold-difference above 1.5 and with adjusted *p*-value < 0.05 are highlighted in red. Differential expression analysis was not possible for M_a macrophages due to their extremely low abundance in aged mice.

(M) Number of genes (nGene) captured by scRNA-seq per cell (left) and median nGene within each cell type per sample (right). HS-AV cells and M_a macrophages were not detected in one or more aged mice.

Figure S4

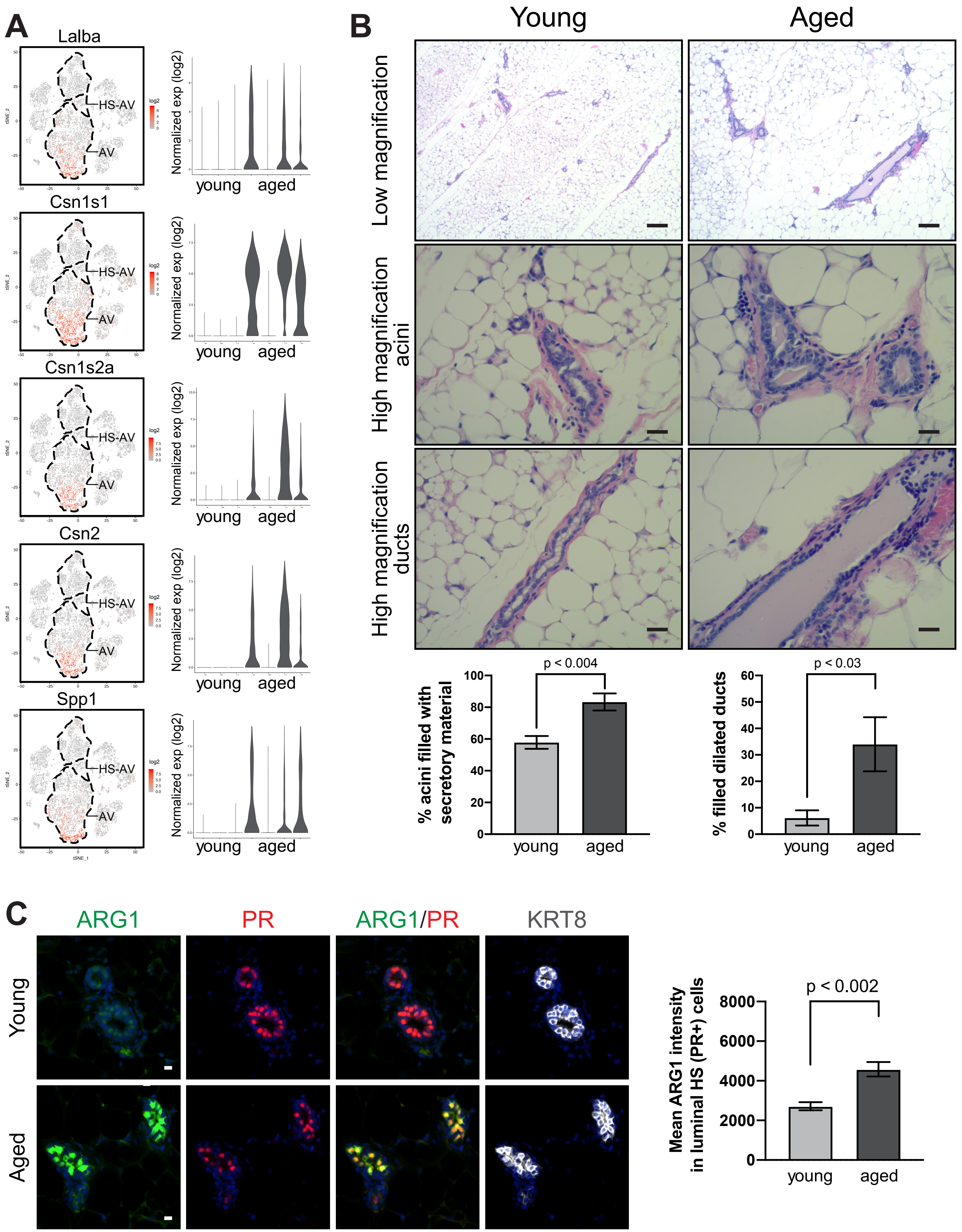
A
Lalba
Csn1s1
Csn1s2a
Csn2
Spp1
HS-AV
AV
Normalized exp (log2)
young
aged
B
Young
Aged
Low magnification
High magnification acini
High magnification ducts
% acini filled with secretory material
p < 0.004
% filled dilated ducts
p < 0.03
C
ARG1
PR
ARG1/PR
KRT8
Mean ARG1 intensity in luminal HS (PR+) cells
p < 0.002

Figure S4 (related to Figure 4). Alveolar and Hormone-Sensing Luminal Cell Changes with Aging
(A) t-SNE plots (left) showing the expression of milk biosynthesis-related genes in a subset of aged AV cells. Violin plots (right) showing that the expression of these genes is upregulated in AV cells in three out of four aged mammary glands compared to young glands.
(B) Representative H&E images and quantification of young and aged mammary acini and ducts. Aged mammary glands show increased secretory material in acini and dilated ducts. H&E at low magnification, scale bar = 100 μm; at high magnification, scale bar = 20 μm. For quantification of acini filled with secretory material, at least 20 acini per gland with diameter < 50 μm were analyzed. For quantification of ducts filled and dilated, at least 10 ducts per gland with cross-section length > 250 μm were analyzed. Ducts were considered dilated if the width:length ratio was > 1:4, i.e. 0.25. Analysis was performed on n = 6 animals per age group using Student's t test.
(C) Representative immunofluorescence staining pattern and quantification of ARG1 expression in luminal HS cells (stained as PR+) in young and aged mammary glands. KRT8 marks luminal cells. Scale bar = 10 μm. For quantification, at least 800 HS cells per mouse were analyzed. Analysis was performed on n = 6 animals per age group using Student's t test.

Figure S5

A

This Study		
Strain	Mixed FVB, 129, C57BL/6J mice	
Parity	Nulliparous	
Estrous staging	Diestrus	
Age	3-4	13-14 months
	n=3	n=4
Cell type	Young	Aged
HS	59%	12%
AV	29%	88%
HS-AV	12%	0.3%
Total luminal cells	100%	100%

Bach et al., 2017	
C57BL/6N mice	
Nulliparous	
Not estrous staged	
2 months	
	n=2
Cell type (orig. name)	Young
HS (HSd)	66%
AV (Lp)	21%
HS-AV (HSp)	13%
Total luminal cells	100%

Pal et al., 2017		
FVB/NJ mice		
Nulliparous		
	Estrus	Diestrus
2.5 months		
	n=2	n=2
Cell type (orig. name)	Young estrus	Young diestrus
HS (Mature luminal)	52%	53%
AV (Luminal progenitor)	33%	42%
HS-AV (Luminal intermediate)	10%	4%
Other 1 (Rare cluster)	1%	1%
Other 2 (Rare cluster)	3%	1%
Total luminal cells	100%	100%

Kanaya et al., 2019				
BALB/cj				
Nulliparous				
Ovariectomy at 9 weeks of age, one-week treatment before analysis				
5 months				
	n=2+2+2	n=2	n=2	n=2
Cell type (orig. name)	All	Vehicle	E2	E2+PBDE
HS (Mature luminal)	49%	28%	51%	53%
AV (Luminal progenitor)	14%	17%	17%	15%
HS-AV (Luminal intermediate)	36%	55%	33%	33%
Total luminal cells	100%	100%	100%	100%

B

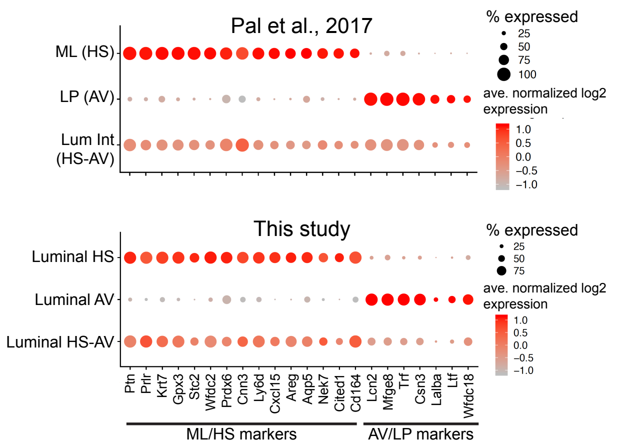

C

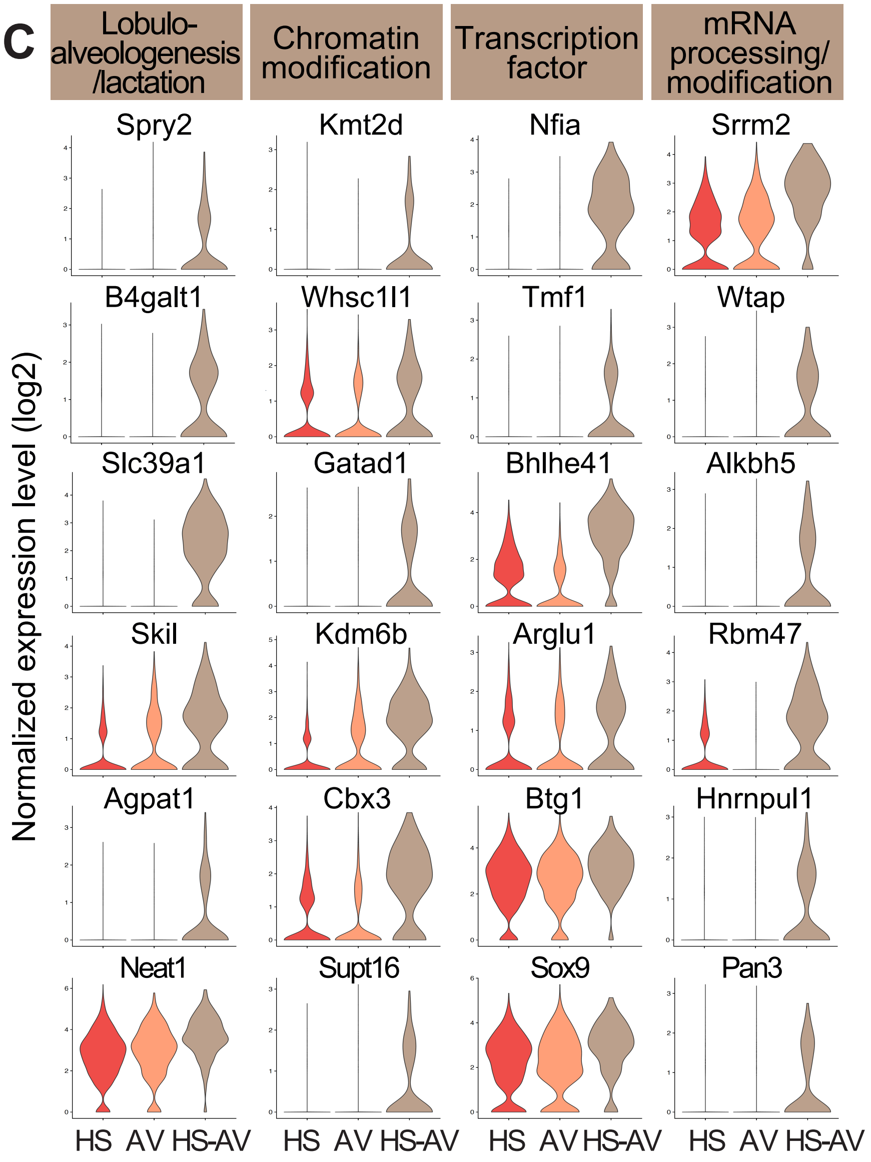

D

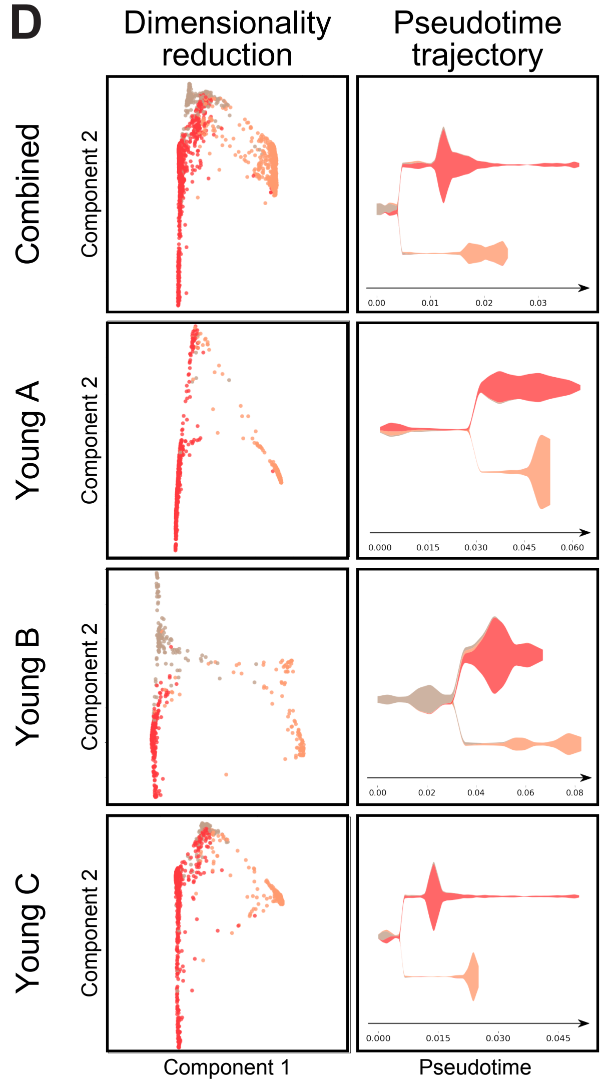

E

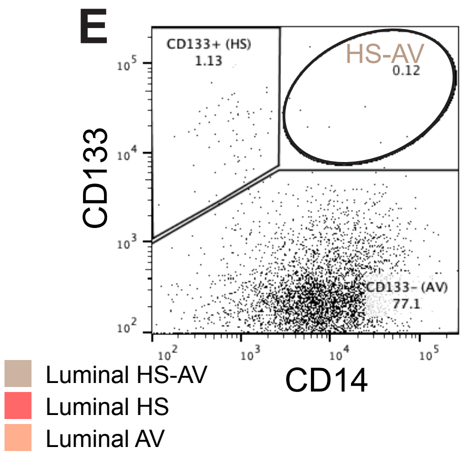

F

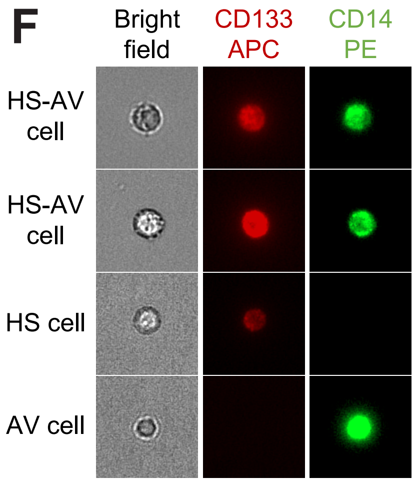

Figure S5 (related to Figure 5). HS-AV Gene Markers and Lineage Trajectory Analysis

(A) Proportions of luminal HS, AV, and HS-AV cells in young mice analyzed in this study compared to equivalent cell populations in other studies of young mice (adapted from Figure 6a in Pal et al., 2017 and Table 1 in Bach et al., 2017) and ovariectomized mice (adapted from Table 2 and Supplementary Figure 10a in Kanaya et al, 2019; E2, 17β-estradiol; PBDE, polybrominated diphenyl ethers).

(B) Expression of select HS and AV markers in Luminal Intermediate cells (Pal et al., 2017) and HS-AV cells (this study). Markers were partially adapted from Figure S4b of Pal et al., 2017.

(C) Violin plots of select genes identified in Figure 5B with expression enriched in HS-AV cells relative to HS cells and AV cells.

(D) Results from lineage trajectory analysis of luminal cells in young mice (n = 3), analyzed either in aggregate or as individual samples. In each case, HS-AV cells are localized to the bifurcation between the HS lineage and AV lineage.

(E) HS-AV cells co-expressing HS marker CD133 and AV marker CD14 are detected by FACS in organoid cultures of primary mammary cells.

(F) Example of HS-AV cells isolated by FACS and examined under the microscope to confirm that they are single cells with co-staining for CD133 and CD14, not doublets composed of CD133+ cell and CD14+ cell. CD133+ HS cells and CD14+ AV cells serve as controls.

Figure S6

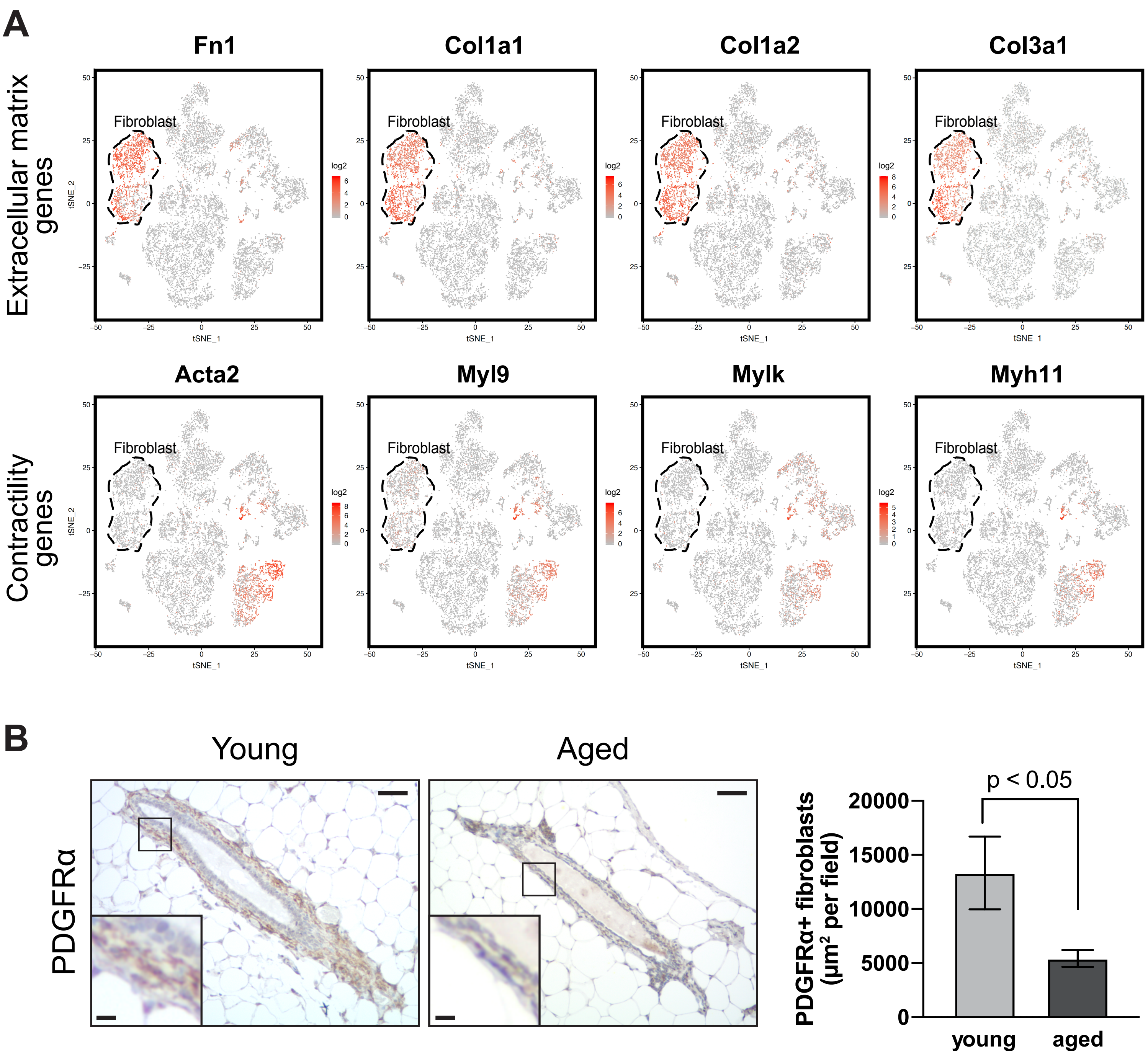
A
Fn1
Col1a1
Col1a2
Col3a1
Extracellular matrix genes
Fibroblast
log2
tSNE_1
tSNE_2
Acta2
Myl9
Mylk
Myh11
Contractility genes
B
Young
Aged
PDGFRα
p < 0.05
PDGFRα+ fibroblasts (μm² per field)
20000
15000
10000
5000
0
young
aged

Figure S6 (related to Figure 6). Fibroblast Markers and Immunostaining Validation of Altered Fibroblast Abundance with Age
(A) The fibroblasts captured by scRNA-seq express high level of extracellular matrix genes (top), but not contractility genes (bottom).
(B) Representative PDGFRα IHC staining of fibroblasts and quantification in young and aged mammary glands. Scale bar = 50 μm or 10 μm (inset). Analysis was performed on at least five regions per gland at 10X magnification, with n = 6 animals per age group, using Student's t test.

Figure S7

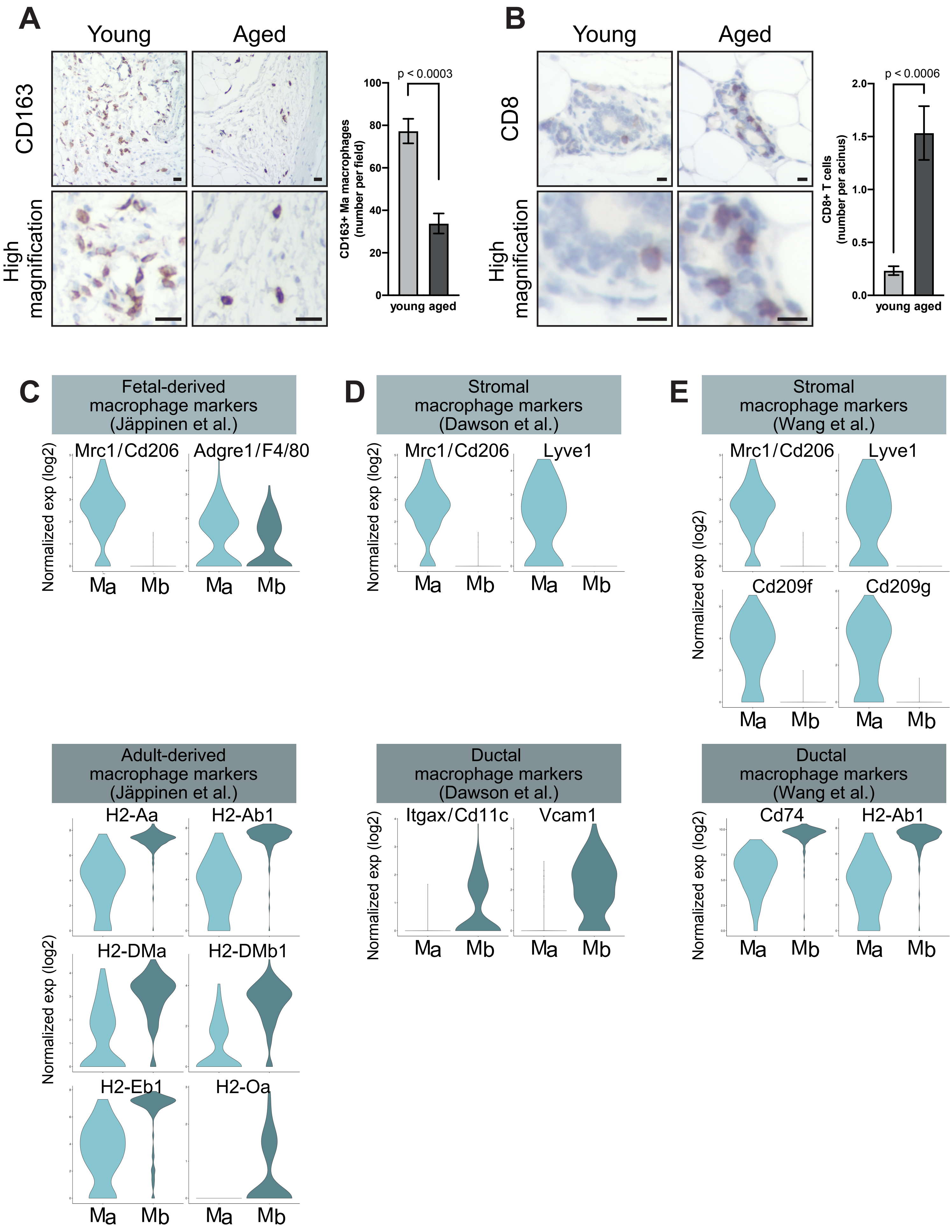
A
Young
Aged
CD163
High magnification
p < 0.0003
CD163+ Ma macrophages (number per field)
100
80
60
40
20
0
young aged
B
Young
Aged
CD8
High magnification
p < 0.0006
CD8+ T cells (number per acinus)
2.0
1.5
1.0
0.5
0.0
young aged
C
Fetal-derived macrophage markers (Jäppinen et al.)
Mrc1/Cd206
Adgre1/F4/80
Normalized exp (log2)
Ma
Mb
D
Stromal macrophage markers (Dawson et al.)
Mrc1/Cd206
Lyve1
E
Stromal macrophage markers (Wang et al.)
Mrc1/Cd206
Lyve1
Cd209f
Cd209g
Adult-derived macrophage markers (Jäppinen et al.)
H2-Aa
H2-Ab1
H2-DMa
H2-DMb1
H2-Eb1
H2-Oa
Ductal macrophage markers (Dawson et al.)
Itgax/Cd11c
Vcam1
Ductal macrophage markers (Wang et al.)
Cd74
H2-Ab1

Figure S7 (related to Figure 7). Immunostaining Validation of Altered Immune Cell Abundance and Comparison of Macrophage Markers

(A) Representative IHC staining of CD163+ M_a macrophages and quantification in young and aged mammary glands. Scale bar = 25 µm. Analysis was performed on 3-6 ECM-rich stromal regions per gland at 10X magnification, with n = 6 animals per age group, using Student's t test.

(B) Representative IHC staining of CD8+ T cells and quantification in young and aged mammary glands. Scale bar = 10 µm. Analysis was performed on at least 15 acini per gland at 20X magnification, with n = 6 animals per age group, using Student's t test.

(C) M_a macrophages express higher levels of markers for fetal-derived macrophages, whereas M_b macrophages express higher levels of markers for adult-derived macrophages. Markers were reported by Jäppinen et al., 2019.

(D-E) M_a macrophages are distinguished by stromal macrophage markers, and M_b macrophages by ductal macrophage markers. Markers were reported by Dawson et al., 2020 (D) and Wang et al, 2020 (E).

Table S1

Click here to access/download

Supplemental Videos and Spreadsheets

Table_S1_Cell type-specific gene signatures.xlsx

Table S2

Click here to access/download

Supplemental Videos and Spreadsheets

Table_S2_Cell Type proportions.xlsx

Table S3

Click here to access/download

Supplemental Videos and Spreadsheets

Table_S3_Young-vs-aged differentially expressed genes.xlsx

Table S4

Click here to access/download
Supplemental Videos and Spreadsheets
Table_S4_Pathway analysis.xlsx

Table S5

Click here to access/download
Supplemental Videos and Spreadsheets
Table_S5_Luminal HS-AV cell markers.xlsx

Table S6

Click here to access/download

Supplemental Videos and Spreadsheets

Table_S6_Macrophage subpopulation markers.xlsx

Click here to access/download
Supplemental File Sets
7.R scripts_Li et al.R
