# Supplementary material for: Aging-Associated Alterations in Mammary Epithelia and Stroma Revealed by Single-Cell RNA Sequencing
Source: Cell Rep. Author manuscript; Available in PMC 2021 Feb 22. (PMC7898263; doi:10.1016/j.celrep.2020.108566)
Supplement: 9 [file NIHMS1658643-supplement-9.pdf]

Cell Reports

Resource

Aging-Associated Alterations in Mammary Epithelia and Stroma Revealed by Single-Cell RNA Sequencing

Graphical Abstract

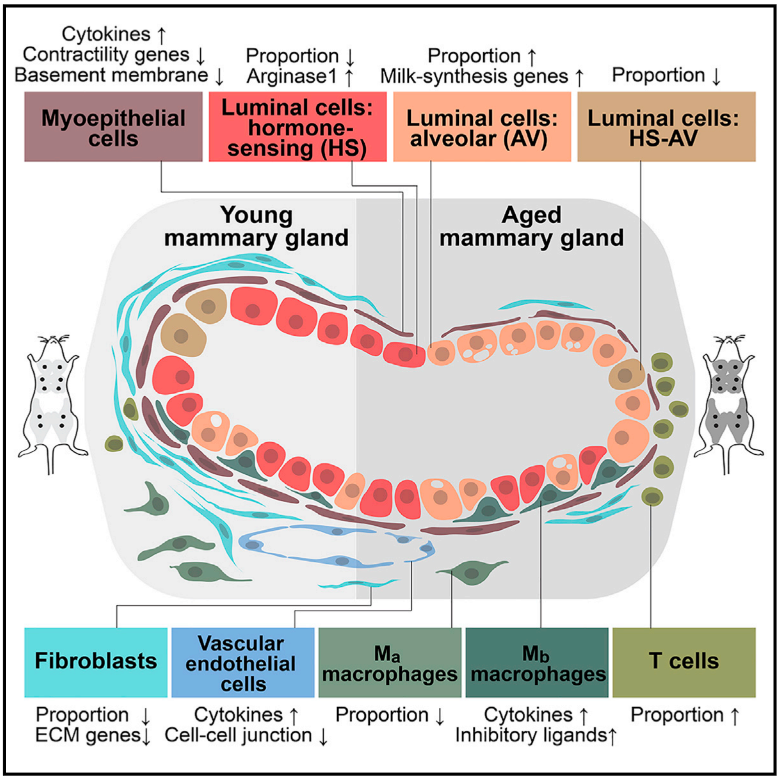

Authors

Carman Man-Chung Li, Hana Shapiro, Christina Tsiobikas, ..., Luca Pinello, Aviv Regev, Joan S. Brugge

Correspondence

joan_brugge@hms.harvard.edu

In Brief

Using single-cell RNA-sequencing, Li et al. compare mammary epithelia and stroma in young and aged mice. Age-dependent changes at cell and gene levels provide evidence suggesting alveolar maturation, functional deterioration, and potential pro-tumorigenic and inflammatory alterations. Additionally, identification of heterogeneous luminal and macrophage subpopulations underscores the complexity of mammary lineages.

Highlights

- scRNA-seq captures aging-associated alterations in mammary epithelia and stroma
- Identification of luminal and macrophage subpopulations reveals lineage complexity
- Aging correlates with alveolar maturation and potential cellular functional decline
- Aging is coupled to a potentially pro-tumorigenic and inflammatory microenvironment

Li et al., 2020, Cell Reports *33*, 108566
December 29, 2020 © 2020 The Author(s).
https://doi.org/10.1016/j.celrep.2020.108566

Cell Reports

CellPress
OPEN ACCESS

Resource

Aging-Associated Alterations in Mammary Epithelia and Stroma Revealed by Single-Cell RNA Sequencing

Carman Man-Chung Li,[1] Hana Shapiro,[1] Christina Tsiobikas,[1] Laura M. Selfors,[1] Huidong Chen,[2,3] Jennifer Rosenbluth,[1] Kaitlin Moore,[1] Kushali P. Gupta,[1] G. Kenneth Gray,[1] Yaara Oren,[1,2] Michael J. Steinbaugh,[4] Jennifer L. Guerriero,[5,6] Luca Pinello,[2,3] Aviv Regev,[2,7] and Joan S. Brugge[1,8,*]

[1]Department of Cell Biology, Harvard Medical School, Boston, MA 02115, USA
[2]Broad Institute of MIT and Harvard, Cambridge, MA 02142, USA
[3]Molecular Pathology Unit & Cancer Center, Massachusetts General Hospital Research Institute and Harvard Medical School, Charlestown, MA 02129, USA
[4]Department of Biostatistics, Harvard T.H. Chan School of Public Health, Boston, MA 02115, USA
[5]Breast Tumor Immunology Laboratory, Dana-Farber Cancer Institute, Boston, MA 02115, USA
[6]Division of Breast Surgery, Department of Surgery, Brigham and Women's Hospital, Boston, MA 02115, USA
[7]Present address: Genentech, 1 DNA Way, South San Francisco, CA 94080, USA
[8]Lead Contact
*Correspondence: joan_brugge@hms.harvard.edu
https://doi.org/10.1016/j.celrep.2020.108566

SUMMARY

Aging is closely associated with increased susceptibility to breast cancer, yet there have been limited systematic studies of aging-induced alterations in the mammary gland. Here, we leverage high-throughput single-cell RNA sequencing to generate a detailed transcriptomic atlas of young and aged murine mammary tissues. By analyzing epithelial, stromal, and immune cells, we identify age-dependent alterations in cell proportions and gene expression, providing evidence that suggests alveolar maturation and physiological decline. The analysis also uncovers potential pro-tumorigenic mechanisms coupled to the age-associated loss of tumor suppressor function and change in microenvironment. In addition, we identify a rare, age-dependent luminal population co-expressing hormone-sensing and secretory-alveolar lineage markers, as well as two macrophage populations expressing distinct gene signatures, underscoring the complex heterogeneity of the mammary epithelia and stroma. Collectively, this rich single-cell atlas reveals the effects of aging on mammary physiology and can serve as a useful resource for understanding aging-associated cancer risk.

INTRODUCTION

The mammary gland undergoes dynamic changes in cellular composition and gene expression over a lifetime (Inman et al., 2015). Although recent single-cell RNA sequencing (scRNA-seq) studies have characterized changes during embryonic development, puberty, and pregnancy (Bach et al., 2017; Giraddi et al., 2018; Pal et al., 2017; Wuidart et al., 2018), little is known about the alterations associated with aging, an important aspect of mammary development closely related to breast cancer (Jenkins et al., 2014). Although mammography, histology, and molecular analyses have revealed prominent changes with age, including decreased mammographic density, altered epithelial proportions, and reduced connective tissue (Azam et al., 2019; Dong et al., 2016; Garbe et al., 2012; Gertig et al., 1999; Hart et al., 1989; Hutson et al., 1985; McCormack et al., 2010; Pelissier Vatter et al., 2018; Pelissier et al., 2014), systematic single-cell transcriptome profiling can better capture age-associated effects at a higher resolution and on a larger scale. Moreover, given that increased age is strongly associated with breast cancer susceptibility in human and mouse models (Jenkins et al., 2014; LaBarge et al., 2016; Raafat et al., 2012), a single-cell atlas for aged mammary glands could help fill the gaps in our knowledge of aging and cancer risk. The use of human samples to study aging has been challenging because of confounding covariates, such as pregnancy history (parity) and accumulated environmental stress (Ginger and Rosen, 2003; Huh et al., 2015; Kamikawa et al., 2009; Russo et al., 2008; Dos Santos et al., 2015; Singletary and McNary, 1992). In contrast, mouse models can complement human studies by providing more-precise control of parity, hormone cycle, genetic background, and environmental factors, thus enabling a focus on age-specific effects.

Mammary gland function depends on both the epithelial and stromal compartments (Inman et al., 2015; Polyak and Kalluri, 2010). The mammary epithelial bilayer contains outer myoepithelial/basal cells and inner luminal cells, which, collectively, form branching ducts and terminal acinus. Myoepithelial cells are distinguished by their contractility and ability to synthesize basement membrane. Luminal cells, on the other hand, consist of two subtypes: hormone-sensing (HS) cells capable of

This is an open access article under the CC BY license (http://creativecommons.org/licenses/by/4.0/).

responding to endocrine signals, such as estrogen, progesterone, and prolactin; and secretory alveolar (AV) cells capable of producing milk. HS and AV cells have also been referred to as mature luminal (ML) and luminal progenitor (LP) cells, respectively, based on their *in vitro* colony-forming ability (Fu et al., 2020; Shackleton et al., 2006; Stingl et al., 2006); however, cumulative evidence suggests that these two lineages are independent in adulthood (Lilja et al., 2018; Van Keymeulen et al., 2017; Wang et al., 2017). Surrounding the epithelial cells are various stromal cells, including fibroblasts, vascular/lymphatic cells, immune cells, and adipocytes. Although initial scRNA-seq studies provided valuable knowledge of mammary epithelia in early and adult development (Bach et al., 2017; Giraddi et al., 2018; Nguyen et al., 2018; Pal et al., 2017; Wuidart et al., 2018), characterization of stromal cells has been limited (Han et al., 2018; Kanaya et al., 2019; Tabula Muris Consortium, 2018, 2020).

Here, we used scRNA-seq to analyze young and aged, murine, virgin mammary glands, identifying a significant diversity of epithelial and stromal cell. We captured a rare population of luminal epithelial cells co-expressing HS and AV lineage markers but distinguishable by a unique gene signature. We also detected heterogeneous stromal/immune populations, including distinct macrophage subtypes. In addition, comparison of young and aged cells identified aging-associated changes in cell-type compositions and transcriptomic profiles, providing evidence that suggests alveolar maturation and physiological decline. The analysis also identified potentially pro-tumorigenic alterations that may provide initial insight into mechanisms for age-associated vulnerability to breast cancer. Overall, these observations are highly consistent across multiple samples, suggesting that the intrinsic effects of aging on mammary tissues may follow a defined biological program and are not merely consequences of stochastic deterioration.

RESULTS

Diverse Epithelial and Stromal Cell Types Identified by scRNA-Seq in Mammary Glands

To study aging in mammary tissues, we performed scRNA-seq analysis using the 10× Chromium platform on young (3–4 months old, n = 3) and aged (13–14 months old, n = 4), virgin mice (Figure 1A), which correspond to human early adulthood (20–30 years old) and perimenopause (45–55 years old), respectively (Diaz Brinton, 2012; Finch et al., 1984; Flurkey et al., 2007). Mice were analyzed at diestrus, when progesterone levels and epithelial cell proliferation peak, to facilitate detection of progenitor cells (Brisken and Ataca, 2015; Fata et al., 2001; Joshi et al., 2010). With stringent filtering at cell and gene levels to eliminate potential doublets, we captured a total of 13,684 cells and 27,998 genes. Single cells were clustered based on gene expression profiles using Seurat and visualized using t-distributed stochastic neighbor embedding (t-SNE) (Butler et al., 2018; Stuart et al., 2019) (Figures 1B and S1A). Doublet estimation using Scrublet (Wolock et al., 2019) and DoubletFinder (McGinnis et al., 2019) demonstrated that doublets likely accounted for a negligible fraction of our data and did not drive clustering (Figure S1B).

Epithelial and stromal cell types were identified using canonical markers (Figures 1B–1F). As expected, we detected epithelial cells (n = 8,901; expressing *Epcam*) of myoepithelial lineage (n = 1,670; expressing *Krt17*, *Krt14*, *Krt5*, *Acta2*, *Myl9*, *Mylk*, and *Myh11*) or luminal lineage (n = 7,231; expressing *Krt19*, *Krt18*, and *Krt8*) (Figure 1E). Luminal cells consisted of both HS cells (n = 2,138; expressing *Prlr*, *Pgr*, *Esr1*, *Cited1*, and *Prom1*) and AV cells (n = 4,820; expressing *Mfge8*, *Trf*, *Csn3*, *Wfdc18*, *Ltf*, and *Elf5*). AV cells expressed luminal progenitor markers (*Kit*, *Aldh1a3*, and *Cd14*) and milk biosynthesis-related genes (*Mfge8*, *Trf*, *Csn3*, *Wfdc18*, and *Ltf*), but not mature alveolar markers associated with pregnancy and lactation (*Wap*, *Glycam1*, and *Olah*) (Figure 1E), consistent with their alveolar progenitor differentiation state in virgin glands (Booth et al., 2007). In addition, we identified a rare luminal population co-expressing hormone-sensing markers and alveolar progenitor markers (n = 273; Figures 1B, 1C, and 1E). We termed these HS-AV cells. This population is unlikely to be doublets based on the doublet analyses above (Figure S1B).

Among stromal cells (n = 4,783), we detected fibroblasts (n = 1,993; expressing *Col1a1*, *Col1a2*, *Col3a1*, and *Fn1*), vascular/lymphatic cells (n = 2,196; expressing *Pecam1*, *Cdh5*, and *Eng*), and immune cells (n = 594; expressing *Ptprc*, which encodes CD45) (Figure 1F). The vascular/lymphatic population consisted of vascular endothelial cells (n = 1,819; expressing *Sox17* and *Sele*), pericytes (n = 349; expressing *Rgs5*, *Des*, and *Notch3*), and lymphatic endothelial cells (n = 28; expressing *Mmrn1*, *Prox1*, *Flt4*, and *Ccl21a*). The immune population contained both myeloid cells (marked by Cd74 and Lyz2) and lymphocytes (marked by *Cd3d*, *Cd3e*, and *Cd3g*). Within myeloid cells, we detected dendritic cells (n = 226; expressing *Napsa*, *Traf1*, *Cd209a*, and *Flt3*) and two populations of macrophages (n = 268; both expressing *Csf1r*, *Fcgr3*, *Adgre1*, and *Ms4a7*, but distinguishable by *Mrc1*, *Cd209f*, and *Cd163* [designated as M_a hereafter] or by *Mmp12, Mmp13*, and *Spic* [designated as M_b]). Within lymphocytes, we detected natural killer (NK) cells (n = 40; expressing *Gzma*, *Ncr1*, and *Itgae*) as well as T and B cells (n = 60), with most being CD8 T cells (marked by *Cd8a* and *Cd8b1*) and a very small fraction being CD4 T cells (marked by *Cd4*) and B cells (marked by *Cd79a* and *Cd79b*). These immune cells represented mammary-tissue-specific cells because we discarded the lymph nodes in sample preparation. The only major stromal population not captured in our analysis was adipocytes, which were removed with the supernatant during tissue dissociation.

We generated gene signatures characterizing each cell type by multiple pairwise differential gene expression analyses (see Method Details). The resulting cell-type-specific signatures (Figure 1G; Table S1) can serve as a useful resource for identifying or isolating cell types within heterogeneous mammary populations. Importantly, the ability to capture relatively rare populations, such as lymphatic endothelial cells and various immune cells, as well as the ability to detect lesser-known populations, such as HS-AV cells and distinctive macrophages, highlights the power of scRNA-seq to identify diverse cell types in the mammary gland without *a priori* knowledge required to pre-isolate these populations.

CellPress
OPEN ACCESS

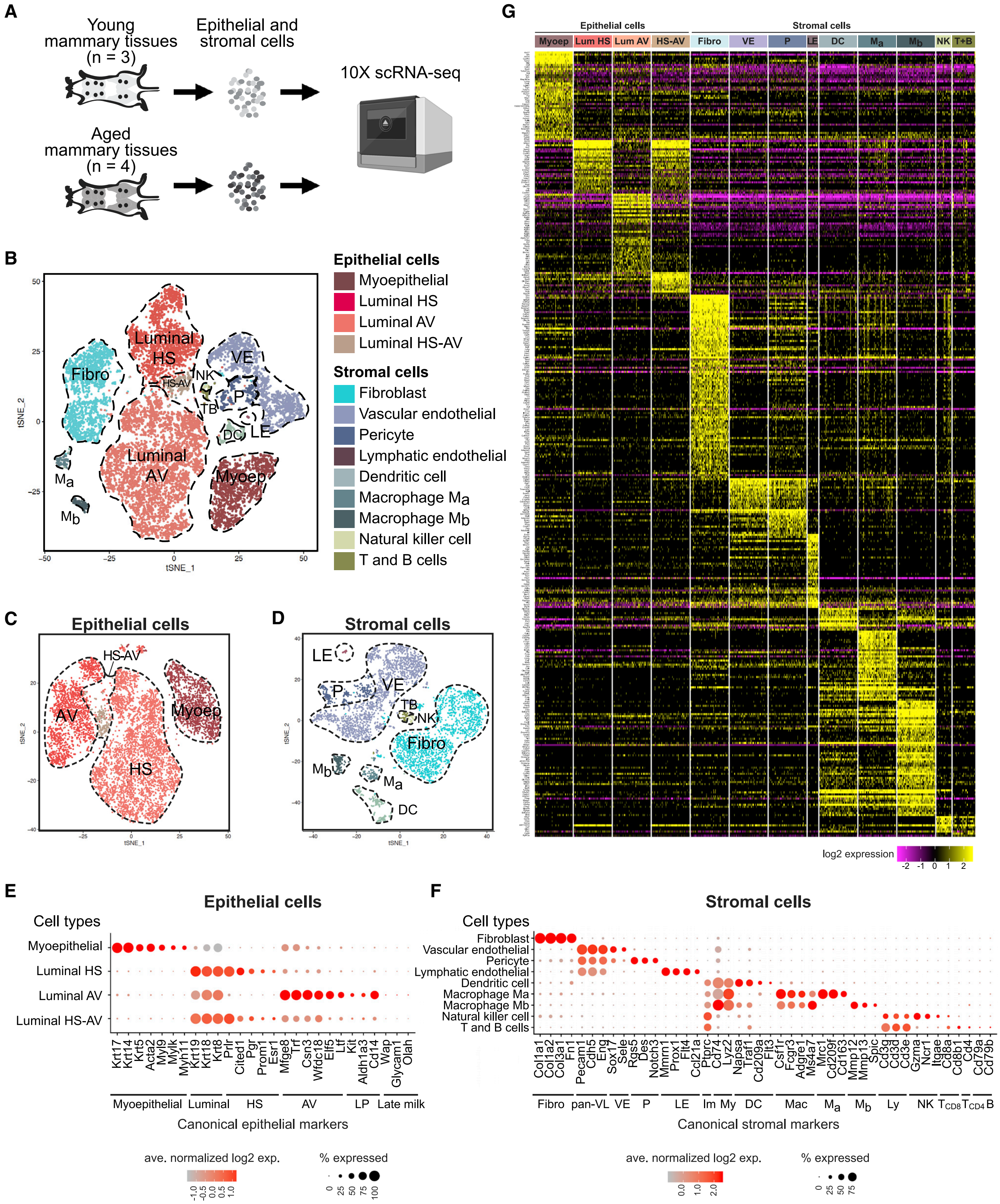

Figure 1. Diverse Epithelial and Stromal Cell Types Identified by scRNA-Seq in Mammary Glands

(A) scRNA-seq experimental setup. Freshly dissociated epithelial and stromal cells were analyzed without FACS enrichment.

(B) t-SNE plot showing epithelial and stromal clusters identified based on characteristic markers.

(C and D) Epithelial (C) and stromal (D) cells subsetted and reclustered.

(legend continued on next page)

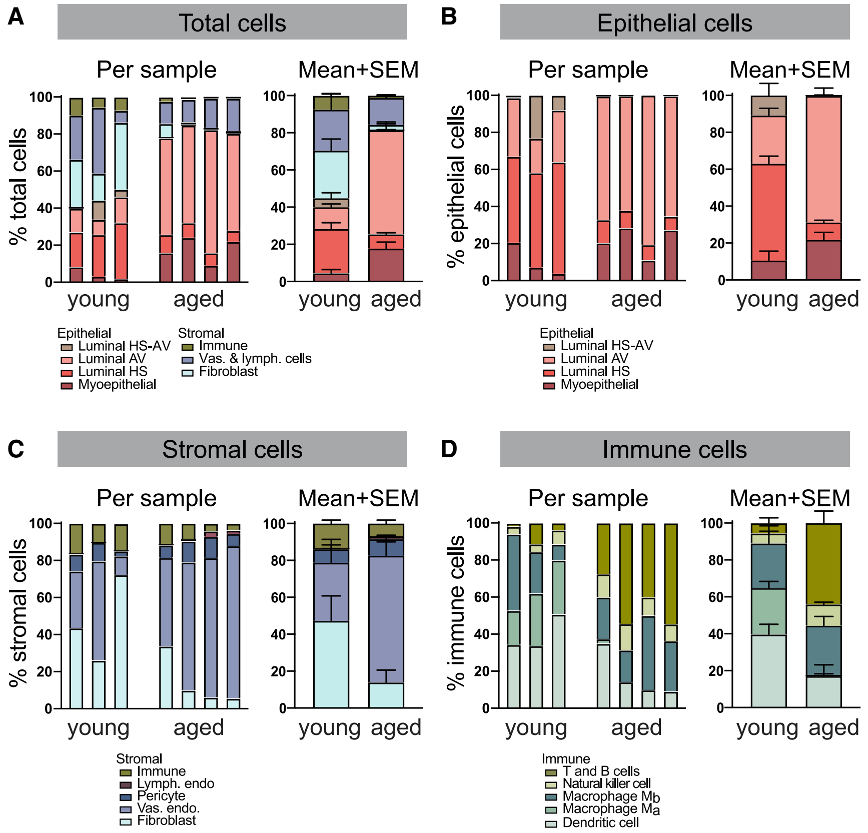

Figure 2. Prevalent Alterations in Cell Proportions in Aged Mammary Glands

Relative proportions of specified cell types among total (A), epithelial (B), stromal (C), and immune (D) cells.

(E and F) Expression of canonical markers of epithelial (E) and stromal (F) cell types.

(G) Heatmap of cell type-specific gene signatures. See also Table S1.

Abbreviations: Myoep, myoepithelial; HS, hormone-sensing; AV, alveolar; LP, luminal progenitors; Fibro, fibroblasts; pan-VL, pan-vascular/lymphatic cells; VE, vascular endothelial cells; P, pericytes; LE, lymphatic endothelial cells; Im, immune cells; My, myeloid cells; DC, dendritic cells; Mac, macrophages; M_a, M_a macrophages, M_b, M_b macrophages; Ly, lymphocytes; NK, natural killer cells; T, T cells; B, B cells.

Prevalent Alterations in Cell Proportions and Gene Expression in Aged Mammary Glands

ScRNA-seq analysis revealed differences between young and aged mammary glands in both cell type composition and gene expression. Cell type composition inferred from the transcriptomic data is consistent among biological replicates despite sample-to-sample variation and differs significantly across age groups (Figures 2A–2D, S1A, and S2A; Table S2). The relative proportion of epithelial cells to stromal cells increased considerably with age: epithelial cells increased from 45% of total cells to 82%, whereas stromal cells decreased commensurately from 55% to 18% (Figure 2A; $p < 0.0001$, Fisher's exact test). Within the epithelial and stromal compartments, cell type composition also changed with age. Among epithelial cells (Figure 2B), AV luminal cells expanded 3-fold ($p < 0.0001$, Fisher's exact test), whereas HS luminal cells diminished 6-fold ($p < 0.0001$, Fisher's exact test). Among stromal cells (Figure 2C), the relative abundance of fibroblasts fell 3-fold with aged cells ($p < 0.0001$, Fisher's exact test), whereas vascular endothelial cells rose 2-fold ($p < 0.0001$, Fisher's exact test). Furthermore, although the proportion of total immune cells within the stromal compartment remained relatively unaltered, the composition of immune cell types changed with age (Figure 2D). The proportion of dendritic cells decreased 2-fold ($p < 0.0004$, Fisher's exact test). M_a macrophages decreased 40-fold ($p < 0.0001$, Fisher's exact test), whereas M_b macrophages remained relatively constant, thus rendering M_b the dominant macrophage population in aged tissues. In contrast to the reduction in myeloid cells, the proportion of lymphocytes increased with age. Specifically, NK cell percentage increased 2-fold ($p < 0.03$, Fisher's exact test), whereas CD8 T cells increased 8-fold ($p < 0.0001$, Fisher's exact test). Gene signature-based cell-cycle inference analysis of epithelial populations revealed varying degrees of proliferation, which is consistent with the high proliferation rate expected at diestrus from progesterone stimulation (Figure S2B) (Fata et al., 2001; Joshi et al., 2010). The proportion of proliferating epithelial cells decreased with age, in accordance with previous observations (Raafat et al., 2012); the decrease was only moderate, again, likely due to the effect of diestrus. Notably, the higher proliferation of certain cell types (myoepithelial and AV cells) compared with others (HS and HS-AV) is concordant with previous studies (Clarke et al., 1997; Giraddi et al., 2015) and may partially explain the accumulation of myoepithelial and AV cells relative to HS and HS-AV cells at older age (Figure 2B). Besides proliferation, other factors, such as cell death, migration, and differentiation, may also account for the age-dependent changes in cell type proportions.

In addition to composition, cell type-specific gene expression differences were also detected in young and aged mammary glands (Figures S3A–S3L). Within the epithelial compartment, myoepithelial cells exhibited the largest number of differentially expressed genes (Figure S3A–S3D), whereas within the stromal compartment, vascular endothelial cells showed the most striking pattern (Figure S3E–S3H). Moreover, M_b macrophage gene

expression was affected to a greater extent compared with other immune cells (Figure S3I–S3L). These age-dependent changes are examined in greater detail below. Analysis of the number of genes detected per cell indicated that these age-associated gene expression patterns were not simply driven by sample variation in total genes captured by scRNA-seq (Figure S3M). Overall, our results demonstrate that both cellular proportion and gene expression are profoundly affected by aging.

Aged Myoepithelial Cells Show Altered Gene Expression

Myoepithelial cells, distinguished by basal keratins *Krt17*, *Krt14*, and *Krt5* (Figure 3A), only increased slightly in abundance with age (Figure 3B); however, they exhibited a striking degree of gene expression changes (Figure 3C; Table S3). Differential expression analysis detected 111 genes upregulated and 106 genes downregulated in aged myoepithelial cells. Pathway analysis indicated that those genes primarily fell into four functional categories—cytokines/growth factors, oxidative phosphorylation, extracellular matrix (ECM), and cytoskeleton/contractility genes (Figures 3D–3H; Table S4). More specifically, aged myoepithelial cells increased expression of multiple cytokines that are known to regulate the immune system (including *Cxcl1*, *Cxcl2*, *Cxcl16*, *Csf1*, and *Csf3*), as well as several growth factors (*Tgfb1*, *Jag1*, and *Vegfa*) (Figure 3E; Table S3). Aged myoepithelial cells also showed lower expression of oxidative phosphorylation genes (*Ndufa3*, *a5*, *a7*, *a8*, *a13*, *b3*, *b9*, *b10, c1*, *v3*, *Atp5j*, *Etfb*, *Uqcr10*, and *Uqcr11*) (Figure 3F; Table S3), which may reflect reduced metabolic activity. Expression of several ECM-related genes decreased with age (*Dcn*, *Col4a1*, *Col4a2*, *Serpinh1*, *Sparc*, *Emid1*, *Dag*, and *Spon2*) (Figure 3G; Table S3), indicating a potential impairment in the function to synthesize basement membrane. Expression is also reduced in multiple actomyosin-related genes (*Acta2*, *Actg2*, *Mylk*, *Myl6*, *Myl9*, and *Myh11*) as well as the cytokeratin intermediate filament *Krt15* (Figure 3H; Table S3), indicating a potential decline in the ability to maintain contractility.

Of note, because *Krt15* and *Acta2* (also known as smooth muscle actin [SMA]) have been reported as myoepithelial markers in murine mammary glands (Bach et al., 2017; Pal et al., 2017), their reduced expression with age would suggest that they may not be reliable markers for older myoepithelial cells. We, therefore, performed immunofluorescence staining to confirm their protein expression pattern in young and aged mammary glands, using KRT14 as a reference myoepithelial marker because its expression remained stable as detected by scRNA-seq. Consistent with scRNA-seq data, immunostaining demonstrated that KRT15 and ACTA2/SMA were expressed in young myoepithelial cells, but their level significantly decreased in aged glands (Figures 3I–3L). Therefore, our findings suggest that *Krt15* and *Acta2*, as well as other cytoskeletal or contractility genes (e.g., *Mylk*, *Myl6*, *Myl9*, and *Myh11*), should be used as myoepithelial markers with caution depending on age.

Alterations in Luminal HS and AV Cells with Age

We next examined age-related changes in luminal HS cells and AV cells. AV cells were identified by their characteristic milk-related genes *Mfge8*, *Csn3*, *Trf*, *Wfdc18*, and *Ltf* (Figure 4A), whereas HS cells were identified by hormone-sensing markers *Prlr*, *Pgr*, and *Esr1* and canonical HS lineage markers *Cited1* and *Prom1* (Figure 4B). The proportion of AV cells expanded with age from 26% to 69% within the epithelial compartment, and HS cells diminished from 53% to 9% (Figures 4C and 4D). Fluorescence-activated cell sorting (FACS) analysis of an independent cohort of mice confirmed the age-dependent increase of AV cells (Lin^{-}, Epcam^{+}, CD133^{-}, CD14$^{+/-}$; from 62% to 77% of the luminal compartment) and decrease of HS cells (Lin^{-}, Epcam^{+}, CD133^{+}, CD14^{-}; from 37% to 22% of the luminal compartment) (Figure 4E). The difference in the exact percentages detected by scRNA-seq and FACS is likely due to technical differences as well as post-transcriptional and/or translational regulations.

Differential expression analysis of young versus aged AV cells revealed 24 upregulated genes and 24 downregulated genes (Figure 4F; Table S3), including increased expression of several milk-biosynthesis regulators that are exclusively detected in AV cells but not HS cells. Those genes include pyruvate dehydrogenase kinase *Pdk4* (Kankel and Reinauer, 1976), milk lipid droplet-coating protein *Plin2* (Russell et al., 2011), and milk bioactive protein *Cd59a* (Bjørge et al., 1996) (Figure 4G). This expression pattern led us to examine other milk-related genes, many of which were also upregulated with age in AV cells in at least three of four replicates, including lactose synthase subunit *Lalba*; caseins *Csn1s1*, *Csn1s2a*, and *Csn2*; and milk osteopontin *Spp1* (Figure S4A). The increased expression of milk biosynthesis genes may potentially reflect a shift toward a more mature alveolar state. This is consistent with previous reports (Raafat et al., 2012) and our observation that aged mammary glands showed signs of activated alveolar cells independent of pregnancy, including increased secretory material in acini and dilated ducts (Figure S4B).

On the other hand, aged HS cells showed upregulation of 18 genes and downregulation of 12 genes (Figure 4H; Table S3). Many of those were regulated by hormone receptors, consistent with their expression in HS cells and potentially reflecting hormonal changes associated with aging (Ace and Okulicz, 1998; Gannon et al., 2010; Gundlah et al., 2005; Hickey et al., 2013; Ono et al., 2014; Sampath et al., 2002; Tsai et al., 2002; Tynan et al., 2005). Two upregulated genes, *Tph1* and *Arg1*, were particularly noteworthy because of their exclusive expression in HS cells and no other cell types (Figure 4I). Expression of *Tph1* (tryptophan hydroxylase 1) in the mammary gland is specifically activated during lactation for serotonin biosynthesis (Laporta et al., 2014; Matsuda et al., 2004). Its increased level in aged HS cells is consistent with the lactation-primed state of aged mammary glands discussed above. *Arg1* (arginase 1) inhibits proliferation and function of T cells and NK cells by depleting L-arginine in the microenvironment (Bronte and Zanovello, 2005; Gantt et al., 2014). *Arg1* is commonly expressed by immune inhibitory cells, such as tumor-associated macrophages and myeloid derived suppressor cells, as well as by a diversity of tumor cells, including breast cancer. We confirmed ARG1 protein expression in HS cells as well as its increase with age by performing tissue staining in an independent cohort of mice (Figure S4C). Increased *Arg1* expression in aged HS cells is consistent with an immunosuppressive microenvironment, as discussed later.

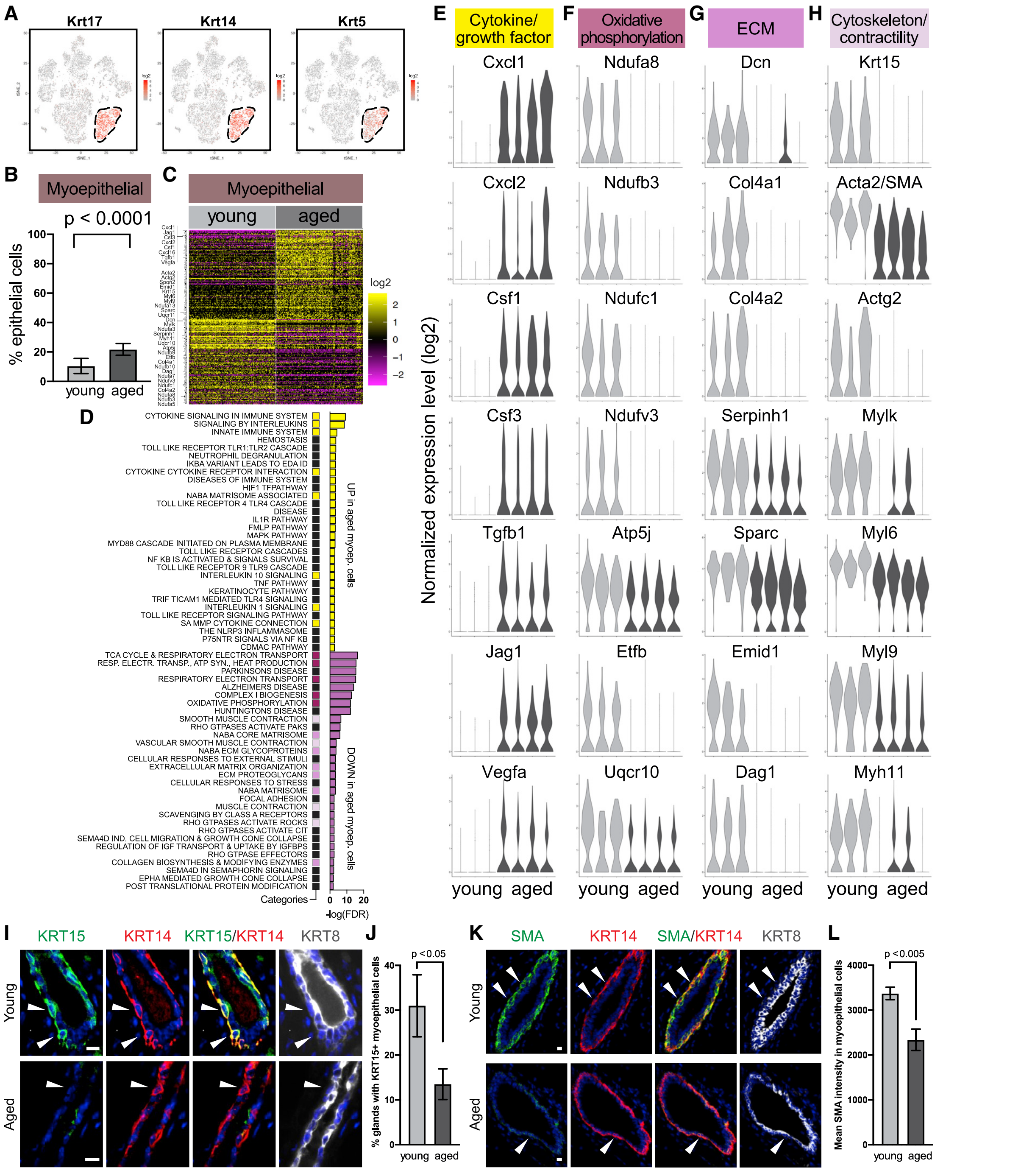

Figure 3. Aged Myoepithelial Cells Show Altered Gene Expression

(A) Myoepithelial cells are identified by basal keratins in scRNA-seq data.

(B) Relative proportion of myoepithelial cells in young (n = 3) and aged (n = 4) mice by scRNA-seq (Fisher's exact test).

(C) Differentially expressed genes in young versus aged myoepithelial cells. See also Table S3.

(D) Top gene sets identified by pathway analysis of differentially expressed genes in (C), with recurring gene sets highlighted. See also Table S4.

(E–H) Violin plots showing expression of select genes from (D).

(I–L) Representative immunofluorescence staining of KRT15 (I) and SMA (K) in myoepithelial cells (stained as $KRT14^{+}$ and $KRT8^{-}$, indicated by arrows). Scale bar, 10 μm. Quantifications are shown in (J) and (L), respectively (n = 6 mice per age group, Student's t test).

CellPress
OPEN ACCESS

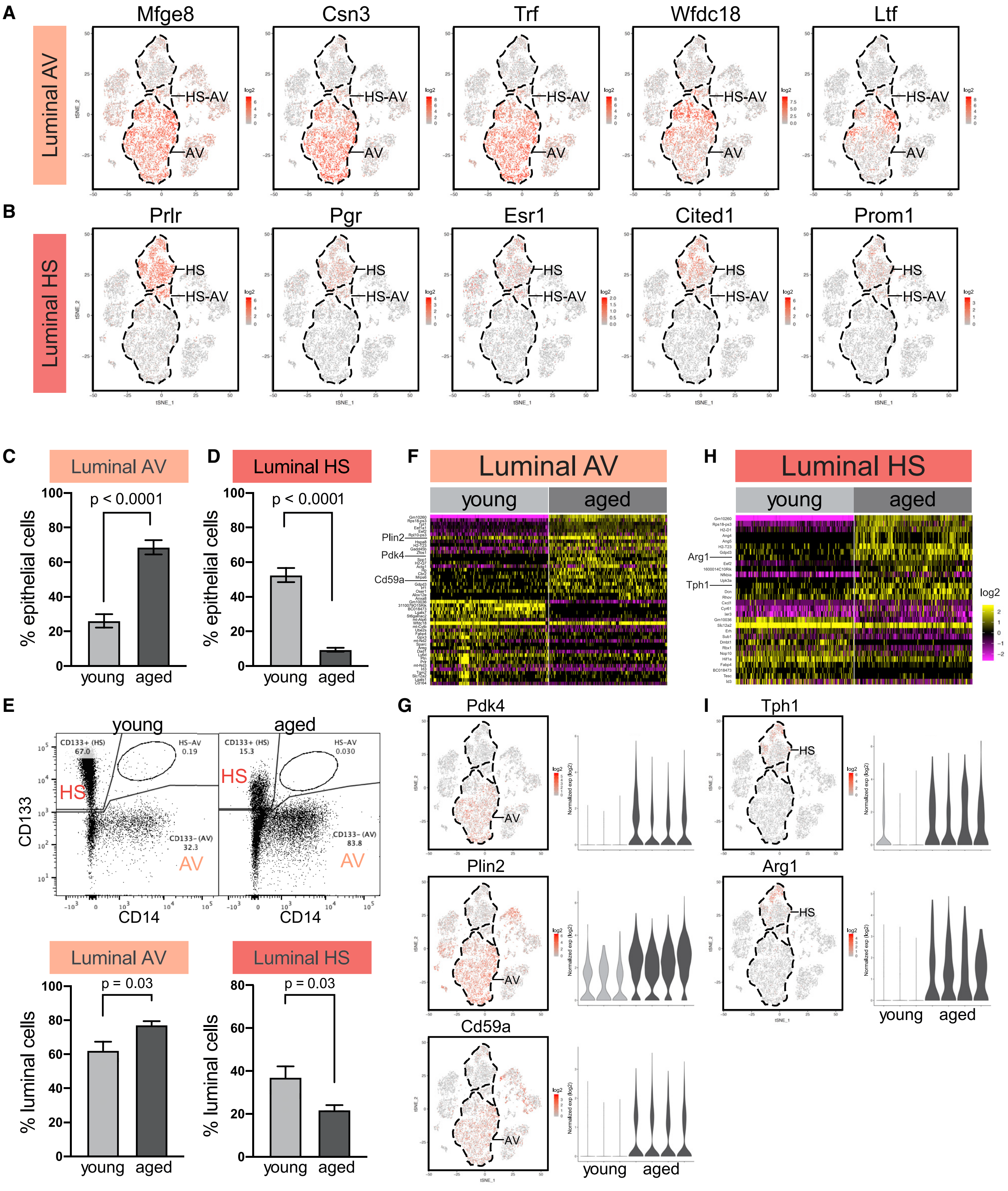

Figure 4. Alterations in Luminal Hormone-Sensing (HS) Cells and Alveolar (AV) Cells with Age

(A) AV cells are distinguished by milk-related genes in scRNA-seq data.

(B) HS cells are distinguished by hormone receptors and canonical HS lineage markers.

(C and D) Relative proportion of AV (C) and HS (D) cells in young (n = 3) and aged (n = 4) mice by scRNA-seq (Fisher's exact test).

(legend continued on next page)

Luminal HS-AV Cells Showed Age-Dependent Abundance

Our scRNA-seq analysis also revealed a rare population of HS-AV cells that co-expressed HS and AV markers (Figures 1E, 4A, and 4B), accounting for an average of 11% of epithelial cells in young mammary glands (Figure 5A). Similar cells expressing HS and AV gene signatures have also been identified in young mice by Pal et al. (2017) and Bach et al. (2017) at comparable proportions and in ovariectomized mice by Kanaya et al. (2019) at higher abundance (Figures S5A and S5B), but they have not been further characterized. We performed differential gene expression analysis to identify markers distinguishing HS-AV cells from HS cells and AV cells, limiting the analysis to young tissues to focus on cell-type-specific difference independent of aging. The results revealed that HS-AV cells are distinguished by higher expression of 72 genes and lower expression of nine genes (Figures 5B and 5C; Table S5). Many of the upregulated markers fell into four functional categories, highlighting the potential implications for the biological roles of HS-AV cells (Figure S5C): (1) regulators of mammary development, lobulo-alveologenesis, and lactation, such as *Spry2*, *B4galt1*, *Slc39a1*, *Skil*, *Agpat1*, and *Neat1*, suggesting that HS-AV cells might play a role in mammary gland morphogenesis and milk production (Agarwal et al., 2017; Crisà et al., 2016; Jahchan et al., 2012; 2010; Littlejohn et al., 2010; Lv et al., 2015; McCormick et al., 2014; Sigurdsson et al., 2013; Standaert et al., 2014; Zhang et al., 2014, 2018b; Zhu and Nelson, 2013); (2) chromatin-modifying enzymes, including *Kmt2d/Mll2*, *Whsc1l1/Nsd3*, *Gatad1/Odag*, *Kdm6b/Jmjd3*, *Cbx3/Hp1γ*, and *Supt16/Fact140*, which have been implicated in promoting stem cell phenotypes and regulating differentiation (Burchfield et al., 2015; Denissov et al., 2014; Froimchuk et al., 2017; Lomniczi et al., 2015; Mas et al., 2018); (3) transcription factors, including *Nfia*, *Tmf1*, *Bhlhe41*, *Arglu1*, *Btg1*, and, in particular, *Sox9*, which is known to regulate luminal progenitor cell fate (Malhotra et al., 2014; Guo et al., 2012; Wang et al., 2017); and (4) mRNA processing and modification enzymes, namely *Srrm2*, *Wtap*, *Alkbh5*, *Rbm47*, *Hnrnpul1*, and *Pan3*, which regulate mRNA splicing, m^6A methylation, mRNA processing, and degradation (Blencowe et al., 1998; Kim et al., 2019; Kzhyshkowska et al., 2003; Wahle and Winkler, 2013; Yue et al., 2018; Zheng et al., 2013). Overall, these gene signatures suggest a potential role for HS-AV cells in differentiation plasticity.

Next, we examined the lineage relationship of HS-AV cells to HS cells and to AV cells by performing STREAM (single-cell trajectories reconstruction, exploration, and mapping) lineage trajectory analysis (Chen et al., 2019) on scRNA-seq data of young mice. HS-AV cells primarily localized to the bifurcation junction between HS cells and AV cells (Figures 5D and S5D). This pattern was highly reproducible when the analysis was repeated within each of the three young samples (Figure S5D). The predicted lineage trajectory is consistent with the gene expression pattern of HS-AV cells being a hybrid state between the HS and AV lineages and suggests the possibility that HS-AV cells might have the potential to differentiate into HS cells and into AV cells.

Because HS-AV cells have not been reported beyond scRNA-seq studies, we further verified their existence using two orthogonal approaches. First, we performed *in situ* immunofluorescence staining in a panel of young mammary glands, using established hormone-sensing markers—progesterone receptor (PR) and estrogen receptor (ER)—and alveolar markers—lactotransferrin (LTF) and milk fat globule-EGF factor 8 (MFGE8). This analysis detected the presence of HS-AV cells co-expressing PR/ER and LTF/MFGE8, scattered within both ducts and acini (Figures 5E and 5F). Quantification showed that PR^+/$MFGE8^+$ cells and ER^+/LTF^+ cells represented 0.72% and 0.16% of luminal cells, respectively (Figures 5G–5J). The lower abundance of ER^+/LTF^+ cells compared with PR^+/$MFGE8^+$ cells by immunostaining is consistent with the lower abundance of LTF^+ cells compared with $MFGE8^+$ cells within the HS-AV population by scRNA-seq (Figure 4A). As further validation, we analyzed an independent set of young mice by FACS, using HS marker CD133 and AV marker CD14. Again, HS-AV cells can be detected as $CD133^+$/$CD14^+$ cells within the luminal population (Lin−, $Epcam^{hi}$) in all young tissues examined, with an average abundance of 0.25% (Figure 5K). Similar $CD133^+$/$CD14^+$ HS-AV cells were also detected in murine mammary organoid cultures (Figure S5E). Importantly, we confirmed that these cells were not doublet artifacts formed by $CD133^+$ cells and $CD14^+$ cells because microscopy examination of FACS-isolated $CD133^+$/$CD14^+$ cells demonstrated that they were indeed singlets (Figure S5F). Of note, although HS-AV cells can be detected using multiple approaches, they were in lower abundance when analyzed at the protein level by immunofluorescence and FACS (Figures 5H, 5J, and 5K) than at the RNA level by scRNA-seq (Figure 5A). The limited number of protein markers used in immunofluorescence and FACS analyses likely only captured a subset of the HS-AV population identified transcriptomically. In addition, the difference may reflect gene expression regulation at the post-transcriptional and/or translational levels.

Interestingly, the abundance of HS-AV cells diminished dramatically with age. ScRNA-seq showed that HS-AV cells accounted for an average of 11% of epithelial cells in young mice but only 0.3% in aged mice (Figure 5A). We confirmed this age-dependent decrease of HS-AV cells by immunofluorescence staining of PR^+/$MFGE8^+$ cells and ER^+/LTF^+ cells in tissues (Figures 5G–5J). In addition, FACS analysis of an independent cohort also demonstrated that aged mice consistently harbored a lower proportion of HS-AV cells than young mice had (Figure 5K). Collectively, these results established an age-dependent existence of the HS-AV population.

Age-Dependent Alterations in Stromal Fibroblasts and Vascular/Lymphatic Cells

In addition to mammary epithelial cells, we analyzed age-associated changes in stromal cells (Figures 6A–6D). The most

(E) FACS analysis of relative abundance of AV cells and HS cells in young (n = 8) and aged (n = 6) mice. In the representative FACS plots, 10,000 cells are displayed. Statistical significance is determined by Student's t test.

(F and G) Differentially expressed genes in young versus aged AV cells presented as heatmap (F) and specific examples (G). See also Table S3.

(H and I) Differentially expressed genes in young versus aged HS cells presented as heatmap (H) and specific examples (I). See also Table S3.

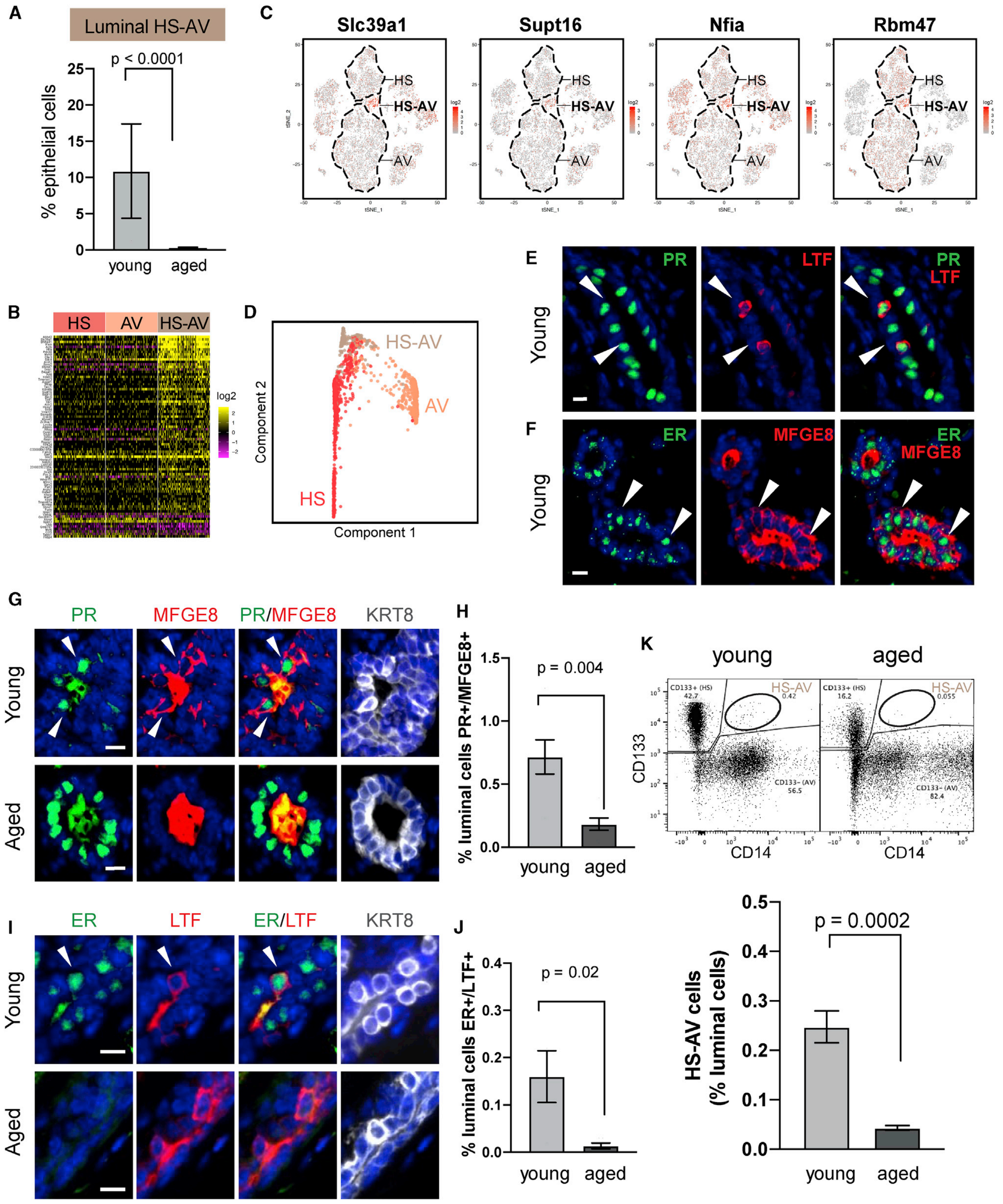

Figure 5. Luminal HS-AV Cells Show Age-Dependent Abundance

(A) Relative proportion of HS-AV cells in young (n = 3) and aged (n = 4) mice by scRNA-seq (Fisher's exact test).

(B) Markers distinguishing HS-AV cells from both HS cells and AV cells in young mice. See also Table S5.

(C) Expression pattern of select HS-AV markers in (B).

(legend continued on next page)

abundant stromal cell type in young tissues was fibroblasts (Figures 6E–6H). These cells homogeneously expressed fibroblast markers *Pdgfra*, *Pdgfrb*, and *Fap* (Figure 6A). They also expressed high level of ECM genes (e.g., *Fn1*, *Col1a1*, *Col1a2*, and *Col3a1*) but not contractility genes (e.g., *Acta2*, *Myl9*, *Mylk*, and *Myh11*) (Figure S6A), indicating that they were ECM-producing fibroblasts instead of contractile myofibroblasts. The relative proportion of fibroblasts decreased with age (Figure 6E), a pattern corroborated by immunohistochemistry (IHC) staining using the fibroblast marker PDGFRα (Figure S6B). In addition, scRNA-seq differential expression analysis revealed 11 genes upregulated and 37 genes downregulated with age (Figure 6I; Table S3), including increased expression of stress-related genes (*Hspa1a*, *Sqstm1*, *Ubc*, *Cebpb*, and *Gadd45b*) and decreased expression of ECM-related genes (*Col5a3*, *Col6a3*, *Fn1*, and *Mmp23*) (Figures 6J and 6K; Table S4). Notably, fibronectin, encoded by *Fn1*, is a major ECM component and a master organizer of the matrix assembly (Halper and Kjaer, 2014; Pankov and Yamada, 2002; Theocharis et al., 2016). The decrease in both fibroblast proportion and ECM gene expression is consistent with previous reports in human mammary glands that aging is associated with a reduction of connective tissues (Gertig et al., 1999; Hutson et al., 1985).

Other stromal populations captured by scRNA-seq included vascular and lymphatic cells, namely vascular endothelial cells (expressing *Pecam1*, *Chd5*, and *Sox17*), pericytes (expressing *Des*, *Rgs5*, and *Notch3*), and lymphatic endothelial cells (expressing *Mmrn1*, *Prox1*, and *Flt4*) (Figures 6B–6D). Among those populations, vascular endothelial cells exhibited the most dramatic changes with age, increasing in relative abundance (Figure 6F) and expressing 159 upregulated and 169 downregulated genes (Figure 6L; Table S3). Pathway analysis revealed that many upregulated genes were associated with cytokines that are known to affect the immune microenvironment (*Csf3, Cxcl1*, *Cxcl16*, and *Il6*), whereas many downregulated genes were associated with cell-cell junctions (including adherens junction genes *Ctnnb1*, *Jup*, *Pvrl2*, *Cdh5*, and *Mllt4* and tight junction genes *Cldn5* and *F11r*) (Figures 6M and 6N; Table S4). These changes may reflect an altered endothelial-immune interaction and vascular permeability. In comparison, pericytes and lymphatic endothelial cells remained relatively constant with age in terms of proportion (Figures 6G and 6H; Table S2) and gene expression (Figures S3G and S3H).

Age-Dependent Alterations in Myeloid and Lymphoid Immune Cells

ScRNA-seq also detected remarkable changes in the composition of immune cell types in aged mammary glands. As mentioned above, the proportion of myeloid cells diminished, whereas that of lymphoid cells expanded with age. Among myeloid cells, dendritic cells (expressing *Napsa*, *Cd209a*, and *Flt3*) and M_a macrophages (expressing *Cd163, Mrc1*, and *Cd209f*) decreased in relative abundance, leaving M_b macrophages (marked by *Mmp12, Mmp13*, and *Spic*) the major myeloid population in older mammary glands (Figures 7A–7C). Among lymphoid cells, the proportion of both NK cells (expressing *Gzma*, *Ncr1*, and *Itgae*) and CD8 T cells (expressing *Cd8a* and *Cd8b1*) increased with age (Figures 7D and 7E). Because M_a macrophages and CD8 T cells represented the most striking proportional changes, we also analyzed their relative abundance by IHC staining. CD163$^+$ M_a macrophages, which are localized preferentially in stroma, decreased in abundance with age (Figure S7A). In contrast, CD8$^+$ T cells, which were closely associated with the mammary epithelium, increased in abundance with age (Figure S7B). Compared with scRNA-seq, IHC is considerably more sensitive in detecting these rare cell types.

Macrophages have critical roles in mammary gland development and breast cancer (Brady et al., 2016; Gouon-Evans et al., 2002; Guerriero, 2018); however, the heterogeneity of resident macrophages within healthy mammary glands remains incompletely understood. To further characterize the two macrophage populations captured by scRNA-seq, we performed a differential gene expression analysis and obtained 241 markers that distinguished M_a and M_b macrophages (Figure 7F; Table S6). The results suggest that M_a and M_b macrophages do not show a clear association with classical M1/M2 polarization. Instead, comparison of these markers to a recent study on mammary macrophages (Jäppinen et al., 2019) revealed that M_a macrophages are enriched for key markers of fetal-derived macrophages, *Mrc1* (also known as *Cd206*) and *Adgre1* (also known as *F4/80*), whereas M_b macrophages are enriched for adult-derived macrophage markers, MHC II genes *H2-Aa*, *H2-Ab1*, *H2-DMa*, *H2-DMb1*, *H2-Eb1*, and *H2-Oa* (Figures 7F and S7C; Table S6). Therefore, M_a cells may represent fetal-derived macrophages arising from fetal yolk sac and liver, whereas M_b cells may represent adult-derived macrophages arising from the bone marrow. In addition, two recent studies proposed that mammary macrophages can be defined by their physical locations. Stromal macrophages (characterized as Mrc1/Cd206$^+$, Lyve1^{hi}, Itgax/Cd11c$^-$, and Vcam1^{lo}, or as Mrc1/Cd206^{hi}, Lyve1^{hi}, Cd209f^{hi}, and Cd209g^{hi}) survey the ECM and largely arise from fetal origin, whereas ductal macrophages (characterized as Mrc1/Cd206$^-$, Lyve1^{lo}, Itgax/Cd11c$^+$, Vcam1^{hi}, or as Mrc1/Cd206^{lo}, Lyve1^{lo}, Cd74^{hi}, H2-Ab1^{hi}) survey the epithelia and largely arise from adult bone marrow after puberty (Dawson et al., 2020; Wang et al., 2020). Notably, these gene signatures are very closely recapitulated by M_a and M_b macrophages, respectively, suggesting that M_a cells may represent stromal

(D) Dimensionality reduction plot from STREAM lineage trajectory analysis of luminal cells in young mice (n = 3). See also Figure S5D.

(E and F) Representative immunofluorescence staining of HS-AV cells (indicated by arrows) co-expressing HS marker (PR or ER) and AV marker (LTF or MFGE8) in young mice.

(G–J) Immunofluorescence staining of HS-AV cells (indicated by arrows) co-expressing PR and MFGE8 (G) or ER and LTF (I) in young and aged mice. Scale bar, 10 μm. Quantifications are shown in (H) and (J), respectively (n = 6 mice per age group, Student's t test). In (G) and (I), MFGE8 and LTF are also detected as secreted proteins in the lumen. KRT8 marks luminal cells.

(K) FACS quantification of HS-AV cells co-expressing HS marker CD133 and AV marker CD14 in an independent cohort of young (n = 8) and aged (n = 6) mice. In the representative FACS plots, 10,000 cells are displayed. Statistical significance is determined by Student's t test.

CellPress
OPEN ACCESS

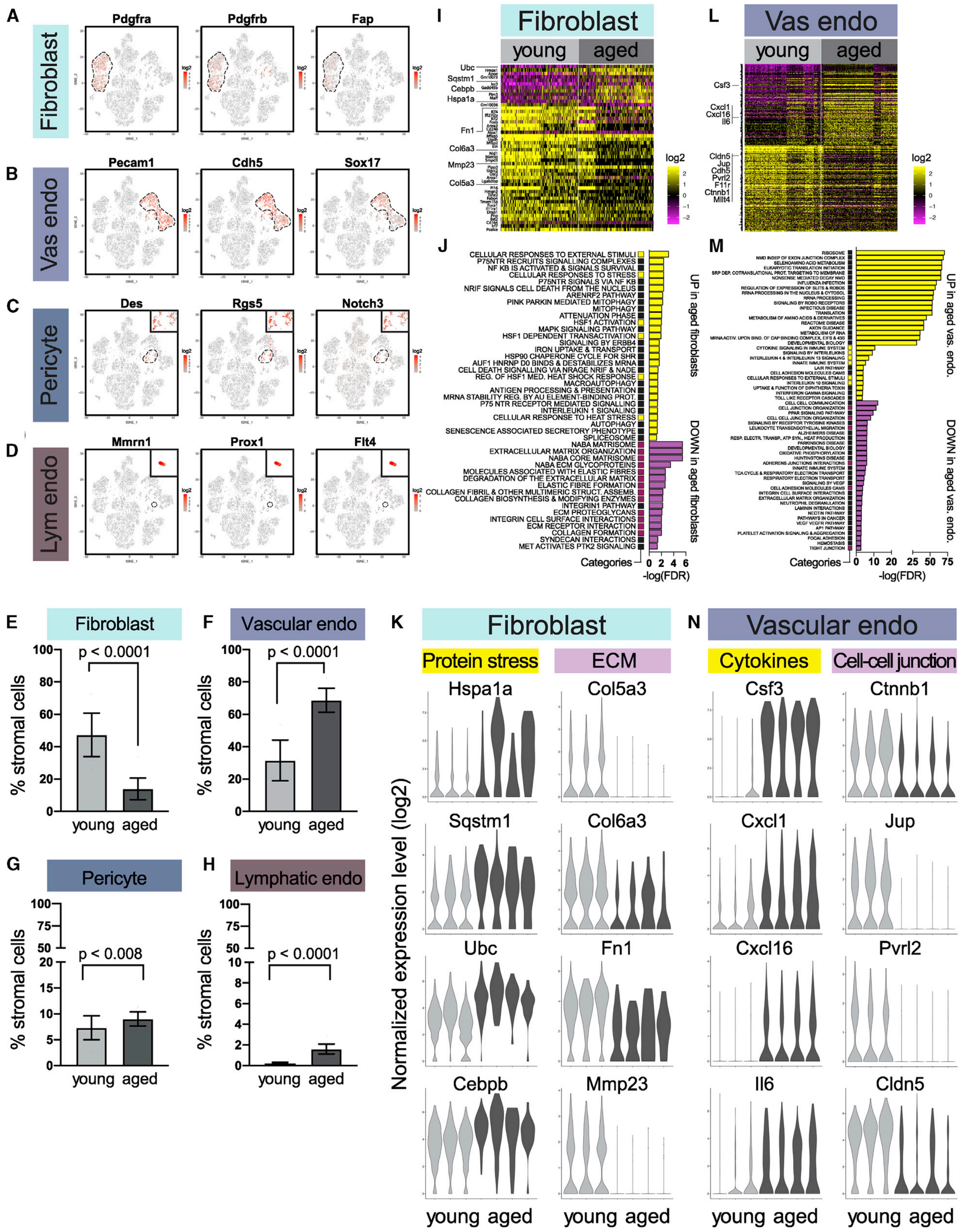

(legend on next page)

macrophages while M_b cells may be ductal macrophages (Figures S7D and S7E; Table S6). In our analysis, M_b macrophages are also distinguishable from M_a macrophages by higher expression of cell-surface antigens *Cd14*, *Cd52*, *Cd63*, *Cd72*, *Cd74*, and *Cd207*; cytokines *Il1a*, *Il1b*, *Il10*, *Il12b*, *Cxcl2*, and *Cxcl16*; and proteases *Mmp12*, *Mmp13*, *Ctss*, and *Ctsz* (Figure 7F). Their increased relative proportion with age is consistent with a replacement of fetal-derived macrophages by adult bone-marrow-derived macrophages as the mammary gland ages (Ginhoux and Guilliams, 2016; Hoeffel and Ginhoux, 2018). The recruitment of M_b macrophages may be mediated by the increased expression of *Csf1* and *Csf3* in aged myoepithelial cells and vascular endothelial cells (Figures 3E and 6N).

Aging induced a very modest number of gene expression changes in M_b macrophages compared with epithelial and non-immune stromal cell types, albeit more than other immune cells (Figure S3J), with 10 genes upregulated and four genes downregulated with age in a consistent manner across all samples (Figure 7G; Table S3). Although the number is very modest, it contains a few intriguing genes, including cytokines *Ccl5*, *Cxcl2*, and *Gdf15* (Figure 7H). CCL5 has been shown to recruit T cells (Arango Duque and Descoteaux, 2014); thus, its increased expression in aged M_b macrophages is consistent with the higher abundance of T cells observed in aged mammary glands. Furthermore, expression of two immunosuppressive ligands targeting T cells and NK cells also increased—*Cd274* (also known as *Pd-l1*) (Hsu et al., 2018; Lu et al., 2019; Yamazaki et al., 2002) and *Lilrb4* (also known as *Ilt3*) (Cella et al., 1997; Deng et al., 2018; Suciu-Foca and Cortesini, 2007) (Figure 7I)—thereby potentially promoting an immunosuppressive microenvironment. Taken together, these results highlight the diverse and dynamic immune landscape in aging mammary tissues.

DISCUSSION

To better understand the effects of aging on mammary glands, we have created a single-cell transcriptomic atlas to capture murine mammary epithelial and stromal changes associated with aging, corresponding to the transition from human early adulthood to perimenopause. Considering the longer human lifespan, there may be additional age-related changes not captured by a murine model. However, unlike human studies, mouse analyses can directly assess aging-specific alterations without confounding factors such as pregnancy history, hormone cycle, genetic background, diet, and carcinogen exposure. Importantly, the cell- and gene-level profiles identified herein are consistent across multiple biological replicates, suggesting that the intrinsic effects of aging may follow a defined developmental program instead of a stochastic degeneration.

Our scRNA-seq data indicate a potential shift of the luminal populations toward a more lactation-primed state in aging. Cell-type proportion analysis suggests a preferential expansion of AV cells relative to HS cells with age. Additionally, aged AV cells acquire higher expression of genes that are associated with milk production and are known to be upregulated in gestation (Bach et al., 2017). These changes are consistent with previous reports (Raafat et al., 2012) and our histological observation that aged nulliparous mice exhibit lobuloalveolar maturation, likely resulting from accumulated exposure to hormonal stimulation through multiple estrous cycles. Similar observation of age-dependent expansion of lobuloalveolar epithelium was reported in humans (Garbe et al., 2012; Hutson et al., 1985; Pelissier Vatter et al., 2018).

Other age-associated alterations detected by scRNA-seq may reflect a decline or dysregulation in cellular functions. For instance, aged myoepithelial cells downregulate genes known to mediate contractility and synthesize basement membrane (Haaksma et al., 2011; Ormerod et al., 1983; Sopel, 2010; Warburton et al., 1981; 1982), in part, consistent with previous reports that thinner and discontinuous basement membrane is associated with aging (Frantz et al., 2010; LaBarge et al., 2016). Similarly, aged fibroblasts decrease in relative abundance and ECM gene expression, suggesting an impaired ability to maintain a stromal matrix, thus consistent with previous reports of reduced connective tissue and mammographic density in older women (Gertig et al., 1999; Hart et al., 1989; Hutson et al., 1985; Li et al., 2005; McCormack et al., 2010; Unsworth et al., 2014). Additionally, aged endothelial cells downregulate expression of cell-cell junction components, potentially contributing to a compromised endothelial barrier and dysregulated vascular permeability (Bazzoni and Dejana, 2004). Vascular hyperpermeability is strongly associated with aging and is partly attributed to inflammatory cytokines, such as *Il6* and *Vegfa* (Donato et al., 2015; Oakley and Tharakan, 2014), which we observe to be upregulated in aged vascular endothelial cells and myoepithelial cells, respectively. Finally, aged mammary glands exhibit signs of pro-inflammatory microenvironment, with increased production of inflammatory cytokines (including *Cxcl1*, *Cxcl2*, *Cxcl16*, *Csfs1*, *Csf3*, *Ccl5*, and *Il6*) by aged myoepithelial cells, vascular endothelial cells, and macrophages and expanded proportions of bone-marrow-derived macrophages, NK cells, and T cells. These observations also suggest potential inter-cell-

Figure 6. Age-Dependent Alterations in Stromal Fibroblasts and Vascular and Lymphatic Cells
(A–D) Fibroblasts (A), vascular endothelial cells (B), pericytes (C), and lymphatic endothelial cells (D) are distinguished by characteristic makers in scRNA-seq data.
(E–H) Relative proportions of fibroblasts (E), vascular endothelial cells (F), pericytes (G), and lymphatic endothelial cells (H) in young (n = 3) and aged (n = 4) mice by scRNA-seq (Fisher's exact test).
(I) Differentially expressed genes in young versus aged fibroblasts. See also Table S3.
(J) Top gene sets identified by pathway analysis of differentially expressed genes in (I), with recurring gene sets highlighted. See also Table S4.
(K) Violin plots of select genes in (I) and (J) for fibroblasts.
(L) Differentially expressed genes in young versus aged vascular endothelial cells. See also Table S3.
(M) Top gene sets identified by pathway analysis of differentially expressed genes in (L), with recurring gene sets highlighted. See also Table S4.
(N) Violin plots of select genes in (L) and (M) for vascular endothelial cells.

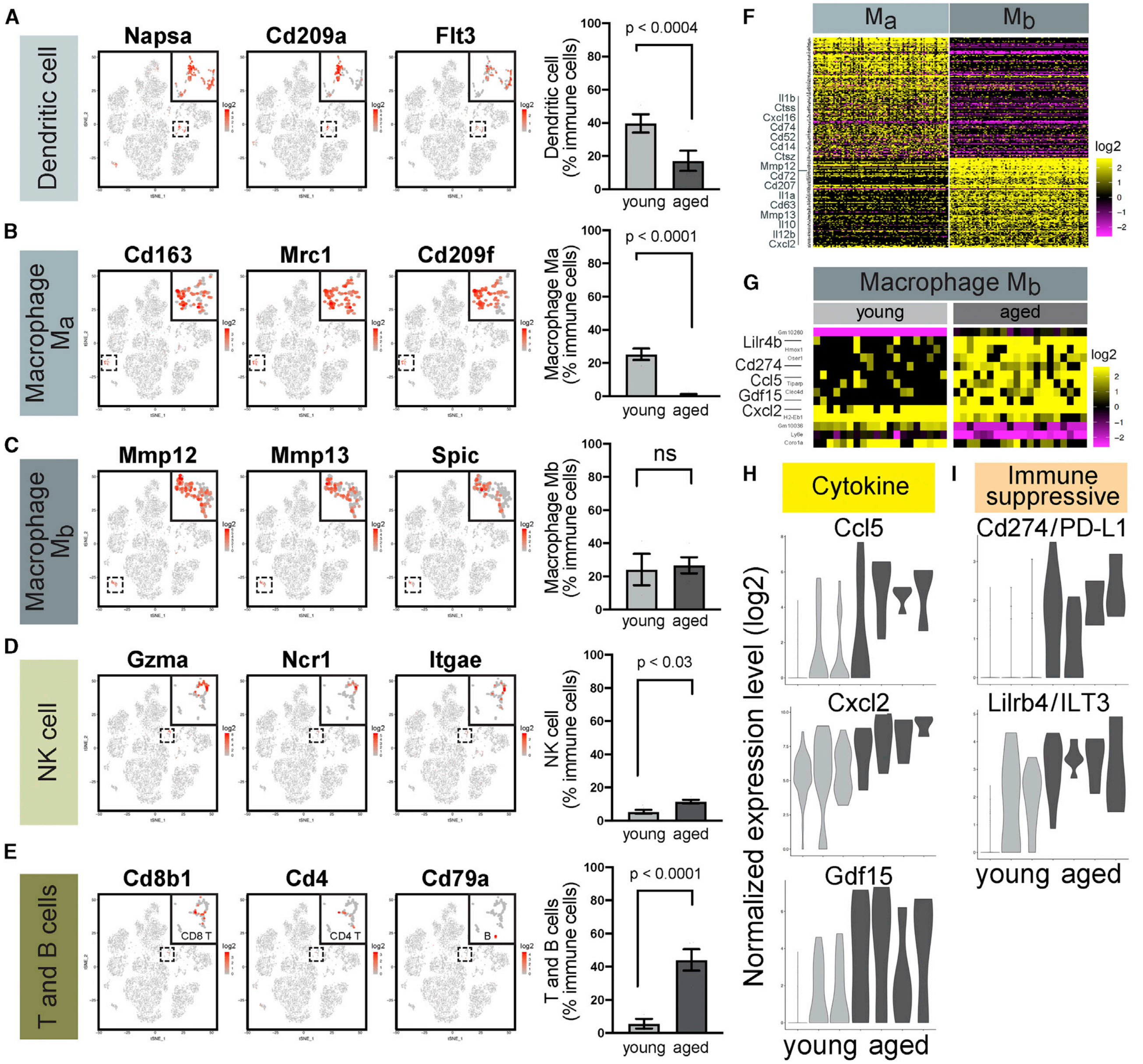

Figure 7. Age-Dependent Alterations in Myeloid and Lymphoid Immune Cells

(A–E) M_a and M_b macrophages, dendritic cells, natural killer (NK) cells, and T and B cells, are distinguished by their respective markers in scRNA-seq data (left). Their relative abundance in young (n = 3) and aged (n = 4) mice are shown on the right (Fisher's exact test).

(F) Genes distinguishing M_a and M_b macrophages. See also Table S6.

(G) Differentially expressed genes in young versus aged M_b macrophages. See also Table S3.

(H and I) Violin plots of cytokines (H) and immunosuppressive ligands (I) upregulated in aged M_b macrophages compared to young cells.

type crosstalk and recruitment, leading to systemic changes in aged mammary glands.

Given that aging is significantly correlated with breast cancer frequency in mice and human (Jenkins et al., 2014; LaBarge et al., 2016; Raafat et al., 2012), our findings may also provide initial insights into its underlying mechanisms. First, the expansion of AV luminal progenitor cells may pose a greater cancer risk because they have been proposed to be the cells of origin for triple-negative breast cancer (Lim et al., 2009; Molyneux et al., 2010; Proia et al., 2011). Second, aged myoepithelial cells display increased expression of *Jag1* and *Tgf*β, which can stimulate tumor proliferation and invasion (Cohen et al., 2010; Hu et al., 2008; Muraoka et al., 2003; Muraoka-Cook et al., 2004). Third, the downregulation of genes associated with contractility and basement membrane may impair the ability of myoepithelial cells to restrain malignant

luminal cell dissemination (Adriance et al., 2005; Pandey et al., 2010; Sirka et al., 2018). Previous studies have demonstrated that reduced myoepithelial cell contractility promotes malignant cell invasion (Sirka et al., 2018), whereas compromised basement membrane integrity is associated with breast cancer progression (Gusterson et al., 1982; Nakano et al., 1999; Natali et al., 1984; Tsubura et al., 1988). Loss of basement membrane has also been shown to promote abnormal luminal cell polarity, a hallmark of cancer (Gudjonsson et al., 2002). Moreover, increased vascular endothelial permeability, as suggested by reduced expression of cell-cell junction components, may further potentiate malignant cell dissemination (Maishi and Hida, 2017).

Additionally, aged mammary glands exhibit numerous signs of an inflammatory microenvironment that may promote the proliferation and metastasis of malignant cells (Greten and Grivennikov, 2019; Landskron et al., 2014; Zhang et al., 2018a). First, the expression of pro-inflammatory cytokines increased in aged myoepithelial cells, vascular endothelial cells, and macrophages. Second, the change in proportions of M_a and M_b macrophages suggests a replacement of fetal yolk sac and liver-derived macrophages by adult bone-marrow-derived cells with age. Bone-marrow-derived macrophages in murine mammary tumors have been associated with pro-tumorigenic properties by their ability to inhibit CD8 T cells (Franklin et al., 2014). In line with this, three key markers of bone-marrow-derived, tumor-associated macrophages described by Franklin et al. (2014), *Vcam1*, *Itgb5*, and *Itgax*, are also more highly expressed in M_b macrophages, suggesting potential similarities between M_b and adult-derived tumor-associated macrophages. Third, aged M_b macrophages upregulate expression of immunosuppressive ligands *Cd274/Pd-l1* and *Lilrb4/Ilt3*, thus, potentially inhibiting NK cells and T cells, creating a pro-tumor immune microenvironment (Lu et al., 2019). Increased inflammation with age was also previously observed in mouse mammary glands and other organs, but the specific source and mediators of inflammation remains incompletely understood (Dong et al., 2016; Sanada et al., 2018). Future studies may focus on the inflammatory cytokines and immune cell types enriched in aged mammary glands as detected herein to further delineate their exact contribution to tumor formation and progression. Of note, basal-like luminal cells have been reported in aged mice with mammary hyperplasia and in older women (Dong et al., 2016; Garbe et al., 2012; Pelissier Vatter et al., 2018), but are undetectable in our healthy aged mice, suggesting that these cells might represent a more-aberrant population that is usually absent in normal murine mammary glands.

In addition to age-related alterations, the identification of HS-AV cells is of considerable interest. These cells were also captured in two initial scRNA-seq studies of young murine mammary glands (Bach et al., 2017; Pal et al., 2017). Although Bach et al. (2017) did not comment on the hybrid nature of their gene expression, Pal et al. (2017) described them as luminal intermediates between the AV and HS lineages. Here, we verify the existence of HS-AV cells by tissue staining and FACS and define a gene signature distinguishing this population from other luminal cells. Furthermore, we find that HS-AV cells decrease in abundance with age, a potential consequence of the lack of self-renewal, differentiation into HS or AV lineage, or out-competition by HS or AV proliferation.

The lineage relationship of HS-AV cells to HS cells and AV cells remains to be determined. Lineage trajectory analysis suggests that HS-AV cells may be more progenitor-like than HS cells and AV cells. However, lineage tracing studies have demonstrated that postnatal HS and AV lineages are largely maintained independently by unipotent progenitors (Lilja et al., 2018; Van Keymeulen et al., 2017; Wang et al., 2017); thus, it is unlikely that, under normal homeostasis, HS-AV cells could function as bi-potent progenitors contributing to the HS and AV lineages. In contrast, it is more likely that HS-AV cells represent a distinct and restricted population, independent of HS cells and AV cells. Furthermore, depending on the rate of their cell division, it is possible that these rare HS-AV cells may not form clones of sufficient size to be detectable in sparse labeling and short-term lineage-tracing analyses.

If HS-AV cells are a stable population, then it raises the question of whether they serve a specific function in mammary glands. One possibility is that they might serve as dormant, resident, unipotent or bipotent luminal progenitors that can be activated during tissue regeneration. Similar examples of dormant progenitors have been described in other organs, such as lungs (Desai et al., 2014; Kim et al., 2005; Vaughan et al., 2015) and liver (Huch et al., 2013; Miyajima et al., 2014). Several observations provide a hint in support of that hypothesis. First, in scRNA-seq lineage trajectory analysis, HS-AV cells are localized to the bifurcation of the HS and AV branches, suggesting the possibility that they might be transcriptionally primed to give rise to cells in either lineage. Second, when compared with HS cells and AV cells, HS-AV cells are enriched for the expression of *Sox9*, a transcription factor reported to confer progenitor property (Guo et al., 2012). HS-AV cells also express other progenitor markers, such as *Kit*, *Aldh1a3*, and *Cd14*. Third, the increased expression of chromatin remodeling proteins in HS-AV cells compared with HS cells and AV cells might suggest a higher potential of differentiation plasticity, given the critical role chromatin modification has in regulating differentiation (Holliday et al., 2018; Wahl and Spike, 2017). Fourth, the diminished abundance of HS-AV cells with age is consistent with age-dependent depletion of stem/progenitor cells in certain tissue types (Encinas et al., 2011; Nishimura et al., 2005; Schultz and Sinclair, 2016). Finally, it remains possible that HS-AV cells represent a transient intermediate between the HS and AV populations, for instance, during differentiation of HS cells from AV cells, as proposed by Pal et al. (2017). However, evidence for such a transition has been limited, and further studies will shed light on that model. Overall, the identification of HS-AV cells suggests a greater complexity in mammary differentiation than was previously appreciated. Future lineage tracing and functional studies will be required to determine the roles of HS-AV cells in normal development and tissue regeneration and to elucidate their relationship with HS and AV lineages.

Taken together, these single-cell transcriptomic profiles reveal the dynamic heterogeneity in mammary tissues at young and old age. They provide a resource for future studies to understand the interactions between epithelial and stromal cells in aging and cancer.

STAR★METHODS

Detailed methods are provided in the online version of this paper and include the following:

- KEY RESOURCES TABLE
- RESOURCE AVAILABILITY
  - Lead Contact
  - Materials Availability
  - Data and Code Availability
- EXPERIMENTAL MODEL AND SUBJECT DETAILS
- METHOD DETAILS
  - Tissue dissociation for single-cell RNA-seq
  - Single-cell RNA library preparation and sequencing
  - Single-cell RNA-seq data processing
  - Cell type-specific gene signatures
  - Differential gene expression across age groups
  - Pathway analysis
  - Histology and immunostaining
  - FACS analysis
- QUANTIFICATION AND STATISTICAL ANALYSIS

SUPPLEMENTAL INFORMATION

Supplemental Information can be found online at https://doi.org/10.1016/j.celrep.2020.108566.

ACKNOWLEDGMENTS

We thank Dr. Aviv Regev's laboratory (Broad Institute), including Danielle Dionne, Lan Nguyen, Julia Waldman, Michael Cuoco, Michal Slyper, Christopher Rodman, Orr Ashenberg, Marcin Tabaka, and Orit Rozenblatt-Rosen, as well as Dr. Asaf Rotem (Dana-Farber Cancer Institute) for scRNA-seq assistance. We thank Dr. Roderick Bronson (Harvard Medical School) for histopathology consultation, the Dana-Farber Flow Cytometry Core for FACS service, the Harvard Nikon Imaging Center for microscopy support, Dr. Lai Ding (NeuroTechnology Studio, Brigham and Women's Hospital) for image analysis consultation and assistance, and all members of the Brugge laboratory, especially Jason Zoeller, Nomeda Girnius, Mackenzie Boedicker, and Angie Martinez Gakidis for assistance in conducting this study and preparing the manuscript. We also thank Dr. Marie Bao for comments on the manuscript. This work was supported in part by funding from a Susan G. Komen postdoctoral fellowship (C.M.L.); a Croucher postdoctoral fellowship (C.M.L.); an NIH F31 fellowship (G.K.G.); a Hope Funds postdoctoral fellowship (Y.O.); Marsha Rivkin Center for Ovarian Cancer Research (Y.O.); the Chan Zuckerberg Initiative Donor-Advised Fund (grant number 2018-182734 to L.P.); an advised fund of the Silicon Valley Community Foundation (L.P.); NHGRI Career Development Award and Genome Innovator Award (R00HG008399 and R35HG010717 to L.P.); the Susan G. Komen Breast Cancer Foundation (J.S.B.); and the Breast Cancer Research Foundation (J.S.B.).

AUTHOR CONTRIBUTIONS

C.M.L. and J.S.B. conceived the study and designed the experiments. C.M.L. performed the experiments and analyses with assistance from G.K.G. for tissue dissociation; A.R. for scRNA-seq; L.M.S., H.C., L.P., Y.O., and M.J.S. for bioinformatics analyses; H.S., C.T., and K.P.G. for tissue staining and microscopy; and H.S., J.R., and K.M. for FACS analysis of tissues and organoids. All authors contributed to the interpretation of experimental results. C.M.L. and J.S.B. wrote the manuscript, with contribution from all authors. J.S.B. provided funding and project supervision.

DECLARATION OF INTERESTS

J.L.G. is a consultant for Glaxo-Smith Kline (GSK), Codagenix, Verseau, Kymera, and Array BioPharma and receives sponsored research support from GSK, Array BioPharma, and Eli Lilly. A.R. is a founder and equity holder of Celsius Therapeutics, an equity holder in Immunitas Therapeutics, and until August 31, 2020, was on the science advisory boards (SABs) of Syros Pharmaceuticals, Neogene Therapeutics, Asimov, and ThermoFisher Scientific. From August 1, 2020, A.R. is an employee of Genentech, a member of the Roche Group. J.S.B. is on the SAB of Agios Pharmaceuticals, eFFECTOR Therapeutics, and Frontier Medicines.

Received: June 24, 2020
Revised: October 13, 2020
Accepted: December 7, 2020
Published: December 29, 2020

REFERENCES

Ace, C.I., and Okulicz, W.C. (1998). A progesterone-induced endometrial homolog of a new candidate tumor suppressor, DMBT1. J. Clin. Endocrinol. Metab. *83*, 3569–3573.

Adriance, M.C., Inman, J.L., Petersen, O.W., and Bissell, M.J. (2005). Myoepithelial cells: good fences make good neighbors. Breast Cancer Res. *7*, 190–197.

Agarwal, A.K., Tunison, K., Dalal, J.S., Nagamma, S.S., Hamra, F.K., Sankella, S., Shao, X., Auchus, R.J., and Garg, A. (2017). Metabolic, Reproductive, and Neurologic Abnormalities in Agpat1-Null Mice. Endocrinology *158*, 3954–3973.

Arango Duque, G., and Descoteaux, A. (2014). Macrophage cytokines: involvement in immunity and infectious diseases. Front. Immunol. *5*, 491.

Azam, S., Sjölander, A., Eriksson, M., Gabrielson, M., Czene, K., and Hall, P. (2019). Determinants of mammographic density change. JNCI Cancer Spectr. *3*, pkz004.

Bach, K., Pensa, S., Grzelak, M., Hadfield, J., Adams, D.J., Marioni, J.C., and Khaled, W.T. (2017). Differentiation dynamics of mammary epithelial cells revealed by single-cell RNA sequencing. Nat. Commun. *8*, 2128.

Bazzoni, G., and Dejana, E. (2004). Endothelial cell-to-cell junctions: molecular organization and role in vascular homeostasis. Physiol. Rev. *84*, 869–901.

Bjørge, L., Jensen, T.S., Kristoffersen, E.K., Ulstein, M., and Matre, R. (1996). Identification of the complement regulatory protein CD59 in human colostrum and milk. Am. J. Reprod. Immunol. *35*, 43–50.

Blencowe, B.J., Issner, R., Nickerson, J.A., and Sharp, P.A. (1998). A coactivator of pre-mRNA splicing. Genes Dev. *12*, 996–1009.

Booth, B.W., Boulanger, C.A., and Smith, G.H. (2007). Alveolar progenitor cells develop in mouse mammary glands independent of pregnancy and lactation. J. Cell. Physiol. *212*, 729–736.

Brady, N.J., Chuntova, P., and Schwertfeger, K.L. (2016). Macrophages: regulators of the inflammatory microenvironment during mammary gland development and breast cancer. Mediators Inflamm. *2016*, 4549676.

Brisken, C., and Ataca, D. (2015). Endocrine hormones and local signals during the development of the mouse mammary gland. Wiley Interdiscip. Rev. Dev. Biol. *4*, 181–195.

Bronte, V., and Zanovello, P. (2005). Regulation of immune responses by L-arginine metabolism. Nat. Rev. Immunol. *5*, 641–654.

Burchfield, J.S., Li, Q., Wang, H.Y., and Wang, R.-F. (2015). JMJD3 as an epigenetic regulator in development and disease. Int. J. Biochem. Cell Biol. *67*, 148–157.

Butler, A., Hoffman, P., Smibert, P., Papalexi, E., and Satija, R. (2018). Integrating single-cell transcriptomic data across different conditions, technologies, and species. Nat. Biotechnol. *36*, 411–420.

Cella, M., Döhring, C., Samaridis, J., Dessing, M., Brockhaus, M., Lanzavecchia, A., and Colonna, M. (1997). A novel inhibitory receptor (ILT3) expressed

on monocytes, macrophages, and dendritic cells involved in antigen processing. J. Exp. Med. *185*, 1743–1751.

Chen, H., Albergante, L., Hsu, J.Y., Lareau, C.A., Lo Bosco, G., Guan, J., Zhou, S., Gorban, A.N., Bauer, D.E., Aryee, M.J., et al. (2019). Single-cell trajectories reconstruction, exploration and mapping of omics data with STREAM. Nat. Commun. *10*, 1903–1914.

Clarke, R.B., Howell, A., Potten, C.S., and Anderson, E. (1997). Dissociation between steroid receptor expression and cell proliferation in the human breast. Cancer Res. *57*, 4987–4991.

Cohen, B., Shimizu, M., Izrailit, J., Ng, N.F.L., Buchman, Y., Pan, J.G., Dering, J., and Reedijk, M. (2010). Cyclin D1 is a direct target of JAG1-mediated Notch signaling in breast cancer. Breast Cancer Res. Treat. *123*, 113–124.

Crisà, A., Ferrè, F., Chillemi, G., and Moioli, B. (2016). RNA-sequencing for profiling goat milk transcriptome in colostrum and mature milk. BMC Vet. Res. *12*, 264.

Dawson, C.A., Pal, B., Vaillant, F., Gandolfo, L.C., Liu, Z., Bleriot, C., Ginhoux, F., Smyth, G.K., Lindeman, G.J., Mueller, S.N., et al. (2020). Tissue-resident ductal macrophages survey the mammary epithelium and facilitate tissue remodelling. Nat. Cell Biol. *22*, 546–558.

Deng, M., Gui, X., Kim, J., Xie, L., Chen, W., Li, Z., He, L., Chen, Y., Chen, H., Luo, W., et al. (2018). LILRB4 signalling in leukaemia cells mediates T cell suppression and tumour infiltration. Nature *562*, 605–609.

Denissov, S., Hofemeister, H., Marks, H., Kranz, A., Ciotta, G., Singh, S., Anastassiadis, K., Stunnenberg, H.G., and Stewart, A.F. (2014). Mll2 is required for H3K4 trimethylation on bivalent promoters in embryonic stem cells, whereas Mll1 is redundant. Development *141*, 526–537.

Desai, T.J., Brownfield, D.G., and Krasnow, M.A. (2014). Alveolar progenitor and stem cells in lung development, renewal and cancer. Nature *507*, 190–194.

Diaz Brinton, R. (2012). Minireview: translational animal models of human menopause: challenges and emerging opportunities. Endocrinology *153*, 3571–3578.

Donato, A.J., Morgan, R.G., Walker, A.E., and Lesniewski, L.A. (2015). Cellular and molecular biology of aging endothelial cells. J. Mol. Cell. Cardiol. *89* (*Pt B*), 122–135.

Dong, Q., Gao, H., Shi, Y., Zhang, F., Gu, X., Wu, A., Wang, D., Chen, Y., Bandyopadhyay, A., Yeh, I.-T., et al. (2016). Aging is associated with an expansion of CD49f^{hi} mammary stem cells that show a decline in function and increased transformation potential. Aging (Albany NY) *8*, 2754–2776.

Dos Santos, C.O., Dolzhenko, E., Hodges, E., Smith, A.D., and Hannon, G.J. (2015). An epigenetic memory of pregnancy in the mouse mammary gland. Cell Rep. *11*, 1102–1109.

Encinas, J.M., Michurina, T.V., Peunova, N., Park, J.-H., Tordo, J., Peterson, D.A., Fishell, G., Koulakov, A., and Enikolopov, G. (2011). Division-coupled astrocytic differentiation and age-related depletion of neural stem cells in the adult hippocampus. Cell Stem Cell *8*, 566–579.

Fata, J.E., Chaudhary, V., and Khokha, R. (2001). Cellular turnover in the mammary gland is correlated with systemic levels of progesterone and not 17β-estradiol during the estrous cycle. Biol. Reprod. *65*, 680–688.

Finch, C.E., Felicio, L.S., Mobbs, C.V., and Nelson, J.F. (1984). Ovarian and steroidal influences on neuroendocrine aging processes in female rodents. Endocr. Rev. *5*, 467–497.

Flurkey, K., Currer, J.M., and Harrison, D.E. (2007). The Mouse in Biomedical Research: Normative Biology, Husbandry, and ModelsSecond Edition, *Volume 3* (Elsevier).

Franklin, R.A., Liao, W., Sarkar, A., Kim, M.V., Bivona, M.R., Liu, K., Pamer, E.G., and Li, M.O. (2014). The cellular and molecular origin of tumor-associated macrophages. Science *344*, 921–925.

Frantz, C., Stewart, K.M., and Weaver, V.M. (2010). The extracellular matrix at a glance. J. Cell Sci. *123*, 4195–4200.

Froimchuk, E., Jang, Y., and Ge, K. (2017). Histone H3 lysine 4 methyltransferase KMT2D. Gene *627*, 337–342.

Fu, N.Y., Nolan, E., Lindeman, G.J., and Visvader, J.E. (2020). Stem cells and the differentiation hierarchy in mammary gland development. Physiol. Rev. *100*, 489–523.

Gannon, P.O., Godin-Ethier, J., Hassler, M., Delvoye, N., Aversa, M., Poisson, A.O., Péant, B., Alam Fahmy, M., Saad, F., Lapointe, R., and Mes-Masson, A.M. (2010). Androgen-regulated expression of arginase 1, arginase 2 and interleukin-8 in human prostate cancer. PLoS ONE *5*, e12107.

Gantt, S., Gervassi, A., Jaspan, H., and Horton, H. (2014). The role of myeloid-derived suppressor cells in immune ontogeny. Front. Immunol. *5*, 387.

Garbe, J.C., Pepin, F., Pelissier, F.A., Sputova, K., Fridriksdottir, A.J., Guo, D.E., Villadsen, R., Park, M., Petersen, O.W., Borowsky, A.D., et al. (2012). Accumulation of multipotent progenitors with a basal differentiation bias during aging of human mammary epithelia. Cancer Res. *72*, 3687–3701.

Gertig, D.M., Stillman, I.E., Byrne, C., Spiegelman, D., Schnitt, S.J., Connolly, J.L., Colditz, G.A., and Hunter, D.J. (1999). Association of age and reproductive factors with benign breast tissue composition. Cancer Epidemiol. Biomarkers Prev. *8*, 873–879.

Ginger, M.R., and Rosen, J.M. (2003). Pregnancy-induced changes in cell-fate in the mammary gland. Breast Cancer Res. *5*, 192–197.

Ginhoux, F., and Guilliams, M. (2016). Tissue-resident macrophage ontogeny and homeostasis. Immunity *44*, 439–449.

Giraddi, R.R., Shehata, M., Gallardo, M., Blasco, M.A., Simons, B.D., and Stingl, J. (2015). Stem and progenitor cell division kinetics during postnatal mouse mammary gland development. Nat. Commun. *6*, 8487.

Giraddi, R.R., Chung, C.-Y., Heinz, R.E., Balcioglu, O., Novotny, M., Trejo, C.L., Dravis, C., Hagos, B.M., Mehrabad, E.M., Rodewald, L.-W., et al. (2018). Single-cell transcriptomes distinguish stem cell state changes and lineage specification programs in early mammary gland development. Cell Rep. *24*, 1653–1666.e1657.

Gouon-Evans, V., Lin, E.Y., and Pollard, J.W. (2002). Requirement of macrophages and eosinophils and their cytokines/chemokines for mammary gland development. Breast Cancer Res. *4*, 155–164.

Greten, F.R., and Grivennikov, S.I. (2019). Inflammation and cancer: triggers, mechanisms, and consequences. Immunity *51*, 27–41.

Gudjonsson, T., Rønnov-Jessen, L., Villadsen, R., Rank, F., Bissell, M.J., and Petersen, O.W. (2002). Normal and tumor-derived myoepithelial cells differ in their ability to interact with luminal breast epithelial cells for polarity and basement membrane deposition. J. Cell Sci. *115*, 39–50.

Guerriero, J.L. (2018). Macrophages: the road less traveled, changing anti-cancer therapy. Trends Mol. Med. *24*, 472–489.

Gundlah, C., Alves, S.E., Clark, J.A., Pai, L.-Y., Schaeffer, J.M., and Rohrer, S.P. (2005). Estrogen receptor-beta regulates tryptophan hydroxylase-1 expression in the murine midbrain raphe. Biol. Psychiatry *57*, 938–942.

Guo, W., Keckesova, Z., Donaher, J.L., Shibue, T., Tischler, V., Reinhardt, F., Itzkovitz, S., Noske, A., Zürrer-Härdi, U., Bell, G., et al. (2012). Slug and Sox9 cooperatively determine the mammary stem cell state. Cell *148*, 1015–1028.

Gusterson, B.A., Warburton, M.J., Mitchell, D., Ellison, M., Neville, A.M., and Rudland, P.S. (1982). Distribution of myoepithelial cells and basement membrane proteins in the normal breast and in benign and malignant breast diseases. Cancer Res. *42*, 4763–4770.

Haaksma, C.J., Schwartz, R.J., and Tomasek, J.J. (2011). Myoepithelial cell contraction and milk ejection are impaired in mammary glands of mice lacking smooth muscle alpha-actin. Biol. Reprod. *85*, 13–21.

Halper, J., and Kjaer, M. (2014). Basic components of connective tissues and extracellular matrix: elastin, fibrillin, fibulins, fibrinogen, fibronectin, laminin, tenascins and thrombospondins. Adv. Exp. Med. Biol. *802*, 31–47.

Han, X., Wang, R., Zhou, Y., Fei, L., Sun, H., Lai, S., Saadatpour, A., Zhou, Z., Chen, H., Ye, F., et al. (2018). Mapping the mouse cell atlas by Microwell-seq. Cell *172*, 1091–1107.e17.

Hart, B.L., Steinbock, R.T., Mettler, F.A., Jr., Pathak, D.R., and Bartow, S.A. (1989). Age and race related changes in mammographic parenchymal patterns. Cancer *63*, 2537–2539.

Hickey, D.K., Fahey, J.V., and Wira, C.R. (2013). Estrogen receptor α antagonists mediate changes in CCL20 and CXCL1 secretions in the murine female reproductive tract. Am. J. Reprod. Immunol. *69*, 159–167.

Hoeffel, G., and Ginhoux, F. (2018). Fetal monocytes and the origins of tissue-resident macrophages. Cell. Immunol. *330*, 5–15.

Holliday, H., Baker, L.A., Junankar, S.R., Clark, S.J., and Swarbrick, A. (2018). Epigenomics of mammary gland development. Breast Cancer Res. *20*, 100–111.

Hsu, J., Hodgins, J.J., Marathe, M., Nicolai, C.J., Bourgeois-Daigneault, M.-C., Trevino, T.N., Azimi, C.S., Scheer, A.K., Randolph, H.E., Thompson, T.W., et al. (2018). Contribution of NK cells to immunotherapy mediated by PD-1/PD-L1 blockade. J. Clin. Invest. *128*, 4654–4668.

Hu, M., Yao, J., Carroll, D.K., Weremowicz, S., Chen, H., Carrasco, D., Richardson, A., Violette, S., Nikolskaya, T., Nikolsky, Y., et al. (2008). Regulation of in situ to invasive breast carcinoma transition. Cancer Cell *13*, 394–406.

Huch, M., Dorrell, C., Boj, S.F., van Es, J.H., Li, V.S.W., van de Wetering, M., Sato, T., Hamer, K., Sasaki, N., Finegold, M.J., et al. (2013). In vitro expansion of single $Lgr5^+$ liver stem cells induced by Wnt-driven regeneration. Nature *494*, 247–250.

Huh, S.J., Clement, K., Jee, D., Merlini, A., Choudhury, S., Maruyama, R., Yoo, R., Chytil, A., Boyle, P., Ran, F.A., et al. (2015). Age- and pregnancy-associated DNA methylation changes in mammary epithelial cells. Stem Cell Reports *4*, 297–311.

Hutson, S.W., Cowen, P.N., and Bird, C.C. (1985). Morphometric studies of age related changes in normal human breast and their significance for evolution of mammary cancer. J. Clin. Pathol. *38*, 281–287.

Inman, J.L., Robertson, C., Mott, J.D., and Bissell, M.J. (2015). Mammary gland development: cell fate specification, stem cells and the microenvironment. Development *142*, 1028–1042.

Jahchan, N.S., You, Y.-H., Muller, W.J., and Luo, K. (2010). Transforming growth factor-beta regulator SnoN modulates mammary gland branching morphogenesis, postlactational involution, and mammary tumorigenesis. Cancer Res. *70*, 4204–4213.

Jahchan, N.S., Wang, D., Bissell, M.J., and Luo, K. (2012). SnoN regulates mammary gland alveologenesis and onset of lactation by promoting prolactin/Stat5 signaling. Development *139*, 3147–3156.

Jäppinen, N., Félix, I., Lokka, E., Tyystjärvi, S., Pynttäri, A., Lahtela, T., Gerke, H., Elima, K., Rantakari, P., and Salmi, M. (2019). Fetal-derived macrophages dominate in adult mammary glands. Nat. Commun. *10*, 281.

Jenkins, E.O., Deal, A.M., Anders, C.K., Prat, A., Perou, C.M., Carey, L.A., and Muss, H.B. (2014). Age-specific changes in intrinsic breast cancer subtypes: a focus on older women. Oncologist *19*, 1076–1083.

Joshi, P.A., Jackson, H.W., Beristain, A.G., Di Grappa, M.A., Mote, P.A., Clarke, C.L., Stingl, J., Waterhouse, P.D., and Khokha, R. (2010). Progesterone induces adult mammary stem cell expansion. Nature *465*, 803–807.

Kamikawa, A., Ichii, O., Yamaji, D., Imao, T., Suzuki, C., Okamatsu-Ogura, Y., Terao, A., Kon, Y., and Kimura, K. (2009). Diet-induced obesity disrupts ductal development in the mammary glands of nonpregnant mice. Dev. Dyn. *238*, 1092–1099.

Kanaya, N., Chang, G., Wu, X., Saeki, K., Bernal, L., Shim, H.-J., Wang, J., Warden, C., Yamamoto, T., Li, J., et al. (2019). Single-cell RNA-sequencing analysis of estrogen- and endocrine-disrupting chemical-induced reorganization of mouse mammary gland. Commun. Biol. *2*, 406–415.

Kankel, K.F., and Reinauer, H. (1976). Activity of pyruvate dehydrogenase complex in the mammary gland of normal and diabetic rats. Diabetologia *12*, 149–154.

Kim, C.F., Jackson, E.L., Woolfenden, A.E., Lawrence, S., Babar, I., Vogel, S., Crowley, D., Bronson, R.T., and Jacks, T. (2005). Identification of bronchioalveolar stem cells in normal lung and lung cancer. Cell *121*, 823–835.

Kim, Y.-E., Won, M., Lee, S.-G., Park, C., Song, C.-H., and Kim, K.K. (2019). RBM47-regulated alternative splicing of TJP1 promotes actin stress fiber assembly during epithelial-to-mesenchymal transition. Oncogene *38*, 6521–6536.

Kzhyshkowska, J., Rusch, A., Wolf, H., and Dobner, T. (2003). Regulation of transcription by the heterogeneous nuclear ribonucleoprotein E1B-AP5 is mediated by complex formation with the novel bromodomain-containing protein BRD7. Biochem. J. *371*, 385–393.

LaBarge, M.A., Mora-Blanco, E.L., Samson, S., and Miyano, M. (2016). Breast cancer beyond the age of mutation. Gerontology *62*, 434–442.

Landskron, G., De la Fuente, M., Thuwajit, P., Thuwajit, C., and Hermoso, M.A. (2014). Chronic inflammation and cytokines in the tumor microenvironment. J. Immunol. Res. *2014*, 149185.

Laporta, J., Keil, K.P., Vezina, C.M., and Hernandez, L.L. (2014). Peripheral serotonin regulates maternal calcium trafficking in mammary epithelial cells during lactation in mice. PLoS ONE *9*, e110190.

Li, T., Sun, L., Miller, N., Nicklee, T., Woo, J., Hulse-Smith, L., Tsao, M.-S., Khokha, R., Martin, L., and Boyd, N. (2005). The association of measured breast tissue characteristics with mammographic density and other risk factors for breast cancer. Cancer Epidemiol. Biomarkers Prev. *14*, 343–349.

Liberzon, A., Birger, C., Thorvaldsdóttir, H., Ghandi, M., Mesirov, J.P., and Tamayo, P. (2015). The Molecular Signatures Database (MSigDB) hallmark gene set collection. Cell Syst. *1*, 417–425.

Lilja, A.M., Rodilla, V., Huyghe, M., Hannezo, E., Landragin, C., Renaud, O., Leroy, O., Rulands, S., Simons, B.D., and Fre, S. (2018). Clonal analysis of Notch1-expressing cells reveals the existence of unipotent stem cells that retain long-term plasticity in the embryonic mammary gland. Nat. Cell Biol. *20*, 677–687.

Lim, E., Vaillant, F., Wu, D., Forrest, N.C., Pal, B., Hart, A.H., Asselin-Labat, M.-L., Gyorki, D.E., Ward, T., Partanen, A., et al.; kConFab (2009). Aberrant luminal progenitors as the candidate target population for basal tumor development in BRCA1 mutation carriers. Nat. Med. *15*, 907–913.

Littlejohn, M.D., Walker, C.G., Ward, H.E., Lehnert, K.B., Snell, R.G., Verkerk, G.A., Spelman, R.J., Clark, D.A., and Davis, S.R. (2010). Effects of reduced frequency of milk removal on gene expression in the bovine mammary gland. Physiol. Genomics *41*, 21–32.

Lomniczi, A., Wright, H., Castellano, J.M., Matagne, V., Toro, C.A., Ramaswamy, S., Plant, T.M., and Ojeda, S.R. (2015). Epigenetic regulation of puberty via Zinc finger protein-mediated transcriptional repression. Nat. Commun. *6*, 10195.

Lu, D., Ni, Z., Liu, X., Feng, S., Dong, X., Shi, X., Zhai, J., Mai, S., Jiang, J., Wang, Z., et al. (2019). Beyond T cells: understanding the role of PD-1/PD-L1 in tumor-associated macrophages. J. Immunol. Res. *2019*, 1919082–1919087.

Lv, Y., Guan, W., Qiao, H., Wang, C., Chen, F., Zhang, Y., and Liao, Z. (2015). Veterinary medicine and omics (veterinomics): metabolic transition of milk triacylglycerol synthesis in sows from late pregnancy to lactation. OMICS *19*, 602–616.

Maishi, N., and Hida, K. (2017). Tumor endothelial cells accelerate tumor metastasis. Cancer Sci. *108*, 1921–1926.

Malhotra, G.K., Zhao, X., Edwards, E., Kopp, J.L., Naramura, M., Sander, M., Band, H., and Band, V. (2014). The role of Sox9 in mouse mammary gland development and maintenance of mammary stem and luminal progenitor cells. BMC Dev. Biol. *14*, 47.

Mas, G., Blanco, E., Ballaré, C., Sansó, M., Spill, Y.G., Hu, D., Aoi, Y., Le Dily, F., Shilatifard, A., Marti-Renom, M.A., and Di Croce, L. (2018). Promoter bivalency favors an open chromatin architecture in embryonic stem cells. Nat. Genet. *50*, 1452–1462.

Matsuda, M., Imaoka, T., Vomachka, A.J., Gudelsky, G.A., Hou, Z., Mistry, M., Bailey, J.P., Nieport, K.M., Walther, D.J., Bader, M., and Horseman, N.D. (2004). Serotonin regulates mammary gland development via an autocrine-paracrine loop. Dev. Cell *6*, 193–203.

McCormack, V.A., Perry, N.M., Vinnicombe, S.J., and Dos Santos Silva, I. (2010). Changes and tracking of mammographic density in relation to Pike's model of breast tissue aging: a UK longitudinal study. Int. J. Cancer *127*, 452–461.

McCormick, N.H., Hennigar, S.R., Kiselyov, K., and Kelleher, S.L. (2014). The biology of zinc transport in mammary epithelial cells: implications for mammary gland development, lactation, and involution. J. Mammary Gland Biol. Neoplasia *19*, 59–71.

McGinnis, C.S., Murrow, L.M., and Gartner, Z.J. (2019). DoubletFinder: doublet detection in single-cell RNA sequencing data using artificial nearest neighbors. Cell Syst. *8*, 329–337.e4.

Miyajima, A., Tanaka, M., and Itoh, T. (2014). Stem/progenitor cells in liver development, homeostasis, regeneration, and reprogramming. Cell Stem Cell *14*, 561–574.

Molyneux, G., Geyer, F.C., Magnay, F.-A., McCarthy, A., Kendrick, H., Natrajan, R., Mackay, A., Grigoriadis, A., Tutt, A., Ashworth, A., et al. (2010). BRCA1 basal-like breast cancers originate from luminal epithelial progenitors and not from basal stem cells. Cell Stem Cell *7*, 403–417.

Muraoka, R.S., Koh, Y., Roebuck, L.R., Sanders, M.E., Brantley-Sieders, D., Gorska, A.E., Moses, H.L., and Arteaga, C.L. (2003). Increased malignancy of Neu-induced mammary tumors overexpressing active transforming growth factor beta1. Mol. Cell. Biol. *23*, 8691–8703.

Muraoka-Cook, R.S., Kurokawa, H., Koh, Y., Forbes, J.T., Roebuck, L.R., Barcellos-Hoff, M.H., Moody, S.E., Chodosh, L.A., and Arteaga, C.L. (2004). Conditional overexpression of active transforming growth factor beta1 in vivo accelerates metastases of transgenic mammary tumors. Cancer Res. *64*, 9002–9011.

Nakano, S., Iyama, K., Ogawa, M., Yoshioka, H., Sado, Y., Oohashi, T., and Ninomiya, Y. (1999). Differential tissular expression and localization of type IV collagen alpha1(IV), alpha2(IV), alpha5(IV), and alpha6(IV) chains and their mRNA in normal breast and in benign and malignant breast tumors. Lab. Invest. *79*, 281–292.

Natali, P.G., Giacomini, P., Bigotti, G., Nicotra, M.R., Bellocci, M., and De Martino, C. (1984). Heterogeneous distribution of actin, myosin, fibronectin and basement membrane antigens in primary and metastatic human breast cancer. Virchows Arch. A Pathol. Anat. Histopathol. *405*, 69–83.

Nguyen, Q.H., Pervolarakis, N., Blake, K., Ma, D., Davis, R.T., James, N., Phung, A.T., Willey, E., Kumar, R., Jabart, E., et al. (2018). Profiling human breast epithelial cells using single cell RNA sequencing identifies cell diversity. Nat. Commun. *9*, 2028.

Nishimura, E.K., Granter, S.R., and Fisher, D.E. (2005). Mechanisms of hair graying: incomplete melanocyte stem cell maintenance in the niche. Science *307*, 720–724.

Oakley, R., and Tharakan, B. (2014). Vascular hyperpermeability and aging. Aging Dis. *5*, 114–125.

Ono, Y.J., Terai, Y., Tanabe, A., Hayashi, A., Hayashi, M., Yamashita, Y., Kyo, S., and Ohmichi, M. (2014). Decorin induced by progesterone plays a crucial role in suppressing endometriosis. J. Endocrinol. *223*, 203–216.

Ormerod, E.J., Warburton, M.J., Hughes, C., and Rudland, P.S. (1983). Synthesis of basement membrane proteins by rat mammary epithelial cells. Dev. Biol. *96*, 269–275.

Pal, B., Chen, Y., Vaillant, F., Jamieson, P., Gordon, L., Rios, A.C., Wilcox, S., Fu, N., Liu, K.H., Jackling, F.C., et al. (2017). Construction of developmental lineage relationships in the mouse mammary gland by single-cell RNA profiling. Nat. Commun. *8*, 1627.

Pandey, P.R., Saidou, J., and Watabe, K. (2010). Role of myoepithelial cells in breast tumor progression. Front. Biosci. *15*, 226–236.

Pankov, R., and Yamada, K.M. (2002). Fibronectin at a glance. J. Cell Sci. *115*, 3861–3863.

Pelissier, F.A., Garbe, J.C., Ananthanarayanan, B., Miyano, M., Lin, C., Jokela, T., Kumar, S., Stampfer, M.R., Lorens, J.B., and LaBarge, M.A. (2014). Age-related dysfunction in mechanotransduction impairs differentiation of human mammary epithelial progenitors. Cell Rep. *7*, 1926–1939.

Pelissier Vatter, F.A., Schapiro, D., Chang, H., Borowsky, A.D., Lee, J.K., Parvin, B., Stampfer, M.R., LaBarge, M.A., Bodenmiller, B., and Lorens, J.B. (2018). High-dimensional phenotyping identifies age-emergent cells in human mammary epithelia. Cell Rep. *23*, 1205–1219.

Polyak, K., and Kalluri, R. (2010). The role of the microenvironment in mammary gland development and cancer. Cold Spring Harb. Perspect. Biol. *2*, a003244.

Proia, T.A., Keller, P.J., Gupta, P.B., Klebba, I., Jones, A.D., Sedic, M., Gilmore, H., Tung, N., Naber, S.P., Schnitt, S., et al. (2011). Genetic predisposition directs breast cancer phenotype by dictating progenitor cell fate. Cell Stem Cell *8*, 149–163.

Raafat, A., Strizzi, L., Lashin, K., Ginsburg, E., McCurdy, D., Salomon, D., Smith, G.H., Medina, D., and Callahan, R. (2012). Effects of age and parity on mammary gland lesions and progenitor cells in the FVB/N-RC mice. PLoS ONE *7*, e43624.

Robinson, M.D., McCarthy, D.J., and Smyth, G.K. (2010). edgeR: a Bioconductor package for differential expression analysis of digital gene expression data. Bioinformatics *26*, 139–140.

Rosenbluth, J.M., Schackmann, R.C.J., Gray, G.K., Selfors, L.M., Li, C.M.-C., Boedicker, M., Kuiken, H.J., Richardson, A., Brock, J., Garber, J., et al. (2020). Organoid cultures from normal and cancer-prone human breast tissues preserve complex epithelial lineages. Nat. Commun. *11*, 1711–1714.

Russell, T.D., Schaack, J., Orlicky, D.J., Palmer, C., Chang, B.H.-J., Chan, L., and McManaman, J.L. (2011). Adipophilin regulates maturation of cytoplasmic lipid droplets and alveolae in differentiating mammary glands. J. Cell Sci. *124*, 3247–3253.

Russo, J., Balogh, G.A., and Russo, I.H. (2008). Full-term pregnancy induces a specific genomic signature in the human breast. Cancer Epidemiol. Biomarkers Prev. *17*, 51–66.

Sampath, D., Winneker, R.C., and Zhang, Z. (2002). The angiogenic factor Cyr61 is induced by the progestin R5020 and is necessary for mammary adenocarcinoma cell growth. Endocrine *18*, 147–159.

Sanada, F., Taniyama, Y., Muratsu, J., Otsu, R., Shimizu, H., Rakugi, H., and Morishita, R. (2018). Source of chronic inflammation in aging. Front. Cardiovasc. Med. *5*, 12.

Schindelin, J., Arganda-Carreras, I., Frise, E., Kaynig, V., Longair, M., Pietzsch, T., Preibisch, S., Rueden, C., Saalfeld, S., Schmid, B., et al. (2012). Fiji: an open-source platform for biological-image analysis. Nat. Methods *9*, 676–682.

Schultz, M.B., and Sinclair, D.A. (2016). When stem cells grow old: phenotypes and mechanisms of stem cell aging. Development *143*, 3–14.

Shackleton, M., Vaillant, F., Simpson, K.J., Stingl, J., Smyth, G.K., Asselin-Labat, M.-L., Wu, L., Lindeman, G.J., and Visvader, J.E. (2006). Generation of a functional mammary gland from a single stem cell. Nature *439*, 84–88.

Sigurdsson, V., Ingthorsson, S., Hilmarsdottir, B., Gustafsdottir, S.M., Franzdottir, S.R., Arason, A.J., Steingrimsson, E., Magnusson, M.K., and Gudjonsson, T. (2013). Expression and functional role of sprouty-2 in breast morphogenesis. PLoS ONE *8*, e60798.

Singletary, K.W., and McNary, M.Q. (1992). Effect of moderate ethanol consumption on mammary gland structural development and DNA synthesis in the female rat. Alcohol *9*, 95–101.

Sirka, O.K., Shamir, E.R., and Ewald, A.J. (2018). Myoepithelial cells are a dynamic barrier to epithelial dissemination. J. Cell Biol. *217*, 3368–3381.

Sopel, M. (2010). The myoepithelial cell: its role in normal mammary glands and breast cancer. Folia Morphol. (Warsz) *69*, 1–14.

Standaert, L., Adriaens, C., Radaelli, E., Van Keymeulen, A., Blanpain, C., Hirose, T., Nakagawa, S., and Marine, J.-C. (2014). The long noncoding RNA Neat1 is required for mammary gland development and lactation. RNA *20*, 1844–1849.

Stingl, J., Eirew, P., Ricketson, I., Shackleton, M., Vaillant, F., Choi, D., Li, H.I., and Eaves, C.J. (2006). Purification and unique properties of mammary epithelial stem cells. Nature *439*, 993–997.

Stuart, T., Butler, A., Hoffman, P., Hafemeister, C., Papalexi, E., Mauck, W.M., 3rd, Hao, Y., Stoeckius, M., Smibert, P., and Satija, R. (2019). Comprehensive integration of single-cell data. Cell *177*, 1888–1902.e21.

Subramanian, A., Tamayo, P., Mootha, V.K., Mukherjee, S., Ebert, B.L., Gillette, M.A., Paulovich, A., Pomeroy, S.L., Golub, T.R., Lander, E.S., and Mesirov, J.P. (2005). Gene set enrichment analysis: a knowledge-based approach

for interpreting genome-wide expression profiles. Proc. Natl. Acad. Sci. USA *102*, 15545–15550.

Suciu-Foca, N., and Cortesini, R. (2007). Central role of ILT3 in the T suppressor cell cascade. Cell. Immunol. *248*, 59–67.

Tabula Muris Consortium (2020). A single-cell transcriptomic atlas characterizes ageing tissues in the mouse. Nature *583*, 590–595. https://www.ncbi.nlm.nih.gov/entrez/query.fcgi?cmd=Retrieve&db=PubMed&list_uids=32669714&dopt=Abstract.

Tabula Muris Consortium; Overall coordination; Logistical coordination; Organ collection and processing; Library preparation and sequencing; Computational data analysis; Cell type annotation; Writing group; Supplemental text writing group; Principal investigators (2018). Single-cell transcriptomics of 20 mouse organs creates a Tabula Muris. Nature *562*, 367–372.

Theocharis, A.D., Skandalis, S.S., Gialeli, C., and Karamanos, N.K. (2016). Extracellular matrix structure. Adv. Drug Deliv. Rev. *97*, 4–27.

Tsai, M.-S., Bogart, D.F., Li, P., Mehmi, I., and Lupu, R. (2002). Expression and regulation of Cyr61 in human breast cancer cell lines. Oncogene *21*, 964–973.

Tsubura, A., Shikata, N., Inui, T., Morii, S., Hatano, T., Oikawa, T., and Matsuzawa, A. (1988). Immunohistochemical localization of myoepithelial cells and basement membrane in normal, benign and malignant human breast lesions. Virchows Arch. A Pathol. Anat. Histopathol. *413*, 133–139.

Tynan, S., Pacia, E., Haynes-Johnson, D., Lawrence, D., D'Andrea, M.R., Guo, J.-Z., Lundeen, S., and Allan, G. (2005). The putative tumor suppressor deleted in malignant brain tumors 1 is an estrogen-regulated gene in rodent and primate endometrial epithelium. Endocrinology *146*, 1066–1073.

Unsworth, A., Anderson, R., and Britt, K. (2014). Stromal fibroblasts and the immune microenvironment: partners in mammary gland biology and pathology? J. Mammary Gland Biol. Neoplasia *19*, 169–182.

Van den Berge, K., Perraudeau, F., Soneson, C., Love, M.I., Risso, D., Vert, J.-P., Robinson, M.D., Dudoit, S., and Clement, L. (2018). Observation weights unlock bulk RNA-seq tools for zero inflation and single-cell applications. Genome Biol. *19*, 24.

Van Keymeulen, A., Fioramonti, M., Centonze, A., Bouvencourt, G., Achouri, Y., and Blanpain, C. (2017). Lineage-restricted mammary stem cells sustain the development, homeostasis, and regeneration of the estrogen receptor positive lineage. Cell Rep. *20*, 1525–1532.

Vaughan, A.E., Brumwell, A.N., Xi, Y., Gotts, J.E., Brownfield, D.G., Treutlein, B., Tan, K., Tan, V., Liu, F.C., Looney, M.R., et al. (2015). Lineage-negative progenitors mobilize to regenerate lung epithelium after major injury. Nature *517*, 621–625.

Wahl, G.M., and Spike, B.T. (2017). Cell state plasticity, stem cells, EMT, and the generation of intra-tumoral heterogeneity. NPJ Breast Cancer *3*, 14.

Wahle, E., and Winkler, G.S. (2013). RNA decay machines: deadenylation by the Ccr4-not and Pan2-Pan3 complexes. Biochim. Biophys. Acta *1829*, 561–570.

Wang, C., Christin, J.R., Oktay, M.H., and Guo, W. (2017). Lineage-biased stem cells maintain estrogen-receptor-positive and -negative mouse mammary luminal lineages. Cell Rep. *18*, 2825–2835.

Wang, Y., Chaffee, T.S., LaRue, R.S., Huggins, D.N., Witschen, P.M., Ibrahim, A.M., Nelson, A.C., Machado, H.L., and Schwertfeger, K.L. (2020). Tissue-resident macrophages promote extracellular matrix homeostasis in the mammary gland stroma of nulliparous mice. eLife *9*, 1399.

Warburton, M.J., Ormerod, E.J., Monaghan, P., Ferns, S., and Rudland, P.S. (1981). Characterization of a myoepithelial cell line derived from a neonatal rat mammary gland. J. Cell Biol. *91*, 827–836.

Warburton, M.J., Mitchell, D., Ormerod, E.J., and Rudland, P. (1982). Distribution of myoepithelial cells and basement membrane proteins in the resting, pregnant, lactating, and involuting rat mammary gland. J. Histochem. Cytochem. *30*, 667–676.

Wolock, S.L., Lopez, R., and Klein, A.M. (2019). Scrublet: computational identification of cell doublets in single-cell transcriptomic data. Cell Syst. *8*, 281–291.e9.

Wuidart, A., Sifrim, A., Fioramonti, M., Matsumura, S., Brisebarre, A., Brown, D., Centonze, A., Dannau, A., Dubois, C., Van Keymeulen, A., et al. (2018). Early lineage segregation of multipotent embryonic mammary gland progenitors. Nat. Cell Biol. *20*, 666–676.

Yamazaki, T., Akiba, H., Iwai, H., Matsuda, H., Aoki, M., Tanno, Y., Shin, T., Tsuchiya, H., Pardoll, D.M., Okumura, K., et al. (2002). Expression of programmed death 1 ligands by murine T cells and APC. J. Immunol. *169*, 5538–5545.

Yue, Y., Liu, J., Cui, X., Cao, J., Luo, G., Zhang, Z., Cheng, T., Gao, M., Shu, X., Ma, H., et al. (2018). VIRMA mediates preferential m^6A mRNA methylation in 3'UTR and near stop codon and associates with alternative polyadenylation. Cell Discov. *4*, 10–17.

Zhang, X., Qiao, G., and Lu, P. (2014). Modulation of fibroblast growth factor signaling is essential for mammary epithelial morphogenesis. PLoS ONE *9*, e92735.

Zhang, S., Yang, X., Wang, L., and Zhang, C. (2018a). Interplay between inflammatory tumor microenvironment and cancer stem cells. Oncol. Lett. *16*, 679–686.

Zhang, Y., Zhang, S., Guan, W., Chen, F., Cheng, L., Lv, Y., and Chen, J. (2018b). GLUT1 and lactose synthetase are critical genes for lactose synthesis in lactating sows. Nutr. Metab. (Lond.) *15*, 40.

Zheng, G., Dahl, J.A., Niu, Y., Fedorcsak, P., Huang, C.-M., Li, C.J., Vågbø, C.B., Shi, Y., Wang, W.-L., Song, S.-H., et al. (2013). ALKBH5 is a mammalian RNA demethylase that impacts RNA metabolism and mouse fertility. Mol. Cell *49*, 18–29.

Zhu, W., and Nelson, C.M. (2013). PI3K regulates branch initiation and extension of cultured mammary epithelia via Akt and Rac1 respectively. Dev. Biol. *379*, 235–245.

STAR★METHODS

KEY RESOURCES TABLE

REAGENT or RESOURCE	SOURCE	IDENTIFIER
Antibodies		
ACTA2/SMA	Dako	Cat#M0851; RRID: AB_2223500
ARG1	Cell Signaling Technology	Cat#66297; RRID: AB_2799705
CD163	Abcam	Cat#ab182422; RRID: AB_2753196
CD8α	Cell Signaling Technology	Cat#98941; RRID: AB_2756376
KRT15	BioLegend	Cat#833904; RRID: AB_2616894
KRT14	BioLegend	Cat#905301; RRID: AB_2565048
KRT14	Abcam	Cat#ab206100; RRID: AB_2811031
KRT8	DSHB	Cat#Troma-I; RRID: AB_531826
KRT8	Abcam	Cat#ab192467; RRID: AB_2864346
PDGFRα	Cell Signaling Technology	Cat#3174; RRID: AB_2162345
PR	Cell Signaling Technology	Cat#8757; RRID: AB_2797144
PR	Dako	Cat#A0098; RRID: AB_2315192
ER	Santa Cruz	Cat#sc-542; RRID: AB_631470
ER	BioRad	Cat#MCA1799T; RRID: AB_2102069
LTF	Bioss	Cat#bs-5810; RRID: AB_11078952
MFGE8	Thermo	Cat#MA5-23913; RRID: AB_2609198
MFGE8	R&D Systems	Cat#MAB2805; RRID: AB_2297564
CD45-FITC	BioLegend	Cat#103108; RRID: AB_312973
CD31-FITC	BioLegend	Cat#102405; RRID: AB_312900
TER119-FITC	BioLegend	Cat#116205; RRID: AB_313706
EPCAM-BV605	BioLegend	Cat#118227; RRID: AB_2563984
CD133-APC	BioLegend	Cat#141207; RRID: AB_10898121
CD14-PE	BioLegend	Cat#150106; RRID: AB_2728189

(*Continued on next page*)

Continued

REAGENT or RESOURCE	SOURCE	IDENTIFIER
Critical Commercial Assays		
Chromium Single Cell 3′ Assay	10x Genomics	N/A
Deposited Data		
scRNA-seq data	This study	GSE150580
Experimental Models: Organisms/Strains		
Mouse: mixed FVB, 129, C57BL/6J background	In house	N/A
Software and Algorithms		
R (3.5.1)	R project	RRID: SCR_001905
Python (2.7, python 3.7)	Python Software Foundation	RRID:SCR_008394
Cell Ranger v2	10x Genomics	RRID:SCR_017344
Seurat v3	Butler et al., 2018; Stuart et al., 2019	RRID:SCR_016341
Scrublet	Wolock et al., 2019	RRID:SCR_018098
DoubletFinder	McGinnis et al., 2019	RRID:SCR_018771
STREAM	Chen et al., 2019	N/A
zinger-edgeR	Van den Berge et al., 2018	N/A
NCBI Homologene	https://www.ncbi.nlm.nih.gov/homologene	RRID:SCR_002924
MSigDB v7.0	Liberzon et al., 2015; Subramanian et al., 2005	RRID:SCR_016863
Fiji	Schindelin et al., 2012	RRID:SCR_002285
FlowJo v10	BD Biosciences	RRID:SCR_008520
Prism v8	Graphpad software	RRID:SCR_002798

RESOURCE AVAILABILITY

Lead Contact

Further information and requests for resources and reagents should be directed to the Lead Contact, Joan Brugge (joan_brugge@hms.harvard.edu).

Materials Availability

This study did not generate new unique reagents.

Data and Code Availability

The accession number for the scRNA-seq data reported in this paper is GEO: GSE150580.

The R scripts used for scRNA-seq analysis are included in the supplemental information (Data S1).

EXPERIMENTAL MODEL AND SUBJECT DETAILS

For single-cell RNA sequencing, young (3-4 months old) and aged (13-14 months old) virgin/nulliparous female mice of a mixed FVB, 129, C57BL/6J background were used in this study. For immunostaining, additional young (3-4 months old) and aged (16-months old) mice were used. For FACS analysis, an independent cohort of young (3-4 months old) and aged (12-14 months old) mice were used. All mice were bred and maintained in house under regular conditions, with 12hr/12hr light/dark cycle, and food (LabDiet 5053) and water *ad libitum*. Daily vaginal cytology was performed to determine the stage of the estrous cycle, and only actively cycling mice in diestrus were analyzed to avoid confounding effects from hormonal changes. Aged mammary glands were confirmed to have normal histology by a rodent pathologist. All animal work was performed in accordance to protocols approved by the Institutional Animal Care and Use Committee at Harvard University.

METHOD DETAILS

Tissue dissociation for single-cell RNA-seq

Abdominal #4 mammary glands from young (n = 3) and aged (n = 4) mice, with lymph nodes removed, were finely minced and then incubated in a digestion solution containing DMEM/F12 (GIBCO 11330), 10% heat-inactivated fetal bovine serum, 2 mg/ml collagenase XI (Sigma C9407), and 0.1 mg/ml hyaluronidase (Sigma H3506) for 1.5 hours at 37°C with constant shaking at 150 rpm. The

dissociated cells were then subjected to red blood cell lysis (Biolegend 420301), a 5-minute treatment with 1 U/ml dispase (Stem Cell Technologies 07913) and 0.1 mg/ml DNase (Stem Cell Technologies 07470), a 5-minute treatment with TrypLE (GIBCO 12605010), and filtered through a 40 μm cell strainer. Cells were resuspended in PBS containing 0.04% BSA, counted manually under the microscope, and adjusted for loading 7,000 viable cells for single-cell RNA-sequencing.

Single-cell RNA library preparation and sequencing

Single cell capturing and cDNA library generation were performed using the 10X Chromium 3′ library construction kit v2 following the manufacturer's instruction. The libraries were then pooled and sequenced by Illumina HiSeq X Ten.

Single-cell RNA-seq data processing

Paired-end reads from Illumina HiSeq were processed and mapped to the mm10 mouse genome using Cell Ranger v2.0. Sequencing saturation as determined by Cell Ranger showed fairly even saturation across samples (young mice: 82.3%, 79.9%, 70.6%; aged mice: 91.3%, 78.6%, 73.3%, 85.1%). We applied stringent filters to eliminate cells with (1) UMI counts < 1,000 or > 60,000, (2) gene counts < 500 or > 2,500, and (3) mitochondrial gene ratio > 10%. This pre-filtering resulted in the detection of 27,998 genes in 13,684 cells, with approximately 1,200-2,800 cells from each sample. A median of 1,649 genes and 5,538 transcripts were captured per cell. The filtered data were then analyzed using Seurat v3 (Butler et al., 2018; Stuart et al., 2019). Cell doublets were estimated using Scrublet (Wolock et al., 2019) and DoubletFinder (McGinnis et al., 2019). Lineage trajectory analysis was performed on the luminal epithelial cells of each of the three young samples, as well as on the three samples combined, using STREAM (Chen et al., 2019). Cell cycle estimation was performed in Seurat using the default cell-cycle gene signatures.

Cell type-specific gene signatures

Cell type-specific gene expression signatures were identified as genes that exhibited expression levels > 1.25-fold greater (with adjusted *p-value*s < 0.05) than each of the other cell types in multiple pairwise differential expression analyses using Seurat's FindMarkers function (Wilcoxon rank sum test). Similarly, gene expression signature for HS-AV luminal cells was generated by identifying genes with expression levels at least 1.25-fold higher or lower (with adjusted *p-value*s < 0.05) when compared to luminal HS cells and to luminal AV cells separately. The list of markers distinguishing M_a and M_b macrophages was generated by identifying genes with expression levels differing by at least 2-fold (with adjusted *p-value*s < 0.05). Heatmaps for visualizing marker gene expression were median-centered and down-sampled to 100 cells per cell type.

Differential gene expression across age groups

Differential gene expression analysis was performed to compare young and aged samples within each cell type by using a combination of Seurat's FindMarkers function (Wilcoxon rank sum test) and zingeR-edgeR zero-inflated negative binomial analysis (Robinson et al., 2010; Van den Berge et al., 2018) for increased stringency. Differentially expressed genes were identified using three criteria: (i) Grouped analysis criteria: an expression difference of at least 1.5-fold and an adjusted *p-value* of < 0.05 in a grouped comparison of young mice (n = 3) versus aged mice (n = 4); (ii) Per-sample analysis criteria: an expression difference of at least 1.25-fold in all of the 12 possible combinations of young-versus-aged per-sample pairwise comparison; (iii) Cross-method criteria: only genes satisfying criteria (i) and (ii) in both FindMarkers and zingeR-edgeR analyses were included in the final list of differentially expressed genes. Only genes with expression detected in at least 10% of cells in either the young or aged population were analyzed. Heatmaps for visualizing the differentially expressed genes were median-centered and down-sampled to 200 cells per age group, or, in the case of M_b macrophages where fewer cells were detected, down-sampled to 20 cells per group.

Pathway analysis

Pathway analysis was performed on differentially expressed genes using MSigDB v7.0 (Liberzon et al., 2015; Subramanian et al., 2005) curated canonical pathway database. To ensure proper gene name mapping, all mouse gene names were converted to their human homologs using NCBI Homologene prior to analysis in MSigDB. Gene sets with FDR < 0.05 were deemed statistically significant. Up to top 30 enriched gene sets were shown.

Histology and immunostaining

Mouse mammary tissues were fixed in 10% neutral buffered formalin overnight. Paraffin embedding and sectioning were performed by the Rodent Histopathology Core at Harvard Medical School. Immunostaining was performed on n = 6 animals per age group. For immunofluorescence staining, the following antibodies were used: ACTA2/SMA (Dako M0851, 1:100), KRT15 (BioLegend 833904, 1:50), KRT14 (BioLegend 905301, 1:250; Abcam ab206100, 1:150), KRT8 (DSHB Troma-I, 1:100; Abcam ab192467, 1:100), ARG1 (CST 66297, 1:100), PR (CST 8757, 1:25; Dako A0098, 1:20), ER (Santa Cruz sc-542, 1:20; BioRad MCA1799T, 1:25), LTF (Bioss bs-5810, 1:50), MFGE8 (Thermo MA5-23913, 1:50; R&D Systems MAB2805, 1:50). LTF and MFGE8 antibodies were validated by staining lactating mammary glands. For immunohistochemistry staining, the following antibodies were used: PDGFRα (CST 3174, 1:200), CD163 (Abcam ab182422, 1:500), CD8 (CST 98941, 1:50). Images were analyzed in a blinded fashion using Fiji (Schindelin et al., 2012).

FACS analysis

For FACS analysis of mammary tissues, freshly dissected #2, 3, and 4 mammary glands from young (n = 8) and aged (n = 6) mice were digested as described above. Cells were then incubated with CD16/32 antibody (eBioscience 16-0161) to block Fc receptor and with rat serum (eBioscience 24-5555) to block non-specific binding, and then stained for 30 minutes with the following antibodies/reagents: CD45-FITC (BioLegend 103108, 1:200), CD31-FITC (BioLegend 102405, 1:50), TER119-FITC (BioLegend 116205, 1:100), EPCAM-BV605 (BioLegend 118227, 1:100), CD133-APC (BioLegend 141207, 1:50), CD14-PE (BioLegend 150106, 1:50), and DAPI (1 μg/mL). Cell sorting and analysis was carried out on a BD FACSAria II cell sorter and FlowJo using the following gating strategy: SSC-H/SSC-W singlets, FSC-H/FSC-W singlets, DAPI- live cells, CD45-/CD31-/TER119- Lin- cells, EPCAM-high luminal epithelial cells. The gated luminal population was then analyzed for expression of CD133 and CD14. For FACS analysis of organoid cultures, primary mammary organoids were generated from 3-4 month-old FVB female mice using the aforementioned tissue digestion protocol and propagated for six weeks in defined basement-membrane extract and culture media, as described previously (Rosenbluth et al., 2020). Organoids were digested into single cells using TrypLE, blocked with CD16/32 antibody and rat serum, and then stained with a similar antibody panel as described above. EPCAM-BV605 was omitted in order to allow for subsequent microscopy examination of cells double positive for CD133-APC and CD14-PE without introducing spectral overlapping from EPCAM-BV605.

QUANTIFICATION AND STATISTICAL ANALYSIS

For cell type composition comparison between young and aged mammary glands in scRNA-seq data, statistically significant difference was determined by Fisher's exact test and Chi-square test. We noted that because all proportions sum to one in scRNA-seq data, an increase in the proportion of one cell population will necessarily lead to a decrease in the proportions of other cell populations. However, alternative multivariate tests for compositional data analysis (e.g., Dirichlet-multinomial regression test) were not applied as they have increased type 2 error over type 1 error. For quantification by immunofluorescence staining, immunohistochemistry staining, and FACS analysis, statistically significant difference was determined by Student's t test. Statistical analysis was performed by using the GraphPad Prism software, and *p-value*s < 0.05 (two-tailed) were deemed statistically significant. Bar graphs represent mean ± SEM.

Cell Reports, Volume *33*

Supplemental Information

Aging-Associated Alterations in Mammary Epithelia and Stroma Revealed by Single-Cell RNA Sequencing

Carman Man-Chung Li, Hana Shapiro, Christina Tsiobikas, Laura M. Selfors, Huidong Chen, Jennifer Rosenbluth, Kaitlin Moore, Kushali P. Gupta, G. Kenneth Gray, Yaara Oren, Michael J. Steinbaugh, Jennifer L. Guerriero, Luca Pinello, Aviv Regev, and Joan S. Brugge

Figure S1

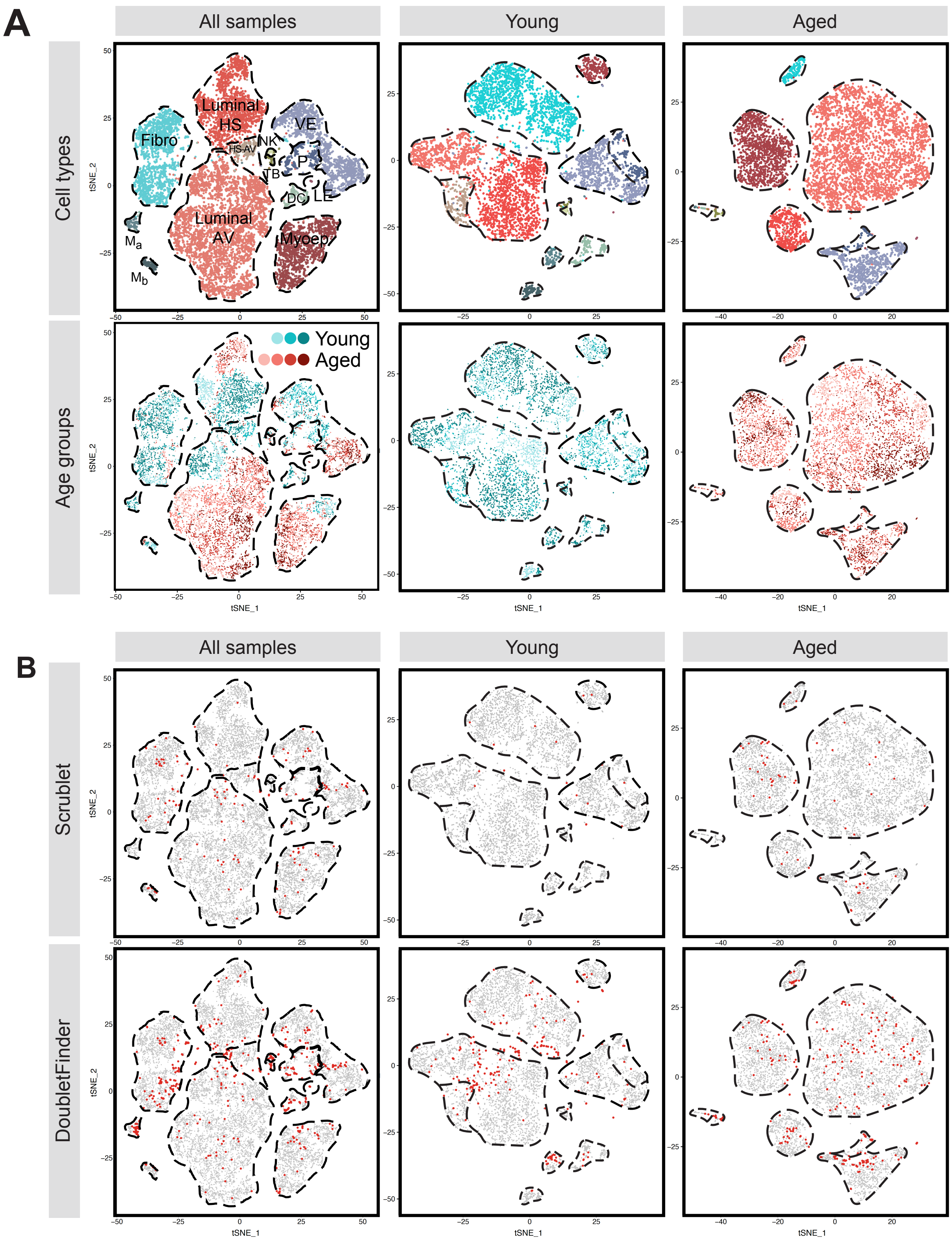
A
All samples
Young
Aged
Cell types
Age groups
Luminal HS
Fibro
VE
NK
HS-AV
P
TB
DC
LE
Luminal AV
Myoep
Ma
Mb
Young
Aged
tSNE_1
tSNE_2
B
All samples
Young
Aged
Scrublet
DoubletFinder
tSNE_1
tSNE_2

Figure S1 (related to Figure 1). t-SNE Plots Showing Distribution of Samples and Identification of Potential Doublets

(A) t-SNE plots colored by age groups and by biological replicates.

(B) t-SNE plot showing predicted doublets in scRNA-seq data using Scrublet and DoubletFinder. If a cluster was overwhelmingly enriched with predicted doublets, it would likely represent an artifact doublet population generated by chance inclusion of two or more cells in a single droplet during the cell capturing step on the 10X Chromium platform. No such cluster is detected in our dataset.

Figure S2

A

Chi-square	Young (A)	Young (B)	Young (C)	Aged (A)	Aged (B)	Aged (C)	Aged (D)
**Young (A)**							
**Young (B)**	348						
**Young (C)**	555	729					
**Aged (A)**	926	990	1499				
**Aged (B)**	1718	1629	2669	141			
**Aged (C)**	1281	1179	1834	152	155		
**Aged (D)**	1227	1210	2039	135	32	105	

All p-values < 0.0001; df = 6

B

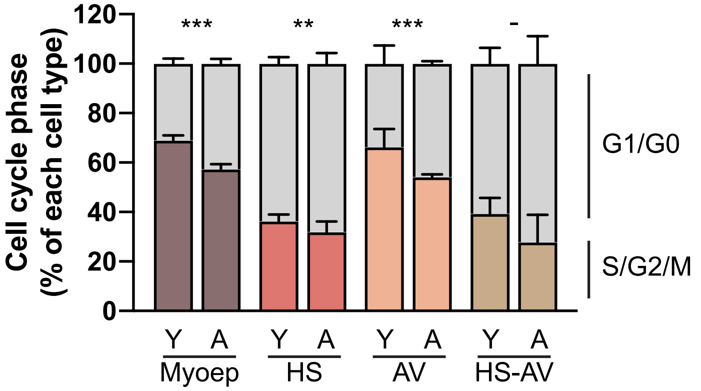

Young

	Myoep	HS	AV	HS-AV
**Myoep**				
**HS**	****			
**AV**	-	****		
**HS-AV**	****	-	****	

Aged

	Myoep	HS	AV	HS-AV
**Myoep**				
**HS**	****			
**AV**	*	****		
**HS-AV**	**	-	*	

Figure S2 (related to Figure 2A). Cell Type Proportions and Cell Cycle Fractions within and across Age Groups

(A) Cell type proportions are consistent within biological replicates and distinct across age groups. Table summarizes Chi-square test results for all per-sample pairwise comparisons of cell type proportions. The lower Chi-square values within each age group reflect consistency among biological replicates, whereas the higher Chi-square values across age groups reflect age-dependent differences.

(B) Bar graph of the fraction of cells in S/G2/M phases (colored bars) or G1/G0 phase (gray bars) within each epithelial cell type. Cell cycle estimation was performed using the default CellCycleScoring function in Seurat, with cells in G2/M/S considered cycling and G1/G0 non-cycling. All values represent mean + SEM. Statistically significant differences between young (Y) and aged (A) groups within a cell type are denoted above the bars (Fisher's exact test). Statistically significant differences between cell types within young mice or within aged mice are denoted in the respective tables (Fisher's exact test). All p-values are denoted by * (< 0.05), ** (<0.01), *** (<0.001), **** (<0.0001), or – (not significant). Within each cell type, n = 3 young mice and n = 4 aged mice were analyzed, except for HS-AV (n = 3+3), where such cells were not detected in one of the four aged mice. Abbreviations: Myoep, myoepithelial cells; HS, hormone-sensing luminal cells; AV, alveolar luminal cells.

Figure S3

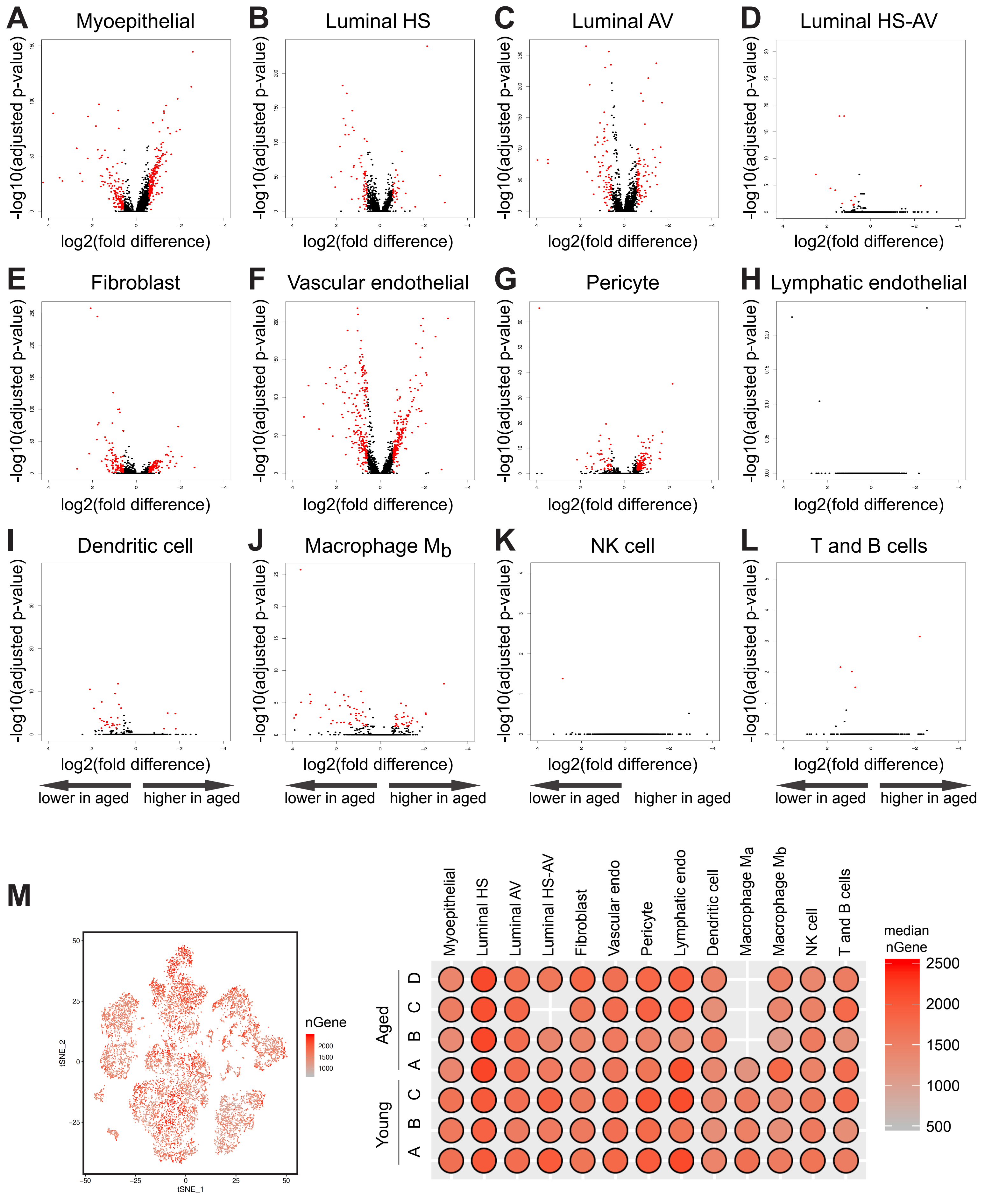

A
Myoepithelial
B
Luminal HS
C
Luminal AV
D
Luminal HS-AV
E
Fibroblast
F
Vascular endothelial
G
Pericyte
H
Lymphatic endothelial
I
Dendritic cell
J
Macrophage Mb
K
NK cell
L
T and B cells
-log10(adjusted p-value)
log2(fold difference)
lower in aged
higher in aged
M
tSNE_1
tSNE_2
nGene
Myoepithelial
Luminal HS
Luminal AV
Luminal HS-AV
Fibroblast
Vascular endo
Pericyte
Lymphatic endo
Dendritic cell
Macrophage Ma
Macrophage Mb
NK cell
T and B cells
Aged
Young
median nGene
2500
2000
1500
1000
500

Figure S3 (related to Figures 3, 4, 6, and 7). Prevalent Alterations in Gene Expression in Aged Mammary Glands

(A-L) Volcano plots show expression levels of all detected genes in aged mammary glands (n = 4) compared to young mammary glands (n = 3) within the specified cell types. Genes with fold-difference above 1.5 and with adjusted *p*-value < 0.05 are highlighted in red. Differential expression analysis was not possible for M_a macrophages due to their extremely low abundance in aged mice.

(M) Number of genes (nGene) captured by scRNA-seq per cell (left) and median nGene within each cell type per sample (right). HS-AV cells and M_a macrophages were not detected in one or more aged mice.

Figure S4

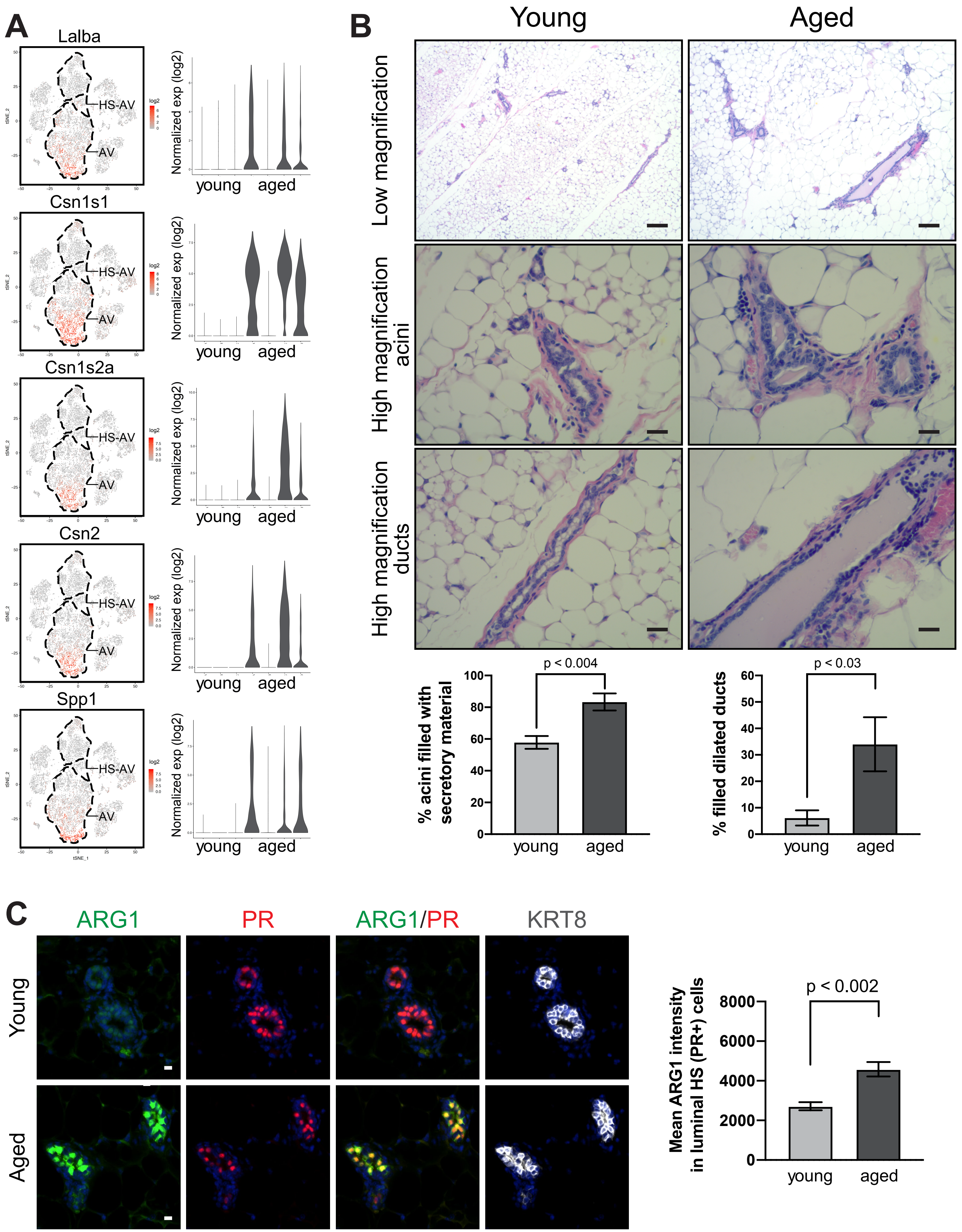
A
Lalba
Csn1s1
Csn1s2a
Csn2
Spp1
HS-AV
AV
Normalized exp (log2)
young aged
B
Young
Aged
Low magnification
High magnification acini
High magnification ducts
% acini filled with secretory material
p < 0.004
% filled dilated ducts
p < 0.03
young
aged
C
ARG1
PR
ARG1/PR
KRT8
Young
Aged
Mean ARG1 intensity in luminal HS (PR+) cells
p < 0.002
young
aged

Figure S4 (related to Figure 4). Alveolar and Hormone-Sensing Luminal Cell Changes with Aging
(A) t-SNE plots (left) showing the expression of milk biosynthesis-related genes in a subset of aged AV cells. Violin plots (right) showing that the expression of these genes is upregulated in AV cells in three out of four aged mammary glands compared to young glands.
(B) Representative H&E images and quantification of young and aged mammary acini and ducts. Aged mammary glands show increased secretory material in acini and dilated ducts. H&E at low magnification, scale bar = 100 μm; at high magnification, scale bar = 20 μm. For quantification of acini filled with secretory material, at least 20 acini per gland with diameter < 50 μm were analyzed. For quantification of ducts filled and dilated, at least 10 ducts per gland with cross-section length > 250 μm were analyzed. Ducts were considered dilated if the width:length ratio was > 1:4, i.e. 0.25. Analysis was performed on n = 6 animals per age group using Student's t test.
(C) Representative immunofluorescence staining pattern and quantification of ARG1 expression in luminal HS cells (stained as PR+) in young and aged mammary glands. KRT8 marks luminal cells. Scale bar = 10 μm. For quantification, at least 800 HS cells per mouse were analyzed. Analysis was performed on n = 6 animals per age group using Student's t test.

Figure S5

A

This Study		
Strain	Mixed FVB, 129, C57BL/6J mice	
Parity	Nulliparous	
Estrous staging	Diestrus	
Age	3-4	13-14 months
	n=3	n=4
Cell type	Young	Aged
HS	59%	12%
AV	29%	88%
HS-AV	12%	0.3%
Total luminal cells	100%	100%

Bach et al., 2017	
C57BL/6N mice	
Nulliparous	
Not estrous staged	
2 months	
	n=2
Cell type (orig. name)	Young
HS (HSd)	66%
AV (Lp)	21%
HS-AV (HSp)	13%
Total luminal cells	100%

Pal et al., 2017		
FVB/NJ mice		
Nulliparous		
	Estrus	Diestrus
2.5 months		
	n=2	n=2
Cell type (orig. name)	Young estrus	Young diestrus
HS (Mature luminal)	52%	53%
AV (Luminal progenitor)	33%	42%
HS-AV (Luminal intermediate)	10%	4%
Other 1 (Rare cluster)	1%	1%
Other 2 (Rare cluster)	3%	1%
Total luminal cells	100%	100%

Kanaya et al., 2019				
BALB/cj				
Nulliparous				
Ovariectomy at 9 weeks of age, one-week treatment before analysis				
5 months				
	n=2+2+2	n=2	n=2	n=2
Cell type (orig. name)	All	Vehicle	E2	E2+PBDE
HS (Mature luminal)	49%	28%	51%	53%
AV (Luminal progenitor)	14%	17%	17%	15%
HS-AV (Luminal intermediate)	36%	55%	33%	33%
Total luminal cells	100%	100%	100%	100%

B

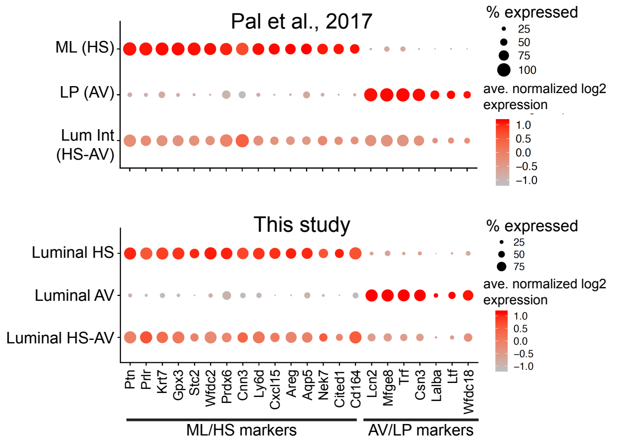

C

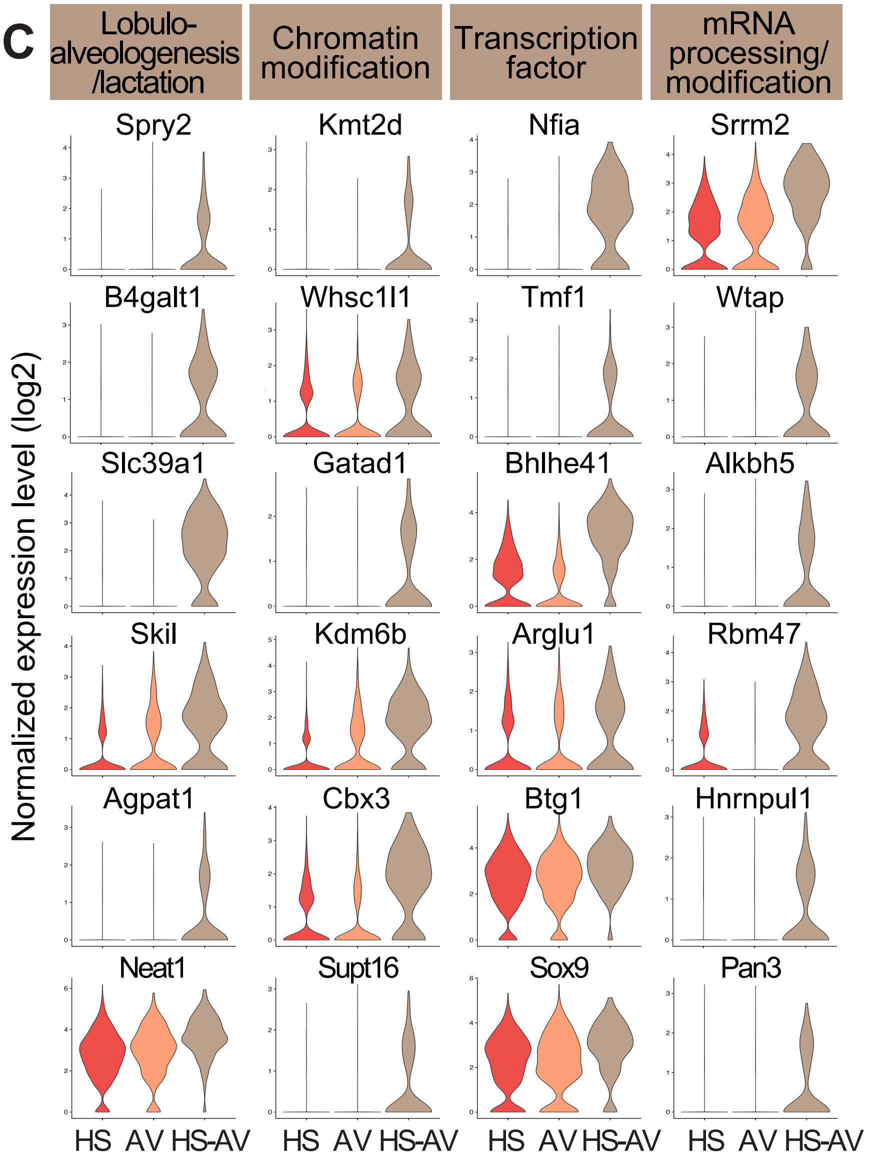

D

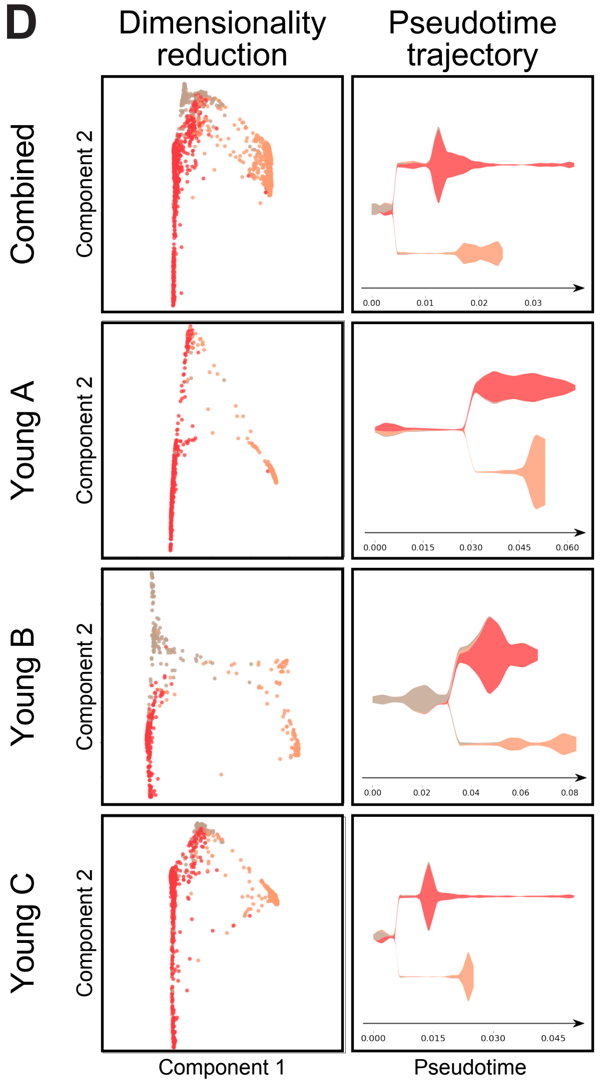

E

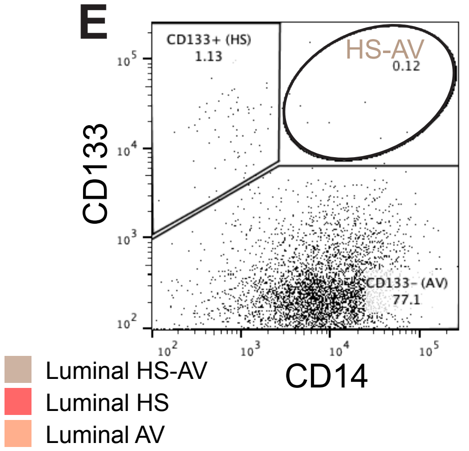

F

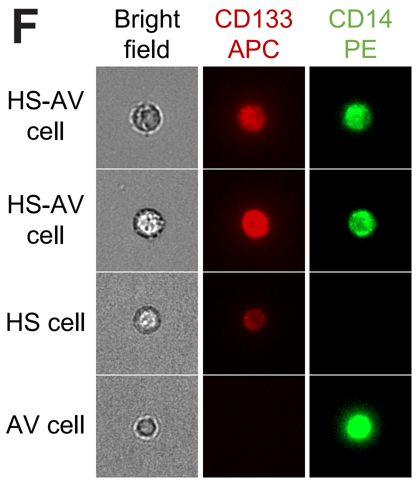

Figure S5 (related to Figure 5). HS-AV Gene Markers and Lineage Trajectory Analysis

(A) Proportions of luminal HS, AV, and HS-AV cells in young mice analyzed in this study compared to equivalent cell populations in other studies of young mice (adapted from Figure 6a in Pal et al., 2017 and Table 1 in Bach et al., 2017) and ovariectomized mice (adapted from Table 2 and Supplementary Figure 10a in Kanaya et al, 2019; E2, 17β-estradiol; PBDE, polybrominated diphenyl ethers).

(B) Expression of select HS and AV markers in Luminal Intermediate cells (Pal et al., 2017) and HS-AV cells (this study). Markers were partially adapted from Figure S4b of Pal et al., 2017.

(C) Violin plots of select genes identified in Figure 5B with expression enriched in HS-AV cells relative to HS cells and AV cells.

(D) Results from lineage trajectory analysis of luminal cells in young mice (n = 3), analyzed either in aggregate or as individual samples. In each case, HS-AV cells are localized to the bifurcation between the HS lineage and AV lineage.

(E) HS-AV cells co-expressing HS marker CD133 and AV marker CD14 are detected by FACS in organoid cultures of primary mammary cells.

(F) Example of HS-AV cells isolated by FACS and examined under the microscope to confirm that they are single cells with co-staining for CD133 and CD14, not doublets composed of CD133+ cell and CD14+ cell. CD133+ HS cells and CD14+ AV cells serve as controls.

Figure S6

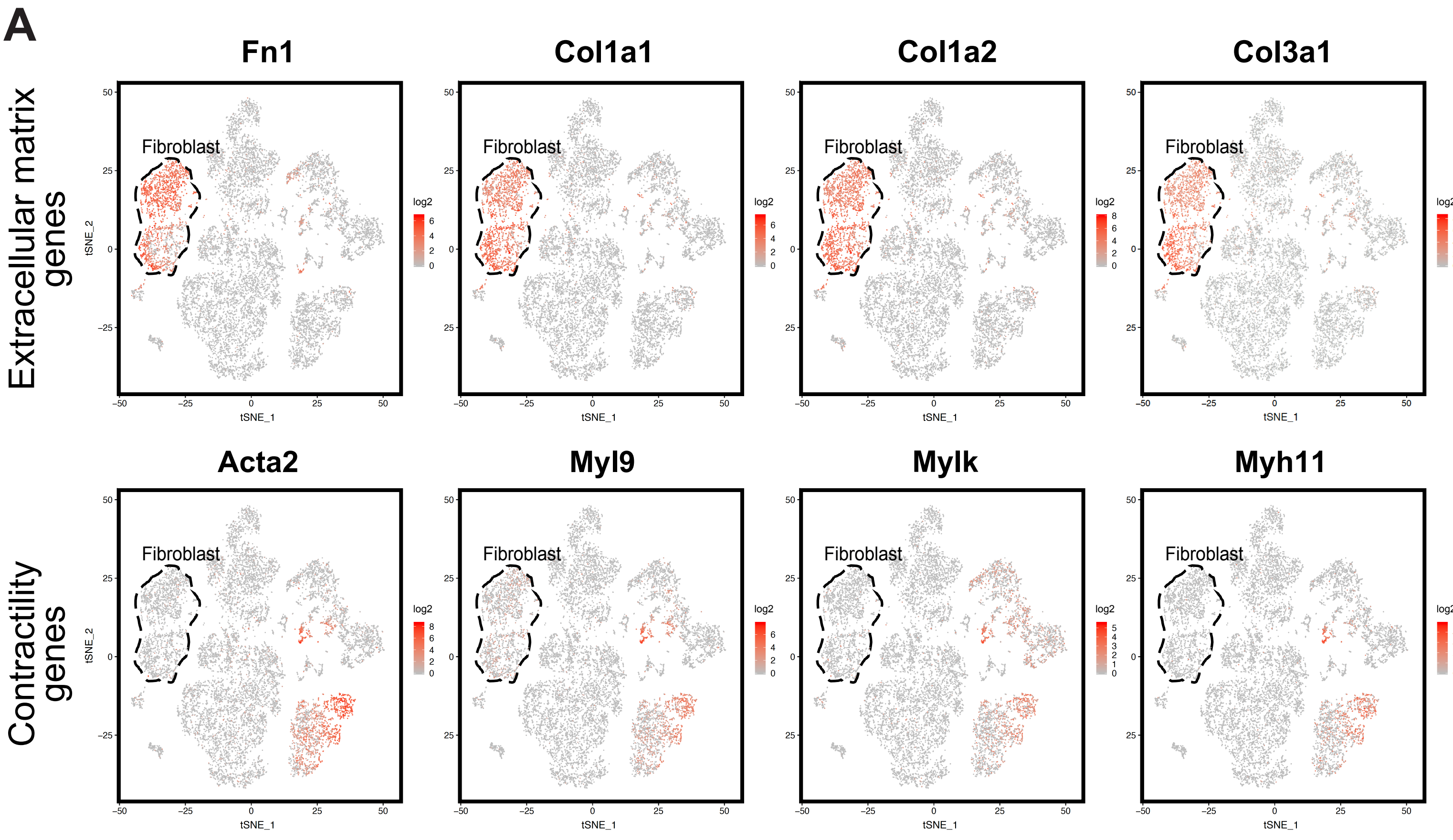
A
Fn1
Col1a1
Col1a2
Col3a1
Extracellular matrix genes
Fibroblast
log2
tSNE_1
tSNE_2
Acta2
Myl9
Mylk
Myh11
Contractility genes

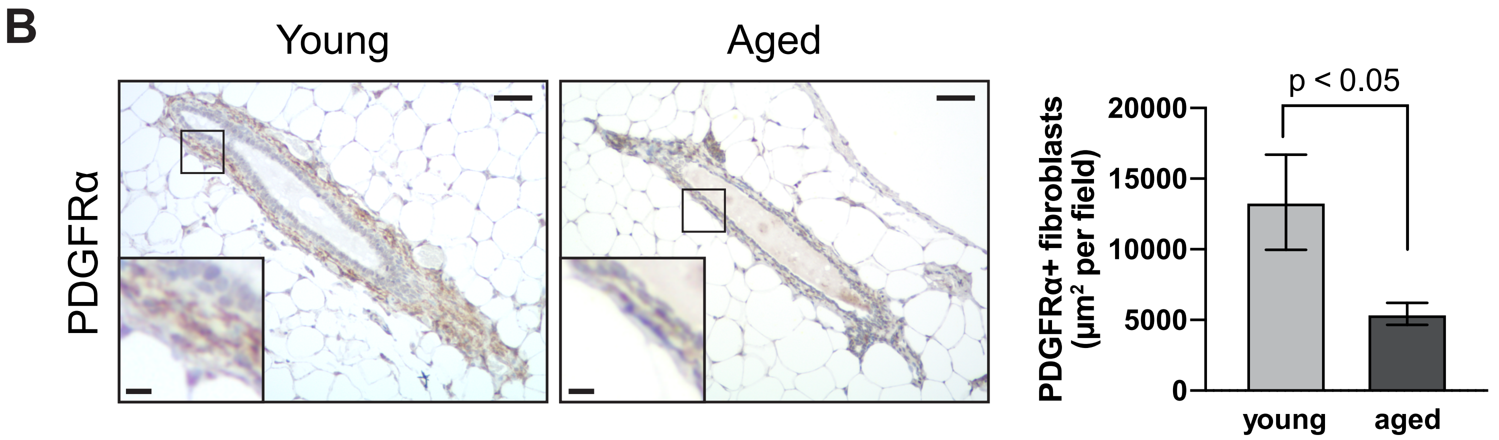
B
Young
Aged
PDGFRα
PDGFRα+ fibroblasts (µm² per field)
p < 0.05
20000
15000
10000
5000
0
young
aged

Figure S6 (related to Figure 6). Fibroblast Markers and Immunostaining Validation of Altered Fibroblast Abundance with Age

(A) The fibroblasts captured by scRNA-seq express high level of extracellular matrix genes (top), but not contractility genes (bottom).

(B) Representative PDGFRα IHC staining of fibroblasts and quantification in young and aged mammary glands. Scale bar = 50 μm or 10 μm (inset). Analysis was performed on at least five regions per gland at 10X magnification, with n = 6 animals per age group, using Student's t test.

Figure S7

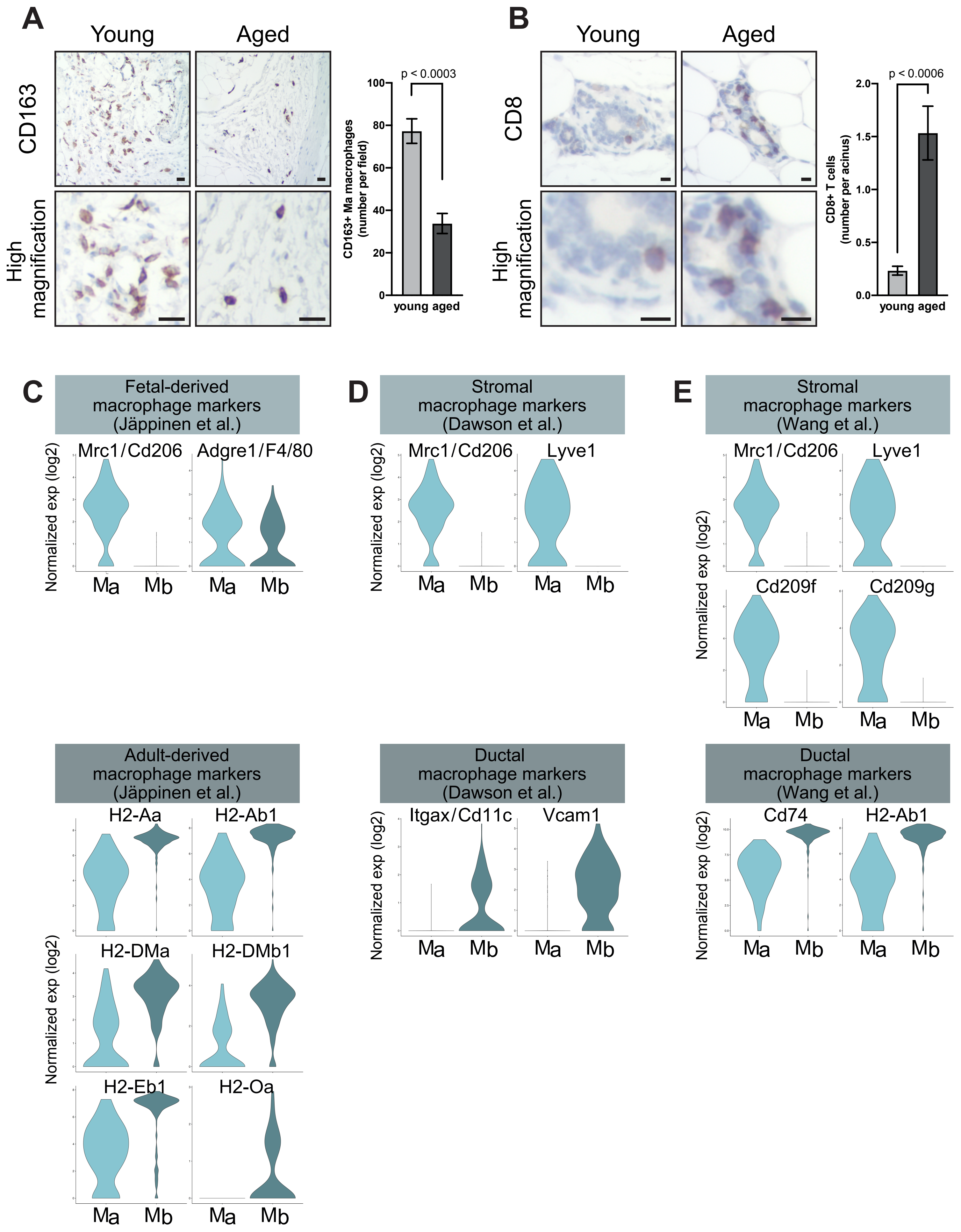

A
Young
Aged
CD163
High magnification
p < 0.0003
CD163+ Ma macrophages (number per field)
young aged
B
Young
Aged
CD8
High magnification
p < 0.0006
CD8+ T cells (number per acinus)
young aged
C
Fetal-derived macrophage markers (Jäppinen et al.)
Mrc1/Cd206
Adgre1/F4/80
Normalized exp (log2)
Ma Mb
D
Stromal macrophage markers (Dawson et al.)
Mrc1/Cd206
Lyve1
E
Stromal macrophage markers (Wang et al.)
Mrc1/Cd206
Lyve1
Cd209f
Cd209g
Adult-derived macrophage markers (Jäppinen et al.)
H2-Aa
H2-Ab1
H2-DMa
H2-DMb1
H2-Eb1
H2-Oa
Ductal macrophage markers (Dawson et al.)
Itgax/Cd11c
Vcam1
Ductal macrophage markers (Wang et al.)
Cd74
H2-Ab1

Figure S7 (related to Figure 7). Immunostaining Validation of Altered Immune Cell Abundance and Comparison of Macrophage Markers

(A) Representative IHC staining of CD163+ M_a macrophages and quantification in young and aged mammary glands. Scale bar = 25 µm. Analysis was performed on 3-6 ECM-rich stromal regions per gland at 10X magnification, with n = 6 animals per age group, using Student's t test.

(B) Representative IHC staining of CD8+ T cells and quantification in young and aged mammary glands. Scale bar = 10 µm. Analysis was performed on at least 15 acini per gland at 20X magnification, with n = 6 animals per age group, using Student's t test.

(C) M_a macrophages express higher levels of markers for fetal-derived macrophages, whereas M_b macrophages express higher levels of markers for adult-derived macrophages. Markers were reported by Jäppinen et al., 2019.

(D-E) M_a macrophages are distinguished by stromal macrophage markers, and M_b macrophages by ductal macrophage markers. Markers were reported by Dawson et al., 2020 (D) and Wang et al, 2020 (E).
